# Gender-transformative Bandebereho couples’ intervention to promote male engagement in reproductive and maternal health and violence prevention in Rwanda: Findings from a randomized controlled trial

**DOI:** 10.1371/journal.pone.0192756

**Published:** 2018-04-04

**Authors:** Kate Doyle, Ruti G. Levtov, Gary Barker, Gautam G. Bastian, Jeffrey B. Bingenheimer, Shamsi Kazimbaya, Anicet Nzabonimpa, Julie Pulerwitz, Felix Sayinzoga, Vandana Sharma, Dominick Shattuck

**Affiliations:** 1 Promundo-US, Washington, DC, United States of America; 2 Gender Innovation Lab, World Bank, Washington, DC, United States of America; 3 Milken Institute School of Public Health, The George Washington University, Washington, DC, United States of America; 4 Rwanda Men’s Resource Center, Kigali, Rwanda; 5 Maternal, Child and Community Health Division, Rwanda Ministry of Health, Rwanda Biomedical Center, Kigali, Rwanda; 6 HIV and AIDS Program, Population Council, Washington, Washington, DC, United States of America; 7 Harvard T.H. Chan School of Public Health, Harvard University, Boston, MA, United States of America; 8 Institute for Reproductive Health, Georgetown University, Washington, DC, United States of America; TNO, NETHERLANDS

## Abstract

**Background:**

Rigorous evidence of the effectiveness of male engagement interventions, particularly on how these interventions impact relationship power dynamics and women’s decision-making, remains limited. This study assessed the impact of the Bandebereho gender-transformative couples’ intervention on impact on multiple behavioral and health-related outcomes influenced by gender norms and power relations.

**Methods:**

We conducted a multi-site randomised controlled trial in four Rwandan districts with expectant/current fathers and their partners, who were randomised to the intervention (n = 575 couples) or control group (n = 624 couples). Primary outcomes include women’s experience of physical and sexual IPV, women’s attendance and men’s accompaniment at ANC, modern contraceptive use, and partner support during pregnancy. At 21-months post-baseline, 1123 men and 1162 partners were included in intention to treat analysis. Generalized estimating equations with robust standard errors were used to fit the models.

**Findings:**

The Bandebereho intervention led to substantial improvements in multiple reported outcomes. Compared to the control group, women in the intervention group reported: less past-year physical (OR 0.37, p<0.001) and sexual IPV (OR 0.34, p<0.001); and greater attendance (IRR 1.09, p<0.001) and male accompaniment at antenatal care (IRR 1.50, p<0.001); and women and men in the intervention group reported: less child physical punishment (women: OR 0.56, p = 0.001; men: OR 0.66, p = 0.005); greater modern contraceptive use (women: OR 1.53, p = 0.004; men: OR 1.65, p = 0.001); higher levels of men’s participation in childcare and household tasks (women: beta 0.39, p<0.001; men: beta 0.33, p<0.001); and less dominance of men in decision-making.

**Conclusions:**

Our study strengthens the existing evidence on male engagement approaches; together with earlier studies our findings suggest that culturally adapted gender-transformative interventions with men and couples can be effective at changing deeply entrenched gender inequalities and a range of health-related behavioral outcomes.

**Trial registration:**

ClinicalTrials.gov NCT02694627

## Introduction

Interest and investment in interventions engaging men in reproductive and maternal health and violence prevention in low- and middle-income countries has grown tremendously since the 1990s [1]. Male engagement interventions have evolved from seeking to involve men to overcome specific barriers, such as women’s limited decision-making power or access to health care, to be increasingly gender-transformative, engaging men and their partners to challenge the inequitable gender and power dynamics that give rise to such barriers [2,3]. However, rigorous evidence of the effectiveness of such interventions, particularly from low- and middle-income countries (LMIC), remains limited [3–5]. In addition, there is a need to measure how these interventions impact relationship power dynamics and women’s decision-making, to ensure male engagement approaches do not undermine women’s autonomy [3]. We undertook a randomized controlled trial (RCT) in Rwanda to assess the effectiveness of the Bandebereho (meaning “role model” in Kinyarwanda) couples’ intervention, a gender-transformative program for men and couples to promote men’s engagement in reproductive and maternal health, caregiving, and healthier couple relations. This study evaluates the intervention’s impact on multiple behavioral and health-related outcomes influenced by gender norms and power relations, which were addressed by the intervention.

Male engagement approaches in LMIC assessed by RCTs, whether targeting men alone or together with women, have ranged widely in scope, from those distributing information to intensive 50-hour participatory interventions, and in the degree to which they emphasize gender inequalities and power dynamics. Several trials have shown positive impacts on outcomes related to intimate partner violence (IPV) [6–10], family planning [8,11], and maternal health [12–14]. However, few studies have evaluated interventions addressing multiple outcomes, and fewer still have examined impact on household gender and power dynamics, such as on equitable decision-making [15] and men’s participation in household tasks [10,15].

Rwanda represents a strategic place to evaluate a gender-transformative male engagement approach. The country has made significant strides in maternal health by ensuring that nearly all women attend at least one antenatal care visit (99%) and deliver in a health facility (91%) [16]. The maternal mortality ratio fell from 476 per 100,000 live births in 2010 to 210 in 2015 [16]. However, 19% of married women still report an unmet need for family planning [16]. Women with limited household decision-making power are less likely to use contraceptives, and only 23% of Rwandan women are the primary decision-makers about their own health care [16]. Intimate partner violence (IPV) is also high: nationally, more than 20% of married women report having experienced physical or sexual violence from a partner in the past year [16]. Accordingly, the Government of Rwanda recognizes that further progress on reproductive and maternal health requires interventions with men and couples to promote equitable gender relations, women’s decision-making power, and reduced IPV [17].

## Methods

We conducted a two-arm multi-site randomized controlled trial to assess the impact of the Bandebereho couples’ intervention on our outcomes of interest. Couples were recruited from local communities in Karongi, Musanze, Nyaruguru and Rwamagana districts in Rwanda from February 19 to March 17, 2015, and followed over a period of 21 months for this study. Men were interviewed at three time points: baseline, 9 months post-baseline, and 21 months post-baseline; due to funding constraints, women were interviewed at only two time-points, at 9 and 21 months post-baseline. In order to highlight the longer-term effects of the intervention, this paper presents the findings from 21 months post-baseline. The Rwanda Men’s Resource Center, a local non-governmental organization implementing the intervention, selected the sites in collaboration with district authorities.

### Participants

For the study, a total of 1199 men were recruited from 48 pre-selected sites within 16 sectors (sub-district administrative units) in the districts selected for the intervention. Couples’ inclusion in the study was determined by men’s eligibility for the intervention. Eligible men were aged 21–35 years, married or cohabitating, expectant and/or fathers of children under-five years (based on self-reports), living within accessible distance of the meeting site, and were not previous Bandebereho intervention participants. The legal age of marriage (21 years) in Rwanda served as the minimum age for participation. Community volunteers facilitating the intervention worked with local community health workers to identify 25 eligible men in each of the 48 sites.

#### Sample size determination

We conducted a power analysis prior to study recruitment, in June 2014, to assess ability to detect intervention effects on selected outcomes. We calculated power for outcomes similar to those we planned to measure, including perpetration of IPV, communication about family planning, and gender attitudes, using estimates from the 2010 Rwanda Demographic and Health Survey [18] and the 2010 International Men and Gender Equality Survey [19], assuming an intervention sample size of 576 couples (48 groups with 12 couples each). We conservatively calculated power for a 5–10% difference in these outcomes, using a two-sided test of equality of two proportions with adjustment made for design effects due to clustering, assuming an intra-class correlation coefficient of less than 0.1 and an alpha of 0.05. We found that the indicators would provide enough power (between 65% and 99%, depending on the indicator).

#### Randomization and masking

Randomization to either the intervention or control group was done after baseline interviews using the individual as the unit of randomization. In each of the 48 sites, 12 men were randomly assigned to the intervention arm (n = 575), and the remaining men were assigned to the control arm (n = 624). Laterite, an independent firm collecting the data, randomized the participants using a random number generator in Stata 12. Bandebereho community facilitators notified men of their assignment. All recruited men remained eligible for randomization to the intervention regardless of participation in baseline data collection: in total, 1199 men out of a possible 1200 were invited to participate in the study, and 1195 men were surveyed at baseline. After randomization, we discovered two facilitators from neighbouring sites had mistakenly recruited the same participant, who was randomized twice into the intervention, resulting in 575 men randomized to the intervention, out of a possible 576.

Due to the nature of the intervention, it was not possible to mask group assignments for participants. Group assignment was also not masked for the data collectors, who were not involved in the intervention. Specific measures to track spillover effects were included in the study design because the intervention and control groups reside in the same communities, and the intervention promotes community outreach. However, we posited that the effects of participation in the intensive intervention would outweigh any spillover effects and that such effects would result in underestimation, rather than over-estimation, of the intervention’s impact.

### Procedure

Structured questionnaires were administered to male participants at baseline from 19 February to 17 March 2015. As noted above, men’s partners were not surveyed at baseline due to funding constraints. After the baseline and randomization, the Bandebereho intervention was implemented with the intervention group from March to July 2015. Follow-up surveys were conducted with men and their current partners at 9 months, from 9 November to 17 December 2015 (4 months post-intervention), and again at 21 months, from 7 November to 23 December 2016 (16 months post-intervention). At 21 months, 99.6% of the women surveyed were the same partner identified at baseline. At each follow-up, the participation of both partners was not required: either partner could be interviewed even if the other was unavailable. Study participants received a 2000 Rwandan franc transport stipend (about US$2.50) for each interview. Sex-matched interviewers from Laterite, who had no involvement in the intervention, conducted the interviews in Kinyarwanda in centrally located settings such as schools. Data were collected on password-protected tablets.

All efforts were made to ensure study participant safety, privacy and comfort. Informed consent was obtained from all participants. The interviewer reviewed the consent form with each participant and answered any questions; participants signed a written consent if they were literate, or provided a thumbprint if they were not. The study was conducted in accordance with international ethical guidelines on researching violence against women, including not interviewing members of the same household about IPV [20]. At follow-up, we asked women about their experiences of IPV, but did not ask men about violence perpetration, and men were not informed of the inclusion of questions about violence in the women’s questionnaire. To minimize risk of harm, we obtained men’s consent to disclose their participation in the study before contacting their partners, and interviews with men and women were conducted on different days. Participants were offered a list of locally available support services after the interviews. Male and female interviewers received ethics and safety training and a female Rwandan counselor met with the female interviewers before, during and after data collection.

The study protocol received approval from the Rwanda National Health Research Committee (25 August 2014, NHRC/2014/PROT/0193), the Rwanda National Ethics Committee (24 October 2014, 346/RNEC/2014), and the Rwanda National Institute of Statistics (9 February 2015, 0082/2015/NISR) prior to study recruitment and data collection. As per Rwandan government requirements, study approval was renewed annually with the Rwanda National Ethics Committee (19 October 2015, 338/RNEC/2015; 21 October 2016, 883/RNEC/2016) and the Rwanda National Institute of Statistics (2 November 2015, 0794/2015/10/NISR; 27 October 2016, 0806/2016/10/NISR). The trial was retrospectively registered at clinicaltrials.gov on February 29, 2016 (NCT02694627) after study enrolment began in February 2015, but before collection of the 21-month follow-up data (reported here) or study completion. The delay in trial registration was due to the authors’ lack of awareness of this requirement for journal publication. We registered the study as soon as we were aware of this requirement. No major changes to the study protocol or study outcomes were made. The authors confirm that all related trials to this intervention were registered; there are no ongoing trials related to this study.

#### Study retention

**Fig 1:**
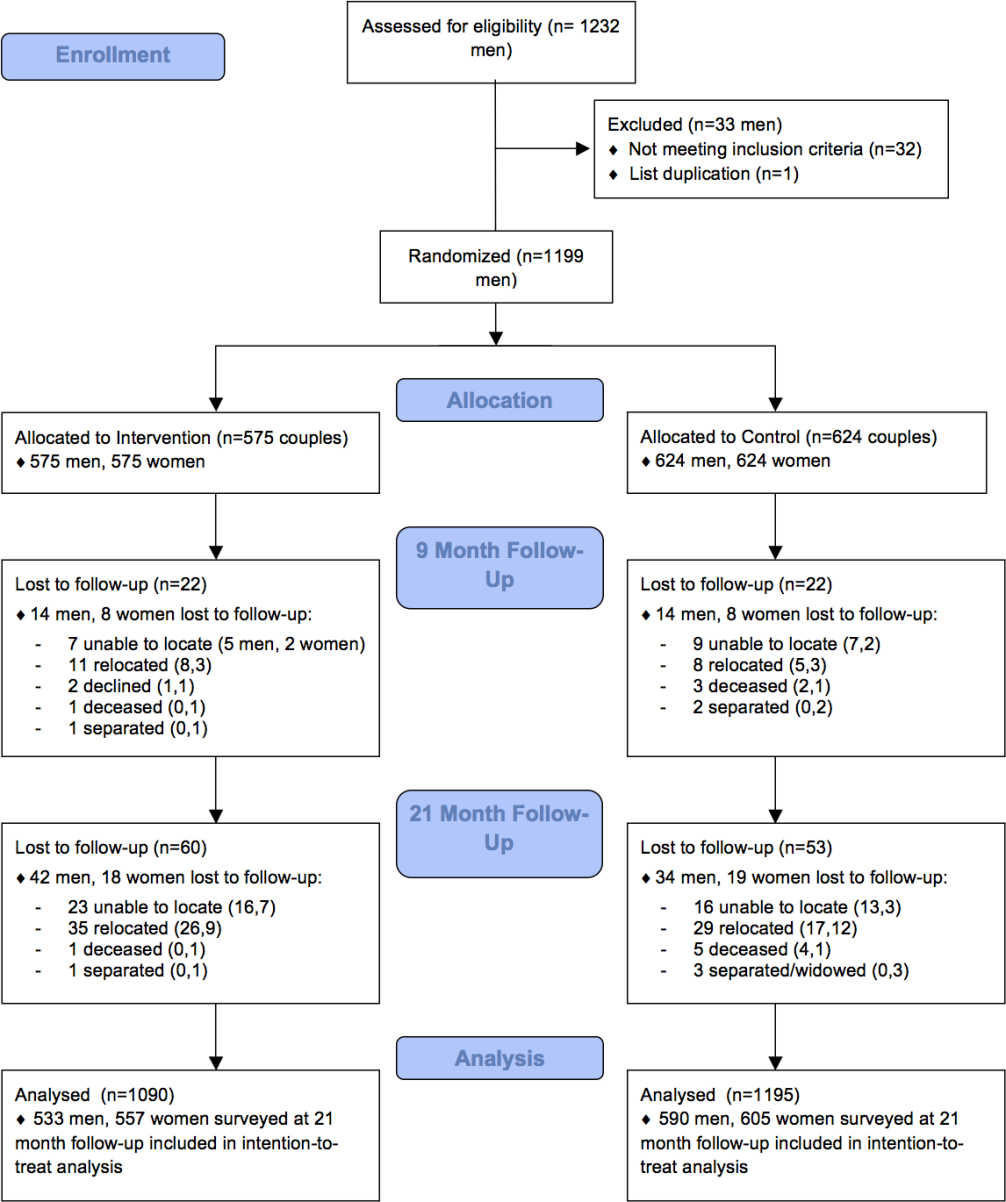
Trial profile.

At 21-month follow-up, 1123 men (94% of the sample) and 1162 women (97%) were surveyed. Respondent attrition was slightly higher for men in the intervention group compared to the control group (7.3 vs. 5.4%), and was essentially identical for women (3.1 vs. 3.0%) (Fig 1). Reasons for loss to follow-up were predominantly inability to find participants due to relocation and respondent unavailability. Men who dropped out were more likely to be out of work and looking for work at baseline compared to men who remained in the study. All available data were included in analyses.

#### The Bandebereho intervention

The Bandebereho couples’ intervention engaged men and their partners in participatory, small group sessions of critical reflection and dialogue. The Rwanda Men’s Resource Center (RWAMREC), a local Rwanda non-governmental organization, implemented the intervention as part of MenCare+, a four-country initiative to engage men in sexual, reproductive, and maternal health. The MenCare+ program was coordinated by Rutgers and Promundo, and financed by the Dutch Ministry of Foreign Affairs. In Rwanda, the MenCare+ program was known as Bandebereho, or “role model”, as it aimed to transform norms around masculinity by demonstrating positive models of fatherhood.

The intervention used a structured 15-session curriculum adapted from Program P, an open source manual for engaging men in maternal and child health, created by Promundo, CulturaSalud, and REDMAS (2013) which includes a curriculum for fathers/couples, resources for designing health provider training and community campaigns [21]. Men participating in the Bandebereho intervention were invited to 15 sessions (maximum 45 hours) and their partners to 8 (maximum 24 hours). Sessions addressed: gender and power; fatherhood; couple communication and decision-making; IPV; caregiving; child development; and male engagement in reproductive and maternal health (See Table 1 for details on curriculum content by session.)

**Table 1:** Bandebereho intervention session overview.

Session	Objectives	Participants
1. Gender Equality	To create a space of trust and confidentiality; to discuss the differences between sex and gender; and to reflect on how gender norms influence the lives and relationships of women and men.	Couples
2. Becoming a Father	To reflect on men’s concerns about becoming a father, and to discuss the benefits that being an involved father can bring to men’s children, their partners and themselves.	Men
3. Pregnancy	To inform expectant fathers and mothers about the biological process of pregnancy, including what men can do to ensure the health of the mother and fetus during and after birth, and to address many of their concerns.	Couples
4. Supporting Your Pregnant Partner	To help men and women understand how men can support women during pregnancy and to discuss the role of men in accompanying their partners to antenatal care visits.	Couples
5. Childbirth	To share ideas and experiences about the role of the father during birth, and to prepare men to accompany their partners during delivery, including the importance of bonding with their new sons and daughters.	Couples
6. Family Planning	To reflect upon the benefits of family planning and the value of couple communication in this process and provide information on different contraceptive methods.	Couples
7. Caring for a Baby	To learn about a baby’s care needs and reflect upon men’s capacity to satisfy these needs and to reflect on how gender stereotypes influence a father and mother’s behavior towards their children.	Men
8. My Parents' Impact	To encourage men to reflect on their parents’ influences on their own lives and reflect on the future they envision for their children, including how to use the positive influences and avoid the negative aspects so they do not repeat themselves.	Men
9. Identifying Violence	To identify the different forms of violence that men perpetrate, or that are committed against them and to become familiar with the different types of violence that exist.	Men
10. Gender-based Violence	To discuss gender-based violence and the law and to reflect on the ways that men can break the culture of silence surrounding violence in families and romantic relationships.	Couples
11. Resolving Conflict	To identify non-violent ways to resolve conflict and to reflect on the importance of strong relationships and social networks when we face difficult moments as fathers and husbands.	Men
12. Alcohol and Drug Abuse	To encourage discussion about the risks and consequences of alcohol and drug abuse and how men can help each other in reducing the harm caused by drugs and alcohol.	Men
13. Raising Children	To make connections between the long-term goals fathers and mothers have for their children (ages 0–5) and how harsh discipline affects those goals.	Couples
14. Sharing Responsibilities at Home	To reflect on how gender roles influence the distribution of care work within the household, and to encourage a more equitable distribution of childcare and housework between men and women. To also promote discussion about household finances and help couples develop a household budget.	Couples
15. Reflection	To reflect on the experiences participants have had in the group sessions and make a commitment to be a more involved father.	Men

Promundo and RWAMREC adapted the curriculum between May 2013 and January 2014, informed by formative research, and input from the Rwanda Ministry of Health, which approved the curriculum for implementation, and from community pilot implementations.

The intervention draws on sociological theories of gender and masculinities that highlight how gender inequalities are reproduced–or transformed–through “everyday interactions in [the] home” [22,23]. The intervention creates a structured space for men and women to: 1) question and critically reflect on gender norms and how these shape their lives; 2) rehearse equitable and non-violent attitudes and behaviors in a comfortable space with supportive peers; and 3) internalize these new gender attitudes and behaviors, and apply them in their own lives and relationships. We hypothesize that becoming aware of inequalities, reflecting on the costs of rigid norms, and learning and practicing new skills (e.g. couple communication and joint decision-making) in a safe, non-judgmental peer environment, can lead to changes across a range of health and relationship behaviors.

Community volunteers (local fathers) met with the same group of 12 men/couples on a weekly basis. The volunteers received a two-week training, material support, and refresher trainings from RWAMREC. Local nurses and police officers co-facilitated the sessions on pregnancy, family planning, and local laws, respectively. Sessions were conducted in local schools and administrative offices. A transportation stipend of 2000 Rwandan francs (about US$2.50) was provided to men/couples for each session attended. RWAMREC staff monitored implementation of the group sessions and mentored the facilitators. Three intervention cycles, each with 570–576 couples, were implemented between March 2014 and July 2015. This study assessed the third cycle, in which men attended on average 14.1 out of 15 sessions, and women 6.8 out of 8 sessions. The control group received no group intervention, though it did have access to community activities and campaigns related to the broader MenCare+ project.

### Measures

We assessed five sets of outcomes specifically targeted by the intervention, each captured through multiple variables: (1) reproductive and maternal health behaviors, including men’s participation in ANC visits; (2) women’s experiences of IPV; (3) use of physical punishment against children; (4) gendered division of childcare and household tasks; and (5) men’s dominance in household decision-making. Table 2 summarizes the key outcome measures.

**Table 2:** Outcome measures.

	Variable	Respondents	Instrument, Indicators	Coding	Expected direction of difference in intervention vs. control group
**Reproductive and maternal health behaviors**	Mean number of ANC visits women attended	Women	Women were asked how many ANC visits they attended during their current pregnancy (if applicable) and during their most recent pregnancy.	Continuous. Variable was coded to include visits during most recent or current pregnancy.	Higher
	Mean number of ANC visits accompanied by man	Women; Men	Women were asked how many times their partner accompanied them to ANC visits; men were asked how many times they accompanied their partner. Accompaniment typically meant waiting in the health facility or attending part of the visit with the partner.	Continuous. Variable was coded to include visits during most recent or current pregnancy.	Higher
	Perceived partner support during pregnancy	Women	Women were asked if during their current or most recent pregnancy their partner demonstrated any of six types support: 1) provided financial support; 2) did any household tasks she normally does; 3) prepared food or made sure she ate nutritious food; 4) encouraged her to take care of herself; 5) provided care or emotional support; or 6) provided spiritual support or guidance.	Continuous, ranging from 0 to 1; composite is a mean of yes = 1 and no = 0 responses to the indicators described at left.	Higher
	% Used modern contraception	Women; Men	Women and men were asked about their or their partner’s current use of any modern contraceptive method (e.g. implant, injection, male or female condom, pill, IUD, vasectomy, hysterectomy).	Binary, coded 1 if using any of the modern contraceptive methods, 0 if answered no to all. Included the full sample (whether pregnant or not), consistent with other measures of contraceptive prevalence.	Higher
**Experiences of intimate partner violence**	Experienced physical violence from partner in past 12 months	Women	Women were asked five items adapted from the WHO multi-country study [24], regarding how many times in the past 12 months their partner had: 1) slapped them or threw something at them that could hurt them; 2) pushed or shoved them; 3) hit them with a fist or with something else that could hurt them; 4) kicked, dragged, beat, choked or burned them; 5) threatened to use or actually used a knife or stick against them. Responses ranged from 0 = never, 1 = once, 2 = a few times, and 3 = frequently.	Binary, coded 1 if responded once or more often to any of the five items listed at left, 0 if never to all.	Lower
	Experienced sexual violence by partner in past 12 months	Women	Women were asked two items regarding how many times in the past 12 months: 1) their partner had forced them to have sex when they did not want to; and 2) they had consented to sex out of fear of what their partner might do if they refused. Responses ranged from 0 = never, 1 = once, 2 = a few times, and 3 = frequently.	Binary, coded 1 if responded once or more often to either of the two items listed at left, 0 if never to all.	Lower
**Use of physical punishment against children**	Used physical punishment on one’s child in past month	Women; Men	Men and women were asked seven items adapted from the Multiple Indicator Cluster Survey (MICS) child discipline module,^a^ including whether or not they: 1) shook the child; 2) spanked, slapped or hit the child on the bottom with a bare hand; 3) hit the child on the bottom or elsewhere on the body with something like a belt, stick or other hard object; 4) hit or slapped the child on the face, head, or ears; 5) hit or slapped the child on the hand, arm, or legs; 6) beat the child up, meaning hit the child over and over as hard as one could; and 7) made the child kneel on the ground for a period of time.	Binary, coded 1 if responded yes to any, 0 if no to all.	Lower
**Gendered division of childcare and household tasks**	Sharing of childcare and household tasks	Women; Men	Men and women were asked how they divided 6 childcare and household tasks with their partner: 1) washing clothes/laundry; 2) cleaning the house and surroundings; 3) cooking for the household; 4) making the bed; 5) providing daily care of children; and 6) bathing children. Responses ranged from 1 = woman always does the task, 3 = shared equally or done together, 5 = man always does the task.	Continuous scale of mean score across the items, ranging from 1 to 5, with 5 indicating men's greater participation.	Higher
	Time spent on childcare and household tasks	Women; Men	This variable represents the number of hours per day that men or women spent on the 6 tasks in the past week. Respondents were asked on how many days in the previous week they did each task, and how much time (in hours or fractions of hours) on average they spent on the task on each of those days.	Continuous, representing hours spent per day: time spent per day for each task was multiplied by the reported days per week. The sum of the total hours per week for all tasks was divided by 7 to produce the hours per day variable. “Not applicable” responses were coded as 0.	Lower for women; higher for men
**Men’s dominance in household decision-making**	Man has final say on household’s weekly/monthly income and expenses	Women; Men	Men and women were asked who has the final say in making the decision: self; partner; both have the same say; someone else; don’t know.	Binary, coded 1 if man had final say, 0 if decision made by woman, made jointly, or respondent didn't know.	Lower
	Man has final say on how many children to have or spacing of children	Women; Men	Men and women were asked who has the final say in making the decision: self; partner; both have the same say; someone else; don’t know.	Binary, coded 1 if man had final say, 0 if decision made by woman, made jointly, or respondent didn't know.	Lower

^a^ MICS surveys can be accessed at http://mics.unicef.org/surveys

#### Statistical analysis

We compared men’s characteristics at baseline using frequencies and descriptive statistics. To estimate the effects of the intervention on outcomes measured at 21-month follow-up, we conducted intention-to-treat analysis using regression models with normal, Bernoulli, and Poisson response distributions and identity, logistic, and log link functions. We used generalized estimating equations to fit the models, and used robust standard errors with clustering by facilitator for hypothesis testing and confidence interval construction. For each outcome we fit both unadjusted and adjusted models; the latter included controls for age, education, and baseline socio-economic status (defined as having basic needs met). All analyses were conducted using Stata/SE 14. In our presentation of results, we use standard abbreviations for statistical terminology, including: SD–standard deviation; CI–confidence interval; OR–odds ratio; and IRR–incidence rate ratio.

## Results

Table 3 presents the baseline characteristics of men by intervention and control groups. Independent samples t-tests and chi-squared tests of association, as appropriate, showed no statistically significant differences in baseline characteristics between the intervention and control group respondents. Men reported a mean age of 28.7 and their partners’ mean age of 26.6 years. More than 60% of men had only primary education or less, and less than a third reported always being able to afford basic items. Nearly all men were employed, with the majority of those self-employed. Three quarters had biological children, and about two thirds were expecting a child. Women were not surveyed at baseline.

**Table 3:** Men’s characteristics at baseline.

	Control Group	Intervention Group	All
	(n = 624)	(n = 571)	(n = 1195)
Age (years): mean (SD)	28.62 (3.76)	28.70 (3.58)	28.65 (3.68)
Age of partner (men's reports)	26.53 (4.05)	26.72 (4.14)	26.62 (4.09)
**Level of education**			
None	63 (10.10%)	49 (8.58%)	112 (9.37%)
Some primary	321 (51.44%)	318 (55.69%)	639 (53.47%)
Primary complete	147 (23.56%)	130 (22.77%)	277 (23.18%)
Secondary, vocational or higher	93 (14.90%)	74 (12.96%)	167 (13.97%)
**Employment status**			
Employed/earning a wage	54 (8.65%)	65 (11.38%)	119 (9.96%)
Self-employed	564 (90.38%)	503 (88.09%)	1067 (89.29%)
Out of work and looking for work	6 (0.96%)	3 (0.53%)	9 (0.75%)
**Household can afford basic items**			
Never or sometimes	245 (39.26%)	218 (38.18%)	463 (38.74%)
Often	185 (29.65%)	175 (30.65%)	360 (30.13%)
Always	194 (31.09%)	178 (31.17%)	372 (31.13%)
Has biological children	474 (75.96%)	434 (76.01%)	908 (75.98%)
Number of children, mean (SD)	1.45 (0.67)	1.51 (0.75)	1.48 (0.71)
Expecting a child at baseline	399 (64.15%)	372 (65.15%)	771 (64.63%)
**Men's participation in RMH**			
# ANC visits accompanied by men mean, (SD)	1.50 (0.94)	1.42 (0.92)	1.46 (0.93)
% Currently using modern contraception	356 (57.05%)	328 (57.44%)	684 (57.24%)
**Gendered division of childcare and household tasks**			
Sharing of tasks mean, (SD)	1.83 (0.43)	1.85 (0.43)	1.84 (0.43)
**Household decision-making**			
Man has final say on household weekly/monthly income and expenses	361 (58.04%)	338 (59.19%)	699 (58.59%)
Man has final say in how many children to have or spacing of children	271 (43.57%)	234 (41.34%)	505 (42.51%)

Notes: Baseline characteristics are only available for men, as women were not interviewed at baseline. There are no statistically significant differences between intervention and control arms at baseline. Questions related to physical punishment against children were not asked at baseline, and questions related to the frequency of tasks were measured differently at baseline compared to follow-up and are therefore not included.

All statistics are n (%) unless otherwise specified.

Men’s reports on key outcomes at baseline were similar across groups. Men reported attending on average 1.50 ANC visits with their partners during their current or most recent pregnancy (SD 0.94), and 57% reported using modern contraception with their partner. A mean score of 1.89 on a scale of 1 to 5 of the gendered division of childcare and household tasks reflects low participation by men in these tasks. Nearly 60% reported that they had the final say on decisions regarding the household’s income and expenses, and about 43% had the final say on how many children to have or the spacing of children.

Table 4 presents the effect of the intervention on the outcomes of interest. Results from analyses adjusted for age, level of education, and socio-economic status, are presented in the text. Outcomes related to IPV were only asked of women. At 21-month follow-up, more than half of women in the control group (56.53%) reported experiencing physical violence from the partner in the previous 12 months, compared to one-third of women in the intervention group (33.33%) (OR 0.37, 95% CI 0.28–0.49 p<0.001). Similarly, rates of sexual violence from a partner were 60·17% among women in the control group compared to 35.01% in the intervention group (OR 0.34, 95% CI 0.25–0.48, p<0.001).

**Table 4:** Effect of the intervention on outcomes, 21-month follow-up.

	Summary Statistics			Intervention effect	
	Control	Intervention	All	Unadjusted	Adjusted*
	(n = 590 men, 605 women)	(n = 533 men, 557 women)	(n = 1123 men, 1162 women)		
**Experiences of intimate partner violence**					
Experienced physical violence from partner in past 12 months (women’s reports)	342 (56.53%)	186 (33.33%)	528 (45.40%)	OR = 0.38 (0.29–0.50) p<0.001	OR = 0.37 (0.28–0.49) p<0.001
Experienced sexual violence by partner in past 12 months (women’s reports)	364 (60.17%)	195 (35.01%)	559 (48.11%)	OR = 0.36 (0.25–0.50) p<0.001	OR = 0.34 (0.25–0.48) p<0.001
**Reproductive and maternal health behaviour**s					
# ANC visits (women’s reports)	3.11 (1.22)	3.40 (1.09)	3.25 (1.17)	IRR = 1.09 (1.05–1.14) p<0.001	IRR = 1.09 (1.05–1.14) p<0.001
# ANC visits accompanied by men (men’s reports)	1.57 (0.92)	2.09 (1.03)	1.82 (1.01)	IRR = 1.33 (1.23–1.45) p<0.001	IRR = 1.33 (1.23–1.45) p<0.001
# ANC visits accompanied by men (women’s reports)	1.15 (0.68)	1.71 (1.02)	1·42 (0·90)	IRR = 1.49 (1.35–1.64) p<0.001	IRR = 1.50 (1.36–1.65) p<0.001
% Currently using modern contraception (men's reports)	382 (64.86%)	401 (75.38%)	783 (69·85%)	OR = 1.65 (1.24–2.21) p = 0.001	OR = 1.65 (1.24–2.20) p = 0.001
% Used modern contraception (women’s reports)	366 (60.50%)	390 (69.89%)	756 (65.00%)	OR = 1.52 (1.15–2.01) p = 0.003	OR = 1.53 (1.15–2.04) p = 0.004
Perceived support during pregnancy (women’s reports)	0.74 (0.34)	0.92 (0.20)	0.82 (0.30)	Beta = 0.18 (0.13–0.22) p<0.001	Beta = 0.18 (0.13–0.23) p<0.001
**Use of physical punishment against children **					
Men’s use of physical punishment (men’s reports)	387 (67.30%)	303 (57.71%)	690 (62.73%)	OR = 0.66 (0.50–0.89) p = 0.006	OR = 0.66 (0.50–0.88) p = 0.005
Women’s use of physical punishment (women’s reports)	467 (79.15%)	374 (68.25%)	841 (73.90%)	OR = 0.56 (0.40–0.79) p = 0.001	OR = 0.56 (0.41–0.79) p = 0.001
**Gendered division of childcare and household tasks**					
Sharing of tasks (men’s reports)	1.77 (0.48)	2.10 (0.50)	1.92 (0.52)	Beta = 0.33 (0.26–0.41) p<0.001	Beta = 0.33 (0.26–0.41) p<0.001
Sharing of tasks (women’s reports)	1.65 (0.48)	2.04 (0.51)	1.83 (0.53)	Beta = 0.39 (0.31–0.47) p<0.001	Beta = 0.39 (0.31–0.47) p<0.001
Time spent on tasks- Hours per day (men’s reports)	1.40 (2.09)	2.26 (2.38)	1.80 (2.27)	Beta = 0.86 (0.49–1.23) p<0.001	Beta = 0.86 (0.50–1.22) p<0.001
Time spent on tasks- Hours per day (women’s reports)	8.34 (5.30)	8.34 (5.05)	8.34 (5.18)	Beta = 0.002 (-0.60–0.61) p = 0.99	Beta = 0.07 (-0.53–0.68) p = 0.81
**Men’s dominance in household decision-making**					
Man has final say on weekly/ monthly income and expenses (men’s reports)	409 (70.27%)	241 (45.47%)	650 (58.45%)	OR = 0.35 (0.26–0.49) p<0.001	OR = 0.35 (0.25–0.48) p<0.001
Man has final say on weekly/ monthly income and expenses (women’s reports)	474 (78.74%)	309 (56.08%)	783 (67.91%)	OR = 0.35 (0.26–0.46) p<0.001	OR = 0.31 (0.24–0.42) p<0.001
Man has final say in how many children to have or spacing of children (men’s reports)	278 (49.03%)	168 (31.94%)	446 (40.81%)	OR = 0.49 (0.37–0.64) p<0.001	OR = 0.48 (0.36–0.63) p<0.001
Man has final say in how many children to have or spacing of children (women’s reports)	284 (47.81%)	192 (34.91%)	476 (41.61%)	OR = 0·59 (0.47–0.73) p<0.001	OR = 0.57 (0.45–0.72) p<0.001

* Analyses adjusted for men’s and women’s self-reported current age and level of education, and men’s reports of socio-economic status at baseline (defined as having basic needs met).

Women in the intervention group reported attending slightly more ANC visits compared to women in the control group (IRR 1.09, 95% CI 1.05–1.14). Both women and men in the intervention group reported higher mean rates of men’s participation in ANC visits compared to women (IRR 1.50, 95% CI 1.36–1.65, p<0.001) and men (IRR 1.33, 95% CI 1.23–1.45, p<0.001) in the control group. Similarly, both women and men in the intervention group reported greater use of modern contraception compared to the control group (OR 1.53, 95% CI 1.15–2.04, p = 0.004 for women; OR 1.65, 95% CI 1.24–2.20, p = 0.001 for men). Women in the intervention group reported higher levels of partner support during pregnancy (mean 0.92, SD 0.20 on a scale from 0 to 1) compared to women in the control group (mean 0.74, SD 0.34).

Physical punishment of children was reported by 79.15% of women and 67.30% of men in the control group, compared to 68.25% of women (OR 0.56, CI 0.41–0.79, p = 0.001) and 57.71% of men in the intervention group (OR 0.66, 95% CI 0.50–0.88, p = 0.005).

Intervention group participants reported higher levels of men’s participation in childcare and household tasks compared to participants in the control group (Beta 0·39, 95% CI 0·31–0.47, p<0.001 for women; beta 0.33, 95% CI 0.26–0.41, p<0.001 for men). While men in the intervention group reported spending more hours on these tasks compared to men in the control group (Beta 0.86, 95% CI 0.50–1.22, p<0.001), there were no statistically significant differences in women’s time spent on these tasks between the intervention and control groups.

There were large differences in reports of men’s dominance in decision-making between control and intervention groups. In the intervention group, 56.08% of women (OR 0.31, 95% CI 0.24–0.42, p<0.001) and 45.47% of men (OR 0.35, 95% CI 0.25–0.48, p<0.001) reported that the man had the final say on decisions regarding the household’s income and expenses, compared to 78.74% and 70.27% in the control group, respectively. For decisions about having children or the spacing of children, 34·91% of women (OR 0.57, 95% CI 0.45–0.72, p<0.001) and 31.94% of men (OR 0.48, 95% CI 0.36–0.63, p<0.001) in the intervention group reported that the man had the final say compared to 47.81% and 49.03% in the control group, respectively.

Since women were not interviewed at baseline, it was not possible to adjust for baseline values for all indicators. However, including baseline values for the available men’s indicators (accompaniment to ANC, contraceptive use, and decision-making variables) yields similar results (not shown).

## Discussion

The Bandebereho intervention led to substantial improvements in multiple reported outcomes, including women’s experience of physical and sexual IPV, women’s ANC attendance, men’s accompaniment at ANC, modern contraceptive use, and partner support during pregnancy. Importantly, the intervention also led to reductions in men’s dominance in household decision-making and improvements in the household division of labor. Notably, our findings at 21-months are similar to those at 9 months (not reported here), suggesting sustained effects over time. Our study strengthens the existing evidence on male engagement approaches; together with earlier studies our findings suggest that culturally adapted gender-transformative interventions with men and couples can be effective at changing deeply entrenched inequalities and a range of health-related behavioral outcomes.

Our IPV findings are especially compelling, with a significant reduction in the likelihood of both physical and sexual violence from a partner reported by women in the intervention group compared to the control group. While previous trials have demonstrated reductions in reported physical IPV [7], sexual IPV [7, 8], and men’s perpetration of IPV [6], the degree of IPV risk reduction we report is seldom achieved in rigorously evaluated interventions [25]. Encouragingly, our study also demonstrated an impact on women’s and men’s physical punishment of children, despite this topic being of relatively limited focus in the intervention curriculum. Consistent with global literature, we find higher rates of women’s use of harsh punishment of children compared to men [26], likely due to the disproportionate amount of time they spend caring for children. Our study strengthens evidence from recent non-trial studies of male engagement approaches, such as the evaluation of REAL Fathers in Uganda that found reductions in both harsh punishment of children and IPV perpetration, by focusing on women’s reports of experiencing violence [27].

This is the first trial of a male engagement intervention, to our knowledge, to demonstrate at least a modest impact on women’s ANC attendance [3,28]. We also show that the Bandebereho intervention increased men’s accompaniment to ANC and their provision of support during pregnancy, factors which may be associated with women’s increased care seeking. Research by Påfs and colleagues in Rwanda has found that and men saw their presence at maternal health services as important for ensuring their partners received quality care [29]. Previous male engagement trials in LMIC have demonstrated positive impact on partner assistance during obstetric emergencies [12], women’s attendance at postpartum visits [13], care seeking for problems during pregnancy and hospital delivery [14], and mixed results at increasing male partner accompaniment to ANC [30]. Non-trial research has found that male partner support is associated with women’s antenatal attendance, birth preparedness and use of a skilled birth attendant [3,28]. Our results strengthen this evidence base.

The Bandebereho intervention also led to a substantial increase in the likelihood of reported modern contraceptive use. We hypothesize that in addition to providing information about contraceptives, the intervention strengthened couple communication, support, and joint decision-making, which positively affect contraceptive behavior. Brunie and colleagues have reported that Rwandan women whose partners support family planning have more than 8 times greater odds of using contraceptives than women whose partners did not, with spousal communication a facilitating factor [31]. Our findings complement evidence from the CHARM and Malawi Male Motivator trials of gender-transformative family planning interventions, which also demonstrated increased modern contraceptive use and couple communication about contraception [8,11]. In both the intervention and control groups, men reported slightly higher rates of modern contraceptive use compared to women. This discrepancy may be due to several reasons, such as men’s lack of awareness of their partner’s use/non-use of contraception at the specific time-point, or to a stronger social desirability bias among men, in both control and intervention groups. Further research could explore this discrepancy.

The Bandebereho intervention demonstrated a reduction in men’s dominance in household decision-making, which is associated with negative health-related outcomes for women and children [32]. Our findings suggest that by emphasizing joint decision-making through skills-based activities and by creating spaces for couple communication, the intervention was successful at targeting underlying, unequal gendered power dynamics. Qualitative research by Doyle and colleagues, in an earlier cycle of the Bandebereho intervention, found that men’s participation in the intervention with their partner led to greater respect and value for their partners’ opinions [33]. Future research should seek to understand how decision-making patterns change, and how interventions that encourage and build joint decision-making skills affect women’s own decision-making power.

The study is unique in measuring both the distribution of tasks between partners and in collecting detailed time use data from both partners, while other studies have shown only changes in men’s participation in these tasks [10, 15]. Encouragingly, the intervention led to changes in the household division of labor, with both men and women reporting greater sharing of childcare and household tasks, and men reporting more time spent on these tasks. Critical reflection on the gendered division of labor and its costs to the family–and skill building around the care of infants–were a core focus for the intervention, leading to increased men’s participation. However, despite greater male involvement, we did not find a reduction in women’s time spent on these tasks, which is quite substantial at more than 8 hours per day. This may be due to women in the intervention group having the time to take on additional aspects of these tasks, or to couples doing these tasks together; further research should seek to understand how tasks change or shift within the household as men take on greater caregiving roles, and how men’s involvement can alleviate women’s care burden.

Our study is not without limitations. We were unable to collect baseline data from women and the intervention constrained the sample design to randomization at the individual level. We were unable to mask group assignment from participants or the data collectors, who were not affiliated with the intervention. Like many behavioral interventions assessing violence and reproductive health, our outcomes are self-reported, and intervention participants may be more likely to report what they presume are desirable answers. However, collecting data from men and women and at 21 months (which was 16 months after completion of the intervention) might have mitigated some of these concerns. It is also important to note that our prevalence findings are not generalizable to the population of Rwanda. Finally, our follow-up time frame is limited to 21 months, which, while longer than many studies, does not give a full picture of changes across the life-course.

## Conclusions

Our study demonstrates that a gender-transformative intervention can positively impact a range of health and gender-related behavioral outcomes. Our study builds on existing evidence of male engagement interventions and makes unique contributions to measuring the impact of male engagement on household power dynamics. While our findings show substantial positive effects, high rates of inequality and violence persist: about one in three women in the intervention group reported experiencing IPV in the past 12 months, the vast majority of parents used physical punishment, and men still dominated household decisions. Further research should examine whether these rates can be lowered if the intervention is implemented over longer time periods or with additional components. Future research could also directly measure health outcomes and use health facility or biomarker data to corroborate self-reported behavior change, and examine the effect of the intervention if implemented over longer time periods, when implemented with greater numbers and in other settings, or when delivered through the public sector. Nevertheless, the findings highlight the promise of the Bandebereho intervention, designed and adapted to fit the particular cultural context. Targeting the transition into fatherhood and parenting, and supporting couples with skills to make their relationships stronger and more equitable, had important effects on the intervention outcomes.

## Supporting information

S1 File Bandebereho women’s 21-month questionnaire (English). (PDF)

S2 File Bandebereho women’s 21-month questionnaire (Kinyarwanda). (PDF)

S3 File Bandebereho men’s 21-month questionnaire (English). (PDF)

S4 File Bandebereho men’s 21-month questionnaire (Kinyarwanda). (PDF)

S5 File Bandebereho study protocol submitted to Rwanda National ethics Committee. (PDF)

S6 File Bandebereho study CONSORT checklist. (PDF)

S7 File Bandebereho study TIDieR checklist. (PDF)
