# Supplementary material for: Gender-transformative Bandebereho couples’ intervention to promote male engagement in reproductive and maternal health and violence prevention in Rwanda: Findings from a randomized controlled trial
Source: PLoS One. 2018 Apr 4;13(4):e0192756. doi: 10.1371/journal.pone.0192756 (PMC5884496; doi:10.1371/journal.pone.0192756)
Supplement: S1 File — (PDF) [file pone.0192756.s001.pdf]

Bandebereho_21_Followup_Female_Survey_Final

Field	Question	Answer
survey		
survey > coversheet		
partcipant_group	ENUMERATOR: Please check on the list of participants and confirm the group	1 Treatment group<br>2 Control group
survey > Part 1. Demographic information		
A01	A01 How old are you?<br>*If respondent doesn't know her age, put 998; if she refuses to answer put 999. [Enter age]*<br>*Response constrained to: .<=60 or .=998 or .=999*	
A02	A02 What is the highest level of school you have attained?<br>*Question relevant when: ${A01} >=18*	0 None<br>1 Some Primary<br>2 Primary Completed<br>3 Some Secondary<br>4 Secondary Completed<br>5 TVET/TTC<br>6 Some University<br>999 Refused to answer
A04	A04 What is your main source of employment?<br>*Question relevant when: ${A01} >=18*	1 Employed / earning a wage<br>2 Self-employed<br>3 Unemployed, but looking for work<br>4 Unemployed and not looking for work<br>5 Unable to work/disabled<br>999 Refused to answer
survey > Part 1. Demographic information > asset<br>*Group relevant when: ${A01} >=1*		
A05	A05 How often can your household afford the following items:<br>*Consider the frequency that the participant is unable to meet any of the needs listed (not all needs). For example, if she always has shelter but she is only able to afford food often, select "often." If she is able to afford food only sometimes, select sometimes. PLEASE READ ALL EXAMPLES:*<br>*Question relevant when: ${A01} >=18*	1 Never<br>2 Sometimes<br>3 Often<br>4 Always
A05a	A05a Basic Items (food or shelter)<br>*Question relevant when: ${A01} >=18*	1 Never<br>2 Sometimes<br>3 Often<br>4 Always
A05b	A05b Important Items (clothing, or school fees, or health insurance)<br>*Question relevant when: ${A01} >=18*	1 Never<br>2 Sometimes<br>3 Often<br>4 Always
A05c	A05c Extra Items (gifts,or travel)<br>*Question relevant when: ${A01} >=18*	1 Never<br>2 Sometimes<br>3 Often<br>4 Always
note_a	I am going to ask you some questions about who lives with you in your household.<br>*Question relevant when: ${A01} >=18*	
A06	A06 How many adult men live in your household?<br>*[Enter number]*<br>*Question relevant when: ${A01} >=18*	
A07	A07 How many adult women, not including yourself, live in your household?<br>*[Enter number]*<br>*Question relevant when: ${A01} >=18*	
A08	A08 How many male children (under age 18) live in your household?<br>*This includes any male child, whether biological or not! [Enter number]*<br>*Question relevant when: ${A01} >=18*	
A09	A09 How many female children (under age 18) live in your household?<br>*This includes any female child, whether biological or not! [Enter number]*<br>*Question relevant when: ${A01} >=18*	
A10	A10 How many biological children do you have?<br>*Only count those already born; living children that are biological children. [Enter number of children]*<br>*Question relevant when: ${A01} >=18*	

A11	A11 What is the age and sex of each of your biological children?<br>*Ask the respondent to list all of her children, starting with her oldest child. If the respondent doesn't know the age of her child, put 998. Enter 0 if child is less than 1 year and specify months in next question.*<br>*Question relevant when: ${A10} !='0' and ${A01} >=18*	
survey > Part 1. Demographic information > Age and sex children (1)<br>*Group relevant when: ${A10} !='0' and ${A01} >=18*		(Repeated group)
A11a	A11a Age of child number 1<br>*[Enter Age]*	
A11b	A11b How many months is the child number 1?<br>*Question relevant when: ${A11a} ='0'*<br>*Response constrained to: .<=11*	
A11c	A11c Sex of child number 1	1 Female<br>2 Male
A12	A12 Who in your household has health insurance?<br>*MARK ALL THAT APPLY*<br>*Question relevant when: ${A01} >=18*<br>*Response constrained to: count-selected(.)=1 or (count-selected(.)>=2 and not(selected(.,0)) and not(selected(.,998)) and not(selected(.,999)))*	0 None<br>1 Self<br>2 My husband/partner<br>3 One or more of my children<br>998 I don't know<br>5 Other<br>999 Refused to answer
A13	A13 Have you put aside any money in the past two weeks for savings, including any contributions to a savings group or rotating savings scheme?<br>*Question relevant when: ${A01} >=18*	0 No<br>1 Yes<br>999 Refused to answer
survey > PART 2. PREGNANCY & BIRTH<br>*Group relevant when: ${A01} >=18*		
note2	In this section I am going to ask you some questions about your experience of having children or preparing to have a child. Remember that all of your responses will be kept confidential.	
B01	B01 Are you currently expecting a child?	0 No<br>1 Yes<br>999 Refused to answer
B02	B02 When is the estimated birth date? Enter how many months until the child is born<br>*Insert 998 if respondent doesn't know. If child is due in less than one month, enter 0. [enter amount in months]*<br>*Question relevant when: ${B01} ='1'*<br>*Response constrained to: .<=9 or .=998*	
B03	B03 How many antenatal care (ANC) visits have you attended so far during your current pregnancy?<br>*[enter amount]*<br>*Question relevant when: ${B01} ='1'*	
B04	B04 How many times has your husband/partner accompanied you to attend an antenatal care (ANC) visit during your current pregnancy?<br>*Insert -98 if respondent doesn't know / remember. [enter amount]*<br>*Question relevant when: ${B01} ='1' and ${B03} !='0'*<br>*Response constrained to: (.<= ${B03} and .>=0) or .=-98*	
B04a	B04a During the last antenatal care visit your partner attended, did he:<br>*PLEASE READ RESPONSE OPTIONS*<br>*Question relevant when: ${B01} ='1' and ${B03} !='0' and ${B04} !='0'*	1 Drop you off at the entrance or wait for you outside<br>2 Wait in the health facility, but not participate in the ANC visit<br>3 Join you for at least part of the visit with the health provider<br>4 Other
B04b	B04b What was your experience when your partner participated in the antenatal care (ANC) visit with you?<br>*READ RESPONSE OPTIONS and MARK ALL THAT APPLY*<br>*Question relevant when: ${B01} ='1' and ${B03} !='0' and ${B04} !='0' and ${B04a} ='3'*	1 I was comfortable<br>2 I was uncomfortable<br>3 I was embarassed<br>4 I was happy<br>5 I felt supported by my partner<br>6 I didn't mind<br>7 I was annoyed or frustrated<br>8 I received more information<br>9 I liked having my partner there<br>10 I did not like having my partner there<br>11 I received better care<br>12 I was treated with more respect

		13 I was seen more quickly (did not have to wait long)<br>14 I felt closer to my partner
B06	B06 Would you like your partner to be with you in the delivery room when you give birth?<br>*Question relevant when: ${B01} ='1'*	0 No<br>1 Yes<br>998 Don't know/not sure
B07	B07 I would like to ask about the pregnancy and birth of your youngest living child. When you were pregnant with your youngest child, how many antenatal care (ANC) visits did you attend?<br>*Insert 998 if respondent doesn't know/remember. [enter number of visits]*<br>*Question relevant when: ${A10} !='0'*	
B08	B08 When you were pregnant with your youngest child, how many times did your partner accompany you to an antenatal care (ANC) visit?<br>*Insert -98 if respondent doesn't know / remember. [enter number of visits]*<br>*Question relevant when: ${A10} !='0' and ${B07} !='0'*<br>*Response constrained to: (.<= ${B07} and .>=0) or .=-98*	
B08a	B08a During the last antenatal care visit your partner attended, did he:<br>*PLEASE READ RESPONSE OPTIONS.*<br>*Question relevant when: (${A10} !='0' and ${B01} ='0' and ${B07} !='0' and ${B08} !='0') or (${A10} !='0' and ${B01} ='1' and ${B04a} !='3' and ${B07} !='0' and ${B08} !='0')*	1 Drop you off at the entrance or wait for you outside<br>2 Wait in the health facility, but not participate in the ANC visit<br>3 Join you for at least part of the visit with the health provider<br>4 Other
B08b	B08b What was your experience when your partner participated in the antenatal care (ANC) visit with you?<br>*READ RESPONSE OPTIONS and MARK ALL THAT APPLY If a woman did not give birth in a health facility, select Not Applicable*<br>*Question relevant when: ${B08a} ='3'*	1 I was comfortable<br>2 I was uncomfortable<br>3 I was embarassed<br>4 I was happy<br>5 I felt supported by my partner<br>6 I didn't mind<br>7 I was annoyed or frustrated<br>8 I received more information<br>9 I liked having my partner there<br>10 I did not like having my partner there<br>11 I received better care<br>12 I was treated with more respect<br>13 I was seen more quickly (did not have to wait long)<br>14 I felt closer to my partner
B11	B11 Where was your partner during the birth of your last child?<br>*PLEASE READ RESPONSE OPTIONS. If a woman did not give birth in a health facility, put 'not applicable'.*<br>*Question relevant when: ${A10} !='0'*	1 He was in the delivery room with me<br>2 He was at the health facility, but not in the room with me<br>3 He was not at the health facility<br>997 Not Applicable<br>998 Don't know/don't remember
B11a	B11a How was your experience of having your partner in the room with you during the birth?<br>*READ RESPONSE OPTIONS and MARK ALL THAT APPLY*<br>*Question relevant when: ${A10} !='0' and ${B11} ='1'*	1 I was comfortable<br>2 I was uncomfortable<br>3 I was embarassed<br>4 I was happy<br>5 I felt supported by my partner<br>6 I didn't mind<br>7 I was annoyed or frustrated<br>8 I received more information<br>9 I liked having my partner there<br>10 I did not like having my partner there<br>11 I received better care<br>12 I was treated with more respect<br>13 I felt closer to my partner

B13	B13 After the birth of your youngest child, did you or your partner take the child for vaccination, or did you take the child together?<br>*Question relevant when: ${A10} !='0'*	0	No
		1	Yes, I took the child
		2	Yes, my partner took the child
		3	Yes, we both took the child, either separately or together
		997	Not applicable
B15	B15 Now, I would like to you to think about your most recent pregnancy. How often did you discuss with your partner:<br>*READ RESPONSE OPTIONS, Most recent pregnancy can refer to either a current pregnancy, or to the pregnancy of the woman's youngest child.*<br>*Question relevant when: ${A10} !='0' or ${B01} ='1'*		
B15c	B15c Health care during pregnancy<br>*Question relevant when: ${A10} !='0' or ${B01} ='1'*	1	Often
		2	Sometimes
		3	Rarely
		4	Never
		997	Not Applicable
B15e	B15e Your concerns about having or raising children<br>*Question relevant when: ${A10} !='0' or ${B01} ='1'*	1	Often
		2	Sometimes
		3	Rarely
		4	Never
		997	Not Applicable
B15f	B15f Whether or when to start using contraception to delay/space the next pregnancy<br>*Question relevant when: ${A10} !='0' or ${B01} ='1'*	1	Often
		2	Sometimes
		3	Rarely
		4	Never
		997	Not Applicable
B16	B16 Now I am going to read you a statement. I want you to think about your most recent pregnancy and tell me if you strongly agree, agree, neither agree nor disagree, disagree or strongly disagree with the statement: During my most recent pregnancy, my partner encouraged or assisted me to attend maternal health services (antenatal care)<br>*Most recent pregnancy can refer to either a current pregnancy, or to the pregnancy of the woman's youngest child.*<br>*Question relevant when: ${A10} !='0' or ${B01} ='1'*	1	Strongly agree
		2	Agree
		3	Neither agree nor disagree
		4	Disagree
		5	Strongly disagree
		999	Refused to answer
B27	B27 During your most recent pregnancy, did your partner do any of the following:<br>*Most recent pregnancy can refer to either a current pregnancy, or to the pregnancy of the woman's youngest child.*<br>*Question relevant when: ${A10} !='0' or ${B01} ='1'*		
B27a	B27a During your most recent pregnancy, did your partner do any of the following: Provided financial support (for medical care, or transport, or for the needs of the child)<br>*Question relevant when: ${A10} !='0' or ${B01} ='1'*	0	No
		1	Yes
		999	Refused to answer
		997	Not Applicable
B27b	B27b During your most recent pregnancy, did your partner do any of the following: Did some of the household tasks that you would normally do<br>*Question relevant when: ${A10} !='0' or ${B01} ='1'*	0	No
		1	Yes
		999	Refused to answer
		997	Not Applicable
B27c	B27c During your most recent pregnancy, did your partner do any of the following: Prepared food for you or made sure that you had nutritious food to eat<br>*Question relevant when: ${A10} !='0' or ${B01} ='1'*	0	No
		1	Yes
		999	Refused to answer
		997	Not Applicable
B27d	B27d During your most recent pregnancy, did your partner do any of the following: Encouraged you to take care of yourself (to rest, or to eat well)<br>*Question relevant when: ${A10} !='0' or ${B01} ='1'*	0	No
		1	Yes
		999	Refused to answer
		997	Not Applicable
B27e	B27e During your most recent pregnancy, did your partner do any of the following: Provided care or emotional support to you (asked how you were feeling, offered words of encouragement when you felt unwell)<br>*Question relevant when: ${A10} !='0' or ${B01} ='1'*	0	No
		1	Yes
		999	Refused to answer
		997	Not Applicable
B27f	B27f During your most recent pregnancy, did your partner do any of the following: Provided spiritual support or guidance (prayed for a healthy pregnancy or the health of the child)<br>*Question relevant when: ${A10} !='0' or ${B01} ='1'*	0	No
		1	Yes
		999	Refused to answer
		997	Not Applicable
B27g	B27g During your most recent pregnancy, did your partner do any of the following: Took care of your other children (if		

	applicable)<br>*Question relevant when: ${A10} !='0' or ${B01} ='1'*	0<br>1<br>999<br>997	No<br>Yes<br>Refused to answer<br>Not Applicable

survey > PART 3: SEXUAL AND REPRODUCTIVE HEALTH

Group relevant when: ${A01} >=18

note3	Now I would like to ask you some questions about your sexual and reproductive health. Let me assure you again that your answers are completely confidential and will not be told to anyone. If we should come to any question that you don't want to answer, just let me know and we will go to the next question.		
C01	C01 Do you or your partner currently use any method to avoid or delay pregnancy?<br>*MARK ALL THAT APPLY*<br>*Response constrained to: count-selected(.)=1 or (count-selected(.)>=2 and not(selected(.,0)) and not(selected(.,10)) and not(selected(.,999)))*	0<br>1<br>2<br>3<br>4<br>5<br>6<br>7<br>8<br>9<br>10<br>11<br>999	None<br>Pill<br>Male Condom<br>Female Condom<br>Injection<br>IUD<br>Man had a vasectomy<br>Woman had a tubal ligation or hysterectomy<br>Implant<br>Traditional methods (e.g. periodic abstinence, withdrawal, cycle beads)<br>I don't know<br>Other<br>Refused to answer
C02	C02 Have you ever used a form of contraception when your partner did not know you were using it?	0<br>1<br>2<br>999	No<br>Yes<br>Not applicable<br>Refused to answer
C02a	C02a Has this happened in the past year (since the time of the last interview?)<br>*Question relevant when: ${C02} ='1'*	0<br>1<br>999	No<br>Yes<br>Refused to answer
C07	C07 Now I am going to ask you a few questions about things that may have happened with your partner in the past year (since the time of the last interview). Please tell me whether you strongly agree, agree, disagree or strongly disagree with the following statements:		
C07a	C07a In the past year (in the period since the last interview): My partner has prevented me from using a contraceptive method when I wanted to use one	1<br>2<br>3<br>4<br>997<br>999	Strongly agree<br>Agree<br>Disagree<br>Strongly disagree<br>Not applicable<br>Refused to answer
C07b	C07b In the past year (in the period since the last interview): My partner has interfered with or made it difficult for me to use a contraceptive method when I wanted to use one	1<br>2<br>3<br>4<br>997<br>999	Strongly agree<br>Agree<br>Disagree<br>Strongly disagree<br>Not applicable<br>Refused to answer
C07c	C07c In the past year (in the period since the last interview): My partner has made me use a contraceptive method when I did not want to use one	1<br>2<br>3<br>4<br>997<br>999	Strongly agree<br>Agree<br>Disagree<br>Strongly disagree<br>Not applicable<br>Refused to answer
C07f	C07f In the past year (in the period since the last interview):My partner has forced me or pressured me to use one type of contraceptive method, when I wanted to use a different contraceptive method	1<br>2<br>3<br>4<br>997<br>999	Strongly agree<br>Agree<br>Disagree<br>Strongly disagree<br>Not applicable<br>Refused to answer
C07e	C07e In the past year (in the period since the last interview): My partner has pressured me to become pregnant	1	Strongly agree

		2	Agree
		3	Disagree
		4	Strongly disagree
		997	Not applicable
		999	Refused to answer
C.10	C.10 I am now going to read a statement. Please tell me if you strongly agree, agree, disagree, or strongly disagree: If I wanted to use a contraceptive method my partner would not allow me to	1	Strongly agree
		2	Agree
		3	Disagree
		4	Strongly disagree
		997	Not applicable
		999	Refused to answer

survey > PART 4: RELATIONSHIPS

Group relevant when: ${A01} >=18

note4	When two people are married, they usually share both good and bad moments. I would like to ask you some questions about your relationship with your husband/partner. All of your responses will be kept completely confidential and will not be told to anyone.		
D01	D01 I am going to read you some statements. Please tell me if this happens 'often', 'sometimes', 'rarely', or 'never'...		
D01a	D01a When I have problems my partner listens to me	1	Often
		2	Sometimes
		3	Rarely
		4	Never
		998	Don't know
D01b	D01b My partner blames me for things that go wrong	1	Often
		2	Sometimes
		3	Rarely
		4	Never
		998	Don't know
D01c	D01c I feel appreciated by my partner	1	Often
		2	Sometimes
		3	Rarely
		4	Never
		998	Don't know
D01d	D01d I feel respected even if we disagree	1	Often
		2	Sometimes
		3	Rarely
		4	Never
		998	Don't know
D01e	D01e We are good at solving our differences	1	Often
		2	Sometimes
		3	Rarely
		4	Never
		998	Don't know
D01f	D01f My partner criticizes my opinions, feelings, or desires	1	Often
		2	Sometimes
		3	Rarely
		4	Never
		998	Don't know
D01g	D01g My partner shows love and affection for me	1	Often
		2	Sometimes
		3	Rarely
		4	Never
		998	Don't know
D02	D02 Now, please tell me if you 'strongly agree', 'agree',' disagree', 'strongly disagree' or 'neither agree nor disagree' with the following statement:		
D02a	D02a My partner and I often talk about things that happen to each of us during the day	1	Strongly agree
		2	Agree
		3	Neither agree nor disagree
		4	Disagree
		5	Strongly disagree

D04	D04 In general, how often would you say that you and your partner quarrel?<br>*READ RESPONSE OPTIONS*	1	Often
		2	Sometimes
		3	Rarely
		4	Never
		999	Refused to answer
D05	D05 Now I'd like to ask you about your communication with your partner. How often do you discuss with your partner your household's weekly or monthly income and expenses?	1	Often
		2	Sometimes
		3	Rarely
		4	Never
		997	Not applicable
		999	Refused to answer
D05a	D05a When you discuss your household's weekly or monthly income and expenses, do you feel that your partner takes your opinions seriously?<br>*Question relevant when: ${D05} !='4' and ${D05} !='997'*	1	Often
		2	Sometimes
		3	Rarely
		4	Never
		998	I don't know
		999	Refused to answer
D05c	D05c Who makes the final decision about your household's weekly or monthly income and expenses?<br>*Question relevant when: ${D05} !='997'*	1	You
		2	Your partner
		3	Both have the same say
		4	Someone else
		998	Don't know
		999	Refused to answer
D06	D06 How often do you discuss with your partner spending money on large investments, such as buying a cow, forest or plot of land?	1	Often
		2	Sometimes
		3	Rarely
		4	Never
		997	Not applicable
		999	Refused to answer
D06a	D06a When you discuss spending money on large investments, such as buying a cow, forest or plot of land, do you feel that your partner takes your opinions seriously?<br>*Question relevant when: ${D06} !='4' and ${D06} !='997'*	1	Often
		2	Sometimes
		3	Rarely
		4	Never
		998	I don't know
		999	Refused to answer
D06c	D06c Who makes the final decision about the spending money on large investments, such as buying a cow, forest or plot of land?<br>*Question relevant when: ${D06} !='997'*	1	You
		2	Your partner
		3	Both have the same say
		4	Someone else
		998	Don't know
		999	Refused to answer
D07	D07 How often do you discuss with your partner how many children to have or the spacing of children?	1	Often
		2	Sometimes
		3	Rarely
		4	Never
		997	Not applicable
		999	Refused to answer
D07a	D07a When you discuss how many children to have or the spacing of children, do you feel that your partner takes your opinions seriously?<br>*Question relevant when: ${D07} !='4' and ${D07} !='997'*	1	Often
		2	Sometimes
		3	Rarely
		4	Never
		998	I don't know
		999	Refused to answer
D07c	D07c Who makes the final decision about how many children to have or the spacing of children?<br>*Question relevant when: ${D07} !='997'*	1	You
		2	Your partner
		3	Both have the same say
		4	Someone else
		998	Don't know
		999	Refused to answer

D08	D08 How often do you discuss with your partner whether you can work outside the home?	1	Often
		2	Sometimes
		3	Rarely
		4	Never
		997	Not applicable
		999	Refused to answer
D08a	D08a When you discuss whether you can work outside the home, do you feel that your partner takes your opinions seriously?<br>*Question relevant when: ${D08} !='4' and ${D08} !='997'*	1	Often
		2	Sometimes
		3	Rarely
		4	Never
		998	I don't know
		999	Refused to answer
D08c	D08c Who makes the final decision about whether you can work outside the home?<br>*Question relevant when: ${D08} !='997'*	1	You
		2	Your partner
		3	Both have the same say
		4	Someone else
		998	Don't know
		999	Refused to answer
D09	D09 How often do you discuss with your partner how his cash earnings are used?	1	Often
		2	Sometimes
		3	Rarely
		4	Never
		997	Not applicable
		999	Refused to answer
D09a	D09a When you discuss how your partner's (the man's) cash earnings are used, do you feel that your partner takes your opinions seriously?<br>*Question relevant when: ${D09} !='4' and ${D09} !='997'*	1	Often
		2	Sometimes
		3	Rarely
		4	Never
		998	I don't know
		999	Refused to answer
D09c	D09c Who makes the final decision about how your partner's (the man's) cash earnings are used?<br>*Question relevant when: ${D09} !='997'*	1	You
		2	Your partner
		3	Both have the same say
		4	Someone else
		998	Don't know
		999	Refused to answer
D10	D10 How often do you discuss with your partner how your (the woman's) cash earnings are used?	1	Often
		2	Sometimes
		3	Rarely
		4	Never
		997	Not applicable
		999	Refused to answer
D10a	D10a When you discuss how your (the woman's) cash earnings are used, do you feel that your partner takes your opinions seriously?<br>*Question relevant when: ${D10} !='4' and ${D10} !='997'*	1	Often
		2	Sometimes
		3	Rarely
		4	Never
		998	I don't know
		999	Refused to answer
D10c	D10c Who makes the final decision about how your (the woman's) cash earnings are used?<br>*Question relevant when: ${D10} !='997'*	1	You
		2	Your partner
		3	Both have the same say
		4	Someone else
		998	Don't know
		999	Refused to answer
D11	D11 How often do you discuss with your partner whether to use contraception or which method to use?	1	Often
		2	Sometimes
		3	Rarely
		4	Never
		997	Not applicable
		999	Refused to answer

D11a	D11a When you discuss whether to use contraception or which method to use, do you feel that your partner takes your opinions seriously?<br>*Question relevant when: ${D11} !='4' and ${D11} !='997'*	1<br>2<br>3<br>4<br>998<br>999	Often<br>Sometimes<br>Rarely<br>Never<br>I don't know<br>Refused to answer
D11c	D11c Who makes the final decision about whether to use contraception or which method to use?<br>*Question relevant when: ${D11} !='997'*	1<br>2<br>3<br>4<br>998<br>999	You<br>Your partner<br>Both have the same say<br>Someone else<br>Don't know<br>Refused to answer
D13	D13 How often do you discuss with your partner about your children's schooling?	1<br>2<br>3<br>4<br>997<br>999	Often<br>Sometimes<br>Rarely<br>Never<br>Not applicable<br>Refused to answer
D13a	D13a When you discuss your children's schooling, do you feel that your partner takes your opinions seriously?<br>*Question relevant when: ${D13} !='4' and ${D13} !='997'*	1<br>2<br>3<br>4<br>998<br>999	Often<br>Sometimes<br>Rarely<br>Never<br>I don't know<br>Refused to answer
D13c	D13c Who makes the final decision about your children's schooling?<br>*Question relevant when: ${D13} !='997'*	1<br>2<br>3<br>4<br>998<br>999	You<br>Your partner<br>Both have the same say<br>Someone else<br>Don't know<br>Refused to answer

survey > PART 5: CAREGIVING

Group relevant when: ${A01} >=18

note5	Now I would like to ask you some questions about your family and about household tasks.		
note_512	I am now going to ask you about how you and your partner divide different childcare tasks.<br>*NOTE: The following questions ask about how a woman and her partner divide childcare tasks. In some cases the child may be too young for the activity to be applicable (for example: to go to school) or the activity may be done by someone else in the household (e.g. a maid or other family member). In both cases, put 'not applicable'.*<br>*Question relevant when: ${A10} !='0'*		
E.12	E.12 If you disregard the outside help you receive from others, how do you and your partner divide the following task: Providing the daily care of your child/ren<br>*Question relevant when: ${A10} !='0'*	1<br>2<br>3<br>4<br>5<br>997	Always you<br>Usually you<br>Shared equally or done together<br>Usually partner<br>Always partner<br>Not applicable
E.12a	E.12a During the last 7 days, on how many days did you do this task?<br>*[enter # days]*<br>*Question relevant when: ${A10} !='0' and ${E.12} !='997'*<br>*Response constrained to: .<=7*		
E.12b	E.12b On a typical day when you do this task, how many hours do you spend doing it?<br>*Please indicate time at 15 minute intervals: for example, if a person might say he spent 15 minutes on a task, mark 0.25 (1 quarter hour). Round any amount up to 15 minutes ; round all other amounts to the closest 15 minute interval (e.g. if 35 minutes, round to 30 minutes, entering 0.5 hours). [enter # hours]*<br>*Question relevant when: ${A10} !='0' and ${E.12} !='997' and ${E.12a} !='0'*<br>*Response constrained to: .<=24 and .>0*		
E.13	E.13 If you disregard the outside help you receive from others, how do you and your partner divide the following task: Holding your child/ren when he or she was a newborn<br>*Question relevant when: ${A10} !='0'*	1<br>2<br>3<br>4<br>5<br>997	Always you<br>Usually you<br>Shared equally or done together<br>Usually partner<br>Always partner<br>Not applicable

E.13a	E.13a During the last 7 days, on how many days did you do this task?<br>*[enter # days]*<br>*Question relevant when: ${A10} !='0' and ${E.13} !='997'*<br>*Response constrained to: .<=7*	
E.13b	E.13b On a typical day when you do this task, how many hours do you spend doing it?<br>*Please indicate time at 15 minute intervals: for example, if a person might say he spent 15 minutes on a task, mark 0.25 (1 quarter hour). Round any amount up to 15 minutes ; round all other amounts to the closest 15 minute interval (e.g. if 35 minutes, round to 30 minutes, entering 0.5 hours). [enter # hours]*<br>*Question relevant when: ${A10} !='0' and ${E.13} !='997' and ${E.13a} !='0'*<br>*Response constrained to: .<=24 and .>0*	
E.14	E.14 If you disregard the outside help you receive from others, how do you and your partner divide the following task:<br>Feeding your child/ren<br>*Question relevant when: ${A10} !='0'*	1 Always you<br>2 Usually you<br>3 Shared equally or done together<br>4 Usually partner<br>5 Always partner<br>997 Not applicable
E.14a	E.14a During the last 7 days, on how many days did you do this task?<br>*[enter # days]*<br>*Question relevant when: ${A10} !='0' and ${E.14} !='997'*<br>*Response constrained to: .<=7*	
E.14b	E.14b On a typical day when you do this task, how many hours do you spend doing it?<br>*Please indicate time at 15 minute intervals: for example, if a person might say he spent 15 minutes on a task, mark 0.25 (1 quarter hour). Round any amount up to 15 minutes ; round all other amounts to the closest 15 minute interval (e.g. if 35 minutes, round to 30 minutes, entering 0.5 hours). [enter # hours]*<br>*Question relevant when: ${A10} !='0' and ${E.14} !='997' and ${E.14a} !='0'*<br>*Response constrained to: .<=24 and .>0*	
E.15	E.15 If you disregard the outside help you receive from others, how do you and your partner divide the following task:<br>Bathing your child/ren<br>*Question relevant when: ${A10} !='0'*	1 Always you<br>2 Usually you<br>3 Shared equally or done together<br>4 Usually partner<br>5 Always partner<br>997 Not applicable
E.15a	E.15a During the last 7 days, on how many days did you do this task?<br>*[enter # days]*<br>*Question relevant when: ${A10} !='0' and ${E.15} !='997'*<br>*Response constrained to: .<=7*	
E.15b	E.15b On a typical day when you do this task, how many hours do you spend doing it?<br>*Please indicate time at 15 minute intervals: for example, if a person might say he spent 15 minutes on a task, mark 0.25 (1 quarter hour). Round any amount up to 15 minutes ; round all other amounts to the closest 15 minute interval (e.g. if 35 minutes, round to 30 minutes, entering 0.5 hours). [enter # hours]*<br>*Question relevant when: ${A10} !='0' and ${E.15} !='997' and ${E.15a} !='0'*<br>*Response constrained to: .<=24 and .>0*	
E.16	E.16 If you disregard the outside help you receive from others, how do you and your partner divide the following task:<br>Soothing your child/ren if he or she is crying or upset<br>*Question relevant when: ${A10} !='0'*	1 Always you<br>2 Usually you<br>3 Shared equally or done together<br>4 Usually partner<br>5 Always partner<br>997 Not applicable
E.16a	E.16a During the last 7 days, on how many days did you do this task?<br>*[enter # days]*<br>*Question relevant when: ${A10} !='0' and ${E.16} !='997'*<br>*Response constrained to: .<=7*	
E.16b	E.16b On a typical day when you do this task, how many hours do you spend doing it?<br>*Please indicate time at 15 minute intervals: for example, if a person might say he spent 15 minutes on a task, mark 0.25 (1 quarter hour). Round any amount up to 15 minutes ; round all other amounts to the closest 15 minute interval (e.g. if 35 minutes, round to 30 minutes, entering 0.5 hours). [enter # hours]*<br>*Question relevant when: ${A10} !='0' and ${E.16} !='997' and ${E.16a} !='0'*<br>*Response constrained to: .<=24 and .>0*	
E.171	E.171 If you disregard the outside help you receive from others, how do you and your partner divide the following task:<br>Taking your child to the health center if sick or for vaccination<br>*Question relevant when: ${A10} !='0'*	1 Always you<br>2 Usually you<br>3 Shared equally or done together<br>4 Usually partner<br>5 Always partner

		997 Not applicable
E.171a	E.171a During the last 7 days, on how many days did you do this task?<br>*[enter # days]*<br>*Question relevant when: ${A10} !='0' and ${E.171} !='997'*<br>*Response constrained to: .<=7*	
E.17	E.17 If you disregard the outside help you receive from others, how do you and your partner divide the following task:<br>Taking and picking up your child/ren from school<br>*Question relevant when: ${A10} !='0'*	1 Always you<br>2 Usually you<br>3 Shared equally or done together<br>4 Usually partner<br>5 Always partner<br>997 Not applicable
E.17a	E.17a During the last 7 days, on how many days did you do this task?<br>*[enter # days]*<br>*Question relevant when: ${A10} !='0' and ${E.17} !='997'*<br>*Response constrained to: .<=7*	
E.17b	E.17b On a typical day when you do this task, how many hours do you spend doing it?<br>*Please indicate time at 15 minute intervals: for example, if a person might say he spent 15 minutes on a task, mark 0.25 (1 quarter hour). Round any amount up to 15 minutes ; round all other amounts to the closest 15 minute interval (e.g. if 35 minutes, round to 30 minutes, entering 0.5 hours). [enter # hours]*<br>*Question relevant when: ${A10} !='0' and ${E.17} !='997' and ${E.17a} !='0'*<br>*Response constrained to: .<=24 and .>0*	
E.18	E.18 If you disregard the outside help you receive from others, how do you and your partner divide the following task:<br>Telling a story, singing a song, or playing with your child/ren<br>*Question relevant when: ${A10} !='0'*	1 Always you<br>2 Usually you<br>3 Shared equally or done together<br>4 Usually partner<br>5 Always partner<br>997 Not applicable
E.18a	E.18a During the last 7 days, on how many days did you do this task?<br>*[enter # days]*<br>*Question relevant when: ${A10} !='0' and ${E.18} !='997'*<br>*Response constrained to: .<=7*	
E.18b	E.18b On a typical day when you do this task, how many hours do you spend doing it?<br>*Please indicate time at 15 minute intervals: for example, if a person might say he spent 15 minutes on a task, mark 0.25 (1 quarter hour). Round any amount up to 15 minutes ; round all other amounts to the closest 15 minute interval (e.g. if 35 minutes, round to 30 minutes, entering 0.5 hours). [enter # hours]*<br>*Question relevant when: ${A10} !='0' and ${E.18} !='997' and ${E.18a} !='0'*<br>*Response constrained to: .<=24 and .>0*	
E.19	E.19 If you disregard the outside help you receive from others, how do you and your partner divide the following task:<br>Teaching your child/ren something<br>*Question relevant when: ${A10} !='0'*	1 Always you<br>2 Usually you<br>3 Shared equally or done together<br>4 Usually partner<br>5 Always partner<br>997 Not applicable
E.19a	E.19a During the last 7 days, on how many days did you do this task?<br>*[enter # days]*<br>*Question relevant when: ${A10} !='0' and ${E.19} !='997'*<br>*Response constrained to: .<=7*	
E.19b	E.19b On a typical day when you do this task, how many hours do you spend doing it?<br>*Please indicate time at 15 minute intervals: for example, if a person might say he spent 15 minutes on a task, mark 0.25 (1 quarter hour). Round any amount up to 15 minutes ; round all other amounts to the closest 15 minute interval (e.g. if 35 minutes, round to 30 minutes, entering 0.5 hours). [enter # hours]*<br>*Question relevant when: ${A10} !='0' and ${E.19} !='997' and ${E.19a} !='0'*<br>*Response constrained to: .<=24 and .>0*	
E.20	E.20 If you disregard the outside help you receive from others, how do you and your partner divide the following task:<br>Disciplining your child/ren (verbal discipline)<br>*Question relevant when: ${A10} !='0'*	1 Always you<br>2 Usually you<br>3 Shared equally or done together<br>4 Usually partner<br>5 Always partner<br>997 Not applicable
E.20a	E.20a During the last 7 days, on how many days did you do this task?<br>*[enter # days]*<br>*Question relevant when: ${A10} !='0' and ${E.20} !='997'*	

	Response constrained to: .<=7	
E.21	E.21 If you disregard the outside help you receive from others, how do you and your partner divide the following task: Spanking or beating your child/ren<br>*Question relevant when: ${A10} !='0'*	1 Always you<br>2 Usually you<br>3 Shared equally or done together<br>4 Usually partner<br>5 Always partner<br>997 Not applicable
E.21a	E.21a During the last 7 days, on how many days did you do this task?<br>*[enter # days]*<br>*Question relevant when: ${A10} !='0' and ${E.21} !='997'*<br>*Response constrained to: .<=7*	
E04	E04 How much do you want your partner to be involved in taking care of your child?<br>*READ RESPONSE OPTIONS.*<br>*Question relevant when: ${A10} !='0'*	1 I'm happy with his current involvement<br>2 I'd like him to be more involved<br>3 I'd like him to be less involved<br>998 I don't know
E06	E06 I would like to ask you some questions about raising children. Adults use certain ways to teach children how to behave well and to correct them when they misbehave. Please tell me if you have used any of these methods in the PAST Month with any of your (biological) children.<br>*Question relevant when: ${A10} !='0'*	
E06a	E06a Took away privileges, or forbade something your child did or did not allow your child to leave the house<br>*This question refers to the past MONTH, and with any biological children.*<br>*Question relevant when: ${A10} !='0'*	0 No<br>1 Yes
E06b	E06b Explained why your child's behavior was wrong<br>*This question refers to the past MONTH, and with any biological children.*<br>*Question relevant when: ${A10} !='0'*	0 No<br>1 Yes
E06c	E06c Shook your child<br>*This question refers to the past MONTH, and with any biological children.*<br>*Question relevant when: ${A10} !='0'*	0 No<br>1 Yes
E06d	E06d Shouted, or yelled at or screamed at your child<br>*This question refers to the past MONTH, and with any biological children.*<br>*Question relevant when: ${A10} !='0'*	0 No<br>1 Yes
E06e	E06e Gave your child something else to do<br>*This question refers to the past MONTH, and with any biological children.*<br>*Question relevant when: ${A10} !='0'*	0 No<br>1 Yes
E06f	E06f Spanked, or hit or slapped your child on the bottom with bare hand<br>*This question refers to the past MONTH, and with any biological children.*<br>*Question relevant when: ${A10} !='0'*	0 No<br>1 Yes
E06g	E06g Hit your child on the bottom or elsewhere on the body with something like a belt, or stick or other hard object<br>*This question refers to the past MONTH, and with any biological children.*<br>*Question relevant when: ${A10} !='0'*	0 No<br>1 Yes
E06h	E06h Called your child stupid, or lazy, or another name like that<br>*This question refers to the past MONTH, and with any biological children.*<br>*Question relevant when: ${A10} !='0'*	0 No<br>1 Yes
E06i	E06i Hit or slapped your child on the face, or head or ears<br>*This question refers to the past MONTH, and with any biological children.*<br>*Question relevant when: ${A10} !='0'*	0 No<br>1 Yes
E06j	E06j Hit or slapped your child on the hand, or arm or leg<br>*This question refers to the past MONTH, and with any biological children.*<br>*Question relevant when: ${A10} !='0'*	0 No<br>1 Yes
E06k	E06k Beat your child up, meaning you hit your child over and over as hard as you could<br>*This question refers to the past MONTH, and with any biological children.*<br>*Question relevant when: ${A10} !='0'*	0 No<br>1 Yes
E06l	E06l Made your child kneel on the ground (for a period of time)<br>*This question refers to the past MONTH, and with any biological children.*<br>*Question relevant when: ${A10} !='0'*	0 No<br>1 Yes
E06m	E06m Made your child hold bricks or stones in his/her hands while sitting down, standing or kneeling<br>*This question refers to the past MONTH, and with any biological children.*<br>*Question relevant when: ${A10} !='0'*	0 No<br>1 Yes
E07	E07 I am going to read you a series of statements. Please tell me if you strongly agree, agree, netiher agree nor disagree, disagree or strongly disagree with each of the statements.<br>*Question relevant when: ${A10} !='0'*	
E07a	E07a Children need to be physically punished in order to be raised properly	1 Strongly agree

	Question relevant when: ${A10} !='0'	2 Agree<br>3 Neither agree nor disagree<br>4 Disagree<br>5 Strongly disagree
E07b	E07b Physical punishment has negative impacts on children<br>*Question relevant when: ${A10} !='0'*	1 Strongly agree<br>2 Agree<br>3 Neither agree nor disagree<br>4 Disagree<br>5 Strongly disagree
note_E22	I am now going to ask you about how you and your partner divide different household tasks.<br>*Note: The following questions ask about how a woman and her partner divide household tasks. In some cases, the activity may not be relevant for the household or may be done by someone else in the household (e.g. maid or other family member). In this case, please put 'Not Applicable'.*	
E.22	E.22 If you disregard the outside help you receive from others, how do you and your partner divide the following task:<br>Fetching water for the household (to and from the water source)	1 Always you<br>2 Usually you<br>3 Shared equally or done together<br>4 Usually partner<br>5 Always partner<br>997 Not applicable
E.22a	E.22a During the last 7 days, on how many days did you do this task?<br>*[enter # days]*<br>*Question relevant when: ${E.22} !='997'*<br>*Response constrained to: .<=7*	
E.22b	E.22b On a typical day when you do this task, how many hours do you spend doing it?<br>*Please indicate time at 15 minute intervals: for example, if a person might say he spent 15 minutes on a task, mark 0.25 (1 quarter hour). Round any amount up to 15 minutes up to 15 minutes; round all other amounts to the closest 15 minute interval (e.g. if 35 minutes, round to 30 minutes, entering 0.5 hours). [enter # hours]*<br>*Question relevant when: ${E.22} !='997' and ${E.22a} !='0'*<br>*Response constrained to: .<=24 and .>0*	
E.23	E.23 If you disregard the outside help you receive from others, how do you and your partner divide the following task:<br>Washing clothes/do laundry	1 Always you<br>2 Usually you<br>3 Shared equally or done together<br>4 Usually partner<br>5 Always partner<br>997 Not applicable
E.23a	E.23a During the last 7 days, on how many days did you do this task?<br>*[enter # days]*<br>*Question relevant when: ${E.23} !='997'*<br>*Response constrained to: .<=7*	
E.23b	E.23b On a typical day when you do this task, how many hours do you spend doing it?<br>*Please indicate time at 15 minute intervals: for example, if a person might say he spent 15 minutes on a task, mark 0.25 (1 quarter hour). Round any amount up to 15 minutes up to 15 minutes; round all other amounts to the closest 15 minute interval (e.g. if 35 minutes, round to 30 minutes, entering 0.5 hours). [enter # hours]*<br>*Question relevant when: ${E.23} !='997' and ${E.23a} !='0'*<br>*Response constrained to: .<=24 and .>0*	
E.24	E.24 If you disregard the outside help you receive from others, how do you and your partner divide the following task:<br>Going to the market for the household for shopping	1 Always you<br>2 Usually you<br>3 Shared equally or done together<br>4 Usually partner<br>5 Always partner<br>997 Not applicable
E.24a	E.24a During the last 7 days, on how many days did you do this task?<br>*[enter # days]*<br>*Question relevant when: ${E.24} !='997'*<br>*Response constrained to: .<=7*	
E.24b	E.24b On a typical day when you do this task, how many hours do you spend doing it?<br>*Please indicate time at 15 minute intervals: for example, if a person might say he spent 15 minutes on a task, mark 0.25 (1 quarter hour). Round any amount up to 15 minutes up to 15 minutes; round all other amounts to the closest 15 minute interval (e.g. if 35 minutes, round to 30 minutes, entering 0.5 hours). [enter # hours]*<br>*Question relevant when: ${E.24} !='997' and ${E.24a} !='0'*<br>*Response constrained to: .<=24 and .>0*	
E.25	E.25 If you disregard the outside help you receive from others, how do you and your partner divide the following task:<br>Cleaning the house and surroundings	1 Always you<br>2 Usually you

		3	Shared equally or done together
		4	Usually partner
		5	Always partner
		997	Not applicable
E.25a	E.25a During the last 7 days, on how many days did you do this task?<br>*[enter # days]*<br>*Question relevant when: ${E.25} !='997'*<br>*Response constrained to: .<=7*		
E.25b	E.25b On a typical day when you do this task, how many hours do you spend doing it?<br>*Please indicate time at 15 minute intervals: for example, if a person might say he spent 15 minutes on a task, mark 0.25 (1 quarter hour). Round any amount up to 15 minutes up to 15 minutes; round all other amounts to the closest 15 minute interval (e.g. if 35 minutes, round to 30 minutes, entering 0.5 hours). [enter # hours]*<br>*Question relevant when: ${E.25} !='997' and ${E.25a} !='0'*<br>*Response constrained to: .<=24 and .>0*		
E.26	E.26 If you disregard the outside help you receive from others, how do you and your partner divide the following task: Cleaning the bathroom or toilet	1	Always you
		2	Usually you
		3	Shared equally or done together
		4	Usually partner
		5	Always partner
		997	Not applicable
E.26a	E.26a During the last 7 days, on how many days did you do this task?<br>*[enter # days]*<br>*Question relevant when: ${E.26} !='997'*<br>*Response constrained to: .<=7*		
E.26b	E.26b On a typical day when you do this task, how many hours do you spend doing it?<br>*Please indicate time at 15 minute intervals: for example, if a person might say he spent 15 minutes on a task, mark 0.25 (1 quarter hour). Round any amount up to 15 minutes up to 15 minutes; round all other amounts to the closest 15 minute interval (e.g. if 35 minutes, round to 30 minutes, entering 0.5 hours). [enter # hours]*<br>*Question relevant when: ${E.26} !='997' and ${E.26a} !='0'*<br>*Response constrained to: .<=24 and .>0*		
E.27	E.27 If you disregard the outside help you receive from others, how do you and your partner divide the following task: Cooking for the household	1	Always you
		2	Usually you
		3	Shared equally or done together
		4	Usually partner
		5	Always partner
		997	Not applicable
E.27a	E.27a During the last 7 days, on how many days did you do this task?<br>*[enter # days]*<br>*Question relevant when: ${E.27} !='997'*<br>*Response constrained to: .<=7*		
E.27b	E.27b On a typical day when you do this task, how many hours do you spend doing it?<br>*Please indicate time at 15 minute intervals: for example, if a person might say he spent 15 minutes on a task, mark 0.25 (1 quarter hour). Round any amount up to 15 minutes up to 15 minutes; round all other amounts to the closest 15 minute interval (e.g. if 35 minutes, round to 30 minutes, entering 0.5 hours). [enter # hours]*<br>*Question relevant when: ${E.27} !='997' and ${E.27a} !='0'*<br>*Response constrained to: .<=24 and .>0*		
E.28	E.28 If you disregard the outside help you receive from others, how do you and your partner divide the following task: Managing the weekly budget?	1	Always you
		2	Usually you
		3	Shared equally or done together
		4	Usually partner
		5	Always partner
		997	Not applicable
E.28a	E.28a During the last 7 days, on how many days did you do this task?<br>*[enter # days]*<br>*Question relevant when: ${E.28} !='997'*<br>*Response constrained to: .<=7*		
E.28b	E.28b On a typical day when you do this task, how many hours do you spend doing it?<br>*Please indicate time at 15 minute intervals: for example, if a person might say he spent 15 minutes on a task, mark 0.25 (1 quarter hour). Round any amount up to 15 minutes up to 15 minutes; round all other amounts to the closest 15 minute interval (e.g. if 35 minutes, round to 30 minutes, entering 0.5 hours). [enter # hours]*<br>*Question relevant when: ${E.28} !='997' and ${E.28a} !='0'*<br>*Response constrained to: .<=24 and .>0*		
E.29	E.29 If you disregard the outside help you receive from others, how do you and your partner divide the following task:	1	Always you

	Foraging for firewood for the household	2 Usually you<br>3 Shared equally or done together<br>4 Usually partner<br>5 Always partner<br>997 Not applicable
E.29a	E.29a During the last 7 days, on how many days did you do this task?<br>*[enter # days]*<br>*Question relevant when: ${E.29} !='997'*<br>*Response constrained to: .<=7*	
E.29b	E.29b On a typical day when you do this task, how many hours do you spend doing it?<br>*Please indicate time at 15 minute intervals: for example, if a person might say he spent 15 minutes on a task, mark 0.25 (1 quarter hour). Round any amount up to 15 minutes up to 15 minutes; round all other amounts to the closest 15 minute interval (e.g. if 35 minutes, round to 30 minutes, entering 0.5 hours). [enter # hours]*<br>*Question relevant when: ${E.29} !='997' and ${E.29a} !='0'*<br>*Response constrained to: .<=24 and .>0*	
E.30	E.30 If you disregard the outside help you receive from others, how do you and your partner divide the following task: Searching for fodder or grazing for the household's animals?	1 Always you<br>2 Usually you<br>3 Shared equally or done together<br>4 Usually partner<br>5 Always partner<br>997 Not applicable
E.30a	E.30a During the last 7 days, on how many days did you do this task?<br>*[enter # days]*<br>*Question relevant when: ${E.30} !='997'*<br>*Response constrained to: .<=7*	
E.30b	E.30b On a typical day when you do this task, how many hours do you spend doing it?<br>*Please indicate time at 15 minute intervals: for example, if a person might say he spent 15 minutes on a task, mark 0.25 (1 quarter hour). Round any amount up to 15 minutes up to 15 minutes; round all other amounts to the closest 15 minute interval (e.g. if 35 minutes, round to 30 minutes, entering 0.5 hours). [enter # hours]*<br>*Question relevant when: ${E.30} !='997' and ${E.30a} !='0'*<br>*Response constrained to: .<=24 and .>0*	
E.31	E.31 If you disregard the outside help you receive from others, how do you and your partner divide the following task: Carrying out any agricultural activity, whether farming, livestock, fishing, or forestry for salary, wages, or in-kind compensation?	1 Always you<br>2 Usually you<br>3 Shared equally or done together<br>4 Usually partner<br>5 Always partner<br>997 Not applicable
E.31a	E.31a During the last 7 days, on how many days did you do this task?<br>*[enter # days]*<br>*Question relevant when: ${E.31} !='997'*<br>*Response constrained to: .<=7*	
E.31b	E.31b On a typical day when you do this task, how many hours do you spend doing it?<br>*Please indicate time at 15 minute intervals: for example, if a person might say he spent 15 minutes on a task, mark 0.25 (1 quarter hour). Round any amount up to 15 minutes up to 15 minutes; round all other amounts to the closest 15 minute interval (e.g. if 35 minutes, round to 30 minutes, entering 0.5 hours). [enter # hours]*<br>*Question relevant when: ${E.31} !='997' and ${E.31a} !='0'*<br>*Response constrained to: .<=24 and .>0*	
E.32	E.32 If you disregard the outside help you receive from others, how do you and your partner divide the following task: Carrying out any agricultural activity, whether farming, livestock, fishing, or forestry for no pay	1 Always you<br>2 Usually you<br>3 Shared equally or done together<br>4 Usually partner<br>5 Always partner<br>997 Not applicable
E.32a	E.32a During the last 7 days, on how many days did you do this task?<br>*[enter # days]*<br>*Question relevant when: ${E.32} !='997'*<br>*Response constrained to: .<=7*	
E.32b	E.32b On a typical day when you do this task, how many hours do you spend doing it?<br>*Please indicate time at 15 minute intervals: for example, if a person might say he spent 15 minutes on a task, mark 0.25 (1 quarter hour). Round any amount up to 15 minutes up to 15 minutes; round all other amounts to the closest 15 minute interval (e.g. if 35 minutes, round to 30 minutes, entering 0.5 hours). [enter # hours]*<br>*Question relevant when: ${E.32} !='997' and ${E.32a} !='0'*	

	Response constrained to: .<=24 and .>0	
E.33	E.33 If you disregard the outside help you receive from others, how do you and your partner divide the following task:<br>Operating a non-farm business for cash or profit for yourself, like a small shop or other income generating activity	1 Always you<br>2 Usually you<br>3 Shared equally or done together<br>4 Usually partner<br>5 Always partner<br>997 Not applicable
E.33a	E.33a During the last 7 days, on how many days did you do this task?<br>*[enter # days]*<br>*Question relevant when: ${E.33} !='997'*<br>*Response constrained to: .<=7*	
E.33b	E.33b On a typical day when you do this task, how many hours do you spend doing it?<br>*Please indicate time at 15 minute intervals: for example, if a person might say he spent 15 minutes on a task, mark 0.25 (1 quarter hour). Round any amount up to 15 minutes up to 15 minutes; round all other amounts to the closest 15 minute interval (e.g. if 35 minutes, round to 30 minutes, entering 0.5 hours). [enter # hours]*<br>*Question relevant when: ${E.33} !='997' and ${E.33a} !='0'*<br>*Response constrained to: .<=24 and .>0*	
E.34	E.34 If you disregard the outside help you receive from others, how do you and your partner divide the following task:<br>Working in a non-farm business belonging to a family member not for pay	1 Always you<br>2 Usually you<br>3 Shared equally or done together<br>4 Usually partner<br>5 Always partner<br>997 Not applicable
E.34a	E.34a During the last 7 days, on how many days did you do this task?<br>*[enter # days]*<br>*Question relevant when: ${E.34} !='997'*<br>*Response constrained to: .<=7*	
E.34b	E.34b On a typical day when you do this task, how many hours do you spend doing it?<br>*Please indicate time at 15 minute intervals: for example, if a person might say he spent 15 minutes on a task, mark 0.25 (1 quarter hour). Round any amount up to 15 minutes up to 15 minutes; round all other amounts to the closest 15 minute interval (e.g. if 35 minutes, round to 30 minutes, entering 0.5 hours). [enter # hours]*<br>*Question relevant when: ${E.34} !='997' and ${E.34a} !='0'*<br>*Response constrained to: .<=24 and .>0*	
E.35	E.35 If you disregard the outside help you receive from others, how do you and your partner divide the following task:<br>Repairing the house	1 Always you<br>2 Usually you<br>3 Shared equally or done together<br>4 Usually partner<br>5 Always partner<br>997 Not applicable
E.35a	E.35a During the last 7 days, on how many days did you do this task?<br>*[enter # days]*<br>*Question relevant when: ${E.35} !='997'*<br>*Response constrained to: .<=7*	
E.35b	E.35b On a typical day when you do this task, how many hours do you spend doing it?<br>*Please indicate time at 15 minute intervals: for example, if a person might say he spent 15 minutes on a task, mark 0.25 (1 quarter hour). Round any amount up to 15 minutes up to 15 minutes; round all other amounts to the closest 15 minute interval (e.g. if 35 minutes, round to 30 minutes, entering 0.5 hours). [enter # hours]*<br>*Question relevant when: ${E.35} !='997' and ${E.35a} !='0'*<br>*Response constrained to: .<=24 and .>0*	
E.36	E.36 If you disregard the outside help you receive from others, how do you and your partner divide the following task:<br>Making the bed	1 Always you<br>2 Usually you<br>3 Shared equally or done together<br>4 Usually partner<br>5 Always partner<br>997 Not applicable
E.36a	E.36a During the last 7 days, on how many days did you do this task?<br>*[enter # days]*<br>*Question relevant when: ${E.36} !='997'*<br>*Response constrained to: .<=7*	
E.36b	E.36b On a typical day when you do this task, how many hours do you spend doing it?<br>*Please indicate time at 15 minute intervals: for example, if a person might say he spent 15 minutes on a task, mark 0.25 (1 quarter hour). Round any amount up to 15 minutes up to 15 minutes; round all other amounts to the closest 15 minute interval (e.g. if 35*	

	minutes, round to 30 minutes, entering 0.5 hours). [enter # hours]<br>*Question relevant when: ${E.36} !='997' and ${E.36a} !='0'*<br>*Response constrained to: .<=24 and .>0*		
E.37	E.37 During the last 7 days, on how many days did you spend social time with family or friends?<br>*[enter # days]*<br>*Response constrained to: .<=7*		
E.37a	E.37a On a typical day when you did this task, how many hours do you spend doing it?<br>*Please indicate time at 15 minute intervals: for example, if a person might say he spent 15 minutes on a task, mark 0.25 (1 quarter hour). Round any amount up to 15 minutes up to 15 minutes; round all other amounts to the closest 15 minute interval (e.g. if 35 minutes, round to 30 minutes, entering 0.5 hours). [enter # hours]*<br>*Question relevant when: ${E.37} !='0'*<br>*Response constrained to: .<=24 and .>0*		
E.38	E.38 During the last 7 days, on average, how many hours did you sleep per day?<br>*Please indicate time at 15 minute intervals: for example, if a person might say he spent 15 minutes on a task, mark 0.25 (1 quarter hour). Round any amount up to 15 minutes up to 15 minutes; round all other amounts to the closest 15 minute interval (e.g. if 35 minutes, round to 30 minutes, entering 0.5 hours). [enter # hours]*<br>*Response constrained to: .<=24 and .>0*		
E10	E10 How much do you want your partner to be involved in household tasks?<br>*READ RESPONSE OPTIONS.*	1	I'm happy with his current involvement
		2	I'd like him to be more involved
		3	I'd like him to be less involved
		998	I don't know
survey > PART 6: ALCOHOL<br>*Group relevant when: ${A01} >=18*			
note6	Now I would to ask you some questions about alcohol. Please remember that all responses will be kept confidential. If you do not want to answer a question, tell me and I will go on to the next question.		
F01	F01 In the past year (since the last interview), how many times have you had a drink containing alcohol?<br>*READ RESPONSE OPTIONS*	0	Never
		1	A few times in the last 9 months
		2	Once every 2 months
		3	Once a month
		4	A couple times a month
		5	Once or twice a week
		6	Every day or almost every day
		998	Do not know
		999	Refused to answer
F01b	F01b In the past year (since the last interview), how often did you drink so much that you became drunk?<br>*READ RESPONSE OPTIONS*<br>*Question relevant when: ${F01} !='0'*	0	Never
		1	A few times in the last 9 months
		2	Once every 2 months
		3	Once a month
		4	A couple times a month
		5	Once or twice a week
		6	Every day or almost every day
		998	Do not know
		999	Refused to answer
F03	F03 In the past year (since the last interview), how often has your partner had a drink containing alcohol?	0	Never
		1	Rarely
		2	Sometimes
		3	Often
		998	I don't know
		999	Refused to answer
F04	F04 In the past year (since the last interview), how often did your partner drink so much that he became drunk?<br>*READ RESPONSE OPTIONS*<br>*Question relevant when: ${F03} !='0'*	0	Never (drinks but does not get drunk)
		1	A few times in the past year.
		2	Once every 2 months
		3	Once a month
		4	A couple times a month
		5	Once or twice a week
		6	Every day or almost every

			day
		998	Do not know
		999	Refused to answer
F05	F05 In the past year (since the last interview), how often has your partner failed to do what was normally expected of him because of his drinking? *Question relevant when: ${F03} !='0'*	0	Never
		1	Rarely
		2	Sometimes
		3	Often
		998	I don't know
		999	Refused to answer
F06	F06 Has your partner ever injured himself or someone else because of his drinking? *Question relevant when: ${F03} !='0'*	0	No
		1	Yes
		998	Don't know
		999	Refused to answer
survey > PART 7: VIOLENCE *Group relevant when: ${A01} >=18*			
note7	I am going to ask you some questions about things which happened in your relationship with your husband/partner in the last year (since the last interview). The questions ask you how often you have experienced a range of different things in the last year. You might find these questions more difficult to answer, but we really hope you will answer them openly. The research is very important in trying to understand the lives of women in Rwanda. Remember that all of your responses will be kept confidential. You do not have to respond if you do not want to.		
G01	G01 I am now going to ask you about some situations that happen to many women and that your partner may have done to you. Please choose the answer that best describes the period covering the past year: *READ RESPONSE OPTIONS. These questions apply to the period of time since the last interview in November/December of last year (in the past year).*		
G01a	G01a In the past year (since the last interview), how many times has your partner become angry if you talked to other men?	0	Never
		1	Once
		2	A few times
		3	Frequently
		999	Refused to answer
		997	Not applicable
G01b	G01b In the past year (since the last interview), how many times has your partner insisted on knowing where you are at all times?	0	Never
		1	Once
		2	A few times
		3	Frequently
		999	Refused to answer
		997	Not applicable
G01c	G01c In the past year (since the last interview), how many times has your partner tried to keep you from seeing your friends?	0	Never
		1	Once
		2	A few times
		3	Frequently
		999	Refused to answer
		997	Not applicable
G01d	G01d In the past year (since the last interview), how many times has your partner tried to restrict contact with your family of birth?	0	Never
		1	Once
		2	A few times
		3	Frequently
		999	Refused to answer
		997	Not applicable
G02	G02 Is your partner often suspicious that you are unfaithful?	0	No
		1	Yes
		998	Don't know
		999	Refused to answer
G03	G03 I am now going to ask you another series of questions about things that happen to many women and that your partner may have done to you. Please choose the answer that best describes the period covering the past year: *READ RESPONSE OPTIONS. These questions apply to the past year / the period of time since the last interview in November/December of last year.*		
G03a	G03a In the past year (since the last interview), how many times has your partner slapped you or thrown something at you that could hurt you?	0	Never
		1	Once
		2	A few times
		3	Frequently
		999	Refused to answer

		997	Not applicable
G03b	G03b In the past year (since the last interview), how many times has your partner pushed or shoved you?	0	Never
		1	Once
		2	A few times
		3	Frequently
		999	Refused to answer
		997	Not applicable
G03c	G03c In the past year (since the last interview), how many times has your partner hit you with a fist or with something else that could hurt you?	0	Never
		1	Once
		2	A few times
		3	Frequently
		999	Refused to answer
		997	Not applicable
G03d	G03d In the past year (since the last interview), how many times has your partner kicked, or dragged, or beaten, or choked or burned you?	0	Never
		1	Once
		2	A few times
		3	Frequently
		999	Refused to answer
		997	Not applicable
G03e	G03e In the past year (since the last interview), how many times has your partner threatened to use or actually used a knife or stick against you?	0	Never
		1	Once
		2	A few times
		3	Frequently
		999	Refused to answer
		997	Not applicable
G03f	G03f During any of the incidents I just asked you about, how often were your children present?<br>*(If respondent does not have children, please mark not applicable.)*<br>*Question relevant when: not((${G03a} ='0' or ${G03a} ='999') and (${G03b} ='0' or ${G03b} ='999') and (${G03c} ='0' or ${G03c} ='999') and (${G03d} ='0' or ${G03d} ='999') and (${G03e} ='0' or ${G03e} ='999'))*	0	Never
		1	Once
		2	A few times
		3	Frequently
		999	Refused to answer
		997	Not applicable
G03g	G03g In the past year (since the last interview), how many times has your partner forced you to have sex when you did not want to?	0	Never
		1	Once
		2	A few times
		3	Frequently
		999	Refused to answer
		997	Not applicable
G03h	G03h In the past year (since the last interview), how many times has your partner insulted you or deliberately made you feel bad about yourself?	0	Never
		1	Once
		2	A few times
		3	Frequently
		999	Refused to answer
		997	Not applicable
G03i	G03i In the past year (since the last interview), how many times has your partner belittled or humiliated you in front of other people?	0	Never
		1	Once
		2	A few times
		3	Frequently
		999	Refused to answer
		997	Not applicable
G03j	G03j In the past year (since the last interview), how many times has your partner done things to scare or intimidate you on purpose? For example, by the way he looked at you, or by yelling or smashing things?	0	Never
		1	Once
		2	A few times
		3	Frequently
		999	Refused to answer
		997	Not applicable
G03k	G03k In the past year (since the last interview), how many times has your partner threatened to hurt you?	0	Never
		1	Once
		2	A few times
		3	Frequently
		999	Refused to answer

		997	Not applicable
G03l	G03l In the past year (since the last interview), how many times has your partner hurt people you care about as a way of hurting you, or damaged things of importance to you?	0	Never
		1	Once
		2	A few times
		3	Frequently
		999	Refused to answer
		997	Not applicable
G03m	G03m In the past year (since the last interview), how many times has your partner prohibited you from getting a job, or going to work, or trading or earning money?	0	Never
		1	Once
		2	A few times
		3	Frequently
		999	Refused to answer
		997	Not applicable
G03n	G03n In the past year (since the last interview), how many times has your partner taken your earnings against your will?	0	Never
		1	Once
		2	A few times
		3	Frequently
		999	Refused to answer
		997	Not applicable
G03o	G03o In the past year (since the last interview), how many times has your partner thrown you out of the house?	0	Never
		1	Once
		2	A few times
		3	Frequently
		999	Refused to answer
		997	Not applicable
G03p	G03p In the past year (since the last interview), how often has your partner kept money from his earnings for alcohol, or tobacco, or other things for himself when he knew you were finding it hard to afford the household expenses?	0	Never
		1	Once
		2	A few times
		3	Frequently
		999	Refused to answer
		997	Not applicable
G03q	G03q In the past year (since the last interview), how often have you consented to sexual activity because you were afraid of what your partner might do if you refused?	0	Never
		1	Once
		2	A few times
		3	Frequently
		999	Refused to answer
		997	Not applicable
G03r	G03r In the past year (since the last interview), how often has your partner threatened to hurt your children?<br>*If respondent does not have children, please mark not applicable.*	0	Never
		1	Once
		2	A few times
		3	Frequently
		997	Not applicable
		999	Refused to answer
G03s	G03s In the past year (since the last interview), how often has your partner threatened to take your children away from you?<br>*If respondent does not have children, please mark not applicable.*	0	Never
		1	Once
		2	A few times
		3	Frequently
		997	Not applicable
		999	Refused to answer
G03t	G03t In the past year (since the last interview), how often have you hit or physically hurt your partner when he was not hitting or physically mistreating you first?	0	Never
		1	Once
		2	A few times
		3	Frequently
		999	Refused to answer
		997	Not applicable
G03u	G03u In the past year (since the last interview), how often have you felt afraid of your partner or what he might do to you, or to your children?	0	Never
		1	Once
		2	A few times
		3	Frequently
		999	Refused to answer

		997	Not applicable
G03v	G03v In the past year (since the last interview), how often has your partner prohibited you from spending the money you earned how you wanted to spend it?	0	Never
		1	Once
		2	A few times
		3	Frequently
		999	Refused to answer
		997	Not applicable
note_thanks_7	NOTE TO THE INTERVIEWER: This can be a very difficult section of the survey. Below we provide some sample texts that you can choose from to thank the respondent for sharing her experiences with you. Please choose the one that feels relevant based on your interview:<br><br>If the woman disclosed violence:<br><br>I would like to thank you very much for helping us. I realize that these questions may have been difficult for you to answer. It is only from hearing from women themselves that we can really understand women's experiences.<br>From what you have told me, I can tell that you have had some difficult times in your life. I can also see that you are strong and have survived through difficult circumstances.<br>At the end of the interview I will share with you some information on organizations that provide support to women in your sector. Please contact them if you feel that you need their assistance, or share the list with another woman who may need these services. Thank you again for answering my questions.		
note_thanks_7a	NOTE TO THE INTERVIEWER: This can be a very difficult section of the survey. Below we provide some sample texts that you can choose from to thank the respondent for sharing her experiences with you. Please choose the one that feels relevant based on your interview:<br><br>If the woman did not disclose violence:<br><br>I would like to thank you very much for helping us. I appreciate the time that you have taken. I understand that these are personal questions, but it is only by hearing from women themselves that we can really understand relationships and families.<br><br>In case you ever hear of another woman who needs help, at the end of the interview, I will share with you some information on organizations that provide support to women in your sector. You can share this information with those who may need it.		
survey > PART 8. GENDER ATTITUDES<br>*Group relevant when: ${A01} >=18*			
note_8	The next set of questions will ask you about your views on relations between men and women. Please tell me if you strongly agree', 'agree' 'neither agree nor disagree', 'disagree' or 'strongly disagree' with the following statements.		
H01	H01 A woman’s most important role is to take care of her home and cook for her family.	1	Strongly agree
		2	Agree
		3	Neither agree nor disagree
		4	Disagree
		5	Strongly disagree
		999	Refused to answer
H02	H02 Changing, bathing and feeding the kids are the mother's responsibility.	1	Strongly agree
		2	Agree
		3	Neither agree nor disagree
		4	Disagree
		5	Strongly disagree
		999	Refused to answer
H04	H04 A man should have the final word about decisions in his home.	1	Strongly agree
		2	Agree
		3	Neither agree nor disagree
		4	Disagree
		5	Strongly disagree
		999	Refused to answer
H05	H05 A man should be respected as the head of the household.	1	Strongly agree
		2	Agree
		3	Neither agree nor disagree
		4	Disagree
		5	Strongly disagree
		999	Refused to answer

H10	H10 A good woman never questions her husband's decisions, even if she disagrees with them.	1	Strongly agree
		2	Agree
		3	Neither agree nor disagree
		4	Disagree
		5	Strongly disagree
		999	Refused to answer
H11	H11 It is a woman’s responsibility to avoid getting pregnant.	1	Strongly agree
		2	Agree
		3	Neither agree nor disagree
		4	Disagree
		5	Strongly disagree
		999	Refused to answer
H13	H13 It is perfectly acceptable for women to work outside the home to help support the family economically	1	Strongly agree
		2	Agree
		3	Neither agree nor disagree
		4	Disagree
		5	Strongly disagree
		999	Refused to answer
H16	H16 A woman who carries a condom on her is a “prostitute."	1	Strongly agree
		2	Agree
		3	Neither agree nor disagree
		4	Disagree
		5	Strongly disagree
		999	Refused to answer
H19	H19 It is natural and right that men have more power than women in the family.	1	Strongly agree
		2	Agree
		3	Neither agree nor disagree
		4	Disagree
		5	Strongly disagree
		999	Refused to answer
H20	H20 Sexual violence (rape) does exist within married couples.	1	Strongly agree
		2	Agree
		3	Neither agree nor disagree
		4	Disagree
		5	Strongly disagree
		999	Refused to answer
H21	H21 Sometimes a woman deserves to be beaten.	1	Strongly agree
		2	Agree
		3	Neither agree nor disagree
		4	Disagree
		5	Strongly disagree
		999	Refused to answer
H22	H22 It is okay for a woman to initiate sex.	1	Strongly agree
		2	Agree
		3	Neither agree nor disagree
		4	Disagree
		5	Strongly disagree
		999	Refused to answer
H33	H33 If a man cooks or cleans, it is shameful for his wife.	1	Strongly agree
		2	Agree
		3	Neither agree nor disagree
		4	Disagree
		5	Strongly disagree
		999	Refused to answer
H23	H23 A man must make the final decision on how money is spent in the family.	1	Strongly agree
		2	Agree
		3	Neither agree nor disagree
		4	Disagree
		5	Strongly disagree
		999	Refused to answer

H24	H24 A woman should tolerate violence in order to keep her family together.	1	Strongly agree
		2	Agree
		3	Neither agree nor disagree
		4	Disagree
		5	Strongly disagree
		999	Refused to answer
H25	H25 If money for schooling is scarce, it is better to spend it on boys first.	1	Strongly agree
		2	Agree
		3	Neither agree nor disagree
		4	Disagree
		5	Strongly disagree
		999	Refused to answer
H26	H26 If children do wrong they should be beaten.	1	Strongly agree
		2	Agree
		3	Neither agree nor disagree
		4	Disagree
		5	Strongly disagree
		999	Refused to answer
H28	H28 Men can take care of children just as well as women can.	1	Strongly agree
		2	Agree
		3	Neither agree nor disagree
		4	Disagree
		5	Strongly disagree
		999	Refused to answer
H29	H29 A woman must tolerate all the challenges she faces in her household (that's the way of things)	1	Strongly agree
		2	Agree
		3	Neither agree nor disagree
		4	Disagree
		5	Strongly disagree
		999	Refused to answer
H31	H31 If a husband tells his friends that he makes joint decisions with his wife his friends would not respect him.	1	Strongly agree
		2	Agree
		3	Neither agree nor disagree
		4	Disagree
		5	Strongly disagree
		999	Refused to answer
H32	H32 A man who is seen cooking or cleaning his house will be ridiculed by others	1	Strongly agree
		2	Agree
		3	Neither agree nor disagree
		4	Disagree
		5	Strongly disagree
		999	Refused to answer
note_h	Now, I'd like to ask you some questions about violence in your community. Remember that everything you tell me will be kept confidential.		
H36	H36 If a husband beats up his wife, do you think others outside the couple should intervene?	0	No
		1	Yes
H37	H37 In your opinion, does a man have a good reason to hit his wife if:		
H37a	H37a In your opinion, does a man have a good reason to hit his wife if: She disobeys him	0	No
		1	Yes
		999	Refused to answer
H37b	H37b In your opinion, does a man have a good reason to hit his wife if: She refuses to have sex with him	0	No
		1	Yes
		999	Refused to answer
H37c	H37c In your opinion, does a man have a good reason to hit his wife if He finds out that she has been unfaithful	0	No
		1	Yes
		999	Refused to answer
H37d	H37d In your opinion, does a man have a good reason to hit his wife if: She neglects taking care of the children	0	No
		1	Yes
		999	Refused to answer
H37e	H37e In your opinion, does a man have a good reason to hit his wife if: She does not complete the household work to his	0	No

	satisfaction	1	Yes
		999	Refused to answer
H37f	H37f In your opinion, does a man have a good reason to hit his wife if: She accuses him of being unfaithful	0	No
		1	Yes
		999	Refused to answer
H37g	H37g In your opinion, does a man have a good reason to hit his wife if: She spends money without consulting him	0	No
		1	Yes
		999	Refused to answer
H37h	H37h In your opinion, does a man have a good reason to hit his wife if: She does not welcome guests in their home to his satisfaction	0	No
		1	Yes
		999	Refused to answer
H37i	H37i In your opinion, does a man have a good reason to hit his wife if : When he finds that she did not have food prepared	0	No
		1	Yes
		999	Refused to answer
H38	H38 If a married woman has been beaten up by her husband, do you think it is okay for her to tell others?	0	No
		1	Yes

survey > PART 9. Campaigns and Support Networks

Group relevant when: ${A01} >=18

note9	We are almost to the end of the survey. Thank you for answering the questions so far.		
I01	I01 I am going to read a series of statements, please tell me if you strongly agree, agree, neither agree nor disagree, disagree, or strongly disagree.		
I01a	I01a I am a role-model in my community	1	Strongly agree
		2	Agree
		3	Neither agree nor disagree
		4	Disagree
		5	Strongly disagree
I01b	I01b I feel that my life is of use to others	1	Strongly agree
		2	Agree
		3	Neither agree nor disagree
		4	Disagree
		5	Strongly disagree
I01c	I01c I have a lot to be happy about	1	Strongly agree
		2	Agree
		3	Neither agree nor disagree
		4	Disagree
		5	Strongly disagree
I01d	I01d I feel that I am a good wife	1	Strongly agree
		2	Agree
		3	Neither agree nor disagree
		4	Disagree
		5	Strongly disagree
I01e	I01d I feel that I am a good mother	1	Strongly agree
		2	Agree
		3	Neither agree nor disagree
		4	Disagree
		5	Strongly disagree
I.21	I.21 I am going to read a few statements. I want you to tell me how often you felt this way in the PAST WEEK: rarely or none of the time, some or a little of the time, occasionally or a moderate amount of time, or all of the time.		
I.21a	I.21a I was bothered by things that usually don't bother me.	1	Rarely of none of the time (less than 1 day in the week)
		2	Some or a little of the time (1-2 days of the week)
		3	Occasionally or a moderate amount of time (3-4 days of the week)
		4	All of the time (5-7 days of the week)
		999	Refused to answer
I21b	I21b I had trouble keeping my mind on what I was doing	1	Rarely of none of the time

			(less than 1 day in the week)
		2	Some or a little of the time (1-2 days of the week)
		3	Occasionally or a moderate amount of time (3-4 days of the week)
		4	All of the time (5-7 days of the week)
		999	Refused to answer
I21c	I21c I felt depressed	1	Rarely of none of the time (less than 1 day in the week)
		2	Some or a little of the time (1-2 days of the week)
		3	Occasionally or a moderate amount of time (3-4 days of the week)
		4	All of the time (5-7 days of the week)
		999	Refused to answer
I21d	I21d I felt that everything I did was an effort	1	Rarely of none of the time (less than 1 day in the week)
		2	Some or a little of the time (1-2 days of the week)
		3	Occasionally or a moderate amount of time (3-4 days of the week)
		4	All of the time (5-7 days of the week)
		999	Refused to answer
I21e	I21e I felt hopeful about the future	1	Rarely of none of the time (less than 1 day in the week)
		2	Some or a little of the time (1-2 days of the week)
		3	Occasionally or a moderate amount of time (3-4 days of the week)
		4	All of the time (5-7 days of the week)
		999	Refused to answer
I21f	I21f I felt fearful	1	Rarely of none of the time (less than 1 day in the week)
		2	Some or a little of the time (1-2 days of the week)
		3	Occasionally or a moderate amount of time (3-4 days of the week)
		4	All of the time (5-7 days of the week)
		999	Refused to answer
I21g	I21g My sleep was restless	1	Rarely of none of the time (less than 1 day in the week)
		2	Some or a little of the time (1-2 days of the week)
		3	Occasionally or a moderate amount of time (3-4 days of the week)
		4	All of the time (5-7 days of the week)
		999	Refused to answer
I21h	I21h I was happy	1	Rarely of none of the time (less than 1 day in the week)

		2 Some or a little of the time (1-2 days of the week)<br>3 Occasionally or a moderate amount of time (3-4 days of the week)<br>4 All of the time (5-7 days of the week)<br>999 Refused to answer
I21i	I21i I felt lonely	1 Rarely of none of the time (less than 1 day in the week)<br>2 Some or a little of the time (1-2 days of the week)<br>3 Occasionally or a moderate amount of time (3-4 days of the week)<br>4 All of the time (5-7 days of the week)<br>999 Refused to answer
I21j	I21j I could not "get going"	1 Rarely of none of the time (less than 1 day in the week)<br>2 Some or a little of the time (1-2 days of the week)<br>3 Occasionally or a moderate amount of time (3-4 days of the week)<br>4 All of the time (5-7 days of the week)<br>999 Refused to answer
I02	I02 In the last year (since the last interview), have you participated in an activity in your community or workplace to talk about men's use of violence against women?<br>*Question relevant when: ${partcipant_group} ='2'*	0 No<br>1 Yes<br>999 Refused to answer
I03	I03 In the last year (since the last interview), have you participated in an activity in your community or workplace to talk about men's role in their children's lives?<br>*Question relevant when: ${partcipant_group} ='2'*	0 No<br>1 Yes
I04	I04 In the last year (since the last interview), have you participated in any activity in your community or workplace to talk about maternal health?<br>*Question relevant when: ${partcipant_group} ='2'*	0 No<br>1 Yes
I06	I06 Do you know anyone who has participated in or facilitated a Bandebereho group?<br>*Question relevant when: ${partcipant_group} ='2'*	0 No<br>1 Yes, one person<br>2 Yes, multiple people
I06a	I06a What is this person or persons' relationship to you?<br>*MARK ALL THAT APPLY.*<br>*Question relevant when: ${partcipant_group} ='2' and ${I06} !='0'*	1 Close family member<br>2 Close friend<br>3 Distant relative<br>4 Acquintance/someone you don't know very well<br>5 Neighbor<br>6 Other
I06b	I06b Have you ever sought advice from one of RWAMREC's Bandebereho facilitators or group members?<br>*Question relevant when: ${partcipant_group} ='2' and ${I06} !='0'*	0 No<br>1 Yes<br>998 I don't know
I07	I07 How many times have you sought advice from one of RWAMREC's Bandebereho facilitatators or group members?<br>*Question relevant when: ${partcipant_group} ='2' and ${I06} !='0' and ${I06b} ='1'*	0 Never<br>1 Once<br>2 A few times (2-5 times)<br>3 Many times (more than 5 times)
noteI	I'm going to ask you a few questions about activities you have participated in during the past year (since the time of the last interview).<br>*Question relevant when: ${partcipant_group} ='1'*	
I08	I08 In the past year (since the last interview), did you participate in any activity, other than the RWAMREC Bandebereho parents' sessions, which talked about men's use of violence against women?<br>*Question relevant when: ${partcipant_group} ='1'*	0 No<br>1 Yes
I09	I09 In the past year (since the last interview), did you participate in any activity, other than the RWAMREC Bandebereho	0 No

	parents' sessions, which talked about men's role in their children's lives?<br>*Question relevant when: ${partcipant_group} ='1'*	1 Yes
I10	I10 In the past year (since the last interview), did you participate in any activity, other than the RWAMREC Bandebereho parents' sessions, which talked about maternal health?<br>*Question relevant when: ${partcipant_group} ='1'*	0 No<br>1 Yes
I15	I15 Have you shared what you discussed in the RWAMREC Bandebereho parents' sessions with anyone else?<br>*Question relevant when: ${partcipant_group} ='1'*	0 No<br>1 Yes
I16	I16 If yes, with whom did you share what you learned?<br>*CHECK ALL THAT APPLY*<br>*Question relevant when: ${partcipant_group} ='1' and ${I15} !='0'*	1 My partner<br>2 My child(ren)<br>3 Close family member<br>4 Close friend<br>5 Distant relative<br>6 Acquintance/someone I don't know very well<br>7 Neighbor<br>8 the public (e.g. gave testimony at umuganda)
I.21y	I.21 Over the past year and a half, you have participated in our interviews up to 3 times. I have asked you some easy and some difficult questions. How has talking about these things made you feel?	1 Good<br>2 Bad<br>3 Neither good nor bad
end_note	We have now reached the end of the survey.<br><br>Thank you very much for taking the time to answer our questions.<br><br>Please know that all the responses will be kept confidential, and cannot be linked back to your name.<br>[ASK THE PARTICIPANT IF SHE WANTS THE REFERRAL SHEET OF SERVICES/ SUPPORT ORGANIZATIONS IN HER SECTOR. PROVIDE IT TO THE RESPONDENT IF SHE WANTS IT.]	
