# Supplementary material for: Gender-transformative Bandebereho couples’ intervention to promote male engagement in reproductive and maternal health and violence prevention in Rwanda: Findings from a randomized controlled trial
Source: PLoS One. 2018 Apr 4;13(4):e0192756. doi: 10.1371/journal.pone.0192756 (PMC5884496; doi:10.1371/journal.pone.0192756)
Supplement: S2 File — (PDF) [file pone.0192756.s002.pdf]

Bandebereho_21_Followup_Female_Survey_Final

Field	Question	Answer
survey		
survey > coversheet		
partcipant_group	UMUKARANI W'IBARURA: Genzura ku rutonde rw'abitabira hanyuma wemeze group aherereyemo	1 Treatment group<br>2 Control group
survey > Igice cya 1: Umwirondoro w'ubazwa n'ibindi biranga urugo rwe		
A01	A01 Ufite imyaka ingahe?<br>*Niba usubiza atazi imyaka ye andika 998: niba yanze gusubiza andika 999.[Andika imyaka]*<br>*Response constrained to: .<=60 or .=998 or .=999*	
A02	A02 Ni uruhe rwego rw'amashuri wize?<br>*Question relevant when: ${A01} >=18*	0 Ntayo<br>1 Yacikije amashuri abanza<br>2 Yarangije amashuri abanza<br>3 Yacikije amashuri yisumbuye<br>4 Yarangije amashuri yisumbuye<br>5 Amashuri y'imyuga/TTC<br>6 Kaminuza<br>999 Yanze gusubiza
A04	A04 Ni uwuhe murimo wingezi ukora?<br>*Question relevant when: ${A01} >=18*	1 Afite akazi kamuhemba<br>2 Arikorera<br>3 Nta kazi afite, arimo kugashaka<br>4 Nta kazi afite, nta n'ako ashaka<br>5 Ntashobora gukora/Yaramugaye<br>999 Yanze gusubiza
survey > Igice cya 1: Umwirondoro w'ubazwa n'ibindi biranga urugo rwe > -<br>*Group relevant when: ${A01} >=1*		
A05	A05 Ni kangahe umuryango wawe ubona mu buryo buhagije ibi bikurikira?<br>*Baza inshuro ubazwa adashobora kwigeza kuri bimwe nkenerwa bikurikira (atari ibikenerwa byose). Urugero, niba ubanzwa afite inzu ariko akaba ashobora kubona ibyokurya gusa kensi, hitamo "kenshi." Niba ashobora kubona ibyo kurya rimwe na rimwe, hitamo rimwe na rimwe. MUSOMERE IBISUBIZO BYOSE BYATANZWE:*<br>*Question relevant when: ${A01} >=18*	1 Nta na rimwe<br>2 Rimwe na rimwe<br>3 Kenshi<br>4 Buri gihe
A05a	A05a Ibyangombwa nkenerwa (ibiribwa cyangwa icumbi…)<br>*Question relevant when: ${A01} >=18*	1 Nta na rimwe<br>2 Rimwe na rimwe<br>3 Kenshi<br>4 Buri gihe
A05b	A05b Iby'ingenzi (imyambaro, cyangwa amafaranga y'ishuri, cyangwa mitiweli…)<br>*Question relevant when: ${A01} >=18*	1 Nta na rimwe<br>2 Rimwe na rimwe<br>3 Kenshi<br>4 Buri gihe
A05c	A05c Iby'inyongera (gutembera cyangwa impano…)<br>*Question relevant when: ${A01} >=18*	1 Nta na rimwe<br>2 Rimwe na rimwe<br>3 Kenshi<br>4 Buri gihe
note_a	Ngiye kukubaza ibibazo byerekeye kubantu batuye mu rugo rwanyu.<br>*Question relevant when: ${A01} >=18*	
A06	A06 Mu rugo rwawe haba abantu bakuru (bafite imyaka 18 cyangwa hejuru yayo) b'igitsina gabo bangahe?<br>*[Andika umubare]*<br>*Question relevant when: ${A01} >=18*	
A07	A07 Mu rugo rwawe haba abantu bakuru (bafite imyaka 18 cyangwa hejuru yayo) b'igitsina gore bangahe wowe utibariyemo?<br>*[Andika umubare]*<br>*Question relevant when: ${A01} >=18*	
A08	A08 Mu rugo rwawe haba abana bangahe b'abahungu (abana bari munsi y'imyaka 18)?<br>*Abana b'abahungu bahaba bose n'ubwo baba atari abawe. [Andika umubare]*<br>*Question relevant when: ${A01} >=18*	
A09	A09 Mu rugo rwawe haba abana bangahe b'abakobwa (abana bari munsi y'imyaka 18)?<br>*Abana b'abakobwa bahaba bose n'ubwo baba atari abawe. [Andika umubare]*<br>*Question relevant when: ${A01} >=18*	

A10	A10 Ufite abana bangahe?<br>*Tubwire abo wabyaye kandi bariho. [Andika umubare w'abana]*<br>*Question relevant when: ${A01} >=18*	
A11	A11 Tubwire imyaka n'igitsina bya buri mwana wawe wabyaye?<br>*Saba usubiza kukubwira abana be bose uhereye ku mukuru.Niba ubazwa atazi imyaka y'umwana andika 998. Andika 0 niba umwana ataruzuza umwaka w'amavuko hanyuma wandike umubare w'amazi ku kibazo gikurikira.*<br>*Question relevant when: ${A10} !='0' and ${A01} >=18*	
survey > Igice cya 1: Umwirondoro w'ubazwa n'ibindi biranga urugo rwe > Igitsina n'imyaka by'abana (1)<br>*Group relevant when: ${A10} !='0' and ${A01} >=18*		(Repeated group)
A11a	A11a Imyaka y'umwana wa 1<br>*[Andika Imyaka]*	
A11b	A11b Umwana wa 1 afite amezi angahe?<br>*Question relevant when: ${A11a} ='0'*<br>*Response constrained to: .<=11*	
A11c	A11c Igitsina cy'Umwana wa 1	1 Gore<br>2 Gabo
A12	A12 Ni nde ufite ubwishingizi bw'ubuzima mubaba mu rugo rwawe?<br>*HITAMO IBISUBIZO BYOSE BISHOBOKA*<br>*Question relevant when: ${A01} >=18*<br>*Response constrained to: count-selected(.)=1 or (count-selected(.)>=2 and not(selected(.,0)) and not(selected(.,998)) and not(selected(.,999)))*	0 Ntawe<br>1 Njyewe<br>2 Umugabo wanjye<br>3 Abana banjye (umwe cyangwa abana benshi)<br>998 Simbizi<br>5 Abandi<br>999 Yanze gusubiza
A13	A13 Ese mu byumweru 2 bishize wigeze ushyira amafaranga ku ruhande yo kwizigama ubariyemo n'ayo waba waratanze mu matsinda yo kwizigama cyangwa mu bimina?<br>*Question relevant when: ${A01} >=18*	0 Oya<br>1 Yego<br>999 Yanze gusubiza
survey > PART 2. Gutwita no kubyara<br>*Group relevant when: ${A01} >=18*		
note2	Muri iki gice ngiye kukubaza ibibazo birebana n'uko wumvaga umeze k'ukugira abana cyangwa kwitegura kugira umwana. Ndakwibutsa ko ibisubizo byawe bigirwa ibinga.	
B01	B01 Ese waba utwite?	0 Oya<br>1 Yego<br>999 Yanze gusubiza
B02	B02 Uteganya kubyara ryari? Andika umubare w'amezi asigaye kugirango umwana avuke<br>*Andika 998 niba usubiza atabizi. Niba umwana azavuka munsi y'ukwezi kumwe, andika 0 [Andika umubare w'amezi]*<br>*Question relevant when: ${B01} ='1'*<br>*Response constrained to: .<=9 or .=998*	
B03	B03 Kuri iyi nda utwite ubu, ni inshuro zingahe umaze kwisuzumisha muri gahunda yo gukurikirana abagore batwite?<br>*[Andika umubare]*<br>*Question relevant when: ${B01} ='1'*	
B04	B04 Kuri iyi nda utwite ubu, ni inshuro zingahe umugabo wawe yaguherekeje kwisuzumisha muri gahunda yo gukurikirana abagore batwite?<br>*Niba uzubiza atabizi/atabyibuka andika -98. [Andika umubare]*<br>*Question relevant when: ${B01} ='1' and ${B03} !='0'*<br>*Response constrained to: (.<= ${B03} and .>=0) or .=-98*	
B04a	B04a Ku isuzuma ry'abagore batwite uherukamo waherekejwe n'umugabo wawe, yaba:<br>*MUSOMERE IBISUBIZO BYOSE BYATANZWE*<br>*Question relevant when: ${B01} ='1' and ${B03} !='0' and ${B04} !='0'*	1 Yarakugejeje ku irembo ry'ivuriro cyangwa yaragutegerereje hanze yaryo<br>2 Yarakugejeje mu ivuriro ariko ntimwinjiranye mu cyumba basuzumiramo<br>3 Yarabaye hamwe nawe mu cyumba basuzumiramo nibura akanya mugihe wari kumwe n'ugukurikirana<br>4 Ikindi
B04b	B04b Wumvaga umerewe ute igihe umugabo wawe yaguherekezaga kwisuzumisha muri gahunda yo gukurikirana abagore batwite?<br>*MUSOMERE IBISUBIZO BYOSE BYATANZWE HANYUMA UHITEMO IBISUBIZO BYOSE BISHOBOKA*<br>*Question relevant when: ${B01} ='1' and ${B03} !='0' and ${B04} !='0' and ${B04a} ='3'*	1 Numvaga merewe neza<br>2 Numvaga ntamerewe neza<br>3 Nari mfite isoni<br>4 Nari nishimye<br>5 Numvise nshyigikiwe n'umugabo wanjye<br>6 Nta kibazo byanteye

		7	Byarangoye cyangwa byantesheje umutwe
		8	Nungutse byinshi (ubumenyi)
		9	Narabikunze kujyana n'umugabo wanjye
		10	Sinabikunze kujyana n'umugabo wanjye
		11	Banyitayeho kurusha ibisanzwe
		12	Baranyubashye kurusha ibisanzwe
		13	Banyakiriye bwangu (sinagombye gutegereza)
		14	Numvise umugabo wanjye ashyize hamwe nanjye kurushaho
B06	B06 Wakwishimira ko umugabo wawe aba ari kumwe nawe mu cyumba cyo kubyariramo mu gihe uzaba uri kubyara?<br>*Question relevant when: ${B01} ='1'*	0	Oya
		1	Yego
		998	Simbizi/Simbihamya
B07	B07 Ndifuza kukubaza ku bijyanye n'inda uherutse gutwita y'umwana uheruka kubyara uriho. Mugihe wari ufite inda y'umwana uheruka kubyara uriho, ni inshuro zingahe wagiye kwisuzumisha muri gahunda yo gukurikirana abagore batwite?<br>*Andika 998 Niba usubiza nta gisubizo azi/atabyibuka [Andika umubare w'inshoro wagoyeyo]*<br>*Question relevant when: ${A10} !='0'*		
B08	B08 Mugihe wari ufite inda y'umwana uheruka kubyara bwanyuma kandi akaba akiriho, ni inshuro zingahe umugabo wawe yaguherekeje kwisuzumisha muri gahunda yo gukirikirana abagore batwite?<br>*Andika -98 Niba usubiza nta gisubizo azi/atabyibuka. [Andika umubare w'inshoro wagoyeyo]*<br>*Question relevant when: ${A10} !='0' and ${B07} !='0'*<br>*Response constrained to: (.<= ${B07} and .>=0) or .=-98*		
B08a	B08a Ku isuzuma ry'abagore batwite uherukamo waherekejwe n'umugabo wawe, yaba:<br>*MUSOMERE IBISUBIZO BYOSE BYATANZWE*<br>*Question relevant when: (${A10} !='0' and ${B01} ='0' and ${B07} !='0' and ${B08} !='0') or (${A10} !='0' and ${B01} ='1' and ${B04a} !='3' and ${B07} !='0' and ${B08} !='0')*	1	Yarakugejeje ku irembo ry'ivuriro cyangwa yaragutegerereje hanze yaryo
		2	Yarakugejeje mu ivuriro ariko ntimwinjiranye mu cyumba basuzumiramo
		3	Yarabaye hamwe nawe mu cyumba basuzumiramo nibura akanya mugihe wari kumwe n'ugukurikirana
		4	Ikindi
B08b	B08b Wumvaga umerewe ute igihe umugabo wawe yaguherekezaga kwisuzumisha muri gahunda yo gukurikirana abagore batwite?<br>*MUSOMERE IBISUBIZO BYOSE BYATANZWE HANYUMA UHITEMO IBISUBIZO BYOSE BISHOBOKA*<br>*Question relevant when: ${B08a} ='3'*	1	Numvaga merewe neza
		2	Numvaga ntamerewe neza
		3	Nari mfite isoni
		4	Nari nishimye
		5	Numvise nshyigikiwe n'umugabo wanjye
		6	Nta kibazo byanteye
		7	Byarangoye cyangwa byantesheje umutwe
		8	Nungutse byinshi (ubumenyi)
		9	Narabikunze kujyana n'umugabo wanjye
		10	Sinabikunze kujyana n'umugabo wanjye
		11	Banyitayeho kurusha ibisanzwe
		12	Baranyubashye kurusha ibisanzwe
		13	Banyakiriye bwangu (sinagombye gutegereza)
		14	Numvise umugabo wanjye ashyize hamwe nanjye

			kurushaho
B11	B11 Umugabo wawe yari he mu gihe wabyaraga ubuheruka?<br>*MUSOMERE IBISUBIZO BYOSE BYATANZWE, Mu gihe umugore yabyari ahandi hatari kwamuganga hitamo " Ntibimureba"*<br>*Question relevant when: ${A10} !='0'*	1	Yari aho nari ndi mu cyumva cyo kubyariramo
		2	Yari mu ivuriro ariko ntitwari hamwe mu cyumba cyo kubyariramo
		3	Ntiyari ku ivuriro
		997	Ntibimureba
		998	Simbizi/Simbyibuka
B11a	B11a Wumvaga umerewe ute igihe wari kumwe n'umugabo wawe mu cyumba ubwo warimo kubyara?<br>*MUSOMERE IBISUBIZO BYOSE BYATANZWE HANYUMA UHITEMO IBISUBIZO BYOSE BISHOBOKA*<br>*Question relevant when: ${A10} !='0' and ${B11} ='1'*	1	Numvaga merewe neza
		2	Numvaga ntamerewe neza
		3	Nari mfite isoni
		4	Nari nishimye
		5	Numvise nshyigikiwe n'umugabo wanjye
		6	Nta kibazo byanteye
		7	Byarangoye cyangwa byantesheje umutwe
		8	Nungutse byinshi (ubumenyi)
		9	Narabikunze kujyana n'umugabo wanjye
		10	Sinabikunze kujyana n'umugabo wanjye
		11	Banyitayeho kurusha ibisanzwe
		12	Baranyubashye kurusha ibisanzwe
		13	Numvise umugabo wanjye ashyize hamwe nanjye kurushaho
B13	B13 Nyuma y'ivuka ry'umwana muheruka kubyara uriho, wigeze ujya gukingiza uwo mwana cyangwa umugabo wawe yigeze amujyana kumukingiza cyangwa se mwembi mwigeze mujyana kumukingiza?<br>*Question relevant when: ${A10} !='0'*	0	Oya
		1	Yego, naramwijyaniye njye ubwanjye
		2	Yego, umugabo wanjye yaramujyanye
		3	Yego, umwana twaramujyanye twembi, tujyanye cyangwa buri wese amujyana ukwe
		997	Ntibimureba
B15	B15 Muri aka kanya ndagira ngo ngusabe gutekereza ku nda uheruka gutwita. Ni kangahe waganiriye n'umugabo wawe ku:<br>*MUSOMERE IBISUBIZO BYOSE BYATANZWE, Inda uheruga gutwita ishobora kuba inda utwise ubu cyangwa inda 'umwana wawe muto*<br>*Question relevant when: ${A10} !='0' or ${B01} ='1'*		
B15c	B15c Ibijyanye n'ubuvuzi mu gihe wari utwite<br>*Question relevant when: ${A10} !='0' or ${B01} ='1'*	1	Kenshi
		2	Rimwe na rimwe
		3	Gake gashoboka
		4	Nta na rimwe
		997	Ntibimureba
B15e	B15e Ibyabahangayikisha mu gihe mwifuza kubyara cyangwa kurera umwana<br>*Question relevant when: ${A10} !='0' or ${B01} ='1'*	1	Kenshi
		2	Rimwe na rimwe
		3	Gake gashoboka
		4	Nta na rimwe
		997	Ntibimureba
B15f	B15f Niba bishoboka ko mwatangira gukoresha uburyo bwo kuboneza urubyaro kugira ngo ubutaha uzatwite haciyemo igihe, n'igihe mwabitangirira<br>*Question relevant when: ${A10} !='0' or ${B01} ='1'*	1	Kenshi
		2	Rimwe na rimwe
		3	Gake gashoboka
		4	Nta na rimwe
		997	Ntibimureba
B16	B16 Ngiye kugusomera interuro. ndagusaba ko utekereza ku nda uheruka gutwita maze umbwire niba ibiyikubiyemo	1	Ndabyemera cyane

	ubyemera cyane, niba ubyemera, niba ntacyo wabivugaho, niba ubihakana, cyangwa niba utabyemera na gato: Ku nda mperuka gutwita, umugabo wanjye yanshishikarije cyangwa yamfashije kujya kwisuzumisha muri gahunda yo gukurikirana abagore batwite.<br>*Inda uheruga gutwita ishobora kuba inda utwise ubu cyangwa inda 'umwana wawe muto*<br>*Question relevant when: ${A10} !='0' or ${B01} ='1'*	2 Ndabyemera<br>3 Ntacyo nabivugaho<br>4 Simbyemera<br>5 Simbyemera na gato<br>999 Yanze gusubiza
B27	B27 Ubwo uheruka gutwita, umugabo wawe yaba hari ibikorwa yakoze muri ibi bikurikira:<br>*Inda uheruga gutwita ishobora kuba inda utwise ubu cyangwa inda 'umwana wawe muto*<br>*Question relevant when: ${A10} !='0' or ${B01} ='1'*	
B27a	B27a Ubwo uheruka gutwita, umugabo wawe yaba hari ibikorwa yakoze muri ibi bikurikira: Yatanze ubufasha bw'amafaranga (yo kwivuza, yo kwishyura ingendo cyangwa kwishyura ibyangombwa by'umwana)<br>*Question relevant when: ${A10} !='0' or ${B01} ='1'*	0 Oya<br>1 Yego<br>999 Yanze gusubiza<br>997 Ntibimureba
B27b	B27b Ubwo uheruka gutwita, umugabo wawe yaba hari ibikorwa yakoze muri ibi bikurikira: Yakoze imwe mu mirimo yo mu rugo nsanzwe nkora<br>*Question relevant when: ${A10} !='0' or ${B01} ='1'*	0 Oya<br>1 Yego<br>999 Yanze gusubiza<br>997 Ntibimureba
B27c	B27c Ubwo uheruka gutwita, umugabo wawe yaba hari ibikorwa yakoze muri ibi bikurikira: Yaguteguriye ifunguro cyangwa yarebye niba ufite indyo ikwiye yo kurya<br>*Question relevant when: ${A10} !='0' or ${B01} ='1'*	0 Oya<br>1 Yego<br>999 Yanze gusubiza<br>997 Ntibimureba
B27d	B27d Ubwo uheruka gutwita, umugabo wawe yaba hari ibikorwa yakoze muri ibi bikurikira: Yagushishikarije kwiyitaho (kuruhuka cyangwa kurya neza)<br>*Question relevant when: ${A10} !='0' or ${B01} ='1'*	0 Oya<br>1 Yego<br>999 Yanze gusubiza<br>997 Ntibimureba
B27e	B27e Ubwo uheruka gutwita, umugabo wawe yaba hari ibikorwa yakoze muri ibi bikurikira: Yakwitayeho aranakumva (yakubajije uko umerewe, yakubwiye amagambo agutera kugira imbaraga igihe utari umeze neza)<br>*Question relevant when: ${A10} !='0' or ${B01} ='1'*	0 Oya<br>1 Yego<br>999 Yanze gusubiza<br>997 Ntibimureba
B27f	B27f Ubwo uheruka gutwita, umugabo wawe yaba hari ibikorwa yakoze muri ibi bikurikira: Yaguteye inkunga mu buryo bwa roho, aranakuyobora (yasengeye gutwita kwawe ngo kugende neza, anasengera umwana ngo agire ubuzima bwiza)<br>*Question relevant when: ${A10} !='0' or ${B01} ='1'*	0 Oya<br>1 Yego<br>999 Yanze gusubiza<br>997 Ntibimureba
B27g	B27g Ubwo uheruka gutwita, umugabo wawe yaba hari ibikorwa yakoze muri ibi bikurikira: Yitaye ku bandi bana banyu (Niba bahari)<br>*Question relevant when: ${A10} !='0' or ${B01} ='1'*	0 Oya<br>1 Yego<br>999 Yanze gusubiza<br>997 Ntibimureba
survey > PART 3. Ubuzima bw'imyororokere<br>*Group relevant when: ${A01} >=18*		
note3	note3 Ngiye kukuganiriza ku birebana n'imyitwarire yawe ku bijyanye n'ubuzima bw'imyororokere. Nk'uko nakomeje kubikwizeza ibisubizo uribumpe biraba ibanga. Niba hari ikibazo wumva udashaka gusubiza turagisimbuka.	
C01	C01 Ese hari uburyo wowe cyangwa umugabo wawe mukoresha ubungubu bwo kwirinda cyangwa gutinza gusama?<br>*HITAMO IBISUBIZO BYOSE BISHOBOKA*<br>*Response constrained to: count-selected(.)=1 or (count-selected(.)>=2 and not(selected(.,0)) and not(selected(.,10)) and not(selected(.,999)))*	0 Ntabwo<br>1 Ibinini<br>2 Agakingirizo k'abagabo<br>3 Agakingirizo k'abagore<br>4 Inshinge<br>5 Agapira ko mu mura<br>6 umugabo wifungishize burundu<br>7 Umugore akoresheje uburyo bwa burundu cyangwa se yabazwe nyababyeyi<br>8 Agapira ko mu kuboko<br>9 Uburyo gakondo (kwifata, kwiyakana, urunigi,..)<br>10 Simbizi<br>11 Ubundi buryo<br>999 Yanze gusubiza
C02	C02 Waba warigeze ukoresha uburyo bwo kuboneza urubyaro umugabo wawe atabizi?	0 Oya<br>1 Yego<br>2 Ntibimureba<br>999 Yanze gusubiza

C02a	C02a Byaba byarabaye mu mwaka ushize (kuva ku ibazwa riheruka?)<br>*Question relevant when: ${C02} ='1'*	0<br>1<br>999	Oya<br>Yego<br>Yanze gusubiza
C07	C07 Ngiye kukubaza ibibazo bike bijyanye n'uko ubintu bishobora kuba byarabaye hagati yawe n'umugabo wawe mu mwaka ushize (kuva kwibazwa riheruka). Urambwira niba ibikubiyemo ubyemera cyane, niba ubyemera, niba utabyemera na gato, cyangwa Ntibimureba:		
C07a	C07a Mu mwaka ushize (kuva kwibazwa riheruka): Umugabo wanjye yambujije gukoresha uburyo bwo kwirinda gusama mu gihe nashakaga kubukoresha	1<br>2<br>3<br>4<br>997<br>999	Ndabyemera cyane<br>Ndabyemera<br>Simbyemera<br>Simbyemera na gato<br>Ntibimureba<br>Yanze gusubiza
C07b	C07b Mu mwaka ushize (kuva kwibazwa riheruka): Umugabo wanjye yagiye amvundira cyangwa atuma binkomerera gukoresha uburyo bwo kwirinda gusama mu gihe nashakaga kubukoresha	1<br>2<br>3<br>4<br>997<br>999	Ndabyemera cyane<br>Ndabyemera<br>Simbyemera<br>Simbyemera na gato<br>Ntibimureba<br>Yanze gusubiza
C07c	C07c Mu mwaka ushize (kuva kwibazwa riheruka): Umugabo wanjye yanteye gukoresha uburyo bwo kwirinda gusama mu gihe ntashakaga kubukoresha	1<br>2<br>3<br>4<br>997<br>999	Ndabyemera cyane<br>Ndabyemera<br>Simbyemera<br>Simbyemera na gato<br>Ntibimureba<br>Yanze gusubiza
C07f	C07f Mu mwaka ushize (kuva kwibazwa riheruka): Umugabo wanjye yantegetse gukoresha uburyo bumwe bwo kuringaniza imbyaro butandukanye n'ubwo njyewe nashakaga gukoresha	1<br>2<br>3<br>4<br>997<br>999	Ndabyemera cyane<br>Ndabyemera<br>Simbyemera<br>Simbyemera na gato<br>Ntibimureba<br>Yanze gusubiza
C07e	C07e Mu mwaka ushize (kuva kwibazwa riheruka): Umugabo wanjye yantitirije gutwita	1<br>2<br>3<br>4<br>997<br>999	Ndabyemera cyane<br>Ndabyemera<br>Simbyemera<br>Simbyemera na gato<br>Ntibimureba<br>Yanze gusubiza
C.10	C.10 Ngiye kugusomera interuro zikurikira. Urajya umbwira niba ibikubiyemo ubyemera cyane, ubyemera, utabyemera, cyangwa utabyemera na gato. Ndamutse nshatse gukoresha uburyo bwo kwirinda gusama umugabo wanjye yambuza	1<br>2<br>3<br>4<br>997<br>999	Ndabyemera cyane<br>Ndabyemera<br>Simbyemera<br>Simbyemera na gato<br>Ntibimureba<br>Yanze gusubiza
survey > PART 4. Imibanire<br>*Group relevant when: ${A01} >=18*			
note4	Iyo abantu babiri bashyingiranwe, ubusanzwe basangira ibyiza n'ibibi. Ngiye kukubaza ibibazo bike byerekeranye n'imibanire yawe n'umugabo wawe. Ibisubizo byawe byose biragirwa ibanga kandi ntawe bizabwirwa n'umwe.		
D01	D01 Ngiye kugusomera interuro zikurikira maze umbwire ibizikubiyemo inshuro bikubaho. Maze unsubize niba bikubaho kenshi, rimwe na rimwe, gake gashoboka, cyangwa nta na rimwe.		
D01a	D01a Iyo mfite ibibazo umugabo wanjye aranyumva	1<br>2<br>3<br>4<br>998	Kenshi<br>Rimwe na rimwe<br>Gake gashoboka<br>Nta na rimwe<br>Simbizi
D01b	D01b Iyo hari ibintu bitagenda neza, umugabo wanjye arabingayira	1<br>2<br>3<br>4<br>998	Kenshi<br>Rimwe na rimwe<br>Gake gashoboka<br>Nta na rimwe<br>Simbizi
D01c	D01c Numva umugabo wanjye anyishimiye	1	Kenshi

		2	Rimwe na rimwe
		3	Gake gashoboka
		4	Nta na rimwe
		998	Simbizi
D01d	D01d Numva nubashywe n'ubwo haba hari ibyo tutumvikanyeho	1	Kenshi
		2	Rimwe na rimwe
		3	Gake gashoboka
		4	Nta na rimwe
		998	Simbizi
D01e	D01e Ibibazo byacu tubikemura neza mu bwumvikane	1	Kenshi
		2	Rimwe na rimwe
		3	Gake gashoboka
		4	Nta na rimwe
		998	Simbizi
D01f	D01f Umugabo wanjye agaya ibitekerezo, amarangamutima n'ibyifuzo byanjye	1	Kenshi
		2	Rimwe na rimwe
		3	Gake gashoboka
		4	Nta na rimwe
		998	Simbizi
D01g	D01g Umugabo wanjye angaragariza urukundo n'ubwuzu	1	Kenshi
		2	Rimwe na rimwe
		3	Gake gashoboka
		4	Nta na rimwe
		998	Simbizi
D02	D02 Ngiye kugusomera interuro zikurikira, urajya umbwira niba ibikubiyemo ubyemera cyane, niba ubyemera, niba ubihakana, niba utabyemera na gato cyangwa se niba ntacyo wabivugaho:		
D02a	D02a Ku mugoroba, njye n'umugabo wanjye tuganira kenshi ku byatubayeho k'umunsi	1	Ndabyemera cyane
		2	Ndabyemera
		3	Ntacyo nabivugaho
		4	Simbyemera
		5	Simbyemera na gato
D04	D04 Ushobora kumbwira inshuro wowe n'umugabo wawe mugirana amakimbirane?<br>*MUSOMERE IBISUBIZO BYOSE BYATANZWE.*	1	Kenshi
		2	Rimwe na rimwe
		3	Gake gashoboka
		4	Nta na rimwe
		999	Yanze gusubiza
D05	D05 Tugiye kuganira ku buryo uganira n'umugabo wawe. Ni kangahe uganira n'umugabo wawe ku mikoreshereze y'amafaranga mwinjiza n'ayo musohora mu rugo?	1	Kenshi
		2	Rimwe na rimwe
		3	Gake gashoboka
		4	Nta na rimwe
		997	Ntibimureba
		999	Yanze gusubiza
D05a	D05a Iyo muganira ku mikoreshereze y'amafaranga mwinjiza n'ayo musohora mu rugo, ubona umugabo wawe abifata gute ese ibitekerezo byawe abiha agaciro?<br>*Question relevant when: ${D05} !='4' and ${D05} !='997'*	1	Kenshi
		2	Rimwe na rimwe
		3	Gake gashoboka
		4	Nta na rimwe
		998	Simbizi
		999	Yanze gusubiza
D05c	D05c Ni nde ufata icyemezo cya nyuma ku bijyanye n'ikoreshwa ry'amafaranga mwinjiza n'ayo musohora mu rugo?<br>*Question relevant when: ${D05} !='997'*	1	Wowe
		2	Umugabo wawe
		3	Mufite uruhare rungana
		4	Undi muntu
		998	Simbizi
		999	Yanze gusubiza
D06	D06 Ni kangahe muganira n'umugabo wawe ku bijyanye no kugura ibintu bitwara amafaranga menshi (urugero: inka, ishyamba, umurima)?	1	Kenshi
		2	Rimwe na rimwe
		3	Gake gashoboka
		4	Nta na rimwe
		997	Ntibimureba
		999	Yanze gusubiza

D06a	D06a Iyo muganira ku bijyanye no kugura ibintu bitwara amafaranga menshi [urugero: inka, ishyamba, umurima] wumva umugabo wawe aha agaciro ibitekerezo byawe?<br>*Question relevant when: ${D06} !='4' and ${D06} !='997'*	1	Kenshi
		2	Rimwe na rimwe
		3	Gake gashoboka
		4	Nta na rimwe
		998	Simbizi
		999	Yanze gusubiza
D06c	D06c Ni nde ufata icyemezo cya nyuma ku bijyanye no kugura ibintu bitwara amafaranga menshi? (urugero: Isambu, umurima, inka…)?<br>*Question relevant when: ${D06} !='997'*	1	Wowe
		2	Umugabo wawe
		3	Mufite uruhare rungana
		4	Undi muntu
		998	Simbizi
		999	Yanze gusubiza
D07	D07 Ni kangahe muganira n'umugabo wawe ku bijyanye n'umubare w'abana mwifuza kubyara n'uko mwabakurikiza?	1	Kenshi
		2	Rimwe na rimwe
		3	Gake gashoboka
		4	Nta na rimwe
		997	Ntibimureba
		999	Yanze gusubiza
D07a	D07a Iyo muganira ku mubare w'abana muzabyara cyangwa uko mwabakurikiza, wumva umugabo wawe aha ibitekerezo byawe agaciro?<br>*Question relevant when: ${D07} !='4' and ${D07} !='997'*	1	Kenshi
		2	Rimwe na rimwe
		3	Gake gashoboka
		4	Nta na rimwe
		998	Simbizi
		999	Yanze gusubiza
D07c	D07c Ni nde ufata icyemezo cya nyuma mu bijyanye n'umubare w'abana mwifuza kubyara cyangwa uko mwabakurikiza?<br>*Question relevant when: ${D07} !='997'*	1	Wowe
		2	Umugabo wawe
		3	Mufite uruhare rungana
		4	Undi muntu
		998	Simbizi
		999	Yanze gusubiza
D08	D08 Ni kangahe muganira n'umugabo wawe niba bishoboka ko wakorera hanze yo mu rugo?	1	Kenshi
		2	Rimwe na rimwe
		3	Gake gashoboka
		4	Nta na rimwe
		997	Ntibimureba
		999	Yanze gusubiza
D08a	D08a Iyo muganira ku bijyanye nuko wakorera ahandi hatari mu rugo, ni kangahe wumva umugabo wawe ahaye ibitekerezo byawe agaciro?<br>*Question relevant when: ${D08} !='4' and ${D08} !='997'*	1	Kenshi
		2	Rimwe na rimwe
		3	Gake gashoboka
		4	Nta na rimwe
		998	Simbizi
		999	Yanze gusubiza
D08c	D08c Ni nde ufata icyemezo cya nyuma ku bijyanye no kuba wajya gukorera ahandi hatari mu rugo?<br>*Question relevant when: ${D08} !='997'*	1	Wowe
		2	Umugabo wawe
		3	Mufite uruhare rungana
		4	Undi muntu
		998	Simbizi
		999	Yanze gusubiza
D09	D09 Ni kangahe muganira n'umugabo wawe ku bijyanye n'imikoreshereze y'amafaranga yinjiza?	1	Kenshi
		2	Rimwe na rimwe
		3	Gake gashoboka
		4	Nta na rimwe
		997	Ntibimureba
		999	Yanze gusubiza
D09a	D09a Iyo muganira ku buryo amafaranga umugabo wawe yinjiza akoreshwa, wumva umugabo wawe aha ibitekerezo byawe agaciro?<br>*Question relevant when: ${D09} !='4' and ${D09} !='997'*	1	Kenshi
		2	Rimwe na rimwe
		3	Gake gashoboka
		4	Nta na rimwe
		998	Simbizi
		999	Yanze gusubiza

D09c	D09c Ni nde ufata icyemezo cya nyuma ku bijyanye n'uburyo amafaranga umugabo wawe yinjiza akoreshwa?<br>*Question relevant when: ${D09} !='997'*	1	Wowe
		2	Umugabo wawe
		3	Mufite uruhare rungana
		4	Undi muntu
		998	Simbizi
		999	Yanze gusubiza
D10	D10 Ni kangahe muganira n'umugabo wawe uburyo amafaranga winjiza akoreshwa?	1	Kenshi
		2	Rimwe na rimwe
		3	Gake gashoboka
		4	Nta na rimwe
		997	Ntibimureba
		999	Yanze gusubiza
D10a	D10a Iyo muganira ku buryo amafaranga winjiza akoreshwa, wumva umugabo wawe aha ibitekerezo byawe agaciro?<br>*Question relevant when: ${D10} !='4' and ${D10} !='997'*	1	Kenshi
		2	Rimwe na rimwe
		3	Gake gashoboka
		4	Nta na rimwe
		998	Simbizi
		999	Yanze gusubiza
D10c	D10c Ni nde ufata icyemezo cya nyuma ku bijyanye n'uburyo amafaranga winjiza akoreshwa?<br>*Question relevant when: ${D10} !='997'*	1	Wowe
		2	Umugabo wawe
		3	Mufite uruhare rungana
		4	Undi muntu
		998	Simbizi
		999	Yanze gusubiza
D11	D11 Ni kangahe muganira n'umugabo wawe niba mwaboneza urubyaro cyangwa uburyo bwo kuboneza urubyaro mukoresha?	1	Kenshi
		2	Rimwe na rimwe
		3	Gake gashoboka
		4	Nta na rimwe
		997	Ntibimureba
		999	Yanze gusubiza
D11a	D11a Iyo muganira kubijyanye no kuboneza urubyaro cyangwa uburyo mwakoreshwa, wumva umugabo wawe aha ibitekerezo byawe agaciro?<br>*Question relevant when: ${D11} !='4' and ${D11} !='997'*	1	Kenshi
		2	Rimwe na rimwe
		3	Gake gashoboka
		4	Nta na rimwe
		998	Simbizi
		999	Yanze gusubiza
D11c	D11c Ni nde ufata icyemezo cya nyuma ku bijyanye no kuboneza urubyaro cyangwa uburyo mwakoreshwa mu kuboneza urubyaro?<br>*Question relevant when: ${D11} !='997'*	1	Wowe
		2	Umugabo wawe
		3	Mufite uruhare rungana
		4	Undi muntu
		998	Simbizi
		999	Yanze gusubiza
D13	D13 Ni kangahe muganira n'umugabo wawe ku bijyanye n'amashuli n'imyigire by'abana?	1	Kenshi
		2	Rimwe na rimwe
		3	Gake gashoboka
		4	Nta na rimwe
		997	Ntibimureba
		999	Yanze gusubiza
D13a	D13a Iyo muganira n'umugabo wawe ku bijyane n'amashuli n'imyigire by'abana, wumva umugabo wawe aha ibitekerezo byawe agaciro?<br>*Question relevant when: ${D13} !='4' and ${D13} !='997'*	1	Kenshi
		2	Rimwe na rimwe
		3	Gake gashoboka
		4	Nta na rimwe
		998	Simbizi
		999	Yanze gusubiza
D13c	D13c Ni nde ufata icyemezo cya nyuma ku bijyanye n'amashuli n'imyigire by'abana?<br>*Question relevant when: ${D13} !='997'*	1	Wowe
		2	Umugabo wawe
		3	Mufite uruhare rungana
		4	Undi muntu
		998	Simbizi

		999 Yanze gusubiza

survey > PART 5. Kwita ku bandi

Group relevant when: ${A01} >=18

note5	Ngiye kukubaza ibibazo bike birebana n'umuryango wawe n'imirimo yo mu rugo.	
note_512	Ubu ngiye kukubaza ukuntu wowe n'umugabo wawe mugabana imirimo itandukanye yo kwita ku mwana.<br>*ICYITONDERWA: Ibibazo bikurikira birabaza uko umugore n'umugabo we basaranganya imirimo yo kwita ku mwana. Kubibazo bimwe na bimwe nkurugero, urazagusanga umwana akiri muto kuba jajya kwishuri, cyangwa hari ibikorwa byabajijwe bikaba bikorwa n'umukozi wo murugo cyangwa umuvandimwe. Kubibazo nkibyo, andika 'Ntibimureba'*<br>*Question relevant when: ${A10} !='0'*	
E.12	E.12 Ukuyemo ubufasha bw'abantu baba hanze y'urugo rwanyu, ni gute wowe n'umugabo wawe musaranganya ibi bikorwa? Kwita ku mwana cyangwa abana buri munsi<br>*Question relevant when: ${A10} !='0'*	1 Burigihe ni wowe<br>2 Kenshi ni wowe<br>3 Turasaranganya cyangwa tubikorera hamwe<br>4 Kenshi ni uwomwashakanye<br>5 Burigihe ni uwomwashakanye<br>997 Ntibimureba
E.12a	E.12a Mu minsi 7 ishize, wakoze kino gikorwa iminsi ingahe?<br>*[Andika umubare w'iminsi]*<br>*Question relevant when: ${A10} !='0' and ${E.12} !='997'*<br>*Response constrained to: .<=7*	
E.12b	E.12b Ku munsi usanzwe, iyo wakoze kino gikorwa, ukoresha amasaha angahe?<br>*Gerageza wandi ighe mu icyikubo cya 15 (Urugero: niba umuntu avuze ko yakoresheje iminota 15 ku murimo andiko 0.25. burungushura ugeze ku minota 15, burungushura indi mibare yose kuza ku gikubo cya 15 kiri hafi aho.(urugero: niba ari iminota 35 uburungushure ushyire ku minota 30, hanyuma wandike amasaha 0.5) [Andika umubare w'amasaha]*<br>*Question relevant when: ${A10} !='0' and ${E.12} !='997' and ${E.12a} !='0'*<br>*Response constrained to: .<=24 and .>0*	
E.13	E.13 Ukuyemo ubufasha bw'abantu baba hanze y'urugo rwanyu, ni gute wowe n'umugabo wawe musaranganya ibi bikorwa? Guterura uruhinja rukimara kuvuka<br>*Question relevant when: ${A10} !='0'*	1 Burigihe ni wowe<br>2 Kenshi ni wowe<br>3 Turasaranganya cyangwa tubikorera hamwe<br>4 Kenshi ni uwomwashakanye<br>5 Burigihe ni uwomwashakanye<br>997 Ntibimureba
E.13a	E.13a Mu minsi 7 ishize, wakoze kino gikorwa iminsi ingahe?<br>*[Andika umubare w'iminsi]*<br>*Question relevant when: ${A10} !='0' and ${E.13} !='997'*<br>*Response constrained to: .<=7*	
E.13b	E.13b Ku munsi usanzwe, iyo wakoze kino gikorwa, ukoresha amasaha angahe?<br>*Gerageza wandi ighe mu icyikubo cya 15 (Urugero: niba umuntu avuze ko yakoresheje iminota 15 ku murimo andiko 0.25. burungushura ugeze ku minota 15, burungushura indi mibare yose kuza ku gikubo cya 15 kiri hafi aho.(urugero: niba ari iminota 35 uburungushure ushyire ku minota 30, hanyuma wandike amasaha 0.5) [Andika umubare w'amasaha]*<br>*Question relevant when: ${A10} !='0' and ${E.13} !='997' and ${E.13a} !='0'*<br>*Response constrained to: .<=24 and .>0*	
E.14	E.14 Ukuyemo ubufasha bw'abantu baba hanze y'urugo rwanyu, ni gute wowe n'umugabo wawe musaranganya ibi bikorwa? Kugaburira umwana<br>*Question relevant when: ${A10} !='0'*	1 Burigihe ni wowe<br>2 Kenshi ni wowe<br>3 Turasaranganya cyangwa tubikorera hamwe<br>4 Kenshi ni uwomwashakanye<br>5 Burigihe ni uwomwashakanye<br>997 Ntibimureba
E.14a	E.14a Mu minsi 7 ishize, wakoze kino gikorwa iminsi ingahe?<br>*[Andika umubare w'iminsi]*<br>*Question relevant when: ${A10} !='0' and ${E.14} !='997'*<br>*Response constrained to: .<=7*	
E.14b	E.14b Ku munsi usanzwe, iyo wakoze kino gikorwa, ukoresha amasaha angahe?<br>*Gerageza wandi ighe mu icyikubo cya 15 (Urugero: niba umuntu avuze ko yakoresheje iminota 15 ku murimo andiko 0.25. burungushura ugeze ku minota 15, burungushura indi mibare yose kuza ku gikubo cya 15 kiri hafi aho.(urugero: niba ari iminota 35 uburungushure ushyire ku minota 30, hanyuma wandike amasaha 0.5) [Andika umubare w'amasaha]*<br>*Question relevant when: ${A10} !='0' and ${E.14} !='997' and ${E.14a} !='0'*<br>*Response constrained to: .<=24 and .>0*	
E.15	E.15 Ukuyemo ubufasha bw'abantu baba hanze y'urugo rwanyu, ni gute wowe n'umugabo wawe musaranganya ibi bikorwa? Kwuhagira umwana<br>*Question relevant when: ${A10} !='0'*	1 Burigihe ni wowe<br>2 Kenshi ni wowe<br>3 Turasaranganya cyangwa

			tubikorera hamwe
		4	Kenshi ni uwomwashakanye
		5	Burigihe ni uwomwashakanye
		997	Ntibimureba
E.15a	E.15a Mu minsi 7 ishize, wakoze kino gikorwa iminsi ingahe?<br>*[Andika umubare w'iminsi]*<br>*Question relevant when: ${A10} !='0' and ${E.15} !='997'*<br>*Response constrained to: .<=7*		
E.15b	E.15b Ku munsi usanzwe, iyo wakoze kino gikorwa, ukoresha amasaha angahe?<br>*Gerageza wandi ighe mu icyikubo cya 15 (Urugero: niba umuntu avuze ko yakoresheje iminota 15 ku murimo andiko 0.25. burungushura ugeze ku minota 15, burungushura indi mibare yose kuza ku gikubo cya 15 kiri hafi aho.(urugero: niba ari iminota 35 uburungushure ushyire ku minota 30, hanyuma wandike amasaha 0.5) [Andika umubare w'amasaha]*<br>*Question relevant when: ${A10} !='0' and ${E.15} !='997' and ${E.15a} !='0'*<br>*Response constrained to: .<=24 and .>0*		
E.16	E.16 Ukuyemo ubufasha bw'abantu baba hanze y'urugo rwanyu, ni gute wowe n'umugabo wawe musaranganya ibi bikorwa? Guhoza umwana igihe arize cyangwa yarakaye<br>*Question relevant when: ${A10} !='0'*	1	Burigihe ni wowe
		2	Kenshi ni wowe
		3	Turasaranganya cyangwa tubikorera hamwe
		4	Kenshi ni uwomwashakanye
		5	Burigihe ni uwomwashakanye
		997	Ntibimureba
E.16a	E.16a Mu minsi 7 ishize, wakoze kino gikorwa iminsi ingahe?<br>*[Andika umubare w'iminsi]*<br>*Question relevant when: ${A10} !='0' and ${E.16} !='997'*<br>*Response constrained to: .<=7*		
E.16b	E.16b Ku munsi usanzwe, iyo wakoze kino gikorwa, ukoresha amasaha angahe?<br>*Gerageza wandi ighe mu icyikubo cya 15 (Urugero: niba umuntu avuze ko yakoresheje iminota 15 ku murimo andiko 0.25. burungushura ugeze ku minota 15, burungushura indi mibare yose kuza ku gikubo cya 15 kiri hafi aho.(urugero: niba ari iminota 35 uburungushure ushyire ku minota 30, hanyuma wandike amasaha 0.5) [Andika umubare w'amasaha]*<br>*Question relevant when: ${A10} !='0' and ${E.16} !='997' and ${E.16a} !='0'*<br>*Response constrained to: .<=24 and .>0*		
E.171	E.171 Ukuyemo ubufasha bw'abantu baba hanze y'urugo rwanyu, ni gute wowe n'umugabo wawe musaranganya ibi bikorwa? Kujyana umwana ku kigo nderabuzima mu gihe arwaye cyangwa kumukingiza<br>*Question relevant when: ${A10} !='0'*	1	Burigihe ni wowe
		2	Kenshi ni wowe
		3	Turasaranganya cyangwa tubikorera hamwe
		4	Kenshi ni uwomwashakanye
		5	Burigihe ni uwomwashakanye
		997	Ntibimureba
E.171a	E.161a Mu minsi 7 ishize, wakoze kino gikorwa iminsi ingahe?<br>*[Andika umubare w'iminsi]*<br>*Question relevant when: ${A10} !='0' and ${E.171} !='997'*<br>*Response constrained to: .<=7*		
E.17	E.17 Ukuyemo ubufasha bw'abantu baba hanze y'urugo rwanyu, ni gute wowe n'umugabo wawe musaranganya ibi bikorwa? Kujyana no kuvana umwana/ abana ku ishuri<br>*Question relevant when: ${A10} !='0'*	1	Burigihe ni wowe
		2	Kenshi ni wowe
		3	Turasaranganya cyangwa tubikorera hamwe
		4	Kenshi ni uwomwashakanye
		5	Burigihe ni uwomwashakanye
		997	Ntibimureba
E.17a	E.17a Mu minsi 7 ishize, wakoze kino gikorwa iminsi ingahe?<br>*[Andika umubare w'iminsi]*<br>*Question relevant when: ${A10} !='0' and ${E.17} !='997'*<br>*Response constrained to: .<=7*		
E.17b	E.17b Ku munsi usanzwe, iyo wakoze kino gikorwa, ukoresha amasaha angahe?<br>*Gerageza wandi ighe mu icyikubo cya 15 (Urugero: niba umuntu avuze ko yakoresheje iminota 15 ku murimo andiko 0.25. burungushura ugeze ku minota 15, burungushura indi mibare yose kuza ku gikubo cya 15 kiri hafi aho.(urugero: niba ari iminota 35 uburungushure ushyire ku minota 30, hanyuma wandike amasaha 0.5) [Andika umubare w'amasaha]*<br>*Question relevant when: ${A10} !='0' and ${E.17} !='997' and ${E.17a} !='0'*<br>*Response constrained to: .<=24 and .>0*		
E.18	E.18 Ukuyemo ubufasha bw'abantu baba hanze y'urugo rwanyu, ni gute wowe n'umugabo wawe musaranganya ibi bikorwa? Gucira umwana/abana imigani, kumuririmbira no kumukinisha<br>*Question relevant when: ${A10} !='0'*	1	Burigihe ni wowe
		2	Kenshi ni wowe

		3 Turasaranganya cyangwa tubikorera hamwe<br>4 Kenshi ni uwomwashakanye<br>5 Burigihe ni uwomwashakanye<br>997 Ntibimureba
E.18a	E.18a Mu minsi 7 ishize, wakoze kino gikorwa iminsi ingahe?<br>*[Andika umubare w'iminsi]*<br>*Question relevant when: ${A10} !='0' and ${E.18} !='997'*<br>*Response constrained to: .<=7*	
E.18b	E.18b Ku munsi usanzwe, iyo wakoze kino gikorwa, ukoresha amasaha angahe?<br>*Gerageza wandi ighe mu icyikubo cya 15 (Urugero: niba umuntu avuze ko yakoresheje iminota 15 ku murimo andiko 0.25. burungushura ugeze ku minota 15, burungushura indi mibare yose kuza ku gikubo cya 15 kiri hafi aho.(urugero: niba ari iminota 35 uburungushure ushyire ku minota 30, hanyuma wandike amasaha 0.5) [Andika umubare w'amasaha]*<br>*Question relevant when: ${A10} !='0' and ${E.18} !='997' and ${E.18a} !='0'*<br>*Response constrained to: .<=24 and .>0*	
E.19	E.19 Ukuyemo ubufasha bw'abantu baba hanze y'urugo rwanyu, ni gute wowe n'umugabo wawe musaranganya ibi bikorwa? Kwigisha umwana/ abana ikintu icyo aricyo cyose.<br>*Question relevant when: ${A10} !='0'*	1 Burigihe ni wowe<br>2 Kenshi ni wowe<br>3 Turasaranganya cyangwa tubikorera hamwe<br>4 Kenshi ni uwomwashakanye<br>5 Burigihe ni uwomwashakanye<br>997 Ntibimureba
E.19a	E.19a Mu minsi 7 ishize, wakoze kino gikorwa iminsi ingahe?<br>*[Andika umubare w'iminsi]*<br>*Question relevant when: ${A10} !='0' and ${E.19} !='997'*<br>*Response constrained to: .<=7*	
E.19b	E.19b Ku munsi usanzwe, iyo wakoze kino gikorwa, ukoresha amasaha angahe?<br>*Gerageza wandi ighe mu icyikubo cya 15 (Urugero: niba umuntu avuze ko yakoresheje iminota 15 ku murimo andiko 0.25. burungushura ugeze ku minota 15, burungushura indi mibare yose kuza ku gikubo cya 15 kiri hafi aho.(urugero: niba ari iminota 35 uburungushure ushyire ku minota 30, hanyuma wandike amasaha 0.5) [Andika umubare w'amasaha]*<br>*Question relevant when: ${A10} !='0' and ${E.19} !='997' and ${E.19a} !='0'*<br>*Response constrained to: .<=24 and .>0*	
E.20	E.20 Ukuyemo ubufasha bw'abantu baba hanze y'urugo rwanyu, ni gute wowe n'umugabo wawe musaranganya ibi bikorwa? Gucyaha umwana/ abana<br>*Question relevant when: ${A10} !='0'*	1 Burigihe ni wowe<br>2 Kenshi ni wowe<br>3 Turasaranganya cyangwa tubikorera hamwe<br>4 Kenshi ni uwomwashakanye<br>5 Burigihe ni uwomwashakanye<br>997 Ntibimureba
E.20a	E.20a Mu minsi 7 ishize, wakoze kino gikorwa iminsi ingahe?<br>*[Andika umubare w'iminsi]*<br>*Question relevant when: ${A10} !='0' and ${E.20} !='997'*<br>*Response constrained to: .<=7*	
E.21	E.21 Ukuyemo ubufasha bw'abantu baba hanze y'urugo rwanyu, ni gute wowe n'umugabo wawe musaranganya ibi bikorwa? Kunyuza akanyafu ku mwana/ abana<br>*Question relevant when: ${A10} !='0'*	1 Burigihe ni wowe<br>2 Kenshi ni wowe<br>3 Turasaranganya cyangwa tubikorera hamwe<br>4 Kenshi ni uwomwashakanye<br>5 Burigihe ni uwomwashakanye<br>997 Ntibimureba
E.21a	E.21a Mu minsi 7 ishize, wakoze kino gikorwa iminsi ingahe?<br>*[Andika umubare w'iminsi]*<br>*Question relevant when: ${A10} !='0' and ${E.21} !='997'*<br>*Response constrained to: .<=7*	
E04	E04 Wifuza ko umugabo wawe agira uruhare rungana iki mu kwita ku mwana wanyu?<br>*MUSOMERE IBISUBIZO BYOSE BYATANZWE*<br>*Question relevant when: ${A10} !='0'*	1 Nishimiye uruhare rwe<br>2 Nakwifuje yuko yongera uruhare rwe<br>3 Nifuza ko yagabanya uruhare rwe<br>998 Simbizi

E06	E06 Ngiye kukubaza ibizazo bike birebana no kurera abana. Ababyeyi bakoresha uburyo runaka mu kwigisha abana babo uko bitwara neza no kubakosora igihe bitwaye nabi. Ndasoma bumwe mu buryo ababyeyi bakoresha. Umbwire niba warigeze ukoresha bumwe muri ubu buryo mu ukwezi gushize kuri umwe mu bana wabyaye.<br>*Question relevant when: ${A10} !='0'*	
E06a	E06a Wamuvanye k'ubutoni, wabujijie umwana wawe ikintu yakoze cyangwa ntiwawemereye umwana wawe gusohoka mu rugo<br>*Iki kibazo kirabaza ku byabaye mu gihe cy'ukwezi gushize(Iminsi 30 ishize) kandi bikareba gusa abana bawe wibyariye*<br>*Question relevant when: ${A10} !='0'*	0 Oya<br>1 Yego
E06b	E06b Wasobanuriye umwana wawe buryo ki imyitwarire ye itari ikwiye<br>*Iki kibazo kirabaza ku byabaye mu gihe cy'ukwezi gushize(Iminsi 30 ishize) kandi bikareba gusa abana bawe wibyariye*<br>*Question relevant when: ${A10} !='0'*	0 Oya<br>1 Yego
E06c	E06c Watigishije umwana wawe<br>*Iki kibazo kirabaza ku byabaye mu gihe cy'ukwezi gushize(Iminsi 30 ishize) kandi bikareba gusa abana bawe wibyariye*<br>*Question relevant when: ${A10} !='0'*	0 Oya<br>1 Yego
E06d	E06d Watonganyije umwana wawe uvuga cyane<br>*Iki kibazo kirabaza ku byabaye mu gihe cy'ukwezi gushize(Iminsi 30 ishize) kandi bikareba gusa abana bawe wibyariye*<br>*Question relevant when: ${A10} !='0'*	0 Oya<br>1 Yego
E06e	E06e Washakiye umwana wawe ikintu cyo gukora<br>*Iki kibazo kirabaza ku byabaye mu gihe cy'ukwezi gushize(Iminsi 30 ishize) kandi bikareba gusa abana bawe wibyariye*<br>*Question relevant when: ${A10} !='0'*	0 Oya<br>1 Yego
E06f	E06f Wakubise umwana urushyi ku kibuno<br>*Iki kibazo kirabaza ku byabaye mu gihe cy'ukwezi gushize(Iminsi 30 ishize) kandi bikareba gusa abana bawe wibyariye*<br>*Question relevant when: ${A10} !='0'*	0 Oya<br>1 Yego
E06g	E06g Wakubise umwana wawe umukandara, inkoni cyangwa ikindi kintu kibabaza ku kibuno cyangwa ahandi ku mubiri<br>*Iki kibazo kirabaza ku byabaye mu gihe cy'ukwezi gushize(Iminsi 30 ishize) kandi bikareba gusa abana bawe wibyariye*<br>*Question relevant when: ${A10} !='0'*	0 Oya<br>1 Yego
E06h	E06h Wise umwana wawe igicucu, umunebwe cyangwa irindi zina nk'iryo<br>*Iki kibazo kirabaza ku byabaye mu gihe cy'ukwezi gushize(Iminsi 30 ishize) kandi bikareba gusa abana bawe wibyariye*<br>*Question relevant when: ${A10} !='0'*	0 Oya<br>1 Yego
E06i	E06i Wakubise urushyi umwana wawe mu maso, mu mutwe cyangwa ku matwi<br>*Iki kibazo kirabaza ku byabaye mu gihe cy'ukwezi gushize(Iminsi 30 ishize) kandi bikareba gusa abana bawe wibyariye*<br>*Question relevant when: ${A10} !='0'*	0 Oya<br>1 Yego
E06j	E06j Wakubise inshyi umwana wawe ku biganza, amaboko cyangwa amaguru<br>*Iki kibazo kirabaza ku byabaye mu gihe cy'ukwezi gushize(Iminsi 30 ishize) kandi bikareba gusa abana bawe wibyariye*<br>*Question relevant when: ${A10} !='0'*	0 Oya<br>1 Yego
E06k	E06k Wakubise umwana wawe uko wari ushoboye kose<br>*Iki kibazo kirabaza ku byabaye mu gihe cy'ukwezi gushize(Iminsi 30 ishize) kandi bikareba gusa abana bawe wibyariye*<br>*Question relevant when: ${A10} !='0'*	0 Oya<br>1 Yego
E06l	E06l Wapfukamishije umwana wawe<br>*Iki kibazo kirabaza ku byabaye mu gihe cy'ukwezi gushize(Iminsi 30 ishize) kandi bikareba gusa abana bawe wibyariye*<br>*Question relevant when: ${A10} !='0'*	0 Oya<br>1 Yego
E06m	E06m Wikoreje umwana wawe amatafari, amabuye, mu biganza yicaye, ahagaze cyangwa apfukamye<br>*Iki kibazo kirabaza ku byabaye mu gihe cy'ukwezi gushize(Iminsi 30 ishize) kandi bikareba gusa abana bawe wibyariye*<br>*Question relevant when: ${A10} !='0'*	0 Oya<br>1 Yego
E07	E07 Ngiye kugusomera interuro hanyuma umbwire niba ibikubiyemo ubyemera cyane, niba ubyemera, niba ntacyo wabivugaho, niba utabyemerea cyangwa se niba utabyemera na gato.<br>*Question relevant when: ${A10} !='0'*	
E07a	E07a Kugirango abana barerwe bikwiye, bakeneye guhabwa ibihano byo ku mubiri<br>*Question relevant when: ${A10} !='0'*	1 Ndabyemera cyane<br>2 Ndabyemera<br>3 Ntacyo nabivugaho<br>4 Simbyemera<br>5 Simbyemera na gato
E07b	E07b Ibihano byo ku mubiri bigira ingaruka mbi ku bana<br>*Question relevant when: ${A10} !='0'*	1 Ndabyemera cyane<br>2 Ndabyemera<br>3 Ntacyo nabivugaho<br>4 Simbyemera<br>5 Simbyemera na gato
note_E22	Ubu ngiye kukubaza ukuntu wowe n'umugore wawe mugabana imirimo itandukanye yo murugo.<br>*ICYITONDERWA: Ibibazo bikurikira birabaza uko umugore n'umugabo we basaranganya imirimo yo murugo. Kubibazo bimwe na bimwe urazagusanga igikorwa kitareba uwo ariwe wese murugo, cyangwa gikaba gokorwa n'undi muntu wo murugo (urugero umukozi wo murugo cyangwa umuvandimwe). Kubibazo nkibyo, andika 'Ntibimureba'*	
E.22	E.22 Ukuyemo ubufasha bw'abantu baba hanze y'urugo rwanyu, ni gute wowe n'umugabo wawe musaranganya ibi bikorwa? Kuvoma amazi yo gukoresha murugo (ubariyemo igihe gikoreshwa kujyayo no kugaruka)	1 Burigihe ni wowe<br>2 Kenshi ni wowe<br>3 Turasaranganya cyangwa tubikorera hamwe<br>4 Kenshi ni uwomwashakanye<br>5 Burigihe ni

		uwomwashakanye<br>997 Ntibimureba
E.22a	E.22a Mu minsi 7 ishize, wakoze kino gikorwa iminsi ingahe?<br>*[Andika umubare w'iminsi]*<br>*Question relevant when: ${E.22} !='997'*<br>*Response constrained to: .<=7*	
E.22b	E.22b Ku munsi usanzwe, iyo wakoze kino gikorwa, ukoresha amasaha angahe?<br>*Gerageza wandi ighe mu icyikubo cya 15 (Urugero: niba umuntu avuze ko yakoresheje iminota 15 ku murimo andiko 0.25. burungushura ugeze ku minota 15, burungushura indi mibare yose kuza ku gikubo cya 15 kiri hafi aho.(urugero: niba ari iminota 35 uburungushure ushyire ku minota 30, hanyuma wandike amasaha 0.5) [Andika umubare w'amasaha]*<br>*Question relevant when: ${E.22} !='997' and ${E.22a} !='0'*<br>*Response constrained to: .<=24 and .>0*	
E.23	E.23 Ukuyemo ubufasha bw'abantu baba hanze y'urugo rwanyu, ni gute wowe n'umugabo wawe musaranganya ibi bikorwa? Kumesa imyenda	1 Burigihe ni wowe<br>2 Kenshi ni wowe<br>3 Turasaranganya cyangwa tubikorera hamwe<br>4 Kenshi ni uwomwashakanye<br>5 Burigihe ni uwomwashakanye<br>997 Ntibimureba
E.23a	E.23a Mu minsi 7 ishize, wakoze kino gikorwa iminsi ingahe?<br>*[Andika umubare w'iminsi]*<br>*Question relevant when: ${E.23} !='997'*<br>*Response constrained to: .<=7*	
E.23b	E.23b Ku munsi usanzwe, iyo wakoze kino gikorwa, ukoresha amasaha angahe?<br>*Gerageza wandi ighe mu icyikubo cya 15 (Urugero: niba umuntu avuze ko yakoresheje iminota 15 ku murimo andiko 0.25. burungushura ugeze ku minota 15, burungushura indi mibare yose kuza ku gikubo cya 15 kiri hafi aho.(urugero: niba ari iminota 35 uburungushure ushyire ku minota 30, hanyuma wandike amasaha 0.5) [Andika umubare w'amasaha]*<br>*Question relevant when: ${E.23} !='997' and ${E.23a} !='0'*<br>*Response constrained to: .<=24 and .>0*	
E.24	E.24 Ukuyemo ubufasha bw'abantu baba hanze y'urugo rwanyu, ni gute wowe n'umugabo wawe musaranganya ibi bikorwa? Kujya kwisoko guhahira urugo	1 Burigihe ni wowe<br>2 Kenshi ni wowe<br>3 Turasaranganya cyangwa tubikorera hamwe<br>4 Kenshi ni uwomwashakanye<br>5 Burigihe ni uwomwashakanye<br>997 Ntibimureba
E.24a	E.24a Mu minsi 7 ishize, wakoze kino gikorwa iminsi ingahe?<br>*[Andika umubare w'iminsi]*<br>*Question relevant when: ${E.24} !='997'*<br>*Response constrained to: .<=7*	
E.24b	E.24b Ku munsi usanzwe, iyo wakoze kino gikorwa, ukoresha amasaha angahe?<br>*Gerageza wandi ighe mu icyikubo cya 15 (Urugero: niba umuntu avuze ko yakoresheje iminota 15 ku murimo andiko 0.25. burungushura ugeze ku minota 15, burungushura indi mibare yose kuza ku gikubo cya 15 kiri hafi aho.(urugero: niba ari iminota 35 uburungushure ushyire ku minota 30, hanyuma wandike amasaha 0.5) [Andika umubare w'amasaha]*<br>*Question relevant when: ${E.24} !='997' and ${E.24a} !='0'*<br>*Response constrained to: .<=24 and .>0*	
E.25	E.25 Ukuyemo ubufasha bw'abantu baba hanze y'urugo rwanyu, ni gute wowe n'umugabo wawe musaranganya ibi bikorwa? Gusukura munzu no hanze yayo	1 Burigihe ni wowe<br>2 Kenshi ni wowe<br>3 Turasaranganya cyangwa tubikorera hamwe<br>4 Kenshi ni uwomwashakanye<br>5 Burigihe ni uwomwashakanye<br>997 Ntibimureba
E.25a	E.25a Mu minsi 7 ishize, wakoze kino gikorwa iminsi ingahe?<br>*[Andika umubare w'iminsi]*<br>*Question relevant when: ${E.25} !='997'*<br>*Response constrained to: .<=7*	
E.25b	E.25b Ku munsi usanzwe, iyo wakoze kino gikorwa, ukoresha amasaha angahe?<br>*Gerageza wandi ighe mu icyikubo cya 15 (Urugero: niba umuntu avuze ko yakoresheje iminota 15 ku murimo andiko 0.25. burungushura ugeze ku minota 15, burungushura indi mibare yose kuza ku gikubo cya 15 kiri hafi aho.(urugero: niba ari iminota 35 uburungushure ushyire ku minota 30, hanyuma wandike amasaha 0.5) [Andika umubare w'amasaha]*<br>*Question relevant when: ${E.25} !='997' and ${E.25a} !='0'*<br>*Response constrained to: .<=24 and .>0*	
E.26	E.26 Ukuyemo ubufasha bw'abantu baba hanze y'urugo rwanyu, ni gute wowe n'umugabo wawe musaranganya ibi	1 Burigihe ni wowe

	bikorwa? Gusukurura ubwiyuhagiriro n'ubwiherero	2 Kenshi ni wowe<br>3 Turasaranganya cyangwa tubikorera hamwe<br>4 Kenshi ni uwomwashakanye<br>5 Burigihe ni uwomwashakanye<br>997 Ntibimureba
E.26a	E.26a Mu minsi 7 ishize, wakoze kino gikorwa iminsi ingahe?<br>*[Andika umubare w'iminsi]*<br>*Question relevant when: ${E.26} !='997'*<br>*Response constrained to: .<=7*	
E.26b	E.26b Ku munsi usanzwe, iyo wakoze kino gikorwa, ukoresha amasaha angahe?<br>*Gerageza wandi ighe mu icyikubo cya 15 (Urugero: niba umuntu avuze ko yakoresheje iminota 15 ku murimo andiko 0.25. burungushura ugeze ku minota 15, burungushura indi mibare yose kuza ku gikubo cya 15 kiri hafi aho.(urugero: niba ari iminota 35 uburungushure ushyire ku minota 30, hanyuma wandike amasaha 0.5) [Andika umubare w'amasaha]*<br>*Question relevant when: ${E.26} !='997' and ${E.26a} !='0'*<br>*Response constrained to: .<=24 and .>0*	
E.27	E.27 Ukuyemo ubufasha bw'abantu baba hanze y'urugo rwanyu, ni gute wowe n'umugabo wawe musaranganya ibi bikorwa? Gutekera urugo	1 Burigihe ni wowe<br>2 Kenshi ni wowe<br>3 Turasaranganya cyangwa tubikorera hamwe<br>4 Kenshi ni uwomwashakanye<br>5 Burigihe ni uwomwashakanye<br>997 Ntibimureba
E.27a	E.27a Mu minsi 7 ishize, wakoze kino gikorwa iminsi ingahe?<br>*[Andika umubare w'iminsi]*<br>*Question relevant when: ${E.27} !='997'*<br>*Response constrained to: .<=7*	
E.27b	E.27b Ku munsi usanzwe, iyo wakoze kino gikorwa, ukoresha amasaha angahe?<br>*Gerageza wandi ighe mu icyikubo cya 15 (Urugero: niba umuntu avuze ko yakoresheje iminota 15 ku murimo andiko 0.25. burungushura ugeze ku minota 15, burungushura indi mibare yose kuza ku gikubo cya 15 kiri hafi aho.(urugero: niba ari iminota 35 uburungushure ushyire ku minota 30, hanyuma wandike amasaha 0.5) [Andika umubare w'amasaha]*<br>*Question relevant when: ${E.27} !='997' and ${E.27a} !='0'*<br>*Response constrained to: .<=24 and .>0*	
E.28	E.28 Ukuyemo ubufasha bw'abantu baba hanze y'urugo rwanyu, ni gute wowe n'umugabo wawe musaranganya ibi bikorwa? Gucunga amafaranga yinjira n'asohoka buri cyumweru	1 Burigihe ni wowe<br>2 Kenshi ni wowe<br>3 Turasaranganya cyangwa tubikorera hamwe<br>4 Kenshi ni uwomwashakanye<br>5 Burigihe ni uwomwashakanye<br>997 Ntibimureba
E.28a	E.28a Mu minsi 7 ishize, wakoze kino gikorwa iminsi ingahe?<br>*[Andika umubare w'iminsi]*<br>*Question relevant when: ${E.28} !='997'*<br>*Response constrained to: .<=7*	
E.28b	E.28b Ku munsi usanzwe, iyo wakoze kino gikorwa, ukoresha amasaha angahe?<br>*Gerageza wandi ighe mu icyikubo cya 15 (Urugero: niba umuntu avuze ko yakoresheje iminota 15 ku murimo andiko 0.25. burungushura ugeze ku minota 15, burungushura indi mibare yose kuza ku gikubo cya 15 kiri hafi aho.(urugero: niba ari iminota 35 uburungushure ushyire ku minota 30, hanyuma wandike amasaha 0.5) [Andika umubare w'amasaha]*<br>*Question relevant when: ${E.28} !='997' and ${E.28a} !='0'*<br>*Response constrained to: .<=24 and .>0*	
E.29	E.29 Ukuyemo ubufasha bw'abantu baba hanze y'urugo rwanyu, ni gute wowe n'umugabo wawe musaranganya ibi bikorwa? Gutashya inkwi	1 Burigihe ni wowe<br>2 Kenshi ni wowe<br>3 Turasaranganya cyangwa tubikorera hamwe<br>4 Kenshi ni uwomwashakanye<br>5 Burigihe ni uwomwashakanye<br>997 Ntibimureba
E.29a	E.29a Mu minsi 7 ishize, wakoze kino gikorwa iminsi ingahe?<br>*[Andika umubare w'iminsi]*<br>*Question relevant when: ${E.29} !='997'*<br>*Response constrained to: .<=7*	

E.29b	E.29b Ku munsi usanzwe, iyo wakoze kino gikorwa, ukoresha amasaha angahe?<br>*Gerageza wandi ighe mu icyikubo cya 15 (Urugero: niba umuntu avuze ko yakoresheje iminota 15 ku murimo andiko 0.25. burungushura ugeze ku minota 15, burungushura indi mibare yose kuza ku gikubo cya 15 kiri hafi aho.(urugero: niba ari iminota 35 uburungushure ushyire ku minota 30, hanyuma wandike amasaha 0.5) [Andika umubare w'amasaha]*<br>*Question relevant when: ${E.29} !='997' and ${E.29a} !='0'*<br>*Response constrained to: .<=24 and .>0*	
E.30	E.30 Ukuyemo ubufasha bw'abantu baba hanze y'urugo rwanyu, ni gute wowe n'umugabo wawe musaranganya ibi bikorwa? Gushaka ibiryo by'amatungo cyangwa kuragira amatungo yo murugo?	1 Burigihe ni wowe<br>2 Kenshi ni wowe<br>3 Turasaranganya cyangwa tubikorera hamwe<br>4 Kenshi ni uwomwashakanye<br>5 Burigihe ni uwomwashakanye<br>997 Ntibimureba
E.30a	E.30a Mu minsi 7 ishize, wakoze kino gikorwa iminsi ingahe?<br>*[Andika umubare w'iminsi]*<br>*Question relevant when: ${E.30} !='997'*<br>*Response constrained to: .<=7*	
E.30b	E.30b Ku munsi usanzwe, iyo wakoze kino gikorwa, ukoresha amasaha angahe?<br>*Gerageza wandi ighe mu icyikubo cya 15 (Urugero: niba umuntu avuze ko yakoresheje iminota 15 ku murimo andiko 0.25. burungushura ugeze ku minota 15, burungushura indi mibare yose kuza ku gikubo cya 15 kiri hafi aho.(urugero: niba ari iminota 35 uburungushure ushyire ku minota 30, hanyuma wandike amasaha 0.5) [Andika umubare w'amasaha]*<br>*Question relevant when: ${E.30} !='997' and ${E.30a} !='0'*<br>*Response constrained to: .<=24 and .>0*	
E.31	E.31 Ukuyemo ubufasha bw'abantu baba hanze y'urugo rwanyu, ni gute wowe n'umugabo wawe musaranganya ibi bikorwa: Gukora mu buhinzi-bworozi akorera amafaranga cyangwa ikindi gihembo?	1 Burigihe ni wowe<br>2 Kenshi ni wowe<br>3 Turasaranganya cyangwa tubikorera hamwe<br>4 Kenshi ni uwomwashakanye<br>5 Burigihe ni uwomwashakanye<br>997 Ntibimureba
E.31a	E.31a Mu minsi 7 ishize, wakoze kino gikorwa iminsi ingahe?<br>*[Andika umubare w'iminsi]*<br>*Question relevant when: ${E.31} !='997'*<br>*Response constrained to: .<=7*	
E.31b	E.31b Ku munsi usanzwe, iyo wakoze kino gikorwa, ukoresha amasaha angahe?<br>*Gerageza wandi ighe mu icyikubo cya 15 (Urugero: niba umuntu avuze ko yakoresheje iminota 15 ku murimo andiko 0.25. burungushura ugeze ku minota 15, burungushura indi mibare yose kuza ku gikubo cya 15 kiri hafi aho.(urugero: niba ari iminota 35 uburungushure ushyire ku minota 30, hanyuma wandike amasaha 0.5) [Andika umubare w'amasaha]*<br>*Question relevant when: ${E.31} !='997' and ${E.31a} !='0'*<br>*Response constrained to: .<=24 and .>0*	
E.32	E.32 Ukuyemo ubufasha bw'abantu baba hanze y'urugo rwanyu, ni gute wowe n'umugabo wawe musaranganya ibi bikorwa? Gukora mu buhinzi-bworozi nta amafaranga cyangwa ikindi gihembo akorera?	1 Burigihe ni wowe<br>2 Kenshi ni wowe<br>3 Turasaranganya cyangwa tubikorera hamwe<br>4 Kenshi ni uwomwashakanye<br>5 Burigihe ni uwomwashakanye<br>997 Ntibimureba
E.32a	E.32a Mu minsi 7 ishize, wakoze kino gikorwa iminsi ingahe?<br>*[Andika umubare w'iminsi]*<br>*Question relevant when: ${E.32} !='997'*<br>*Response constrained to: .<=7*	
E.32b	E.32b Ku munsi usanzwe, iyo wakoze kino gikorwa, ukoresha amasaha angahe?<br>*Gerageza wandi ighe mu icyikubo cya 15 (Urugero: niba umuntu avuze ko yakoresheje iminota 15 ku murimo andiko 0.25. burungushura ugeze ku minota 15, burungushura indi mibare yose kuza ku gikubo cya 15 kiri hafi aho.(urugero: niba ari iminota 35 uburungushure ushyire ku minota 30, hanyuma wandike amasaha 0.5) [Andika umubare w'amasaha]*<br>*Question relevant when: ${E.32} !='997' and ${E.32a} !='0'*<br>*Response constrained to: .<=24 and .>0*	
E.33	E.33 Ukuyemo ubufasha bw'abantu baba hanze y'urugo rwanyu, ni gute wowe n'umugabo wawe musaranganya ibi bikorwa? Gukora umurimo utari uw'ubuhinzi ubyara amafaranga cyangwa indi nyungu nko gukora muri butike cg ikindi gikorwa kibyara inyungu	1 Burigihe ni wowe<br>2 Kenshi ni wowe<br>3 Turasaranganya cyangwa tubikorera hamwe<br>4 Kenshi ni uwomwashakanye<br>5 Burigihe ni

		uwomwashakanye<br>997 Ntibimureba
E.33a	E.33a Mu minsi 7 ishize, wakoze kino gikorwa iminsi ingahe?<br>*[Andika umubare w'iminsi]*<br>*Question relevant when: ${E.33} !='997'*<br>*Response constrained to: .<=7*	
E.33b	E.33b Ku munsi usanzwe, iyo wakoze kino gikorwa, ukoresha amasaha angahe?<br>*Gerageza wandi ighe mu icyikubo cya 15 (Urugero: niba umuntu avuze ko yakoresheje iminota 15 ku murimo andiko 0.25. burungushura ugeze ku minota 15, burungushura indi mibare yose kuza ku gikubo cya 15 kiri hafi aho.(urugero: niba ari iminota 35 uburungushure ushyire ku minota 30, hanyuma wandike amasaha 0.5) [Andika umubare w'amasaha]*<br>*Question relevant when: ${E.33} !='997' and ${E.33a} !='0'*<br>*Response constrained to: .<=24 and .>0*	
E.34	E.34 Ukuyemo ubufasha bw'abantu baba hanze y'urugo rwanyu, ni gute wowe n'umugabo wawe musaranganya ibi bikorwa? Gukora umurimo utari uw'ubuhinzi ubyara amafaranga cyangwa indi nyungu ukorerwa undi umuntu wo mumuryango atawuhemberwa?	1 Burigihe ni wowe<br>2 Kenshi ni wowe<br>3 Turasaranganya cyangwa tubikorera hamwe<br>4 Kenshi ni uwomwashakanye<br>5 Burigihe ni uwomwashakanye<br>997 Ntibimureba
E.34a	E.34a Mu minsi 7 ishize, wakoze kino gikorwa iminsi ingahe?<br>*[Andika umubare w'iminsi]*<br>*Question relevant when: ${E.34} !='997'*<br>*Response constrained to: .<=7*	
E.34b	E.34b Ku munsi usanzwe, iyo wakoze kino gikorwa, ukoresha amasaha angahe?<br>*Gerageza wandi ighe mu icyikubo cya 15 (Urugero: niba umuntu avuze ko yakoresheje iminota 15 ku murimo andiko 0.25. burungushura ugeze ku minota 15, burungushura indi mibare yose kuza ku gikubo cya 15 kiri hafi aho.(urugero: niba ari iminota 35 uburungushure ushyire ku minota 30, hanyuma wandike amasaha 0.5) [Andika umubare w'amasaha]*<br>*Question relevant when: ${E.34} !='997' and ${E.34a} !='0'*<br>*Response constrained to: .<=24 and .>0*	
E.35	E.35 Ukuyemo ubufasha bw'abantu baba hanze y'urugo rwanyu, ni gute wowe n'umugabo wawe musaranganya ibi bikorwa? Gusana inzu	1 Burigihe ni wowe<br>2 Kenshi ni wowe<br>3 Turasaranganya cyangwa tubikorera hamwe<br>4 Kenshi ni uwomwashakanye<br>5 Burigihe ni uwomwashakanye<br>997 Ntibimureba
E.35a	E.35a Mu minsi 7 ishize, wakoze kino gikorwa iminsi ingahe?<br>*[Andika umubare w'iminsi]*<br>*Question relevant when: ${E.35} !='997'*<br>*Response constrained to: .<=7*	
E.35b	E.35b Ku munsi usanzwe, iyo wakoze kino gikorwa, ukoresha amasaha angahe?<br>*Gerageza wandi ighe mu icyikubo cya 15 (Urugero: niba umuntu avuze ko yakoresheje iminota 15 ku murimo andiko 0.25. burungushura ugeze ku minota 15, burungushura indi mibare yose kuza ku gikubo cya 15 kiri hafi aho.(urugero: niba ari iminota 35 uburungushure ushyire ku minota 30, hanyuma wandike amasaha 0.5) [Andika umubare w'amasaha]*<br>*Question relevant when: ${E.35} !='997' and ${E.35a} !='0'*<br>*Response constrained to: .<=24 and .>0*	
E.36	E.36 Ukuyemo ubufasha bw'abantu baba hanze y'urugo rwanyu, ni gute wowe n'umugabo wawe musaranganya ibi bikorwa: Gusasa igitanda	1 Burigihe ni wowe<br>2 Kenshi ni wowe<br>3 Turasaranganya cyangwa tubikorera hamwe<br>4 Kenshi ni uwomwashakanye<br>5 Burigihe ni uwomwashakanye<br>997 Ntibimureba
E.36a	E.36a Mu minsi 7 ishize, wakoze kino gikorwa iminsi ingahe?<br>*[Andika umubare w'iminsi]*<br>*Question relevant when: ${E.36} !='997'*<br>*Response constrained to: .<=7*	
E.36b	E.36b Ku munsi usanzwe, iyo wakoze kino gikorwa, ukoresha amasaha angahe?<br>*Gerageza wandi ighe mu icyikubo cya 15 (Urugero: niba umuntu avuze ko yakoresheje iminota 15 ku murimo andiko 0.25. burungushura ugeze ku minota 15, burungushura indi mibare yose kuza ku gikubo cya 15 kiri hafi aho.(urugero: niba ari iminota 35 uburungushure ushyire ku minota 30, hanyuma wandike amasaha 0.5) [Andika umubare w'amasaha]*<br>*Question relevant when: ${E.36} !='997' and ${E.36a} !='0'*<br>*Response constrained to: .<=24 and .>0*	

E.37	E.37 Mu minsi 7 ishize, wasabanye n'inshuti n'abandimwe iminsi ingahe?<br>*[Andika umubare w'iminsi]*<br>*Response constrained to: .<=7*	
E.37a	E.37a Ku munsi usanzwe, iyo wakoze kino gikorwa, ukoresha amasaha angahe?<br>*Gerageza wandi ighe mu icyikubo cya 15 (Urugero: niba umuntu avuze ko yakoresheje iminota 15 ku murimo andiko 0.25. burungushura ugeze ku minota 15, burungushura indi mibare yose kuza ku gikubo cya 15 kiri hafi aho.(urugero: niba ari iminota 35 uburungushure ushyire ku minota 30, hanyuma wandike amasaha 0.5) [Andika umubare w'amasaha]*<br>*Question relevant when: ${E.37} !='0'*<br>*Response constrained to: .<=24 and .>0*	
E.38	E.38 Ugereranyije waryame amasaha angahe ku munsi mu minsi 7 ishize?<br>*Gerageza wandi ighe mu icyikubo cya 15 (Urugero: niba umuntu avuze ko yakoresheje iminota 15 ku murimo andiko 0.25. burungushura ugeze ku minota 15, burungushura indi mibare yose kuza ku gikubo cya 15 kiri hafi aho.(urugero: niba ari iminota 35 uburungushure ushyire ku minota 30, hanyuma wandike amasaha 0.5) [Andika umubare w'amasaha]*<br>*Response constrained to: .<=24 and .>0*	
E10	E10 Wifuza ko umugabo wawe agira uruhare rungana iki mu mirimo yo mu rugo?<br>*MUSOMERE IBISUBIZO BYOSE BYATANZWE*	1 Nishimiye uruhare rwe<br>2 Nakwifuje yuko yongera uruhare rwe<br>3 Nifuza ko yagabanya uruhare rwe<br>998 Simbizi

survey > PART 6. Inzoga

Group relevant when: ${A01} >=18

note6	Ngiye kukuganiriza ku ikoreshwa ry'inzoga. Nk'uko nakomeje kubikwizeza ibisubizo uribumpe biraba ibanga.	
F01	F01 Kuva mu mwaka ushize (kuva kwibazwa riheruka), umaze kunywa inzoga inshuro zingahe?<br>*MUSOMERE IBISUBIZO BYOSE BYATANZWE*	0 Nta na rimwe<br>1 Inshuro nke mu mezi icyenda ashize<br>2 Rimwe buri mezi abiri (2)<br>3 Rimwe buri kwezi<br>4 Inshuro nke mu kwezi<br>5 Rimwe cyangwa kabiri mu cyumweru<br>6 Buri munsi cyangwa hafi ya buri munsi<br>998 Simbizi<br>999 Yanze gusubiza
F01b	F01b Mu mwaka ushize (kuva kwibazwa riheruka), ni kangahe wanyweye inzoga nyinshi kugeza aho usinda?<br>*ICYITONDERWA: SOMERA UBAZWA IBISUBIZO*<br>*Question relevant when: ${F01} !='0'*	0 Nta na rimwe<br>1 Inshuro nke mu mezi icyenda ashize<br>2 Rimwe buri mezi abiri (2)<br>3 Rimwe buri kwezi<br>4 Inshuro nke mu kwezi<br>5 Rimwe cyangwa kabiri mu cyumweru<br>6 Buri munsi cyangwa hafi ya buri munsi<br>998 Simbizi<br>999 Yanze gusubiza
F03	F03 Mu mwaka ushize (kuva kwibazwa riheruka), ni kangahe umugabo wawe amaze kunywa inzoga?	0 Nta na rimwe<br>1 Gake gashoboka<br>2 Rimwe na rimwe<br>3 Kenshi<br>998 Simbizi<br>999 Yanze gusubiza
F04	F04 Mu mwaka ushize (kuva kwibazwa riheruka), ni kangahe umugabo wawe yanyweye inzoga nyinshi kugeza aho asinda?<br>*MUSOMERE IBISUBIZO BYOSE BYATANZWE*<br>*Question relevant when: ${F03} !='0'*	0 Ntibirabaho (aranywa ariko ntageza aho gusinda)<br>1 Inshuro nke kuva ku ibazwa riheruka<br>2 Rimwe buri mezi abiri<br>3 Rimwe mu kwezi<br>4 Inshuro nke mu kwezi<br>5 Rimwe cyangwa kabiri mu cyumweru<br>6 Buri munsi cyangwa hafi ya buri munsi

		998	Simbizi
		999	Yanze gusubiza
F05	F05 Mu mwaka ushize (kuva kwibazwa riheruka), ni kangahe umugabo wawe yananiwe gukora ibyo yagombaga gukora kubera kunywa inzoga?<br>*Question relevant when: ${F03} !='0'*	0	Nta na rimwe
		1	Gake gashoboka
		2	Rimwe na rimwe
		3	Kenshi
		998	Simbizi
		999	Yanze gusubiza
F06	F06 Umugabo wawe yigeze akomereka cyangwa akomeretsa undi muntu kubera gusinda?<br>*Question relevant when: ${F03} !='0'*	0	Oya
		1	Yego
		998	Simbizi
		999	Yanze gusubiza
survey > PART 7. Ihohoterwa rikorerwa hagati y'abashakanye n'irishingiye kugitsina<br>*Group relevant when: ${A01} >=18*			
note7	Ngiye kukubaza ibibazo ku bintu byabaye mu minanire yawe n'umugabo wawe mu mwaka ushize (kuva kwibazwa riheruka). Ibibazo birabaza niba ari kangahe wagezweho n'ibyo bintu mu mwaka ushize. Ushobora gusanga ibi bibazo bikomeye kubisubiza, ariko twizeye ko uza kubisubiza wisanzuye. Ubu bushakashatsi ni ingenzi cyane mu gushaka kumenya imibereho y'abagore mu Rwanda. Ndakwibutsa ko ibisubizo byawe bigirwa ibinga. Ntabwo ugomba gusubiza mu gihe wumva utabishaka.		
G01	G01 Ngiye kukubaza uruhererekane rw'ibibazo ku bikunze kuba ku bagore benshi kandi umugabo wawe ashobora kuba yaragukoreye. Uhitamo igisubizo wumva kijyanye neza n'igihe kiri mu mwaka ushize.<br>*MUSOMERE IBISUBIZO BYOSE BYATANZWE. Iki kibazo kirabaza ibyabaye kuva ibazwa ryo muri Ugushyingo/ Ukuboza (Umwaka ushize).*		
G01a	G01a Mu mwaka ushize (kuva kwibazwa riheruka), ni kangahe umugabo wawe yakurakariye abonye uvugisha abandi bagabo?	0	Nta na rimwe
		1	Rimwe
		2	Inshuro nke
		3	Kenshi
		999	Yanze gusubiza
		997	Ntibimureba
G01b	G01b Mu mwaka ushize (kuva kwibazwa riheruka), ni kangahe umugabo wawe yagutitirije ashaka kumenya aho uba uri igihe cyose?	0	Nta na rimwe
		1	Rimwe
		2	Inshuro nke
		3	Kenshi
		999	Yanze gusubiza
		997	Ntibimureba
G01c	G01c Mu mwaka ushize (kuva kwibazwa riheruka), ni kangahe umugabo wawe yagerageje kukubuza kubonana n'inshuti zawe?	0	Nta na rimwe
		1	Rimwe
		2	Inshuro nke
		3	Kenshi
		999	Yanze gusubiza
		997	Ntibimureba
G01d	G01d Mu mwaka ushize (kuva kwibazwa riheruka), ni kangahe umugabo wawe yagerageje kukubuza kuvugana n'umuryango wawe?	0	Nta na rimwe
		1	Rimwe
		2	Inshuro nke
		3	Kenshi
		999	Yanze gusubiza
		997	Ntibimureba
G02	G02 Umugabo wawe yaba agukeka kenshi ko umuca inyuma?	0	Oya
		1	Yego
		998	Simbizi
		999	Yanze gusubiza
G03	G03 Ngiye kukubaza ku bikunze kuba ku bagore benshi. Ndakwibutsa ko ibisubizo byose utanga bigirwa ibanga. Ntutegetswe gusubiza mu gihe utabishaka. Ngiye kukubaza uruhererekane rw'ibibazo. Uhitamo igisubizo wumva kijyanye neza n'igihe cy'umwaka ushize.<br>*MUSOMERE IBISUBIZO BYOSE BYATANZWE. Iki kibazo kirabaza ibyabaye kuva ibazwa ryo muri Ugushyingo/ Ukuboza (Umwaka ushize).*		
G03a	G03a Mu mwaka ushize (kuva kwibazwa riheruka), ni inshuro zingahe umugabo wawe yagukubise urushyi cyangwa yaguteye ikindi ikintu cyashoboraga kugukomeretsa?	0	Nta na rimwe
		1	Rimwe
		2	Inshuro nke
		3	Kenshi

		999	Yanze gusubiza
		997	Ntibimureba
G03b	G03b Mu mwaka ushize (kuva kwibazwa riheruka), ni inshuro zingahe umugabo wawe yagusunitse cyangwa yaguhutaje?	0	Nta na rimwe
		1	Rimwe
		2	Inshuro nke
		3	Kenshi
		999	Yanze gusubiza
		997	Ntibimureba
G03c	G03c Mu mwaka ushize (kuva kwibazwa riheruka), ni inshuro zingahe umugabo wawe yaguteye igipfunsi cyangwa yagukubitishije ikindi kintu cyashoboraga kugukomeretsa cyangwa kukubabaza?	0	Nta na rimwe
		1	Rimwe
		2	Inshuro nke
		3	Kenshi
		999	Yanze gusubiza
		997	Ntibimureba
G03d	G03d Mu mwaka ushize (kuva kwibazwa riheruka), ni inshuro zingahe umugabo wawe yaguteye imigeri, cyangwa yagukurubanye, cyangwa yagukubise, cyangwa yakunize, cyangwa yagutwitse?	0	Nta na rimwe
		1	Rimwe
		2	Inshuro nke
		3	Kenshi
		999	Yanze gusubiza
		997	Ntibimureba
G03e	G03e Mu mwaka ushize (kuva kwibazwa riheruka), ni inshuro zingahe umugabo wawe yagukangishije kugutera icyuma cyangwa kugukubita inkoni?	0	Nta na rimwe
		1	Rimwe
		2	Inshuro nke
		3	Kenshi
		999	Yanze gusubiza
		997	Ntibimureba
G03f	G03f Ni kangahe ibi tumaze kuvuga byabaye abana bahari?<br>*Niba usubiza nta bana afite koresha 'Ntibimureba'*<br>*Question relevant when: not((${G03a} ='0' or ${G03a} ='999') and (${G03b} ='0' or ${G03b} ='999') and (${G03c} ='0' or ${G03c} ='999') and (${G03d} ='0' or ${G03d} ='999') and (${G03e} ='0' or ${G03e} ='999'))*	0	Nta na rimwe
		1	Rimwe
		2	Inshuro nke
		3	Kenshi
		999	Yanze gusubiza
		997	Ntibimureba
G03g	G03g Mu mwaka ushize (kuva kwibazwa riheruka), ni inshuro zingahe umugabo wawe yaguhatiye gukora imibonano mpuzabitsina igihe wowe utabishakaga?	0	Nta na rimwe
		1	Rimwe
		2	Inshuro nke
		3	Kenshi
		999	Yanze gusubiza
		997	Ntibimureba
G03h	G03h Mu mwaka ushize (kuva kwibazwa riheruka), ni inshuro zingahe umugabo wawe yagututse cyangwa yagusebeje nkana?	0	Nta na rimwe
		1	Rimwe
		2	Inshuro nke
		3	Kenshi
		999	Yanze gusubiza
		997	Ntibimureba
G03i	G03i Mu mwaka ushize (kuva kwibazwa riheruka), ni inshuro zingahe umugabo wawe yagupfobeje cyangwa yagusuzuguje imbere y'abandi?	0	Nta na rimwe
		1	Rimwe
		2	Inshuro nke
		3	Kenshi
		999	Yanze gusubiza
		997	Ntibimureba
G03j	G03j Mu mwaka ushize (kuva kwibazwa riheruka), ni inshuro zingahe umugabo wawe yakoze ibintu bigutera gutinya cyangwa bigutera ubwoba yabigambiriye? urugero: kukureba igitsure, kugukankamira, guhondagura cyangwa kumenagura ibintu?	0	Nta na rimwe
		1	Rimwe
		2	Inshuro nke
		3	Kenshi
		999	Yanze gusubiza
		997	Ntibimureba
G03k	G03k Mu mwaka ushize (kuva kwibazwa riheruka), ni inshuro zingahe umugabo wawe yagukangishije kukugirira nabi?	0	Nta na rimwe
		1	Rimwe
		2	Inshuro nke
		3	Kenshi

		999	Yanze gusubiza
		997	Ntibimureba
G03l	G03l Mu mwaka ushize (kuva kwibazwa riheruka), ni inshuro zingahe umugabo wawe yagiriye nabi abantu ukunda agambiriye kugushavuza cyangwa yononnye ibintu bigufitiye akamaro?	0	Nta na rimwe
		1	Rimwe
		2	Inshuro nke
		3	Kenshi
		999	Yanze gusubiza
		997	Ntibimureba
G03m	G03m Mu mwaka ushize (kuva kwibazwa riheruka), ni inshuro zingahe umugabo wawe yakubujije gushaka akazi, kujya ku kazi, gucuruza cyangwa gukorera amafaranga?	0	Nta na rimwe
		1	Rimwe
		2	Inshuro nke
		3	Kenshi
		999	Yanze gusubiza
		997	Ntibimureba
G03n	G03n Mu mwaka ushize (kuva kwibazwa riheruka), ni inshuro zingahe umugabo wawe yafashe amafaranga yawe wakoreye utabimwemereye?	0	Nta na rimwe
		1	Rimwe
		2	Inshuro nke
		3	Kenshi
		999	Yanze gusubiza
		997	Ntibimureba
G03o	G03o Mu mwaka ushize (kuva kwibazwa riheruka), ni inshuro zingahe umugabo wawe yakwirukanye mu rugo?	0	Nta na rimwe
		1	Rimwe
		2	Inshuro nke
		3	Kenshi
		999	Yanze gusubiza
		997	Ntibimureba
G03p	G03p Mu mwaka ushize (kuva kwibazwa riheruka), ni inshuro zingahe umugabo wawe yafashe amafaranga ye akorera agamije kuyanywera inzoga, kugura itabi cyangwa ibindi bintu bye yishakira mu gihe yari abizi ko bigukomereye kubona amafaranga ukoresha mu rugo?	0	Nta na rimwe
		1	Rimwe
		2	Inshuro nke
		3	Kenshi
		999	Yanze gusubiza
		997	Ntibimureba
G03q	G03q Mu mwaka ushize (kuva kwibazwa riheruka), ni kangahe wemeye gukora imibonano mpuzabitsina kubera gutinya ibyo umugabo wawe yakora uramutse ubyanze?	0	Nta na rimwe
		1	Rimwe
		2	Inshuro nke
		3	Kenshi
		999	Yanze gusubiza
		997	Ntibimureba
G03r	G03r Mu mwaka ushize (kuva kwibazwa riheruka), ni kangahe umugabo wawe yakangishije kubabaza abana banyu? *Niba usubiza nta bana afite koresha 'Ntibimureba'*	0	Nta na rimwe
		1	Rimwe
		2	Inshuro nke
		3	Kenshi
		997	Ntibimureba
		999	Yanze gusubiza
G03s	G03s Mu mwaka ushize (kuva kwibazwa riheruka), ni kangahe umugabo wawe yakangishije kukwambura abana? *Niba usubiza nta bana afite koresha 'Ntibimureba'*	0	Nta na rimwe
		1	Rimwe
		2	Inshuro nke
		3	Kenshi
		997	Ntibimureba
		999	Yanze gusubiza
G03t	G03t Mu mwaka ushize (kuva kwibazwa riheruka), ni kangahe wakubise cyangwa wakomerekeje umugabo wawe atakubanje?	0	Nta na rimwe
		1	Rimwe
		2	Inshuro nke
		3	Kenshi
		999	Yanze gusubiza
		997	Ntibimureba
G03u	G03u Mu mwaka ushize (kuva kwibazwa riheruka), ni kangahe watewe ubwoba n'ibyo umugabo wawe ashobora kugukorera cyangwa gukorera abana bawe?	0	Nta na rimwe
		1	Rimwe
		2	Inshuro nke
		3	Kenshi

		999	Yanze gusubiza
		997	Ntibimureba
G03v	G03v Mu mwaka ushize (kuva kwibazwa riheruka), ni inshuro zingahe umugabo wawe yakubujije gukoresha amafaranga yawe wakoreye uko washakaga kuyakoresha?	0	Nta na rimwe
		1	Rimwe
		2	Inshuro nke
		3	Kenshi
		999	Yanze gusubiza
		997	Ntibimureba
note_thanks_7	ICYITONDERWA K'UBAZA: Iki gice cyibaza gishobora kuba aricyo kigoranye. Hano munsi twatanze ingero z'amagambo ushobora guhitamo mugihe urimo gushimira ubazwa kuba yatubwiye kubyamubayeho. Muguhitamo aya magambo ashimira toranya amwe ajyanye n'iribaza. Niba umugore yavuze ihohoterwa yakorewe:<br><br>Nagirango ngushimire byimazeyo uburyo wadufashije. Nabonye ko ibi bibazo bishobora kuba byari bikomeye kubisubiza. Mu by'ukuri, tubasha kumenya imibereho y'abagore iyo tuganiriye nabo tukabatega amatwi bakatubwira ibyabo. Mpereye kubyo umaze kumbwira, biraboneka ko wagize ibihe bigoranye mu buzima bwawe. Biragaragara kandi ko ukomeye kuba warabashije kuva muri izo ngorane.<br>Mu gusoza ikiganiro ndaza kukubwira imiryango ifasha abagore mu murenge wanyu. Uzabegere nuramuka wumva ukeneye ubufasha bwabo, kandi ushobora no guha uru rutonde undi mugore wese ushobora gukenera ubwo bufasha. Nongeye kugushimira kuba wasubije ibi bibazo.		
note_thanks_7a	ICYITONDERWA K'UBAZA: Iki gice cyibaza gishobora kuba aricyo kigoranye. Hano munsi twatanze ingero z'amagambo ushobora guhitamo mugihe urimo gushimira ubazwa kuba yatubwiye kubyamubayeho. Muguhitamo aya magambo ashimira toranya amwe ajyanye n'iribaza.<br><br>Niba umugore atavuze ihohoterwa yaba yarakorewe:<br><br>Nagirango ngushimire byimazeyo uburyo wadufashije. Ndagushimira umwanyawawe wigomwe. Ndumva ibi ari ibibazo bireba umuntu ku giti cye, mu by'ukuri, tubasha kumva ibijyanye n’imibanire n’imiryango iyo tuganiriye n’abagore tukabatega amatwi bakatubwira.<br><br>Uramutse wumvise undi mugore ukeneye ubufasha, nyuma y’iri bazwa ndaza kuguha amakuru ku mishinga ifasha abagore mu murenge wanyu. Ushobora guha aya makuru abandi wakumva bayakeneye.		

survey > PART 8. Imyumvire irebana n'uburinganire

Group relevant when: ${A01} >=18

note_8	Ibi bibazo bikurikira birabaza ibirebana n'imyumvire yawe ku mibanire y'umugabo n'umugore. Ngiye kugusomera interuro zikurikira, urajya umbwira niba ibikubiyemo ubyemera cyane, niba ubyemera, niba ntacyo wabivugaho, niba ubihakana, cyangwa niba utabyemera na gato.		
H01	H01 Inshingano zikomeye z’umugore ni ukwita ku rugo rwe no gutekera abagize umuryango we.	1	Ndabyemera cyane
		2	Ndabyemera
		3	Ntacyo nabivugaho
		4	Simbyemera
		5	Simbyemera na gato
		999	Yanze gusubiza
H02	H02 Kwambika, gukarabya no kugaburira abana ni inshingano z’abagore.	1	Ndabyemera cyane
		2	Ndabyemera
		3	Ntacyo nabivugaho
		4	Simbyemera
		5	Simbyemera na gato
		999	Yanze gusubiza
H04	H04 Umugabo niwe ugomba gufata icyemezo cya nyuma mu rugo rwe	1	Ndabyemera cyane
		2	Ndabyemera
		3	Ntacyo nabivugaho
		4	Simbyemera
		5	Simbyemera na gato
		999	Yanze gusubiza
H05	H05 Umugabo agomba kubahwa nk'umutware w'urugo.	1	Ndabyemera cyane
		2	Ndabyemera
		3	Ntacyo nabivugaho
		4	Simbyemera
		5	Simbyemera na gato
		999	Yanze gusubiza

H10	H10 Umugore w'umutima ntiyibaza kubyemezo by'umugabo we n'ubwo yaba atemeranywa nawe.		1	Ndabyemera cyane
			2	Ndabyemera
			3	Ntacyo nabivugaho
			4	Simbyemera
			5	Simbyemera na gato
			999	Yanze gusubiza
H11	H11 Umugore niwe ufite inshingano zo kwirinda gusama.		1	Ndabyemera cyane
			2	Ndabyemera
			3	Ntacyo nabivugaho
			4	Simbyemera
			5	Simbyemera na gato
			999	Yanze gusubiza
H13	H13 Ni byiza ko abagore bakora kugirango urugo rubone ibirutunga bihagije.		1	Ndabyemera cyane
			2	Ndabyemera
			3	Ntacyo nabivugaho
			4	Simbyemera
			5	Simbyemera na gato
			999	Yanze gusubiza
H16	H16 Umugore ugendana agakingirizo “aba ari indaya."		1	Ndabyemera cyane
			2	Ndabyemera
			3	Ntacyo nabivugaho
			4	Simbyemera
			5	Simbyemera na gato
			999	Yanze gusubiza
H19	H19 Birasanzwe kandi birakwiye ko abagabo bagira ububasha buruta ubw'abagore mu muryango.		1	Ndabyemera cyane
			2	Ndabyemera
			3	Ntacyo nabivugaho
			4	Simbyemera
			5	Simbyemera na gato
			999	Yanze gusubiza
H20	H20 Ihohoterwa rishingiye ku gitsina (gufata ku ngufu) ribaho hagati y'abashakanye.		1	Ndabyemera cyane
			2	Ndabyemera
			3	Ntacyo nabivugaho
			4	Simbyemera
			5	Simbyemera na gato
			999	Yanze gusubiza
H21	H21 Rimwe na rimwe umugore agomba gukubitwa		1	Ndabyemera cyane
			2	Ndabyemera
			3	Ntacyo nabivugaho
			4	Simbyemera
			5	Simbyemera na gato
			999	Yanze gusubiza
H22	H22 Umugore ashobora gusaba umugabo we ko bakora imibonano mpuzabitsina.		1	Ndabyemera cyane
			2	Ndabyemera
			3	Ntacyo nabivugaho
			4	Simbyemera
			5	Simbyemera na gato
			999	Yanze gusubiza
H33	H33 Iyo umugabo atetse cyangwa akoze isuku, kiba ari igisebo ku mugore we		1	Ndabyemera cyane
			2	Ndabyemera
			3	Ntacyo nabivugaho
			4	Simbyemera
			5	Simbyemera na gato
			999	Yanze gusubiza
H23	H23 Umugabo niwe ufata icyemezo cya nyuma cy’ikoreshwa ry’amafaranga mu rugo.		1	Ndabyemera cyane
			2	Ndabyemera
			3	Ntacyo nabivugaho
			4	Simbyemera
			5	Simbyemera na gato
			999	Yanze gusubiza

H24	H24 Umugore yakagombye kwihanganira ihohoterwa kugira ngo urugo rwe rudasenyuka.	1 Ndabyemera cyane<br>2 Ndabyemera<br>3 Ntacyo nabivugaho<br>4 Simbyemera<br>5 Simbyemera na gato<br>999 Yanze gusubiza
H25	H25 Niba amafaranga y'ishuli ari makeya, ni byiza kuyaharira abana b'abahungu.	1 Ndabyemera cyane<br>2 Ndabyemera<br>3 Ntacyo nabivugaho<br>4 Simbyemera<br>5 Simbyemera na gato<br>999 Yanze gusubiza
H26	H26 Iyo umwana akoze nabi agomba gukubitwa.	1 Ndabyemera cyane<br>2 Ndabyemera<br>3 Ntacyo nabivugaho<br>4 Simbyemera<br>5 Simbyemera na gato<br>999 Yanze gusubiza
H28	H28 Umugabo ashobora kwita ku bana kimwe nk'uko umugore abikora.	1 Ndabyemera cyane<br>2 Ndabyemera<br>3 Ntacyo nabivugaho<br>4 Simbyemera<br>5 Simbyemera na gato<br>999 Yanze gusubiza
H29	H29 Umugore yagombye kwihanganira ingorane zose ahura nazo mu rugo rwe (niko zubakwa).	1 Ndabyemera cyane<br>2 Ndabyemera<br>3 Ntacyo nabivugaho<br>4 Simbyemera<br>5 Simbyemera na gato<br>999 Yanze gusubiza
H31	H31 Umugabo aramutse abwiye inshuti ze ko afatanya n'umugore we mu gufata ibyemezo, inshuti ze ntizaba zikimwubashye	1 Ndabyemera cyane<br>2 Ndabyemera<br>3 Ntacyo nabivugaho<br>4 Simbyemera<br>5 Simbyemera na gato<br>999 Yanze gusubiza
H32	H32 Umugabo ugaragaye atetse cyangwa asukura inzu ye/urugo rwe abandi batuye muri ako gace baramukwena	1 Ndabyemera cyane<br>2 Ndabyemera<br>3 Ntacyo nabivugaho<br>4 Simbyemera<br>5 Simbyemera na gato<br>999 Yanze gusubiza
note_h	Muri aka kanya ndagirango nkubaze ibibazo bike byerekeranye n'ihohoterwa aho utuye. Nabibutsaga ko amakuru yose muri buduhe azaba ari ibanga.	
H36	H36 Igihe umugabo akubise umugore we, utekereza ko ari ngombwa ko abandi bo hanze babakiza?	0 Oya<br>1 Yego
H37	H37 Ku bwawe, umugabo afite impamvu yumvikana ituma akubita umugore we mu gihe:	
H37a	H37a Ku bwawe, umugabo afite impamvu yumvikana ituma akubita umugore we mu gihe: atamwubashye	0 Oya<br>1 Yego<br>999 Yanze gusubiza
H37b	H37b Ku bwawe, umugabo afite impamvu yumvikana ituma akubita umugore we mu gihe: yanze gukorana imibonano mpuzabitsina nawe	0 Oya<br>1 Yego<br>999 Yanze gusubiza
H37c	H37c Ku bwawe, umugabo afite impamvu yumvikana ituma akubita umugore we mu gihe: asanze yajyaga amuca inyuma	0 Oya<br>1 Yego<br>999 Yanze gusubiza
H37d	H37d Ku bwawe, umugabo afite impamvu yumvikana ituma akubita umugore we mu gihe: yirengagiza kwita ku mwana (abana)	0 Oya<br>1 Yego<br>999 Yanze gusubiza

H37e	H37e Ku bwawe, umugabo afite impamvu yumvikana ituma akubita umugore we mu gihe: adakora imirimo yo mu rugo ku buryo bumushimisha	0	Oya
		1	Yego
		999	Yanze gusubiza
H37f	H37f Ku bwawe, umugabo afite impamvu yumvikana ituma akubita umugore we mu gihe: umugore amushinja kumuca inyuma	0	Oya
		1	Yego
		999	Yanze gusubiza
H37g	H37g Ku bwawe, umugabo afite impamvu yumvikana ituma akubita umugore we mu gihe: yakoresheje amafaranga atabanje kumugisha inama	0	Oya
		1	Yego
		999	Yanze gusubiza
H37h	H37h Ku bwawe, umugabo afite impamvu yumvikana ituma akubita umugore we mu gihe: atakira abashyitsi mu rugo kuburyo bumushimisha	0	Oya
		1	Yego
		999	Yanze gusubiza
H37i	H37i Ku bwawe, umugabo afite impamvu yumvikana ituma akubita umugore we mu gihe: Asanze atateguye ibyo kurya	0	Oya
		1	Yego
		999	Yanze gusubiza
H38	H38 Umugore ufite umugabo aramutse akubiswe n'umugabo we, wumva bikwiye ko yabibwira abandi?	0	Oya
		1	Yego

survey > PART 9. Ibikorwa by'ubukangurambaga n'amahuriro atanga ubufasha

Group relevant when: ${A01} >=18

note9	Turi hafi gusoza ikiganiro cyacu.		
I01	I01 Ngiye kugusomera interuro zikurikira, urajya umbwira niba ibikubiyemo ubyemera cyane, niba ubyemera, niba ntacyo wabivugaho, niba ubihakana, cyangwa niba utabyemera na gato.		
I01a	I01a Ndi intangarugero aho ntuye	1	Ndabyemera cyane
		2	Ndabyemera
		3	Ntacyo nabivugaho
		4	Simbyemera
		5	Simbyemera na gato
I01b	I01b Numva ubuzima bwanjye bufitiye akamaro abandi	1	Ndabyemera cyane
		2	Ndabyemera
		3	Ntacyo nabivugaho
		4	Simbyemera
		5	Simbyemera na gato
I01c	I01c Muri rusange mfite byinshi byo kwishimira	1	Ndabyemera cyane
		2	Ndabyemera
		3	Ntacyo nabivugaho
		4	Simbyemera
		5	Simbyemera na gato
I01d	I01d Numva ndi umugore ukwiye	1	Ndabyemera cyane
		2	Ndabyemera
		3	Ntacyo nabivugaho
		4	Simbyemera
		5	Simbyemera na gato
I01e	I01d Numva ndi umubyeyi mwiza	1	Ndabyemera cyane
		2	Ndabyemera
		3	Ntacyo nabivugaho
		4	Simbyemera
		5	Simbyemera na gato
I.21	I.21 Ngiye kugusomera interuro zikurikira. Urajya umbwira uko wiyumvaga mucyumweru gishize: Ntibikunzekuba cyangwa nta na rimwe, gace cyangwa inshuro nkeya, rimwe na rimwe cyangwa mukigereranyo, cyangwa igihe cyose.		
I.21a	I.21a Nahangayikishijwe n'ibintu bitajaga bimpangayikisha ubusanzwe	1	Gake gashoboka cyangwa nta na rimwe (munsi y'umunsi 1 mucyumweru)
		2	Gace cyangwa inshuro nkeya (iminsi 1-2 mucyumweru)
		3	Rimwe na rimwe cyangwa cyangwa mukigereranyo (iminsi 3-4 mucyumweru)
		4	Igihe cyose (iminsi 5-7

			mucyumweru)
		999	Yanze gusubiza
I21b	I21b Nagorwaga no gushyira ibitekerezo byanjye kubyonakoraga	1	Gake gashoboka cyangwa nta na rimwe (munsi y'umunsi 1 mucyumweru)
		2	Gace cyangwa inshuro nkeya (iminsi 1-2 mucyumweru)
		3	Rimwe na rimwe cyangwa cyangwa mukigereranyo (iminsi 3-4 mucyumweru)
		4	Igihe cyose (iminsi 5-7 mucyumweru)
		999	Yanze gusubiza
I21c	I21c Nabaye nkuwihebye	1	Gake gashoboka cyangwa nta na rimwe (munsi y'umunsi 1 mucyumweru)
		2	Gace cyangwa inshuro nkeya (iminsi 1-2 mucyumweru)
		3	Rimwe na rimwe cyangwa cyangwa mukigereranyo (iminsi 3-4 mucyumweru)
		4	Igihe cyose (iminsi 5-7 mucyumweru)
		999	Yanze gusubiza
I21d	I21d Niyumvagamo ko ibyonakoraga byose ari kwihata.	1	Gake gashoboka cyangwa nta na rimwe (munsi y'umunsi 1 mucyumweru)
		2	Gace cyangwa inshuro nkeya (iminsi 1-2 mucyumweru)
		3	Rimwe na rimwe cyangwa cyangwa mukigereranyo (iminsi 3-4 mucyumweru)
		4	Igihe cyose (iminsi 5-7 mucyumweru)
		999	Yanze gusubiza
I21e	I21e Numvaga mfite ibyiringiro by'ejo hazaza	1	Gake gashoboka cyangwa nta na rimwe (munsi y'umunsi 1 mucyumweru)
		2	Gace cyangwa inshuro nkeya (iminsi 1-2 mucyumweru)
		3	Rimwe na rimwe cyangwa cyangwa mukigereranyo (iminsi 3-4 mucyumweru)
		4	Igihe cyose (iminsi 5-7 mucyumweru)
		999	Yanze gusubiza
I21f	I21f Niyumvagamo ubwoba	1	Gake gashoboka cyangwa nta na rimwe (munsi y'umunsi 1 mucyumweru)
		2	Gace cyangwa inshuro nkeya (iminsi 1-2 mucyumweru)
		3	Rimwe na rimwe cyangwa cyangwa mukigereranyo (iminsi 3-4 mucyumweru)
		4	Igihe cyose (iminsi 5-7 mucyumweru)
		999	Yanze gusubiza

I21g	I21g Sinasinziraga neza	1	Gake gashoboka cyangwa nta na rimwe (munsi y'umunsi 1 mucyumweru)
		2	Gace cyangwa inshuro nkeya (iminsi 1-2 mucyumweru)
		3	Rimwe na rimwe cyangwa cyangwa mukigereranyo (iminsi 3-4 mucyumweru)
		4	Igihe cyose (iminsi 5-7 mucyumweru)
		999	Yanze gusubiza
I21h	I21h Nari mfite umunezero	1	Gake gashoboka cyangwa nta na rimwe (munsi y'umunsi 1 mucyumweru)
		2	Gace cyangwa inshuro nkeya (iminsi 1-2 mucyumweru)
		3	Rimwe na rimwe cyangwa cyangwa mukigereranyo (iminsi 3-4 mucyumweru)
		4	Igihe cyose (iminsi 5-7 mucyumweru)
		999	Yanze gusubiza
I21i	I21i Niyumvaga nkuwahawe akato	1	Gake gashoboka cyangwa nta na rimwe (munsi y'umunsi 1 mucyumweru)
		2	Gace cyangwa inshuro nkeya (iminsi 1-2 mucyumweru)
		3	Rimwe na rimwe cyangwa cyangwa mukigereranyo (iminsi 3-4 mucyumweru)
		4	Igihe cyose (iminsi 5-7 mucyumweru)
		999	Yanze gusubiza
I21j	I21j Sinabashaga kugira icyo nkora	1	Gake gashoboka cyangwa nta na rimwe (munsi y'umunsi 1 mucyumweru)
		2	Gace cyangwa inshuro nkeya (iminsi 1-2 mucyumweru)
		3	Rimwe na rimwe cyangwa cyangwa mukigereranyo (iminsi 3-4 mucyumweru)
		4	Igihe cyose (iminsi 5-7 mucyumweru)
		999	Yanze gusubiza
I02	I02 Mu mwaka ushize (kuva kwibazwa riheruka), waba warigeze witabira ibikorwa by'aho utuye cyangwa aho ukorera bivuga ku ihotera abagabo bakorera abagore?<br>*Question relevant when: ${partcipant_group} ='2'*	0	Oya
		1	Yego
		999	Yanze gusubiza
I03	I03 Mu mwaka ushize (kuva kwibazwa riheruka), aho utuye cyangwa ukorera, waba warigeze kwitabira ibikorwa binyuranye bigamije gukangurira abagabo kugira uruhare mu buzima bw'abana babo?<br>*Question relevant when: ${partcipant_group} ='2'*	0	Oya
		1	Yego
I04	I04 Mu mwaka ushize (kuva kwibazwa riheruka),waba warigeze witabira gahunda zerekeranye n'ibiganiro k'ubuzima bw'ababyeyi aho utuye cyangwa aho ukorera?<br>*Question relevant when: ${partcipant_group} ='2'*	0	Oya
		1	Yego
I06	I06 Hari umuntu uzi wigize ajya cyangwa wigishaga mu matsinda ya BANDEBEREHO?<br>*Question relevant when: ${partcipant_group} ='2'*	0	Oya
		1	Yego, umuntu 1
		2	Yego, nzi abantu benshi
I06a	I06a Uwo muntu cyangwa abo bantu mupfana iki?	1	Dufitanye isano ya hafi

	HITAMO IBISUBIZO BYOSE BISHOBOKA<br>*Question relevant when: ${partcipant_group} ='2' and ${I06} !='0'*	2 Ni inshuti cyane<br>3 Mwene wacu wa kure<br>4 Ni umuntu ntazi neza<br>5 Umuturanyi<br>6 Undi muntu
I06b	I06b Waba warigeze usaba ubujyanama kuva kuri umwe mubakangurambaga ba RWAMREC Bandebereho cyangwa undi muntu witabiriye ayo matsinda?<br>*Question relevant when: ${partcipant_group} ='2' and ${I06} !='0'*	0 Oya<br>1 Yego<br>998 Simbizi
I07	I07 Ni inshuro zingahe umaze gusaba ubujyanama umwe mu bakangurambaga ba Bandebereho ya RWAMREC cyangwa abagize itsinda?<br>*Question relevant when: ${partcipant_group} ='2' and ${I06} !='0' and ${I06b} ='1'*	0 Nta na rimwe<br>1 Rimwe<br>2 Inshuro nkeye (2-5)<br>3 Inshuro nyinshi (zirenze 5)
noteI	Ngiye kukubaza ibibazo bike bijyanye n'ibikorwa waba waritabiriye kuva mu mwaka ushize (kuva kwibazwa riheruka).<br>*Question relevant when: ${partcipant_group} ='1'*	
I08	I08 Mu mwaka ushize (kuva kwibazwa ryashize), waba waritabiriye igikorwa icyo ari cyo cyose kitari ibiganiro by'ababyeyi bya Bandebereho ya RWAMREC kivuga ku ihohotera abagabo bakorera abagore?<br>*Question relevant when: ${partcipant_group} ='1'*	0 Oya<br>1 Yego
I09	I09 Mu mwaka ushize (kuva kwibazwa ryashize). waba waritabiriye igikorwa icyo ari cyo cyose kitari ibiganiro by'ababyeyi bya Bandebereho ya RWAMREC kivuga ku ruhare rw'abagabo mu mibereho y'abana babo?<br>*Question relevant when: ${partcipant_group} ='1'*	0 Oya<br>1 Yego
I10	I10 Mu mwaka ushize (kuva kwibazwa ryashize), waba waritabiriye igikorwa icyo ari cyo cyose kitari ibiganiro by'ababyeyi bya Bandebereho ya RWAMREC kivuga ku buzima bw'umubyeyi?<br>*Question relevant when: ${partcipant_group} ='1'*	0 Oya<br>1 Yego
I15	I15 Wigeze usangiza undi uwo ari we wese ibyo wigiye mu biganiro by'ababyeyi Bandebereho bya RWAMREC?<br>*Question relevant when: ${partcipant_group} ='1'*	0 Oya<br>1 Yego
I16	I16 Ni nde wasangije ibyo wize?<br>*MARK ALL THAT APPLY*<br>*Question relevant when: ${partcipant_group} ='1' and ${I15} !='0'*	1 Umugabo wanjye<br>2 Abana banjye<br>3 Abo dufitanye isano ya hafi<br>4 Ni inshuti cyane<br>5 Mwene wacu wa kure<br>6 Ni umuntu ntazi neza<br>7 Umuturanyi<br>8 Mu ruhame (urugero, natanze ubuhamya mu muganda)
I.21y	I.21 Mugihe cy'umwaka umwe n'igice, witabiriye ubushakashatsi bwacu kugeza inshuro 2. Mubibazo nakubajije bimwe byari byoroshye n'ibindi byari bikomeye. Ni gute kuvuga kuri ibi bintu byatumye wiyumva?	1 Neza<br>2 Nabi<br>3 Si neza kandi sinanabi
end_note	Uyu niwo musozo w'ikiganiro twagiranye.<br><br>Wakoze cyane ku bw'uyu mwanya tumaranye.<br><br>Nk'uko nabikubwiye, ndakwizeza ko ibisubizo byawe byose ari ibanga kandi ko ntaho izina ryawe rizagaragara.<br><br>[ASK THE PARTICIPANT IF SHE WANTS THE REFERRAL SHEET OF SERVICES/ SUPPORT ORGANIZATIONS IN HER SECTOR. PROVIDE IT TO THE RESPONDENT IF SHE WANTS IT.]	
