# Supplementary material for: Gender-transformative Bandebereho couples’ intervention to promote male engagement in reproductive and maternal health and violence prevention in Rwanda: Findings from a randomized controlled trial
Source: PLoS One. 2018 Apr 4;13(4):e0192756. doi: 10.1371/journal.pone.0192756 (PMC5884496; doi:10.1371/journal.pone.0192756)
Supplement: S3 File — (PDF) [file pone.0192756.s003.pdf]

Bandebereho_21_Followup_Male_survey_Final

Field	Question	Answer
survey		
survey > coversheet		
partcipant_group	ENUMERATOR: Please check on the list of participants and confirm the group	1 Treatment group<br>2 Control group
survey > Part 1. Demographic information		
A.1	A.1 How old are you?<br>*If respondent doesn't know his age, put 998; if he refuses to answer put 999 [Enter age] Note: If the respondent is under 18 the survey will terminate.*<br>*Response constrained to: .<=60 or .=998 or .=999*	
A.2	A.2 What is the highest level of school you have attained?<br>*Question relevant when: ${A.1} >=18*	0 None<br>1 Some Primary<br>2 Primary Completed<br>3 Some Secondary<br>4 Secondary Completed<br>5 TVET/TTC<br>6 Some University<br>999 Refused to answer
A.4	A.4 What is your main source of employment?<br>*Question relevant when: ${A.1} >=18*	1 Employed / earning a wage<br>2 Self-employed<br>3 Unemployed, but looking for work<br>4 Unemployed and not looking for work<br>5 Unable to work / disabled<br>999 Refused to answer
survey > Part 1. Demographic information > afford_item<br>*Group relevant when: ${A.1} >=18*		
A.5	A.5 How often can your household afford the following items:<br>*Consider the frequency that the participant is unable to meet any of the needs listed (not all needs). For example, if he always has shelter but he is only able to afford food often, select "often." If he is able to afford food only sometimes, select sometimes. PLEASE READ ALL EXAMPLES:*	1 Never<br>2 Sometimes<br>3 Often<br>4 Always
A.5.1	A.5.1 Basic Items (food or shelter)	1 Never<br>2 Sometimes<br>3 Often<br>4 Always
A.5.2	A.5.2 Important Items (clothing, or school fees, or health insurance)	1 Never<br>2 Sometimes<br>3 Often<br>4 Always
A.5.3	A.5.3 Extra Items (gifts or travel)	1 Never<br>2 Sometimes<br>3 Often<br>4 Always
A.7	A.7 What is your marital status?<br>*If respondent says he is married, probe to know whether legally married or not.*<br>*Question relevant when: ${A.1} >=18*	1 Single<br>2 Legally married<br>3 Partner, but not legally married<br>4 Divorced / not living together anymore<br>5 Widowed
A.8	A.8 Do you or did you ever have more than one wife at a time?<br>*Question relevant when: ${A.1} >=18 and ${A.7} ='2' or ${A.7} ='3'*	0 No<br>1 Yes<br>999 Refused to answer
note_A12	I am going to ask you some questions about who lives with you in your household.<br>*Question relevant when: ${A.1} >=18 and ${A.7} ='2' or ${A.7} ='3'*	
A.12	A.12 How many adult men, not including yourself, live in your household?<br>*[Enter number]*<br>*Question relevant when: ${A.1} >=18 and ${A.7} ='2' or ${A.7} ='3'*	

A.13	A.13 How many adult women live in your household?<br>*[Enter number]*<br>*Question relevant when: ${A.1} >=18 and ${A.7} ='2' or ${A.7} ='3'*	
A.14	A.14 How many male children (under age 18) live in your household?<br>*This includes any male child, whether biological or not! [Enter number]*<br>*Question relevant when: ${A.1} >=18 and ${A.7} ='2' or ${A.7} ='3'*	
A.15	A.15 How many female children (under age 18) live in your household?<br>*This includes any female child, whether biological or not! [Enter number]*<br>*Question relevant when: ${A.1} >=18 and ${A.7} ='2' or ${A.7} ='3'*	
A.16	A.16 How many biological children do you have?<br>*Only count those already born, living children that are biological children. [Enter number of children]*<br>*Question relevant when: ${A.1} >=18 and ${A.7} ='2' or ${A.7} ='3'*	
A.17	A.17 What is the age and sex of each of your biological children?<br>*Ask the respondent to list all of his children, starting with his oldest child. If the respondent doesn't know the age of his child, put 998. Enter 0 if child is less than 1 year and specify months in next question.*<br>*Question relevant when: ${A.1} >=18 and ${A.16} !='0' and ${A.7} ='2' or ${A.7} ='3'*	
survey > Part 1. Demographic information > Age and sex of child number (1)<br>*Group relevant when: ${A.1} >=18 and ${A.7} ='2' or ${A.7} ='3' and ${A.16} !='0'*		(Repeated group)
A.17.1	A.17.1 Age of the Child number 1<br>*[Enter Age], If the respondent doesn't know the age of his child, put 998. Enter 0 if child is less than 1 year and specify months in next question*	
A.17.1a	A.17.1a How many months old is the child number 1?<br>*Enter number of months*<br>*Question relevant when: ${A.17.1} ='0'*<br>*Response constrained to: .<=11*	
A.17.2	A.17.2 Sex of child number 1	1 Female<br>2 Male
A.18	A.18 How many rooms are there in your household?<br>*Write down a number. Enumerator: Do not consider bathrooms, hallways, garage, cellar. Rooms divided by a curtain should be counted as two rooms. All dwellings should be included.[Enter number]*<br>*Question relevant when: ${A.1} >=18 and ${A.7} ='2' or ${A.7} ='3'*	
A.19	A.19 What is the household’s main source of water?<br>*Question relevant when: ${A.1} >=18 and ${A.7} ='2' or ${A.7} ='3'*	1 Piped water on premises<br>2 Piped community water<br>3 Catchment’s tank<br>4 Public well<br>5 Private well<br>6 Covered spring<br>7 Uncovered spring<br>8 River/Lake<br>9 Borehole<br>10 Other<br>999 Refused to answer
A.20	A.20 What is the main material of the household’s floor?<br>*Enumerator: If more than one house, refer to the main one (best quality)*<br>*Question relevant when: ${A.1} >=18 and ${A.7} ='2' or ${A.7} ='3'*	1 Beaten earth<br>2 Hardened dung<br>3 Clay tiles<br>4 Cement<br>5 Bricks<br>6 Other<br>999 Refused to answer
A.21	A.21 What is the main material of the household’s walls?<br>*Enumerator: If more than one house, refer to the main one (best quality)*<br>*Question relevant when: ${A.1} >=18 and ${A.7} ='2' or ${A.7} ='3'*	1 Mud bricks<br>2 Mud bricks covered with cement<br>3 Oven fired bricks<br>4 Cement bricks<br>5 Wooden planks<br>6 Stones<br>7 Tree trunks with mud<br>8 Tree trunks with mud and cement<br>9 Plastic sheets<br>10 Other<br>999 Refused to answer
A.22	A.22 What type of roof does the main room in your house have?<br>*Question relevant when: ${A.1} >=18 and ${A.7} ='2' or ${A.7} ='3'*	1 Thatch/leaves<br>2 Wood<br>3 Corrugated iron sheets

		4	Brick/Clay tiles
		5	Plastic/plywood
		6	Asbestos sheets
		7	Other
		999	Refused to answer
A.23	A.23 Does the household have access to electricity?<br>*Question relevant when: ${A.1} >=18 and ${A.7} ='2' or ${A.7} ='3'*	0	No
		1	Yes
		999	Refused to answer
survey > Part 1. Demographic information > own_item<br>*Group relevant when: ${A.1} >=18 and ${A.7} ='2' or ${A.7} ='3'*			
A.24	A.24 Do you or does anyone in the household own any of the following items that are in working condition?<br>*Enumerator: read out loud.*		
A24.1	A24.1 Do you or does anyone in the household own: Cell phone	0	No
		1	Yes
		999	Refused to answer
A24.2	A24.2 Do you or does anyone in the household own: Sofa	0	No
		1	Yes
		999	Refused to answer
A24.3	A24.3 Do you or does anyone in the household own: Electric/gas stove	0	No
		1	Yes
		999	Refused to answer
A24.4	A24.4 Do you or does anyone in the household own: Fridge/Freezer	0	No
		1	Yes
		999	Refused to answer
A24.5	A24.5 Do you or does anyone in the household own: Radio/Audio equipment	0	No
		1	Yes
		999	Refused to answer
A24.6	A24.6 Do you or does anyone in the household own: Landline phone	0	No
		1	Yes
		999	Refused to answer
A24.7	A24.7 Do you or does anyone in the household own: Sewing/knitting machine	0	No
		1	Yes
		999	Refused to answer
A24.8	A24.8 Do you or does anyone in the household own: Motorised vehicle including motorcycle (used or new)	0	No
		1	Yes
		999	Refused to answer
A24.9	A24.9 Do you or does anyone in the household own: Bicycle	0	No
		1	Yes
		999	Refused to answer
A24.10	A24.10 Do you or does anyone in the household own: Livestock such as cows, sheep, goats, chicken, etc.	0	No
		1	Yes
		999	Refused to answer
A24.11	A24.11 Do you or does anyone in the household own: Land	0	No
		1	Yes
		999	Refused to answer
A24.12	A24.12 Do you or does anyone in the household own: Building/house	0	No
		1	Yes
		999	Refused to answer
A.6	A.6 Who in your household has health insurance?<br>*MARK ALL THAT APPLY*<br>*Question relevant when: ${A.7} ='2' or ${A.7} ='3'*<br>*Response constrained to: count-selected(.)=1 or (count-selected(.)>=2 and not(selected(.,0)) and not(selected(.,998)) and not(selected(.,999)))*	0	None
		1	Self
		2	My wife/partner
		3	One or more of my children
		4	Other
		998	I don't know
		999	Refused to answer
A.25	A.25 Have you put aside any money in the past two weeks for savings, including any contributions to a savings group or rotating savings scheme?<br>*Question relevant when: ${A.7} ='2' or ${A.7} ='3'*	0	No
		1	Yes
		999	Refused to answer
survey > PART 2. PREGNANCY & BIRTH<br>*Group relevant when: ${A.1} >=18 and ${A.7} ='2' or ${A.7} ='3'*			

note_b	In this section I am going to ask you some questions about your experience of having children or preparing to have a child. Remember that all of your responses will be kept confidential.	
B.1	B.1 Are you currently expecting a child?	0 No<br>1 Yes<br>999 Refused to answer
B.2	B.2 When is the estimated birth date? Enter how many months until the child is born<br>*Insert 998 if respondent doesn't know. If child is due in less than one month, enter 0. [enter amount in months]*<br>*Question relevant when: ${B.1} ='1'*<br>*Response constrained to: .<=9 or .=998*	
B.3	B.3 How many times did you accompany your partner to an antenatal care (ANC) visit during the current pregnancy?<br>*[enter number]*<br>*Question relevant when: ${B.1} ='1'*	
B.3.1	B.3.1 During the last antenatal care visit you attended, did you:<br>*PLEASE READ RESPONSE OPTIONS*<br>*Question relevant when: ${B.1} ='1' and ${B.3} !='0' and ${B.3} !=998*	1 Drop her off at the entrance or wait for her outside<br>2 Wait in the health facility, but not participate in the ANC visit<br>3 Join her for at least part of the visit with the health provider<br>4 Other
B.3.2	B.3.2 Please tell us about your experience at the last antenatal care visit you attended. Do you 'agree' or 'disagree' with the following statements about your experience:<br>*Question relevant when: ${B.1} ='1' and ${B.3} !='0' and ${B.3} !=998 and ${B.3.1} ='3'*	
B.3.2.1	B.3.2.1 The health provider was very welcoming<br>*Question relevant when: ${B.1} ='1' and ${B.3} !='0' and ${B.3} !=998 and ${B.3.1} ='3'*	0 Disagree<br>1 Agree
B.3.2.2	B.3.2.2 The health provider ignored me<br>*Question relevant when: ${B.1} ='1' and ${B.3} !='0' and ${B.3} !=998 and ${B.3.1} ='3'*	0 Disagree<br>1 Agree
B.3.2.3	B.3.2.3 I learned a lot about pregnancy and birth<br>*Question relevant when: ${B.1} ='1' and ${B.3} !='0' and ${B.3} !=998 and ${B.3.1} ='3'*	0 Disagree<br>1 Agree
B.3.2.4	B.3.2.4 It helped prepare me for the birth of the child<br>*Question relevant when: ${B.1} ='1' and ${B.3} !='0' and ${B.3} !=998 and ${B.3.1} ='3'*	0 Disagree<br>1 Agree
B.3.2.5	B.3.2.5 I felt closer to my partner<br>*Question relevant when: ${B.1} ='1' and ${B.3} !='0' and ${B.3} !=998 and ${B.3.1} ='3'*	0 Disagree<br>1 Agree
B.3.3.	B.3.3. Why did you not join your partner for at least part of the ANC visit with the health provider?<br>*MARK ALL THAT APPLY*<br>*Question relevant when: ${B.1} ='1' and ${B.3} !='0' and ${B.3} !=998 and ${B.3.1} !='4' and ${B.3.1} !='3'*	1 I thought it was not necessary to go with her<br>2 It is not a man's responsibility to attend ANC<br>3 My partner did not want me to attend<br>4 Other people discouraged me from attending<br>5 The health staff said I was not allowed<br>6 I had to work<br>7 I was traveling or living away from my partner<br>8 I did not have the time to attend<br>9 I did not know I was allowed to enter the antenatal consultation<br>996 Other
B.6	B.6 Are you planning to be present at the birth of your child?<br>*Question relevant when: ${B.1} ='1' and ${B.3} !='0' and ${B.3} !=998 and ${B.3.1} !='4'*	0 No<br>1 Yes<br>999 Refused to answer
N.B.1	N.B.1 Now I would like to ask about the last pregnancy your partner had, meaning the pregnancy of your youngest living child.<br>*Question relevant when: ${A.16} !='0'*	
N.B.1.1	N.B.1.1 During your partner's last pregnancy, did you accompany her to any antenatal care (ANC) visits?<br>*Question relevant when: ${A.16} !='0'*	0 No<br>1 Yes<br>2 My partner did not attend antental care (ANC)

B.7	B.7 During your partner's last pregnancy, how many times did you accompany her to an antenatal care (ANC) visit?<br>*The questions that follow ask about the last pregnancy your partner had, meaning the pregnancy of your youngest child. Insert 998 if respondent doesn't know/remember. [enter number]*<br>*Question relevant when: ${A.16} !='0' and ${N.B.1.1} ='1'*<br>*Response constrained to: .>0*	
B.8	B.8 During the last antenatal care visit you attended, did you:<br>*PLEASE READ RESPONSE OPTIONS.*<br>*Question relevant when: ${A.16} !='0' and ${N.B.1.1} ='1' and ${B.7} !='0'*	1 Drop her off at the entrance or wait for her outside<br>2 Wait in the health facility, but not participate in the ANC visit<br>3 Join her for at least part of the visit with the health provider<br>4 Other
B.9	B.9 Please tell us about your experience at the last antenatal care visit you attended. Do you 'agree' or 'disagree' with the following statements about your experience:<br>*These questions concerns the previous pregnancy of your youngest child*<br>*Question relevant when: ${A.16} !='0' and ${N.B.1.1} ='1' and ${B.7} !='0' and ${B.8} ='3'*	
B.9.1	B.9.1 The health provider was very welcoming<br>*Question relevant when: ${A.16} !='0' and ${N.B.1.1} ='1' and ${B.7} !='0' and ${B.8} ='3'*	0 Disagree<br>1 Agree
B.9.2	B.9.2 The health provider ignored me<br>*Question relevant when: ${A.16} !='0' and ${N.B.1.1} ='1' and ${B.7} !='0' and ${B.8} ='3'*	0 Disagree<br>1 Agree
B.9.3	B.9.3 I learned a lot about pregnancy and birth<br>*Question relevant when: ${A.16} !='0' and ${N.B.1.1} ='1' and ${B.7} !='0' and ${B.8} ='3'*	0 Disagree<br>1 Agree
B.9.4	B.9.4 It helped prepare me for the birth of the child<br>*Question relevant when: ${A.16} !='0' and ${N.B.1.1} ='1' and ${B.7} !='0' and ${B.8} ='3'*	0 Disagree<br>1 Agree
B.9.5	B.9.5 I felt closer to my partner<br>*Question relevant when: ${A.16} !='0' and ${N.B.1.1} ='1' and ${B.7} !='0' and ${B.8} ='3'*	0 Disagree<br>1 Agree
B.10	B.10 Why did you not join your partner for at least part of the ANC visit with the health provider?<br>*MARK ALL THAT APPLY*<br>*Question relevant when: ${A.16} !='0' and ${N.B.1.1} ='1' and ${B.7} !='0' and ${B.8} !='3' and ${B.8} !='4'*	1 I thought it was not necessary to go with her<br>2 It is not a man's responsibility to attend ANC<br>3 My partner did not want me to attend<br>4 Other people discouraged me from attending<br>5 The health staff said I was not allowed<br>6 I had to work<br>7 I was traveling or living away from my partner<br>8 I did not have the time to attend<br>9 I did not know I was allowed to enter the antenatal consultation<br>10 Other
B.11	B.11 Where did your partner give birth?<br>*Question relevant when: ${A.16} !='0'*	1 Health facility (for example, a hospital, or polyclinic, or clinic or health center.)<br>2 At home<br>3 Other
B.12a	B.12a Where were you during the birth of your last child?<br>*PLEASE READ RESPONSE OPTIONS.*<br>*Question relevant when: ${A.16} !='0' and ${B.11} ='1'*	1 I was in the same room with my partner<br>2 I was at the health facility, but not in the room with my partner<br>3 I was not at the health facility<br>4 Other
B.12b	B.12b Where were you during the birth of your last child?<br>*PLEASE READ RESPONSE OPTIONS.*<br>*Question relevant when: ${A.16} !='0' and ${B.11} ='2'*	1 I was in the same room with my partner<br>2 I was at home, but not in the room with my partner<br>3 I was not at home<br>4 Other

B.13	B.13 After the birth, how did you feel about having been in the room with your partner?<br>*PLEASE READ RESPONSE OPTIONS AND MARK ALL THAT APPLY.*<br>*Question relevant when: ${A.16} !='0' and ${B.11} !='3' and (${B.12a} ='1' or ${B.12b} ='1')*	1 I felt closer to my partner<br>2 I felt closer to my child<br>3 I appreciated my partner more because of her suffering<br>4 I was disgusted<br>5 I felt useless<br>6 I was scared
B.14	B.14 Could you tell me why you were not in the room with your partner when she gave birth?<br>*READ RESPONSE OPTIONS AND MARK ALL THAT APPLY*<br>*Question relevant when: ${B.12a} ='2' or ${B.12a} ='3'or ${B.12a} ='4' or ${B.12b} ='2' or ${B.12b} ='3' or ${B.12b} ='4'*	1 I thought it was not necessary to go with my partner<br>2 It is not a man’s responsibility to be present<br>3 Men should not be present at childbirth<br>4 My partner did not want me present<br>5 Other people discouraged me from being present<br>6 I didn't want to see my wife suffering<br>7 The health staff said I was not allowed<br>8 The birth was sudden or unexpected<br>9 I had to work<br>10 I was traveling or living away from my partner<br>11 I was afraid<br>12 Other
B.15	B.15 After the birth of your last child, did you or your partner take the child for vaccination, or did you take the child together?<br>*Question relevant when: ${A.16} !='0' and ${B.1} ='1' and ${B.3} !='0' and ${B.3} !='998'*	0 No<br>1 Yes, I took the child<br>2 Yes, my partner took the child<br>3 Yes, we both took the child, either separately or together<br>997 Not applicable
noteb2	I am going to read several statements, please tell me whether you 'agree' or 'disagree' with the following statements:	
B.26	B.26 A man should not accompany his wife to an ANC visit	0 Disagree<br>1 Agree
B.27	B.27 Being present at the birth of a child brings a man closer to the child	0 Disagree<br>1 Agree
B.28	B.28 Being present at the birth of a child brings a man closer to his partner	0 Disagree<br>1 Agree
B.29	B.29 A woman should be able to get help from a skilled birth attendant when she needs it, even without her husband's approval.	0 Disagree<br>1 Agree
N.B.2	N.B.2 It is forbidden for a man to attend more than 1 antenatal care (ANC) visit	0 Disagree<br>1 Agree
survey > PART 3: SEXUAL AND REPRODUCTIVE HEALTH<br>*Group relevant when: ${A.1} >=18 and ${A.7} ='2' or ${A.7} ='3'*		
note_c	Now I would like to ask you some questions about your sexual and reproductive health. Let me assure you again that your answers are completely confidential and will not be told to anyone. If we should come to any question that you don't want to answer, just let me know and we will go to the next question.	
C.9	C.9 Do you or your partner currently use any method to avoid or delay pregnancy?<br>*MARK ALL THAT APPLY*<br>*Response constrained to: count-selected(.)=1 or (count-selected(.)>=2 and not(selected(.,0)) and not(selected(.,998)) and not(selected(.,999)))*	0 None<br>1 Pill<br>2 Male Condom<br>3 Female Condom<br>4 Injection<br>5 IUD<br>6 Man had a vasectomy<br>7 Woman had a tubal ligation or hysterectomy<br>8 Implant

		9	Traditional methods (e.g. periodic abstinence, withdrawal, cycle beads)
		998	I don't know
		11	Other
		999	Refused to answer
C.10	C.10 Have you ever heard of vasectomy?	0	No
		1	Yes
		998	I don't know
C.11	C.11 What is your opinion about vasectomy?<br>*PLEASE READ RESPONSE OPTIONS.*<br>*Question relevant when: ${C.10} ='1'*	1	Very positive
		2	Positive
		3	Neither positive nor negative
		4	Negative
		5	Very negative
C.12	C.12 I will now read you a few statements about vasectomy, please let me know if you 'agree' or 'disagree' with each statement.<br>*Question relevant when: ${C.10} ='1'*		
C.12.1	C.12.1 A man still feels sexual pleasure after having a vasectomy.<br>*Question relevant when: ${C.10} ='1'*	0	Disagree
		1	Agree
C.12.2	C.12.2 A woman will still get sexual pleasure after her husband/partner has a vasectomy<br>*Question relevant when: ${C.10} ='1'*	0	Disagree
		1	Agree
C.12.3	C.12.3 If people find out that a man has had a vasectomy, they will make fun of him or think he is less of a man.<br>*Question relevant when: ${C.10} ='1'*	0	Disagree
		1	Agree
N.C.12.4	N.C.12.4 Vasectomy is the best option to avoid pregnancy if a man and his wife/partner do not wish to have any more children<br>*Question relevant when: ${C.10} ='1'*	0	Disagree
		1	Agree
C.13	C.13 Would you consider having a vasectomy as a method of family planning?<br>*Question relevant when: ${C.10} ='1'*	0	No
		1	Yes
		999	Refused to answer
C.17	C.17 I am going to read several statements. Please tell me whether you 'strongly agree', 'agree' 'neither agree nor disagree', 'disagree' or 'strongly disagree' with the statements.		
C.17.1	C.17.1 I don't think it is good to use contraception	1	Strongly agree
		2	Agree
		3	Neither agree nor disagree
		4	Disagree
		5	Strongly disagree
C.17.3	C.17.3 I would feel embarrassed discussing contraception with my wife/partner	1	Strongly agree
		2	Agree
		3	Neither agree nor disagree
		4	Disagree
		5	Strongly disagree
C.17.4	C.17.4 Contraceptives are difficult to obtain	1	Strongly agree
		2	Agree
		3	Neither agree nor disagree
		4	Disagree
		5	Strongly disagree
C.17.5	C.17.5 Contraceptives are embarassing to obtain	1	Strongly agree
		2	Agree
		3	Neither agree nor disagree
		4	Disagree
		5	Strongly disagree
C.17.6	C.17.6 Couples should talk about contraception before having sex	1	Strongly agree
		2	Agree
		3	Neither agree nor disagree
		4	Disagree
		5	Strongly disagree
C.17.7	C.17.7 Two people who are having sex should use some form of contraceptives if they aren't ready for a child	1	Strongly agree
		2	Agree
		3	Neither agree nor disagree

		4	Disagree
		5	Strongly disagree
C.17.8	C.17.8 Contraceptives have bad side effects for women	1	Strongly agree
		2	Agree
		3	Neither agree nor disagree
		4	Disagree
		5	Strongly disagree
C.17.10	C.17.10 Condoms are reliable for preventing pregnancy	1	Strongly agree
		2	Agree
		3	Neither agree nor disagree
		4	Disagree
		5	Strongly disagree
C.17.11	C.17.11 I would feel embarassed to suggest using condoms	1	Strongly agree
		2	Agree
		3	Neither agree nor disagree
		4	Disagree
		5	Strongly disagree
C.17.12	C.17.12 Condoms ruin the sex act	1	Strongly agree
		2	Agree
		3	Neither agree nor disagree
		4	Disagree
		5	Strongly disagree

survey > PART 4: RELATIONSHIPS

Group relevant when: ${A.1} >=18 and ${A.7} ='2' or ${A.7} ='3'

note_d	Now, I will ask you questions about your relationship with your wife/partner. Remember, your responses will be kept completely confidential and will not be told to anyone.		
D.1	D.1 I am going to read you some statements. Please tell me if this happens 'often', 'sometimes', 'rarely', or 'never'...		
D.1.1	D.1.1 When I have problems my partner listens to me	1	Often
		2	Sometimes
		3	Rarely
		4	Never
		998	Don't know
D.1.2	D.1.2 My partner blames me for things that go wrong	1	Often
		2	Sometimes
		3	Rarely
		4	Never
		998	Don't know
D.1.3	D.1.3 I feel appreciated by my partner	1	Often
		2	Sometimes
		3	Rarely
		4	Never
		998	Don't know
D.1.4	D.1.4 I feel respected even if we disagree	1	Often
		2	Sometimes
		3	Rarely
		4	Never
		998	Don't know
D.1.5	D.1.5 We are good at solving our differences	1	Often
		2	Sometimes
		3	Rarely
		4	Never
		998	Don't know
D.1.6	D.1.6 My partner criticizes my opinions, feelings, or desires	1	Often
		2	Sometimes
		3	Rarely
		4	Never
		998	Don't know
D.1.7	D.1.7 My partner shows love and affection for me	1	Often
		2	Sometimes
		3	Rarely

		4	Never
		998	Don't know
D.2	D.2 Now, please tell me if you 'strongly agree', 'agree',' disagree', 'strongly disagree' or 'neither agree nor disagree' with the following statements:		
D.2.1	D.2.1 My partner and I often talk about things that happen to each of us during the day	1	Strongly agree
		2	Agree
		3	Neither agree nor disagree
		4	Disagree
		5	Strongly disagree
D.3	D.3 In general, how often would you say that you and your partner quarrel?<br>*READ RESPONSE OPTIONS*	1	Often
		2	Sometimes
		3	Rarely
		4	Never
		998	Don't know
note_d5	Now I'd like to ask you about your communication with your partner.		
D.5	D.5 How often do you discuss with your partner your household's weekly or monthly income and expenses?	1	Often
		2	Sometimes
		3	Rarely
		4	Never
		997	Not applicable
		999	Refused to answer
D.5.3	D.5.3 Who makes the final decision about your household's weekly or monthly income and expenses?<br>*Question relevant when: ${D.5} !='997'*	1	You
		2	Your partner
		3	Both have the same say
		4	Someone else
		998	Don't know
		999	Refused to answer
D.6	D.6 How often do you discuss with your partner spending money on large investments, such as buying a cow, forest or plot of land?	1	Often
		2	Sometimes
		3	Rarely
		4	Never
		997	Not applicable
		999	Refused to answer
D.6.3	D.6.3 Who makes the final decision about the spending money on large investments, such as buying a cow, forest or plot of land?<br>*Question relevant when: ${D.6} !='997'*	1	You
		2	Your partner
		3	Both have the same say
		4	Someone else
		998	Don't know
		999	Refused to answer
D.7	D.7 How often do you discuss with your partner how many children to have or the spacing of children?	1	Often
		2	Sometimes
		3	Rarely
		4	Never
		997	Not applicable
		999	Refused to answer
D.7.3	D.7.3 Who makes the final decision about how many children to have or the spacing of children?<br>*Question relevant when: ${D.7} !='997'*	1	You
		2	Your partner
		3	Both have the same say
		4	Someone else
		998	Don't know
		999	Refused to answer
D.8	D.8 How often do you discuss with your partner whether she can work outside the home?	1	Often
		2	Sometimes
		3	Rarely
		4	Never
		997	Not applicable
		999	Refused to answer
D.8.3	D.8.3 Who makes the final decision about whether your partner can work outside the home?<br>*Question relevant when: ${D.8} !='997'*	1	You
		2	Your partner

		3	Both have the same say
		4	Someone else
		998	Don't know
		999	Refused to answer
D.9	D.9 How often do you discuss with your partner about how your partner's cash earnings are used ?	1	Often
		2	Sometimes
		3	Rarely
		4	Never
		997	Not applicable
		999	Refused to answer
D.9.3	D.9.3 Who makes the final decision about how your partner's cash earnings are used?<br>*Question relevant when: ${D.9} !='997'*	1	You
		2	Your partner
		3	Both have the same say
		4	Someone else
		998	Don't know
		999	Refused to answer
D.10	D.10 How often do you discuss with your partner about how your (the man's) cash earnings are used?	1	Often
		2	Sometimes
		3	Rarely
		4	Never
		997	Not applicable
		999	Refused to answer
D.10.3	D.10.3 Who makes the final decision about how your (the man's) cash earnings are used?<br>*Question relevant when: ${D.10} !='997'*	1	You
		2	Your partner
		3	Both have the same say
		4	Someone else
		998	Don't know
		999	Refused to answer
D.11	D.11 How often do you discuss with your partner about whether to use contraception or which method to use?	1	Often
		2	Sometimes
		3	Rarely
		4	Never
		997	Not applicable
		999	Refused to answer
D.11.3	D.11.3 Who makes the final decision about whether to use contraception or which method to use?<br>*Question relevant when: ${D.11} !='997'*	1	You
		2	Your partner
		3	Both have the same say
		4	Someone else
		998	Don't know
		999	Refused to answer
D.13	D.13 How often do you discuss with your partner about your children's schooling?	1	Often
		2	Sometimes
		3	Rarely
		4	Never
		997	Not applicable
		999	Refused to answer
D.13.3	D.13.3 Who makes the final decision about your children's schooling?<br>*Question relevant when: ${D.13} !='997'*	1	You
		2	Your partner
		3	Both have the same say
		4	Someone else
		998	Don't know
		999	Refused to answer

survey > PART 5: CAREGIVING

Group relevant when: ${A.1} >=18 and ${A.7} ='2' or ${A.7} ='3'

note_5	Now I would like to ask you some questions about your family and about household tasks.<br>*Question relevant when: ${A.16} !='0'*	
note_5b	I am now going to ask you about how you and your partner divide different childcare tasks.<br>*NOTE: The following questions ask about how a man and his partner divide childcare tasks. In some cases the child may be too young for the activity to be applicable (for example: to go to school) or the activity may be done by someone else in the household (e.g. a maid or other family member). In both cases, put 'not applicable'.*<br>*Question relevant when: ${A.16} !='0'*	

E.12	E.12 If you disregard the outside help you receive from others, how do you and your partner divide the following task: Providing the daily care of your child(ren)<br>*Question relevant when: ${A.16} !='0'*	1 Always you<br>2 Usually you<br>3 Shared equally or done together<br>4 Usually partner<br>5 Always partner<br>997 Not applicable
E.12a	E.12a During the last 7 days, on how many days did you do this task?<br>*Question relevant when: ${A.16} !='0' and ${E.12} !='997'*<br>*Response constrained to: .<=7*	
E.12b	E.12b On a typical day when you do this task, how many hours do you spend doing it?<br>*Please indicate time at 15 minute intervals: for example, if a person might say he spent 15 minutes on a task, mark 0.25 (1 quarter hour). Round any amount up to 15 minutes ; round all other amounts to the closest 15 minute interval (e.g. if 35 minutes, round to 30 minutes, entering 0.5 hours) [enter # hours]*<br>*Question relevant when: ${A.16} !='0' and ${E.12} !='997' and ${E.12a} !='0'*<br>*Response constrained to: .<=24*	
E.13	E.13 If you disregard the outside help you receive from others, how do you and your partner divide the following task: Holding your child/ren when he or she was a newborn<br>*Question relevant when: ${A.16} !='0'*	1 Always you<br>2 Usually you<br>3 Shared equally or done together<br>4 Usually partner<br>5 Always partner<br>997 Not applicable
E.13a	E.13a During the last 7 days, on how many days did you do this task?<br>*[enter # days]*<br>*Question relevant when: ${A.16} !='0' and ${E.13} !='997'*<br>*Response constrained to: .<=7*	
E.13b	E.13b On a typical day when you do this task, how many hours do you spend doing it?<br>*Please indicate time at 15 minute intervals: for example, if a person might say he spent 15 minutes on a task, mark 0.25 (1 quarter hour). Round any amount up to 15 minutes ; round all other amounts to the closest 15 minute interval (e.g. if 35 minutes, round to 30 minutes, entering 0.5 hours) [enter # hours]*<br>*Question relevant when: ${A.16} !='0' and ${E.13} !='997'and ${E.13a} !='0'*<br>*Response constrained to: .<=24*	
E.14	E.14 If you disregard the outside help you receive from others, how do you and your partner divide the following task: Feeding your child/ren<br>*Question relevant when: ${A.16} !='0'*	1 Always you<br>2 Usually you<br>3 Shared equally or done together<br>4 Usually partner<br>5 Always partner<br>997 Not applicable
E.14a	E.14a During the last 7 days, on how many days did you do this task?<br>*[enter # days]*<br>*Question relevant when: ${A.16} !='0' and ${E.14} !='997'*<br>*Response constrained to: .<=7*	
E.14b	E.14b On a typical day when you do this task, how many hours do you spend doing it?<br>*Please indicate time at 15 minute intervals: for example, if a person might say he spent 15 minutes on a task, mark 0.25 (1 quarter hour). Round any amount up to 15 minutes ; round all other amounts to the closest 15 minute interval (e.g. if 35 minutes, round to 30 minutes, entering 0.5 hours) [enter # hours]*<br>*Question relevant when: ${A.16} !='0' and ${E.14} !='997' and ${E.14a} !='0'*<br>*Response constrained to: .<=24*	
E.15	E.15 If you disregard the outside help you receive from others, how do you and your partner divide the following task: Bathing your child/ren<br>*Question relevant when: ${A.16} !='0'*	1 Always you<br>2 Usually you<br>3 Shared equally or done together<br>4 Usually partner<br>5 Always partner<br>997 Not applicable
E.15a	E.15a During the last 7 days, on how many days did you do this task?<br>*[enter # days]*<br>*Question relevant when: ${A.16} !='0' and ${E.15} !='997'*<br>*Response constrained to: .<=7*	
E.15b	E.15b On a typical day when you do this task, how many hours do you spend doing it?<br>*Please indicate time at 15 minute intervals: for example, if a person might say he spent 15 minutes on a task, mark 0.25 (1 quarter hour). Round any amount up to 15 minutes ; round all other amounts to the closest 15 minute interval (e.g. if 35 minutes, round to 30 minutes, entering 0.5 hours) [enter # hours]*<br>*Question relevant when: ${A.16} !='0' and ${E.15} !='997' and ${E.15a} !='0'*	

	Response constrained to: .<=24	
E.16	E.16 If you disregard the outside help you receive from others, how do you and your partner divide the following task:<br>Soothing your child/ren if he or she is crying or upset<br>*Question relevant when: ${A.16} !='0'*	1 Always you<br>2 Usually you<br>3 Shared equally or done together<br>4 Usually partner<br>5 Always partner<br>997 Not applicable
E.16a	E.16a During the last 7 days, on how many days did you do this task?<br>*[enter # days]*<br>*Question relevant when: ${A.16} !='0' and ${E.16} !='997'*<br>*Response constrained to: .<=7*	
E.16b	E.16b On a typical day when you do this task, how many hours do you spend doing it?<br>*Please indicate time at 15 minute intervals: for example, if a person might say he spent 15 minutes on a task, mark 0.25 (1 quarter hour). Round any amount up to 15 minutes ; round all other amounts to the closest 15 minute interval (e.g. if 35 minutes, round to 30 minutes, entering 0.5 hours) [enter # hours]*<br>*Question relevant when: ${A.16} !='0' and ${E.16} !='997' and ${E.16a} !='0'*<br>*Response constrained to: .<=24*	
E.171	E.171 If you disregard the outside help you receive from others, how do you and your partner divide the following task:<br>Taking your child to the health center if sick or for vaccination<br>*Question relevant when: ${A.16} !='0'*	1 Always you<br>2 Usually you<br>3 Shared equally or done together<br>4 Usually partner<br>5 Always partner<br>997 Not applicable
E.171a	E.171a During the last 7 days, on how many days did you do this task?<br>*[enter # days]*<br>*Question relevant when: ${A.16} !='0' and ${E.171} !='997'*<br>*Response constrained to: .<=7*	
E.17	E.17 If you disregard the outside help you receive from others, how do you and your partner divide the following task:<br>Taking and picking up your child/ren from school<br>*Question relevant when: ${A.16} !='0'*	1 Always you<br>2 Usually you<br>3 Shared equally or done together<br>4 Usually partner<br>5 Always partner<br>997 Not applicable
E.17a	E.17a During the last 7 days, on how many days did you do this task?<br>*[enter # days]*<br>*Question relevant when: ${A.16} !='0' and ${E.17} !='997'*<br>*Response constrained to: .<=7*	
E.17b	E.17b On a typical day when you do this task, how many hours do you spend doing it?<br>*Please indicate time at 15 minute intervals: for example, if a person might say he spent 15 minutes on a task, mark 0.25 (1 quarter hour). Round any amount up to 15 minutes ; round all other amounts to the closest 15 minute interval (e.g. if 35 minutes, round to 30 minutes, entering 0.5 hours) [enter # hours]*<br>*Question relevant when: ${A.16} !='0' and ${E.17} !='997' and ${E.17a} !='0'*<br>*Response constrained to: .<=24*	
E.18	E.18 If you disregard the outside help you receive from others, how do you and your partner divide the following task:<br>Telling a story, singing a song, or playing with your child/ren<br>*Question relevant when: ${A.16} !='0'*	1 Always you<br>2 Usually you<br>3 Shared equally or done together<br>4 Usually partner<br>5 Always partner<br>997 Not applicable
E.18a	E.18a During the last 7 days, on how many days did you do this task?<br>*[enter # days]*<br>*Question relevant when: ${A.16} !='0' and ${E.18} !='997'*<br>*Response constrained to: .<=7*	
E.18b	E.18b On a typical day when you do this task, how many hours do you spend doing it?<br>*Please indicate time at 15 minute intervals: for example, if a person might say he spent 15 minutes on a task, mark 0.25 (1 quarter hour). Round any amount up to 15 minutes ; round all other amounts to the closest 15 minute interval (e.g. if 35 minutes, round to 30 minutes, entering 0.5 hours) [enter # hours]*<br>*Question relevant when: ${A.16} !='0' and ${E.18} !='997' and ${E.18a} !='0'*<br>*Response constrained to: .<=24*	
E.19	E.19 If you disregard the outside help you receive from others, how do you and your partner divide the following task:<br>Teaching your child/ren something	1 Always you<br>2 Usually you

	Question relevant when: ${A.16} !='0'	3 Shared equally or done together<br>4 Usually partner<br>5 Always partner<br>997 Not applicable
E.19a	E.19a During the last 7 days, on how many days did you do this task?<br>*[enter # days]*<br>*Question relevant when: ${A.16} !='0' and ${E.19} !='997'*<br>*Response constrained to: .<=7*	
E.19b	E.19b On a typical day when you do this task, how many hours do you spend doing it?<br>*Please indicate time at 15 minute intervals: for example, if a person might say he spent 15 minutes on a task, mark 0.25 (1 quarter hour). Round any amount up to 15 minutes ; round all other amounts to the closest 15 minute interval (e.g. if 35 minutes, round to 30 minutes, entering 0.5 hours) [enter # hours]*<br>*Question relevant when: ${A.16} !='0' and ${E.19} !='997' and ${E.19a} !='0'*<br>*Response constrained to: .<=24*	
E.20	E.20 If you disregard the outside help you receive from others, how do you and your partner divide the following task: Disciplining your child/ren (verbal discipline)<br>*Question relevant when: ${A.16} !='0'*	1 Always you<br>2 Usually you<br>3 Shared equally or done together<br>4 Usually partner<br>5 Always partner<br>997 Not applicable
E.20a	E.20a During the last 7 days, on how many days did you do this task?<br>*[enter # days]*<br>*Question relevant when: ${A.16} !='0' and ${E.20} !='997'*<br>*Response constrained to: .<=7*	
E.21	E.21 If you disregard the outside help you receive from others, how do you and your partner divide the following task: Spanking or beating your child/ren<br>*Question relevant when: ${A.16} !='0'*	1 Always you<br>2 Usually you<br>3 Shared equally or done together<br>4 Usually partner<br>5 Always partner<br>997 Not applicable
E.21a	E.21a During the last 7 days, on how many days did you do this task?<br>*[enter # days]*<br>*Question relevant when: ${A.16} !='0' and ${E.21} !='997'*<br>*Response constrained to: .<=7*	
E.40	E.40 How much involvement would you like to have in taking care of your child (ren)<br>*Question relevant when: ${A.16} !='0'*	1 I am happy with my current involvement<br>2 I'd like to be more involved<br>3 I'd like to be less involved
E.4	E.4 How much do you think your partner wants you to be involved in taking care of your child/ren?<br>*READ RESPONSE OPTIONS.*<br>*Question relevant when: ${A.16} !='0'*	1 She's happy with my current involvement<br>2 She'd like me to be more involved<br>3 She'd like me to be less involved<br>998 I don't know
E.10	E.10 I would like to ask you some questions about raising children. Adults use certain ways to teach children how to behave well and to correct them when they misbehave. Please tell me if you have used any of these methods in the PAST Month with any of your (biological) children.<br>*Question relevant when: ${A.16} !='0'*	
E.10.1	E.10.1 Took away privileges, or forbade something your child did or did not allow your child to leave the house<br>*This question refers to the past MONTH, and with any biological children.*<br>*Question relevant when: ${A.16} !='0'*	0 No<br>1 Yes
E.10.2	E.10.2 Explained why your child's behavior was wrong<br>*This question refers to the past MONTH, and with any biological children.*<br>*Question relevant when: ${A.16} !='0'*	0 No<br>1 Yes
E.10.3	E.10.3 Shook your child<br>*This question refers to the past MONTH, and with any biological children.*<br>*Question relevant when: ${A.16} !='0'*	0 No<br>1 Yes
E.10.4	E.10.4 Shouted, or yelled at or screamed at your child<br>*This question refers to the past MONTH, and with any biological children.*<br>*Question relevant when: ${A.16} !='0'*	0 No<br>1 Yes

E.10.5	E.10.5 Gave your child something else to do<br>*This question refers to the past MONTH, and with any biological children.*<br>*Question relevant when: ${A.16} !='0'*	0 No<br>1 Yes
E.10.6	E.10.6 Spanked, or hit or slapped your child on the bottom with bare hand<br>*This question refers to the past MONTH, and with any biological children.*<br>*Question relevant when: ${A.16} !='0'*	0 No<br>1 Yes
E.10.7	E.10.7 Hit your child on the bottom or elsewhere on the body with something like a belt, or stick or other hard object<br>*This question refers to the past MONTH, and with any biological children.*<br>*Question relevant when: ${A.16} !='0'*	0 No<br>1 Yes
E.10.8	E.10.8 Called your child stupid, or lazy, or another name like that<br>*This question refers to the past MONTH, and with any biological children.*<br>*Question relevant when: ${A.16} !='0'*	0 No<br>1 Yes
E.10.9	E.10.9 Hit or slapped your child on the face, or head or ears<br>*This question refers to the past MONTH, and with any biological children.*<br>*Question relevant when: ${A.16} !='0'*	0 No<br>1 Yes
E.10.10	E.10.10 Hit or slapped your child on the hand, or arm or leg<br>*This question refers to the past MONTH, and with any biological children.*<br>*Question relevant when: ${A.16} !='0'*	0 No<br>1 Yes
E.10.11	E.10.11 Beat your child up, meaning you hit your child over and over as hard as you could<br>*This question refers to the past MONTH, and with any biological children.*<br>*Question relevant when: ${A.16} !='0'*	0 No<br>1 Yes
E.10.12	E.10.12 Made your child kneel on the ground (for a period of time)<br>*This question refers to the past MONTH, and with any biological children.*<br>*Question relevant when: ${A.16} !='0'*	0 No<br>1 Yes
E.10.13	E.10.13 Made your child hold bricks or stones in his/her hands while sitting down, standing or kneeling<br>*This question refers to the past MONTH, and with any biological children.*<br>*Question relevant when: ${A.16} !='0'*	0 No<br>1 Yes
E.11	E.11 I am going to read you a series of statements. Please tell me if you strongly agree, agree, netiher agree nor disagree, disagree or strongly disagree with each of the statements.<br>*Question relevant when: ${A.16} !='0'*	
E.11.1	E.11.1 Children need to be physically punished in order to be raised properly<br>*Question relevant when: ${A.16} !='0'*	1 Strongly agree<br>2 Agree<br>3 Neither agree nor disagree<br>4 Disagree<br>5 Strongly disagree
E.11.2	E.11.2 Physical punishment has negative impacts on children<br>*Question relevant when: ${A.16} !='0'*	1 Strongly agree<br>2 Agree<br>3 Neither agree nor disagree<br>4 Disagree<br>5 Strongly disagree
note_E22	I am now going to ask you about how you and your partner divide different household tasks.<br>*Note: The following questions ask about how a man and his partner divide household tasks. In some cases, the activity may not be relevant for the household or may be done by someone else in the household (e.g. maid or other family member). In this case, please put 'Not Applicable'.*	
E.22	E.22 If you disregard the outside help you receive from others, how do you and your partner divide the following task: Fetching water for the household (to and from the water source)	1 Always you<br>2 Usually you<br>3 Shared equally or done together<br>4 Usually partner<br>5 Always partner<br>997 Not applicable
E.22a	E.22a During the last 7 days, on how many days did you do this task?<br>*[enter # days]*<br>*Question relevant when: ${E.22} !='997'*<br>*Response constrained to: .<=7*	
E.22b	E.22b On a typical day when you do this task, how many hours do you spend doing it?<br>*Please indicate time at 15 minute intervals: for example, if a person might say he spent 15 minutes on a task, mark 0.25 (1 quarter hour). Round any amount up to 15 minutes up to 15 minutes; round all other amounts to the closest 15 minute interval (e.g. if 35 minutes, round to 30 minutes, entering 0.5 hours) [enter # hours]*<br>*Question relevant when: ${E.22} !='997' and ${E.22a} !='0'*<br>*Response constrained to: .<=24*	
E.23	E.23 If you disregard the outside help you receive from others, how do you and your partner divide the following task: Washing clothes/do laundry	1 Always you<br>2 Usually you<br>3 Shared equally or done together<br>4 Usually partner

		5 Always partner<br>997 Not applicable
E.23a	E.23a During the last 7 days, on how many days did you do this task?<br>*[enter # days]*<br>*Question relevant when: ${E.23} !='997'*<br>*Response constrained to: .<=7*	
E.23b	E.23b On a typical day when you do this task, how many hours do you spend doing it?<br>*Please indicate time at 15 minute intervals: for example, if a person might say he spent 15 minutes on a task, mark 0.25 (1 quarter hour). Round any amount up to 15 minutes up to 15 minutes; round all other amounts to the closest 15 minute interval (e.g. if 35 minutes, round to 30 minutes, entering 0.5 hours) [enter # hours]*<br>*Question relevant when: ${E.23} !='997' and ${E.23a} !='0'*<br>*Response constrained to: .<=24*	
E.24	E.24 If you disregard the outside help you receive from others, how do you and your partner divide the following task:<br>Going to the market for the household for shopping	1 Always you<br>2 Usually you<br>3 Shared equally or done together<br>4 Usually partner<br>5 Always partner<br>997 Not applicable
E.24a	E.24a During the last 7 days, on how many days did you do this task?<br>*[enter # days]*<br>*Question relevant when: ${E.24} !='997'*<br>*Response constrained to: .<=7*	
E.24b	E.24b On a typical day when you do this task, how many hours do you spend doing it?<br>*Please indicate time at 15 minute intervals: for example, if a person might say he spent 15 minutes on a task, mark 0.25 (1 quarter hour). Round any amount up to 15 minutes up to 15 minutes; round all other amounts to the closest 15 minute interval (e.g. if 35 minutes, round to 30 minutes, entering 0.5 hours) [enter # hours]*<br>*Question relevant when: ${E.24} !='997' and ${E.24a} !='0'*<br>*Response constrained to: .<=24*	
E.25	E.25 If you disregard the outside help you receive from others, how do you and your partner divide the following task:<br>Cleaning the house and surroundings	1 Always you<br>2 Usually you<br>3 Shared equally or done together<br>4 Usually partner<br>5 Always partner<br>997 Not applicable
E.25a	E.25a During the last 7 days, on how many days did you do this task?<br>*[enter # days]*<br>*Question relevant when: ${E.25} !='997'*<br>*Response constrained to: .<=7*	
E.25b	E.25b On a typical day when you do this task, how many hours do you spend doing it?<br>*Please indicate time at 15 minute intervals: for example, if a person might say he spent 15 minutes on a task, mark 0.25 (1 quarter hour). Round any amount up to 15 minutes up to 15 minutes; round all other amounts to the closest 15 minute interval (e.g. if 35 minutes, round to 30 minutes, entering 0.5 hours) [enter # hours]*<br>*Question relevant when: ${E.25} !='997' and ${E.25a} !='0'*<br>*Response constrained to: .<=24*	
E.26	E.26 If you disregard the outside help you receive from others, how do you and your partner divide the following task:<br>Cleaning the bathroom or toilet	1 Always you<br>2 Usually you<br>3 Shared equally or done together<br>4 Usually partner<br>5 Always partner<br>997 Not applicable
E.26a	E.26a During the last 7 days, on how many days did you do this task?<br>*[enter # days]*<br>*Question relevant when: ${E.26} !='997'*<br>*Response constrained to: .<=7*	
E.26b	E.26b On a typical day when you do this task, how many hours do you spend doing it?<br>*Please indicate time at 15 minute intervals: for example, if a person might say he spent 15 minutes on a task, mark 0.25 (1 quarter hour). Round any amount up to 15 minutes up to 15 minutes; round all other amounts to the closest 15 minute interval (e.g. if 35 minutes, round to 30 minutes, entering 0.5 hours) [enter # hours]*<br>*Question relevant when: ${E.26} !='997' and ${E.26a} !='0'*<br>*Response constrained to: .<=24*	
E.27	E.27 If you disregard the outside help you receive from others, how do you and your partner divide the following task:<br>Cooking for the household	1 Always you<br>2 Usually you<br>3 Shared equally or done together

		4 Usually partner<br>5 Always partner<br>997 Not applicable
E.27a	E.27a During the last 7 days, on how many days did you do this task?<br>*[enter # days]*<br>*Question relevant when: ${E.27} !='997'*<br>*Response constrained to: .<=7*	
E.27b	E.27b On a typical day when you do this task, how many hours do you spend doing it?<br>*Please indicate time at 15 minute intervals: for example, if a person might say he spent 15 minutes on a task, mark 0.25 (1 quarter hour). Round any amount up to 15 minutes up to 15 minutes; round all other amounts to the closest 15 minute interval (e.g. if 35 minutes, round to 30 minutes, entering 0.5 hours) [enter # hours]*<br>*Question relevant when: ${E.27} !='997' and ${E.27a} !='0'*<br>*Response constrained to: .<=24*	
E.28	E.28 If you disregard the outside help you receive from others, how do you and your partner divide the following task: Managing the weekly budget?	1 Always you<br>2 Usually you<br>3 Shared equally or done together<br>4 Usually partner<br>5 Always partner<br>997 Not applicable
E.28a	E.28a During the last 7 days, on how many days did you do this task?<br>*[enter # days]*<br>*Question relevant when: ${E.28} !='997'*<br>*Response constrained to: .<=7*	
E.28b	E.28b On a typical day when you do this task, how many hours do you spend doing it?<br>*Please indicate time at 15 minute intervals: for example, if a person might say he spent 15 minutes on a task, mark 0.25 (1 quarter hour). Round any amount up to 15 minutes up to 15 minutes; round all other amounts to the closest 15 minute interval (e.g. if 35 minutes, round to 30 minutes, entering 0.5 hours) [enter # hours]*<br>*Question relevant when: ${E.28} !='997' and ${E.28a} !='0'*<br>*Response constrained to: .<=24*	
E.29	E.29 If you disregard the outside help you receive from others, how do you and your partner divide the following task: Foraging for firewood for the household	1 Always you<br>2 Usually you<br>3 Shared equally or done together<br>4 Usually partner<br>5 Always partner<br>997 Not applicable
E.29a	E.29a During the last 7 days, on how many days did you do this task?<br>*[enter # days]*<br>*Question relevant when: ${E.29} !='997'*<br>*Response constrained to: .<=7*	
E.29b	E.29b On a typical day when you do this task, how many hours do you spend doing it?<br>*Please indicate time at 15 minute intervals: for example, if a person might say he spent 15 minutes on a task, mark 0.25 (1 quarter hour). Round any amount up to 15 minutes up to 15 minutes; round all other amounts to the closest 15 minute interval (e.g. if 35 minutes, round to 30 minutes, entering 0.5 hours) [enter # hours]*<br>*Question relevant when: ${E.29} !='997' and ${E.29a} !='0'*<br>*Response constrained to: .<=24*	
E.30	E.30 If you disregard the outside help you receive from others, how do you and your partner divide the following task: Searching for fodder or grazing for the household’s animals?	1 Always you<br>2 Usually you<br>3 Shared equally or done together<br>4 Usually partner<br>5 Always partner<br>997 Not applicable
E.30a	E.30a During the last 7 days, on how many days did you do this task?<br>*[enter # days]*<br>*Question relevant when: ${E.30} !='997'*<br>*Response constrained to: .<=7*	
E.30b	E.30b On a typical day when you do this task, how many hours do you spend doing it?<br>*Please indicate time at 15 minute intervals: for example, if a person might say he spent 15 minutes on a task, mark 0.25 (1 quarter hour). Round any amount up to 15 minutes up to 15 minutes; round all other amounts to the closest 15 minute interval (e.g. if 35 minutes, round to 30 minutes, entering 0.5 hours) [enter # hours]*<br>*Question relevant when: ${E.30} !='997' and ${E.30a} !='0'*<br>*Response constrained to: .<=24*	
E.31	E.31 If you disregard the outside help you receive from others, how do you and your partner divide the following task: Carrying out any agricultural activity, whether farming, livestock, fishing, or forestry for salary, wages, or in-kind compensation?	1 Always you<br>2 Usually you<br>3 Shared equally or done

		together<br>4 Usually partner<br>5 Always partner<br>997 Not applicable
E.31a	E.31a During the last 7 days, on how many days did you do this task?<br>*[enter # days]*<br>*Question relevant when: ${E.31} !='997'*<br>*Response constrained to: .<=7*	
E.31b	E.31b On a typical day when you do this task, how many hours do you spend doing it?<br>*Please indicate time at 15 minute intervals: for example, if a person might say he spent 15 minutes on a task, mark 0.25 (1 quarter hour). Round any amount up to 15 minutes up to 15 minutes; round all other amounts to the closest 15 minute interval (e.g. if 35 minutes, round to 30 minutes, entering 0.5 hours) [enter # hours]*<br>*Question relevant when: ${E.31} !='997' and ${E.31a} !='0'*<br>*Response constrained to: .<=24*	
E.32	E.32 If you disregard the outside help you receive from others, how do you and your partner divide the following task:<br>Carrying out any agricultural activity, whether farming, livestock, fishing, or forestry for no pay	1 Always you<br>2 Usually you<br>3 Shared equally or done together<br>4 Usually partner<br>5 Always partner<br>997 Not applicable
E.32a	E.32a During the last 7 days, on how many days did you do this task?<br>*[enter # days]*<br>*Question relevant when: ${E.32} !='997'*<br>*Response constrained to: .<=7*	
E.32b	E.32b On a typical day when you do this task, how many hours do you spend doing it?<br>*Please indicate time at 15 minute intervals: for example, if a person might say he spent 15 minutes on a task, mark 0.25 (1 quarter hour). Round any amount up to 15 minutes up to 15 minutes; round all other amounts to the closest 15 minute interval (e.g. if 35 minutes, round to 30 minutes, entering 0.5 hours) [enter # hours]*<br>*Question relevant when: ${E.32} !='997' and ${E.32a} !='0'*<br>*Response constrained to: .<=24*	
E.33	E.33 If you disregard the outside help you receive from others, how do you and your partner divide the following task:<br>Operating a non-farm business for cash or profit for yourself, like a small shop or other income generating activity	1 Always you<br>2 Usually you<br>3 Shared equally or done together<br>4 Usually partner<br>5 Always partner<br>997 Not applicable
E.33a	E.33a During the last 7 days, on how many days did you do this task?<br>*[enter # days]*<br>*Question relevant when: ${E.33} !='997'*<br>*Response constrained to: .<=7*	
E.33b	E.33b On a typical day when you do this task, how many hours do you spend doing it?<br>*Please indicate time at 15 minute intervals: for example, if a person might say he spent 15 minutes on a task, mark 0.25 (1 quarter hour). Round any amount up to 15 minutes up to 15 minutes; round all other amounts to the closest 15 minute interval (e.g. if 35 minutes, round to 30 minutes, entering 0.5 hours) [enter # hours]*<br>*Question relevant when: ${E.33} !='997' and ${E.33a} !='0'*<br>*Response constrained to: .<=24*	
E.34	E.34 If you disregard the outside help you receive from others, how do you and your partner divide the following task:<br>Working in a non-farm business belonging to a family member not for pay	1 Always you<br>2 Usually you<br>3 Shared equally or done together<br>4 Usually partner<br>5 Always partner<br>997 Not applicable
E.34a	E.34a During the last 7 days, on how many days did you do this task?<br>*[enter # days]*<br>*Question relevant when: ${E.34} !='997'*<br>*Response constrained to: .<=7*	
E.34b	E.34b On a typical day when you do this task, how many hours do you spend doing it?<br>*Please indicate time at 15 minute intervals: for example, if a person might say he spent 15 minutes on a task, mark 0.25 (1 quarter hour). Round any amount up to 15 minutes up to 15 minutes; round all other amounts to the closest 15 minute interval (e.g. if 35 minutes, round to 30 minutes, entering 0.5 hours) [enter # hours]*<br>*Question relevant when: ${E.34} !='997' and ${E.34a} !='0'*<br>*Response constrained to: .<=24*	
E.35	E.35 If you disregard the outside help you receive from others, how do you and your partner divide the following task:<br>Repairing the house	1 Always you<br>2 Usually you

		3 Shared equally or done together<br>4 Usually partner<br>5 Always partner<br>997 Not applicable
E.35a	E.35a During the last 7 days, on how many days did you do this task?<br>*[enter # days]*<br>*Question relevant when: ${E.35} !='997'*<br>*Response constrained to: .<=7*	
E.35b	E.35b On a typical day when you do this task, how many hours do you spend doing it?<br>*Please indicate time at 15 minute intervals: for example, if a person might say he spent 15 minutes on a task, mark 0.25 (1 quarter hour). Round any amount up to 15 minutes up to 15 minutes; round all other amounts to the closest 15 minute interval (e.g. if 35 minutes, round to 30 minutes, entering 0.5 hours) [enter # hours]*<br>*Question relevant when: ${E.35} !='997' and ${E.35a} !='0'*<br>*Response constrained to: .<=24*	
E.36	E.36 If you disregard the outside help you receive from others, how do you and your partner divide the following task: Making the bed	1 Always you<br>2 Usually you<br>3 Shared equally or done together<br>4 Usually partner<br>5 Always partner<br>997 Not applicable
E.36a	E.36a During the last 7 days, on how many days did you do this task?<br>*[enter # days]*<br>*Question relevant when: ${E.36} !='997'*<br>*Response constrained to: .<=7*	
E.36b	E.36b On a typical day when you do this task, how many hours do you spend doing it?<br>*Please indicate time at 15 minute intervals: for example, if a person might say he spent 15 minutes on a task, mark 0.25 (1 quarter hour). Round any amount up to 15 minutes up to 15 minutes; round all other amounts to the closest 15 minute interval (e.g. if 35 minutes, round to 30 minutes, entering 0.5 hours) [enter # hours]*<br>*Question relevant when: ${E.36} !='997' and ${E.36a} !='0'*<br>*Response constrained to: .<=24*	
E.37	E.37 During the last 7 days, on how many days did you spend social time with family or friends?<br>*[enter # days]*<br>*Response constrained to: .<=7*	
E.37a	E.37a On a typical day when you did this task, how many hours do you spend doing it?<br>*Please indicate time at 15 minute intervals: for example, if a person might say he spent 15 minutes on a task, mark 0.25 (1 quarter hour). Round any amount up to 15 minutes up to 15 minutes; round all other amounts to the closest 15 minute interval (e.g. if 35 minutes, round to 30 minutes, entering 0.5 hours) [enter # hours]*<br>*Question relevant when: ${E.37} !='0'*<br>*Response constrained to: .<=24*	
E.38	E.38 During the last 7 days, on average, how many hours did you sleep per day?<br>*Please indicate time at 15 minute intervals: for example, if a person might say he spent 15 minutes on a task, mark 0.25 (1 quarter hour). Round any amount up to 15 minutes up to 15 minutes; round all other amounts to the closest 15 minute interval (e.g. if 35 minutes, round to 30 minutes, entering 0.5 hours) [enter # hours]*<br>*Response constrained to: .<=24*	
E.39	E.39 How much involvement would you like to have in household tasks?<br>*READ RESPONSE OPTIONS.*	1 I am happy with my current involvement<br>2 I'd like to be more involved<br>3 I'd like to be less involved
E.8	E.8 How much do you think your partner wants you to be involved in household tasks?<br>*READ RESPONSE OPTIONS.*	1 She's happy with my current involvement<br>2 She'd like me to be more involved<br>3 She'd like me to be less involved<br>998 I don't know

survey > PART 6: ALCOHOL

Group relevant when: ${A.1} >=18 and ${A.7} ='2' or ${A.7} ='3'

note_6	Now I would like to ask you some questions about alcohol. Please remember that all responses will be kept confidential. If you do not want to answer a question, tell me and I will go on to the next question.	
N.F.1	N.F.1 In the past year (since the last interview), how many times have you had a drink containing alcohol?<br>*READ RESPONSE OPTIONS*	0 Never<br>1 A few times in the past year<br>2 Once every 2 months<br>3 Once a month<br>4 A couple times a month

		5	Once or twice a week
		6	Every day or almost every day
		998	Do not know
		999	Refused to answer
N.F.2	N.F.2 In the past year (since the last interview), how often did you drink so much that you got drunk?<br>*READ RESPONSE OPTIONS*<br>*Question relevant when: ${N.F.1} !='0'*	0	Never (drinks but does not get drunk)
		1	A few times in the past year
		2	Once every 2 months
		3	Once a month
		4	A couple times a month
		5	Once or twice a week
		6	Every day or almost every day
		998	Do not know
		999	Refused to answer
F.3	F.3 In the past year (since the last interview), how often have you…<br>*Question relevant when: ${N.F.1} !='0'*		
F.3.1	F.3.1 Had 5 or more drinks on one occasion?<br>*Question relevant when: ${N.F.1} !='0'*	1	Often
		2	Sometimes
		3	Rarely
		0	Never
		999	Refused to answer
F.3.2	F.3.2 Failed to do what was normally expected of you because of drinking?<br>*Question relevant when: ${N.F.1} !='0'*	1	Often
		2	Sometimes
		3	Rarely
		0	Never
		999	Refused to answer
F.3.3.	F.3.3. Had a feeling of guilt or remorse after drinking?<br>*Question relevant when: ${N.F.1} !='0'*	1	Often
		2	Sometimes
		3	Rarely
		0	Never
		999	Refused to answer
F.4	F.4 Have you or someone else ever been injured because of your drinking?<br>*Question relevant when: ${N.F.1} !='0'*	0	No
		1	Yes
		998	Don't know
		999	Refused to answer
survey > PART 7: VIOLENCE<br>*Group relevant when: ${A.1} >=18 and ${A.7} ='2' or ${A.7} ='3'*			
note_7	Now, I'd like to ask you some questions about violence in your community. Remember that everything you tell me will be kept confidential.		
N.G.1	N.G.1 If a husband beats up his wife, do you think others outside the couple should intervene?	0	No
		1	Yes
		999	Refused to answer
N.G.2	N.G.2 In your opinion, does a man have a good reason to hit his wife if:		
N.G.2.1	N.G.2.1 In your opinion, does a man have a good reason to hit his wife if: She disobeys him	0	No
		1	Yes
		999	Refused to answer
N.G.2.2	N.G.2.2 In your opinion, does a man have a good reason to hit his wife if: She refuses to have sex with him	0	No
		1	Yes
		999	Refused to answer
N.G.2.3	N.G.2.3 In your opinion, does a man have a good reason to hit his wife if: He finds out that she has been unfaithful	0	No
		1	Yes
		999	Refused to answer
N.G.2.4	N.G.2.4 In your opinion, does a man have a good reason to hit his wife if: She neglects taking care of the children	0	No
		1	Yes
		999	Refused to answer
N.G.2.5	N.G.2.5 In your opinion, does a man have a good reason to hit his wife if: She does not complete the household work to his satisfaction	0	No
		1	Yes
		999	Refused to answer

N.G.2.6	N.G.2.6 In your opinion, does a man have a good reason to hit his wife if: She accuses him of being unfaithful	0 No<br>1 Yes<br>999 Refused to answer
N.G.2.7	N.G.2.7 In your opinion, does a man have a good reason to hit his wife if: She spends money without consulting him	0 No<br>1 Yes<br>999 Refused to answer
N.G.2.8	N.G.2.8 In your opinion, does a man have a good reason to hit his wife if: She does not welcome guests in their home to his satisfaction	0 No<br>1 Yes<br>999 Refused to answer
N.G.2.9	N.G.2.9 In your opinion, does a man have a good reason to hit his wife if: When he finds that she did not have food prepared.	0 No<br>1 Yes<br>999 Refused to answer
G.3	G.3 Do you have a male friend or neighbor who uses physical violence against his wife/partner?	0 No<br>1 Yes<br>998 Don't know<br>999 Refused to answer
G.4	G.4 Would you be capable of questioning or challenging his behavior?<br>*PLEASE READ RESPONSE OPTIONS.*<br>*Question relevant when: ${G.3} ='1'*	0 No<br>1 Yes, I would<br>2 Yes, I already have<br>999 Refused to answer
G.5	G.5 What did you do when you saw a male friend or neighbor use violence against a woman?<br>*Question relevant when: ${G.3} ='1' and ${G.4} ='2'*	1 Intervened during the episode<br>2 Spoke to him after the episode<br>3 Avoided/shunned the guy<br>4 Called the police<br>5 Called the local authorities<br>6 Mobilized the neighbors<br>7 Other<br>999 Refused to answer
N.G.3	N.G.3 If a married woman has been beaten up by her husband, do you think it is okay for her to tell others?	0 No<br>1 Yes
G.6	G.6 Are there any laws in your country about gender-based violence?	0 No<br>1 Yes<br>998 I don't know
G.7	G.7 I am going to read some statements about laws about gender-based violence. Please tell me if you 'strongly agree', 'agree', 'neither agree nor disagree', 'disagree' or 'strongly disagree'.<br>*Question relevant when: ${G.6} ='1'*	
G.7.1	G.7.1 The laws make it too easy for a woman to bring a violence charge against a man.<br>*Question relevant when: ${G.6} ='1'*	1 Strongly agree<br>2 Agree<br>3 Neither agree nor disagree<br>4 Disagree<br>5 Strongly disagree<br>999 Refused to answer
G.7.2	G.7.2 The laws are too harsh.<br>*Question relevant when: ${G.6} ='1'*	1 Strongly agree<br>2 Agree<br>3 Neither agree nor disagree<br>4 Disagree<br>5 Strongly disagree<br>999 Refused to answer
G.7.3	G.7.3 The laws are not harsh enough.<br>*Question relevant when: ${G.6} ='1'*	1 Strongly agree<br>2 Agree<br>3 Neither agree nor disagree<br>4 Disagree<br>5 Strongly disagree<br>999 Refused to answer
G.7.4	G.7.4 The laws provide enough protection for the victim of violence.<br>*Question relevant when: ${G.6} ='1'*	1 Strongly agree<br>2 Agree<br>3 Neither agree nor disagree

		4	Disagree
		5	Strongly disagree
		999	Refused to answer
G.7.5	G.7.5 The laws also protect men.<br>*Question relevant when: ${G.6} ='1'*	1	Strongly agree
		2	Agree
		3	Neither agree nor disagree
		4	Disagree
		5	Strongly disagree
		999	Refused to answer
G.7.6	G.7.6 The laws contribute to conflict in the family.<br>*Question relevant when: ${G.6} ='1'*	1	Strongly agree
		2	Agree
		3	Neither agree nor disagree
		4	Disagree
		5	Strongly disagree
		999	Refused to answer
G.7.7	G.7.7 Women are using the laws to dominate men.<br>*Question relevant when: ${G.6} ='1'*	1	Strongly agree
		2	Agree
		3	Neither agree nor disagree
		4	Disagree
		5	Strongly disagree
		999	Refused to answer
survey > PART 8. GENDER ATTITUDES<br>*Group relevant when: ${A.1} >=18 and ${A.7} ='2' or ${A.7} ='3'*			
note_8	The next set of questions will ask you about your views on relations between men and women. Please tell me if you strongly agree', 'agree' 'neither agree nor disagree', 'disagree' or 'strongly disagree' with the following statements.		
H.1	H.1 A woman's most important role is to take care of her home and cook for her family.	1	Strongly agree
		2	Agree
		3	Neither agree nor disagree
		4	Disagree
		5	Strongly disagree
		999	Refused to answer
H.2	H.2 Changing, bathing and feeding the kids are the mother's responsibility.	1	Strongly agree
		2	Agree
		3	Neither agree nor disagree
		4	Disagree
		5	Strongly disagree
		999	Refused to answer
H.4	H.4 A man should have the final word about decisions in his home.	1	Strongly agree
		2	Agree
		3	Neither agree nor disagree
		4	Disagree
		5	Strongly disagree
		999	Refused to answer
H.5	H.5 A man should be respected as the head of the household.	1	Strongly agree
		2	Agree
		3	Neither agree nor disagree
		4	Disagree
		5	Strongly disagree
		999	Refused to answer
H.10	H.10 A good woman never questions her husband's decisions, even if she disagrees with them.	1	Strongly agree
		2	Agree
		3	Neither agree nor disagree
		4	Disagree
		5	Strongly disagree
		999	Refused to answer
H.11	H.11 It is a woman's responsibility to avoid getting pregnant.	1	Strongly agree
		2	Agree
		3	Neither agree nor disagree
		4	Disagree
		5	Strongly disagree

		999	Refused to answer
H.13	H.13 It is perfectly acceptable for women to work outside the home to help support the family economically.	1	Strongly agree
		2	Agree
		3	Neither agree nor disagree
		4	Disagree
		5	Strongly disagree
		999	Refused to answer
H.16	H.16 A woman who carries a condom on her is a “prostitute."	1	Strongly agree
		2	Agree
		3	Neither agree nor disagree
		4	Disagree
		5	Strongly disagree
		999	Refused to answer
H.20	H.20 It is natural and right that men have more power than women in the family.	1	Strongly agree
		2	Agree
		3	Neither agree nor disagree
		4	Disagree
		5	Strongly disagree
		999	Refused to answer
H.21	H.21 Sexual violence (rape) does exist within married couples.	1	Strongly agree
		2	Agree
		3	Neither agree nor disagree
		4	Disagree
		5	Strongly disagree
		999	Refused to answer
H.22	H.22 Sometimes a woman deserves to be beaten.	1	Strongly agree
		2	Agree
		3	Neither agree nor disagree
		4	Disagree
		5	Strongly disagree
		999	Refused to answer
N.H.3	N.H.3 If a man cooks or cleans, it is shameful for his wife.	1	Strongly agree
		2	Agree
		3	Neither agree nor disagree
		4	Disagree
		5	Strongly disagree
		999	Refused to answer
H.24	H.24 A man must make the final decision on how money is spent in the family.	1	Strongly agree
		2	Agree
		3	Neither agree nor disagree
		4	Disagree
		5	Strongly disagree
		999	Refused to answer
H.25	H.25 A woman should tolerate violence in order to keep her family together.	1	Strongly agree
		2	Agree
		3	Neither agree nor disagree
		4	Disagree
		5	Strongly disagree
		999	Refused to answer
H.26	H.26 If money for schooling is scarce, it is better to spend it on boys first.	1	Strongly agree
		2	Agree
		3	Neither agree nor disagree
		4	Disagree
		5	Strongly disagree
		999	Refused to answer
H.27	H.27 If children do wrong they should be beaten.	1	Strongly agree
		2	Agree
		3	Neither agree nor disagree
		4	Disagree
		5	Strongly disagree

		999	Refused to answer
H.29	H.29 Men can take care of children just as well as women can.	1	Strongly agree
		2	Agree
		3	Neither agree nor disagree
		4	Disagree
		5	Strongly disagree
		999	Refused to answer
H.30	H.30 A woman must tolerate all the challenges she faces in her household (that's the way of things)	1	Strongly agree
		2	Agree
		3	Neither agree nor disagree
		4	Disagree
		5	Strongly disagree
		999	Refused to answer
N.H.1	N.H.1 If a husband tells his friends that he makes joint decisions with his wife his friends would not respect him.	1	Strongly agree
		2	Agree
		3	Neither agree nor disagree
		4	Disagree
		5	Strongly disagree
		999	Refused to answer
N.H.2	N.H.2 A man who is seen cooking or cleaning his house will be ridiculed by others	1	Strongly agree
		2	Agree
		3	Neither agree nor disagree
		4	Disagree
		5	Strongly disagree
		999	Refused to answer

survey > PART 9. Campaigns and Support Networks

Group relevant when: ${A.1} >=18 and ${A.7} ='2' or ${A.7} ='3'

note_9	We are almost to the end of the survey. Thank you for answering the questions so far.		
I.9	I.9 I am going to read a series of statements, please tell me if you strongly agree, agree, neither agree nor disagree, disagree, or strongly disagree.		
I.9.1	I.9.1 I am a role-model in my community	1	Strongly agree
		2	Agree
		3	Neither agree nor disagree
		4	Disagree
		5	Strongly disagree
I.9.2	I.9.2 I feel that my life is of use to others	1	Strongly agree
		2	Agree
		3	Neither agree nor disagree
		4	Disagree
		5	Strongly disagree
I.9.3	I.9.3 I have a lot to be happy about	1	Strongly agree
		2	Agree
		3	Neither agree nor disagree
		4	Disagree
		5	Strongly disagree
N.I.9.4	N.I.9.4 I feel that I am a good husband/partner	1	Strongly agree
		2	Agree
		3	Neither agree nor disagree
		4	Disagree
		5	Strongly disagree
N.I.9.5	N.I.9.5 I feel that I am a good father<br>*Question relevant when: ${partcipant_group} ='2'*	1	Strongly agree
		2	Agree
		3	Neither agree nor disagree
		4	Disagree
		5	Strongly disagree
I.1	I.1 In the past year (since the last interview), have you participated in any activity in your community or workplace to talk about men's use of violence against women?<br>*Question relevant when: ${partcipant_group} ='2'*	0	No
		1	Yes
		999	Refused to answer
I.2	I.2 In the past year (since the last interview), have you participated in any activity in your community or workplace to talk about men's role in their children's lives?	0	No
		1	Yes

	Question relevant when: ${partcipant_group} ='2'	
N.I.1	N.I.1 In the past year (since the last interview), have you participated in any activity in your community or workplace to talk about maternal health?<br>*Question relevant when: ${partcipant_group} ='2'*	0 No<br>1 Yes
I.7	I.7 Do you know anyone who has participated in or facilitated a Bandebereho group?<br>*Question relevant when: ${partcipant_group} ='2'*	0 No<br>1 Yes, one person<br>2 Yes, multiple people<br>998 I don't know
I.8	I.8 What is this person or persons' relationship to you?<br>*MARK ALL THAT APPLY.*<br>*Question relevant when: ${partcipant_group} ='2' and ${I.7} !='0' and ${I.7} !='998'*	1 Close family member<br>2 Close friend<br>3 Distant relative<br>4 Acquaintance/someone you don't know very well<br>5 Neighbor<br>6 Other
I.8.1	I.8.1 Have you ever sought advice from one of RWAMREC's Bandebereho facilitators or group members?<br>*Question relevant when: ${partcipant_group} ='2' and ${I.7} !='0' and ${I.7} !='998'*	0 No<br>1 Yes<br>998 I don't know
N.I.2	N.I.2 How many times have you sought advice from one of RWAMREC's Bandebereho facilitatators or group members?<br>*Question relevant when: ${partcipant_group} ='2' and ${I.7} !='0' and ${I.8.1} ='1' and ${I.7} !='998'*	1 Once<br>2 A few times (2-5 times)<br>3 Many times (more than 5 times)
note_NI3	I'm going to ask you a few questions about activities you have participated in during the past year (since the time of the last interview).<br>*Question relevant when: ${partcipant_group} ='1'*	
N.I.3	N.I.3 In the past year (since the last interview), did you participate in any activity, other than the RWAMREC Bandebereho parents' sessions, which talked about men's use of violence against women?<br>*Question relevant when: ${partcipant_group} ='1'*	0 No<br>1 Yes
N.I.4	N.I.4 In the past year (since the last interview), did you participate in any activity, other than the RWAMREC Bandebereho parents' sessions, which talked about men's role in their children's lives?<br>*Question relevant when: ${partcipant_group} ='1'*	0 No<br>1 Yes
N.I.5	N.I.5 In the past year (since the last interview), did you participate in any activity, other than the RWAMREC Bandebereho parents' sessions, which talked about maternal health?<br>*Question relevant when: ${partcipant_group} ='1'*	0 No<br>1 Yes
N.I.10	N.I.10 Have you shared what you discussed in the RWAMREC Bandebereho parents' sessions with anyone else?<br>*Question relevant when: ${partcipant_group} ='1'*	0 No<br>1 Yes
N.I.11	N.I.11 If yes, with whom did you share what you learned?<br>*CHECK ALL THAT APPLY*<br>*Question relevant when: ${partcipant_group} ='1' and ${N.I.10} ='1'*	1 My partner<br>2 My child(ren)<br>3 Close family member<br>4 Close friend<br>5 Distant relative<br>6 Acquaintance/someone I don't know very well<br>7 Neighbor<br>8 The public (e.g. gave testimony at umuganda)
I.13	I.13 Over the past year and a half, you have participated in our interviews up to 3 times. I have asked you some easy and some difficult questions. How has talking about these things made you feel?	1 Good<br>2 Bad<br>3 Neither good nor bad
end_note	We have now reached the end of the survey.<br><br>Thank you very much for taking the time to answer our questions.<br><br>Please know that all the responses will be kept confidential, and cannot be linked back to your name. ASK THE PARTICIPANT IF HE WANTS THE REFERRAL SHEET OF SERVICES/ SUPPORT ORGANIZATIONS IN HIS SECTOR. PROVIDE IT TO THE RESPONDENT IF HE WANTS IT.	
