# Supplementary material for: Gender-transformative Bandebereho couples’ intervention to promote male engagement in reproductive and maternal health and violence prevention in Rwanda: Findings from a randomized controlled trial
Source: PLoS One. 2018 Apr 4;13(4):e0192756. doi: 10.1371/journal.pone.0192756 (PMC5884496; doi:10.1371/journal.pone.0192756)
Supplement: S4 File — (PDF) [file pone.0192756.s004.pdf]

Bandebereho_21_Followup_Male_survey_Final

Field	Question	Answer
survey		
survey > coversheet		
partcipant_group	UMUKARANI W'IBARURA: Genzura ku rutonde rw'abitabira hanyuma wemeze group aherereyemo	1 Treatment group<br>2 Control group
survey > Igice cya 1: Umwirondoro w'ubazwa n'ibindi biranga urugo rwe		
A.1	A.1 Ufite imyaka ingahe?<br>*Iniba usubiza atazi imyaka ye andika 998, niba adashaka gusubiza andika 999 [Enter age]*<br>*Response constrained to: .<=60 or .=998 or .=999*	
A.2	A.2 Ni uruhe rwego rw'amashuri wize?<br>*Question relevant when: ${A.1} >=18*	0 Ntayo<br>1 Nacikije amashuri abanza<br>2 Narangije amashuri abanza<br>3 Nacikije amashuri yisumbuye<br>4 Narangije amashuri yisumbuye<br>5 Amashuri y'imyuga/TTC<br>6 Kaminuza<br>999 Yanze gusubiza
A.4	A.4 Ni uwuhe murimo w'ingezi ukora?<br>*Question relevant when: ${A.1} >=18*	1 Mfite akazi kampemba<br>2 Ndikorera<br>3 Nta kazi mfite, ndi kugashaka<br>4 Nta kazi mfite, nta n'ako nshaka<br>5 Sinshobora gukora<br>999 Yanze gusubiza
survey > Igice cya 1: Umwirondoro w'ubazwa n'ibindi biranga urugo rwe > afford_item<br>*Group relevant when: ${A.1} >=18*		
A.5	A.5 Ni kangahe umuryango wawe ubona mu buryo buhagije ibi bikurikira?<br>*Consider the frequency that the participant is unable to meet any of the needs listed (not all needs). For example, if he always has shelter but he is only able to afford food often, select "often." If he is able to afford food only sometimes, select sometimes. MUSOMERE IBISUBIZO BYOSE BYATANZWE:*	1 Nta na rimwe<br>2 Rimwe na rimwe<br>3 Kenshi<br>4 Buri gihe
A.5.1	A.5.1 Ibyangombwa nkenerwa (ibiribwa cyangwa icumbi…)	1 Nta na rimwe<br>2 Rimwe na rimwe<br>3 Kenshi<br>4 Buri gihe
A.5.2	A.5.2. Ibindi nkenerwa (imyambaro cyangwa amafaranga, y'ishuri, cyangwa mitiweli…)	1 Nta na rimwe<br>2 Rimwe na rimwe<br>3 Kenshi<br>4 Buri gihe
A.5.3	A.5.3 Iby'inyongera (gutembera cyangwa impano…)	1 Nta na rimwe<br>2 Rimwe na rimwe<br>3 Kenshi<br>4 Buri gihe
A.7	A.7 Ni iyihe rangamimerere yawe?<br>*Niba usubiza avuze ko afite umugore, mubaze niba barasezeranye byemewe n'amategeko.*<br>*Question relevant when: ${A.1} >=18*	1 Ingaragu<br>2 Nashatse byemewe n'amategeko<br>3 Mfite umugore ariko ntitwasezeranye<br>4 Twaratandukanye<br>5 Umupfakazi
A.8	A.8 Ufite cyangwa wigeze ugira abagore barenze umwe icyarimwe?<br>*Question relevant when: ${A.1} >=18 and ${A.7} ='2' or ${A.7} ='3'*	0 Oya<br>1 Yego<br>999 Yanze gusubiza
note_A12	Ngiye kukubaza ibibazo birebana n'abantu mubana mu rugo.<br>*Question relevant when: ${A.1} >=18 and ${A.7} ='2' or ${A.7} ='3'*	
A.12	A.12 Mu rugo rwawe haba abantu bakuru (barengeje cg bafite imyaka 18) b'igitsina gabo bangahe wowe utibariyemo?<br>*[Andika umubare]*	

	Question relevant when: ${A.1} >=18 and ${A.7} ='2' or ${A.7} ='3'	
A.13	A.13 Mu rugo rwawe haba abantu bakuru (barengeje cg abfite imyaka 18) b'igitsina gore bangahe?<br>*[Andika umubare]*<br>*Question relevant when: ${A.1} >=18 and ${A.7} ='2' or ${A.7} ='3'*	
A.14	A.14 Mu rugo rwawe haba abana bangahe b'abahungu (abana bari munsi y'imyaka 18)?<br>*Abana b'abahungu bahaba bose n'ubwo baba atari abawe. [Andika umubare]*<br>*Question relevant when: ${A.1} >=18 and ${A.7} ='2' or ${A.7} ='3'*	
A.15	A.15 Mu rugo rwawe haba abana bangahe b'abakobwa (abana bari munsi y'imyaka 18)?<br>*Abana b'abakobwa bahaba bose n'ubwo baba atari abawe [Andika umubare]*<br>*Question relevant when: ${A.1} >=18 and ${A.7} ='2' or ${A.7} ='3'*	
A.16	A.16 Ufite abana bangahe?<br>*Tubwire abo wabyaye kandi bariho. [Andika umubare w'abana]*<br>*Question relevant when: ${A.1} >=18 and ${A.7} ='2' or ${A.7} ='3'*	
A.17	A.17 Tubwire imyaka n'igitsina bya buri mwana wawe wabyaye?<br>*Bwira ubazwa aguhe urutonde rw'abana bose uhereye ku mukuru. Niba ubazwa atazi imyaka y'umwana we andika 998. Wandike 0 niba umwana ari munsi y'umwaka umwe hanyuma wandike umubare w'amezi afite mugasanduku gakurikira.*<br>*Question relevant when: ${A.1} >=18 and ${A.16} !='0' and ${A.7} ='2' or ${A.7} ='3'*	
survey > Igice cya 1: Umwirondoro w'ubazwa n'ibindi biranga urugo rwe > Imyaka n'igitsina by'umwana wa (1)<br>*Group relevant when: ${A.1} >=18 and ${A.7} ='2' or ${A.7} ='3' and ${A.16} !='0'*		(Repeated group)
A.17.1	A.17.1 Imyaka y'umwana wa 1<br>*[Andika imyaka] Niba ubazwa atazi imyaka y'umwana we andika 998. Wandike 0 niba umwana ari munsi y'umwaka umwe hanyuma wandike umubare w'amezi afite mugasanduku gakurikira.*	
A.17.1a	A.17.1a Uyu mwana wawe wa 1 afite amzi angahe?<br>*Andika amezi*<br>*Question relevant when: ${A.17.1} ='0'*<br>*Response constrained to: .<=11*	
A.17.2	A.17.2 Igitsina cy'umwana wa 1	1 Gore<br>2 Gabo
A.18	A.18 Inzu mutuyemo ifite ibyumba bingahe?<br>*Andika umubare. Umukarani w'ibarura: Ntushyiremo urwogero, kuridoro, igaraje, ubuvumo<br>Icyumba kigabanyijwemo 2 n'umwenda kigomba kubarwamo ibyumba 2. Inzu zose zituwemwo zigomba kubarwa. [Enter number]*<br>*Question relevant when: ${A.1} >=18 and ${A.7} ='2' or ${A.7} ='3'*	
A.19	A.19 Ni hehe h'ibanze urugo rwanyu ruvoma amazi?<br>*Question relevant when: ${A.1} >=18 and ${A.7} ='2' or ${A.7} ='3'*	1 Ivomero riri mu rugo<br>2 Ivomero rya rusange<br>3 Ikigega gifata amazi<br>4 Iriba rusange<br>5 Iriba ry'undi muntu ku giti cye<br>6 Isoko itunganyije<br>7 Isoko idatunganyije<br>8 Umugezi/ikiyaga<br>9 Ivomero rikurura amazi mu butaka<br>10 Ibindi.<br>999 Yanze gusubiza
A.20	A.20 Ese hasi imbere mu nzu yawe hubakishijwe iki?<br>*Umukarani w'ibarura: niba ubazwa afite inzu zirenze imwe, wibande ku nzu y'ibanze (inziza kurusha izindi)*<br>*Question relevant when: ${A.1} >=18 and ${A.7} ='2' or ${A.7} ='3'*	1 Igitaka gitsindagiye<br>2 Amase yumye<br>3 Amakaro<br>4 Sima<br>5 Amatafari<br>6 Ibindi.<br>999 Yanze gusubiza
A.21	A.21 Ni ikihe gikoresho cy'ingenzi cyubakishije inkuta z'inzu mutuyemo?<br>*Umukarani w'ibarura: Niba hari inzu zirenze imwe, wibande ku nzu y'ibanze (inziza kurusha izindi)*<br>*Question relevant when: ${A.1} >=18 and ${A.7} ='2' or ${A.7} ='3'*	1 Rukarakara<br>2 Rukarakara isubirijwe na sima<br>3 Amatafari ahiye<br>4 Brokesima<br>5 Imbaho<br>6 Amabuye<br>7 Ibiti n'icyondo<br>8 Ibiti, icyindo na sima<br>9 Inkuta zikozwe muri plastike<br>10 Ibindi.<br>999 Yanze gusubiza
A.22	A.22 Ni iki cyubakishije igice kinini cy'isakaro ku nzu yawe?<br>*Question relevant when: ${A.1} >=18 and ${A.7} ='2' or ${A.7} ='3'*	1 Ibyatsi cyangwa amababi<br>2 Ibiti

		3	Amabati
		4	Amategura
		5	Plastike cyangwa imbaho
		6	Isakaro rya Asbestos/fibrosima
		7	Ibindi.
		999	Yanze gusubiza
A.23	A.23 Urugo rwanyu rufite amashanyarazi?<br>*Question relevant when: ${A.1} >=18 and ${A.7} ='2' or ${A.7} ='3'*	0	Oya
		1	Yego
		999	Yanze gusubiza
survey > Igice cya 1: Umwirondoro w'ubazwa n'ibindi biranga urugo rwe > own_item<br>*Group relevant when: ${A.1} >=18 and ${A.7} ='2' or ${A.7} ='3'*			
A.24	A.24 Haba hari umwe mu baba mu rugo rwanyu utunze ibi bikurikira kandi bikora neza?<br>*Kumubaruzi: Musomere bino.*		
A24.1	A24.1 Haba hari umwe mu baba mu rugo rwanyu utunze ibi bikurikira kandi bikora neza: Telefone ngendanwa	0	Oya
		1	Yego
		999	Yanze gusubiza
A24.2	A24.2 Haba hari umwe mu baba mu rugo rwanyu utunze ibi bikurikira kandi bikora neza: Intebe z'imisego	0	Oya
		1	Yego
		999	Yanze gusubiza
A24.3	A24.3 Haba hari umwe mu baba mu rugo rwanyu utunze ibi bikurikira kandi bikora neza: Ishyiga rikoreshwa n'amashanyarazi cyangwa gaze	0	Oya
		1	Yego
		999	Yanze gusubiza
A24.4	A24.4 Haba hari umwe mu baba mu rugo rwanyu utunze ibi bikurikira kandi bikora neza: Icyuma gikonjesha (firigo)	0	Oya
		1	Yego
		999	Yanze gusubiza
A24.5	A24.5 Haba hari umwe mu baba mu rugo rwanyu utunze ibi bikurikira kandi bikora neza: Radiyo / icyuma gisohora amajwi	0	Oya
		1	Yego
		999	Yanze gusubiza
A24.6	A24.6 Haba hari umwe mu baba mu rugo rwanyu utunze ibi bikurikira kandi bikora neza: Telefone itagendanwa	0	Oya
		1	Yego
		999	Yanze gusubiza
A24.7	A24.7 Haba hari umwe mu baba mu rugo rwanyu utunze ibi bikurikira kandi bikora neza: Imashini idoda	0	Oya
		1	Yego
		999	Yanze gusubiza
A24.8	A24.8 Haba hari umwe mu baba mu rugo rwanyu utunze ibi bikurikira kandi bikora neza: Ikinyabiziga gikoresha na moteri (yaba cyarakozeho cyangwa ari gishya)	0	Oya
		1	Yego
		999	Yanze gusubiza
A24.9	A24.9 Haba hari umwe mu baba mu rugo rwanyu utunze ibi bikurikira kandi bikora neza: Igare	0	Oya
		1	Yego
		999	Yanze gusubiza
A24.10	A24.10 Haba hari umwe mu baba mu rugo rwanyu utunze ibi bikurikira kandi bikora neza: Amatungo (urugero: inka, intama, ihene, inkoko)	0	Oya
		1	Yego
		999	Yanze gusubiza
A24.11	A24.11 Haba hari umwe mu baba mu rugo rwanyu utunze ibi bikurikira kandi bikora neza: Ubutaka	0	Oya
		1	Yego
		999	Yanze gusubiza
A24.12	A24.12 Haba hari umwe mu baba mu rugo rwanyu utunze ibi bikurikira kandi bikora neza: Inyubako cyangwa inzu	0	Oya
		1	Yego
		999	Yanze gusubiza
A.6	A.6 Mu rugo rwawe, ni nde ufite ubwishingizi bw'ubuzima?<br>*HITAMO IBISUBIZO BYOSE BISHOBOKA*<br>*Question relevant when: ${A.7} ='2' or ${A.7} ='3'*<br>*Response constrained to: count-selected(.)=1 or (count-selected(.)>=2 and not(selected(.,0)) and not(selected(.,998)) and not(selected(.,999)))*	0	Ntawe
		1	Njyewe
		2	Umugore wanjye
		3	Abana banjye (umwe cyangwa abana benshi)
		4	Abandi
		998	Simbizi
		999	Yanze gusubiza
A.25	A.25 Ese mu byumweru 2 bishize wigeze ushyira amafaranga ku ruhande yo kwizigama ubariyemo n'ayo waba	0	Oya

	waratanze mu matsinda yo kwizigama cyangwa mu bimina?<br>*Question relevant when: ${A.7} ='2' or ${A.7} ='3'*	1 Yego<br>999 Yanze gusubiza
survey > PART 2. Gutwita no kubyara<br>*Group relevant when: ${A.1} >=18 and ${A.7} ='2' or ${A.7} ='3'*		
note_b	Muri iki gice, ngiye kukubaza ibibazo birebana no gutwita no kubyara. Ndakwizeza ko ibisubizo byawe byose ari ibanga.	
B.1	B.1 Ese umugore wawe aratwite?	0 Oya<br>1 Yego<br>999 Yanze gusubiza
B.2	B.2 Muteganya ko azabyara ryari? Andika umubare w'amezi asigaye kugirango umwana avuke<br>*Andika 998 niba usubiza atabizi. Andika "0" mugihe hasigaye munsi y'ukwezi [enter amount in months]*<br>*Question relevant when: ${B.1} ='1'*<br>*Response constrained to: .<=9 or .=998*	
B.3	B.3 Kuva umugore wawe yasama, ni kangahe umaze kumuherekeza kwisuzumisha kwa muganga?<br>*Andika umubare*<br>*Question relevant when: ${B.1} ='1'*	
B.3.1	B.3.1 Ubwo uheruka guherekeza umugore wawe kwa muganga kwisuzumisha ubwo yari atwite, wabigenje ute?<br>*ICYITONDERWA: SOMERA UBAZWA IBISUBIZO.*<br>*Question relevant when: ${B.1} ='1' and ${B.3} !='0' and ${B.3} !=998*	1 Namugezaga ku irembo ry'ivuriro cyangwa ngasigara hanze yaryo<br>2 Namugezaga kwa muganga ariko ntitwinjiranye mu cyumba basuzumiramo<br>3 Nigeze kuba ndi kumwe nawe mu cyumba basuzumiramo<br>4 Izindi mpamvu.
B.3.2	B.3.2 Watubwira ukuntu wagiye mubijyanye no kwisuzumisha inda bwa mbere.<br>Uremeranya cyangwa ntiwemeranya n'ibi bikurikira bijyanye n'ubunararibonye bwawe:<br>*Question relevant when: ${B.1} ='1' and ${B.3} !='0' and ${B.3} !=998 and ${B.3.1} ='3'*	
B.3.2.1	B.3.2.1 Umukozi wo kwa muganga yatwakiriye neza<br>*Question relevant when: ${B.1} ='1' and ${B.3} !='0' and ${B.3} !=998 and ${B.3.1} ='3'*	0 Simbyemera<br>1 Ndabyemera
B.3.2.2	B.3.2.2 Umukozi wo kwa muganga yaranyirengagije<br>*Question relevant when: ${B.1} ='1' and ${B.3} !='0' and ${B.3} !=998 and ${B.3.1} ='3'*	0 Simbyemera<br>1 Ndabyemera
B.3.2.3	B.3.2.3 Nahamenyeye byinshi birebana no gutwita<br>*Question relevant when: ${B.1} ='1' and ${B.3} !='0' and ${B.3} !=998 and ${B.3.1} ='3'*	0 Simbyemera<br>1 Ndabyemera
B.3.2.4	B.3.2.4 Byamfashije kwitegura ivuka ry'umwana<br>*Question relevant when: ${B.1} ='1' and ${B.3} !='0' and ${B.3} !=998 and ${B.3.1} ='3'*	0 Simbyemera<br>1 Ndabyemera
B.3.2.5	B.3.2.5 Byatumye numva ndi hafi y'umugore wanjye<br>*Question relevant when: ${B.1} ='1' and ${B.3} !='0' and ${B.3} !=998 and ${B.3.1} ='3'*	0 Simbyemera<br>1 Ndabyemera
B.3.3.	B.3.3. Kubera iki utigeze kuba uri kumwe n'umugore wawe mu cyumba basuzumiramo?<br>*HITAMO IBISUBIZO BYOSE BISHOBOKA.*<br>*Question relevant when: ${B.1} ='1' and ${B.3} !='0' and ${B.3} !=998 and ${B.3.1} !='4' and ${B.3.1} !='3'*	1 Numvaga atari ngombwa ko njyana nawe kwa muganga<br>2 Si inshingano z'umugabo guherekeza umugore kwa muganga<br>3 Umugore wanjye yarabyanze ko tujyana<br>4 Abandi bantu banciye intege zo kujyayo<br>5 Muganga yambwiye ko ntabyemerewe<br>6 Nari nagiye ku kazi<br>7 Nari mu rugendo<br>8 Nta mwanya nari mfite wo kumuherekeza<br>9 Ntago narinzo ko nemerewe kwinjira mu cyumba basuzumiramo umubyeyi utwite<br>996 Izindi mpamvu.
B.6	B.6 Uteganya kuzaherekeza umugore wawe mu cyumba cyo kubyariramo?<br>*Question relevant when: ${B.1} ='1' and ${B.3} !='0' and ${B.3} !=998 and ${B.3.1} !='4'*	0 Oya<br>1 Yego<br>999 Yanze gusubiza
N.B.1	N.B.1 Ndifuza kukubaza ku bijyanye ni nda umugore wawe aherutse gutwita, iy'umwana uriho<br>*Question relevant when: ${A.16} !='0'*	

N.B.1.1	N.B.1.1 Mugihe umugore wawe yari afite inda aheruka gutwita, wigeze umuherekeza kwisuzumusha ku ivuriro muri gahunda yo gukirikirana abagore batwite?<br>*Question relevant when: ${A.16} !='0'*	0 Oya<br>1 Yego<br>2 Umugore wanjye ntiyigeze yitabira gahunda y'ubuvuzi bw'abagore batwite
B.7	B.7 Ubwo umugore wawe yari atwite umwana aheruka kubyara, ni kangahe wamuherekeje kwisuzumisha kwa muganga?<br>*Ndifuza kukubaza ku bijyanye ni inda umugore wawe aherutse gutwita, iy'umwana uriho. Andika 998 niba usubiza atabizi/atabyibuka. [Andika umubare]*<br>*Question relevant when: ${A.16} !='0' and ${N.B.1.1} ='1'*<br>*Response constrained to: .>0*	
B.8	B.8 Ubwo uheruka guherekeza umugore wawe kwa muganga, wabigenje ute?<br>*ICYITONDERWA: SOMERA UBAZWA IBISUBIZO.*<br>*Question relevant when: ${A.16} !='0' and ${N.B.1.1} ='1' and ${B.7} !='0'*	1 Namugezaga ku irembo ry'ivuriro cyangwa ngasigara hanze yaryo<br>2 Namugezaga kwa muganga ariko ntitwinjiranye mu cyumba basuzumiramo<br>3 Nigeze kuba ndi kumwe nawe mu cyumba basuzumiramo<br>4 Izindi mpamvu.
B.9	B.9 Watubwira ukuntu wagiye mubijyanye no kwisuzumisha inda bwa mbere.<br>Uremeranya cyangwa ntiwemeranya n'ibi bikurikira bijyanye n'ubunararibonye bwawe:<br>*Ibi bibazo birabaza ku inda umugore wawe aheruka ku byara y'umwana muto uriho*<br>*Question relevant when: ${A.16} !='0' and ${N.B.1.1} ='1' and ${B.7} !='0' and ${B.8} ='3'*	
B.9.1	B.9.1 Umukozi wo kwa muganga yatwakiriye neza<br>*Question relevant when: ${A.16} !='0' and ${N.B.1.1} ='1' and ${B.7} !='0' and ${B.8} ='3'*	0 Simbyemera<br>1 Ndabyemera
B.9.2	B.9.2 Umukozi wo kwa muganga yaranyirengagije<br>*Question relevant when: ${A.16} !='0' and ${N.B.1.1} ='1' and ${B.7} !='0' and ${B.8} ='3'*	0 Simbyemera<br>1 Ndabyemera
B.9.3	B.9.3 Nahamenyeye byinshi birebana no gutwita<br>*Question relevant when: ${A.16} !='0' and ${N.B.1.1} ='1' and ${B.7} !='0' and ${B.8} ='3'*	0 Simbyemera<br>1 Ndabyemera
B.9.4	B.9.4 Byamfashije kwitegura ivuka ry'umwana<br>*Question relevant when: ${A.16} !='0' and ${N.B.1.1} ='1' and ${B.7} !='0' and ${B.8} ='3'*	0 Simbyemera<br>1 Ndabyemera
B.9.5	B.9.5 Byatumye numva ndi hafi y'umugore wanjye<br>*Question relevant when: ${A.16} !='0' and ${N.B.1.1} ='1' and ${B.7} !='0' and ${B.8} ='3'*	0 Simbyemera<br>1 Ndabyemera
B.10	B.10 Kubera iki utigeze kuba uri kumwe n'umugore wawe mu cyumba basuzumiramo?<br>*HITAMO IBISUBIZO BYOSE BISHOBOKA.*<br>*Question relevant when: ${A.16} !='0' and ${N.B.1.1} ='1' and ${B.7} !='0' and ${B.8} !='3' and ${B.8} !='4'*	1 Numvaga atari ngombwa ko njyana nawe kwa muganga<br>2 Si inshingano z'umugabo guherekeza umugore kwa muganga<br>3 Umugore wanjye yarabyanze ko tujyana<br>4 Abandi bantu banciye intege zo kujyayo<br>5 Muganga yambwiye ko ntabyemerewe<br>6 Nari nagiye ku kazi<br>7 Nari mu rugendo<br>8 Nta mwanya nari mfite wo kumuherekeza<br>9 Ntago narinzo ko nemerewe kwinjira mu cyumba basuzumiramo umubyeyi utwite<br>10 Izindi mpamvu.
B.11	B.11 Umugore wawe yabyariye hehe?<br>*Question relevant when: ${A.16} !='0'*	1 Kwa muganga (urugero, ibitaro, cyangwa ivuriro cyangwa ikigonderabuzima.)<br>2 Mu rugo<br>3 Ahandi
B.12a	B.12a Wari uri he ubwo umwana wawe muto yavukaga?<br>*ICYITONDERWA: SOMERA UBAZWA IBISUBIZO.*<br>*Question relevant when: ${A.16} !='0' and ${B.11} ='1'*	1 Nari ndi mu cyumba umugore wanjye yabyariyemo<br>2 Nari ndi kwa muganga ahatari

		mu cyumba umugore wanjye yabyariyemo<br>3 Ntabwo nageze kwa muganga<br>4 Izindi mpavu
B.12b	B.12b Wari uri he ubwo umwana wawe muto yavukaga?<br>*ICYITONDERWA: SOMERA UBAZWA IBISUBIZO.*<br>*Question relevant when: ${A.16} !='0' and ${B.11} ='2'*	1 Nari ndi mu cyumba umugore wanjye yabyariyemo<br>2 Nari ndi mu rugo ahatari mu cyumba umugore wanjye yabyariyemo<br>3 Ntabwo narindi mu rugo<br>4 Izindi mpavu
B.13	B.13 Umwana wawe amaze kuvuka wumvise umerewe ute kuba wari mu cyumba umugore wawe yabyariyemo?<br>*ICYITONDERWA: SOMERA UBAZWA IBISUBIZO. HITAMO IBISUBIZO BYOSE BISHOBOKA.*<br>*Question relevant when: ${A.16} !='0' and ${B.11} !='3' and (${B.12a} ='1' or ${B.12b} ='1')*	1 Numvise ndushijeho gukunda umugore wanjye<br>2 Numvise nishimiye umwana wanjye<br>3 Nashimye ubutwari bw'umugore wanjye kubera kwihangana yagaragaje<br>4 Byanteye iseseme<br>5 Numvise mbuze icyo nakora<br>6 Byanteye ubwoba
B.14	B.14 Wambwira impamvu utari mu cyumba umugore wawe yabyariyemo ubwo aheruka kubyara?<br>*ICYITONDERWA: SOMERA UBAZWA IBISUBIZO. HITAMO IBISUBIZO BYOSE BISHOBOKA.*<br>*Question relevant when: ${B.12a} ='2' or ${B.12a} ='3'or ${B.12a} ='4' or ${B.12b} ='2' or ${B.12b} ='3' or ${B.12b} ='4'*	1 Numvaga atari ngombwa ko njyana nawe mu cyumba cyo kubyariramo<br>2 Si inshingano z'umugabo kwinjira mu cyumba cyo kubyariramo<br>3 Abagabo ntibagomba kuba hafi y'abagore babo mu gihe babyara<br>4 Umugore wanjye yanze ko abyara mpari<br>5 Abandi bantubanciye intege<br>6 Sinashakaga kubona umugore wanjye ababara<br>7 Muganga yambwiye ko ntabyemerewe<br>8 Yabyaye mu buryo bwihuse cyangwa butunguranye<br>9 Nari nagiye ku kazi<br>10 Nari mu rugendo<br>11 Nati mfite ubwoba<br>12 Izindi mpamvu.
B.15	B.15 Nyuma y'ivuka ry'umwana muheruka kubyara, wigeze ujya gukingiza uwo mwana cyangwa umufasha wawe yigeze amujyana kumukingiza cyangwa se mwembi mwigeze mujyana kumugingiza?<br>*Question relevant when: ${A.16} !='0' and ${B.1} ='1' and ${B.3} !='0' and ${B.3} !='998'*	0 Oya<br>1 Yego, naramwijyaniye njye ubwanjye<br>2 Yego, umufasha wanjye yaramujyanye<br>3 Yego, umwana twaramujyanye twembi, tujyanye cyangwa buri wese amujyana ukwe<br>997 Ntibimureba
noteb2	Ngiye kugusomera interuro zikurikira, urajya umbwira niba ibikubiyemo ubyemera cyangwa utabyemera:	
B.26	B.26 Umugabo ntagomba guherekeza umugore we kwipimisha inda kwa muganga	0 Simbyemera<br>1 Ndabyemera
B.27	B.27 Kuba umugabo ari mu cyumba cyo kubyariramo mu gihe umwana we avuka bimufasha kwegerana nawe no kumukunda cyane	0 Simbyemera<br>1 Ndabyemera
B.28	B.28 Kuba umugabo ari mu cyumba cyo kubyariramo mu gihe umugore we abyara bituma barushaho gukundana	0 Simbyemera<br>1 Ndabyemera

B.29	B.29 Umugore utwite agomba kubona umubyaza ubifitiye ubumenyi mu gihe cyo kubyara n'ubwo umugabo we yaba atabishaka.	0 Simbyemera<br>1 Ndabyemera
N.B.2	N.B.2 Ntibyemewe ko umugabo yitabira inshuro zirenze imwe gahunda zijyanye no kwipimisha inda ku mugore utwite	0 Simbyemera<br>1 Ndabyemera
survey > Igice cya 3. Ubuzima bw'imyororokere<br>*Group relevant when: ${A.1} >=18 and ${A.7} ='2' or ${A.7} ='3'*		
note_c	Ngiye kukuganiriza ku birebana n'imyitwarire yawe ku bijyanye n'ubuzima bw'imyororokere. Nk'uko nakomeje kubikwizeza ibisubizo uribumpe biraba ibanga. Niba hari ikibazo wumva udashaka gusubiza turagisimbuka.	
C.9	C.9 Ese hari uburyo bwo kuboneza urubyaro mukoresha ubungubu wowe n'umugore wawe?<br>*USHOBORA GUHITAMO IGISUBIZO KIRENZE KIMWE.*<br>*Response constrained to: count-selected(.)=1 or (count-selected(.)>=2 and not(selected(.,0)) and not(selected(.,998)) and not(selected(.,999)))*	0 Ntabwo<br>1 Ibinini<br>2 Agakingirizo k'abagabo<br>3 Agakingirizo k'abagore<br>4 Inshinge<br>5 Agapira ko mu mura<br>6 umugabo wifungishize burundu<br>7 Umugore akoresheje uburyo bwa burundu cyangwa se yabazwe nyababyeyi<br>8 Agapira ko mu kuboko<br>9 Uburyo gakondo (kwifata, kwiyakana, urunigi,..)<br>998 Simbizi<br>11 Ubundi buryo<br>999 Yanze gusubiza
C.10	C.10 Waba warigeze wumva uburyo bwo kwifungisha burundu ku bagabo?	0 Oya<br>1 Yego<br>998 Simbizi
C.11	C.11 Ni iki utekereza ku kwifungisha burundu kw'abagabo?<br>*ICYITONDERWA: SOMERA UBAZWA IBISUBIZO*<br>*Question relevant when: ${C.10} ='1'*	1 Nabyakiriye neza cyane<br>2 Nabyakiriye neza<br>3 Numvise ntacyo mbitekerezaho<br>4 Nabyakiriye nabi<br>5 Nabyakiriye nabi cyane
C.12	C.12 Ngiye kugusomera interuro zikurikira, urajya umbwira niba ibikubiyemo ubyemera cyangwa utabyemera:<br>*Question relevant when: ${C.10} ='1'*	
C.12.1	C.12.1 Umugabo waboneje urubyaro akoresheje uburyo bwa burundu akomeza gushimishwa no gukora imibonano mpuzabitsina.<br>*Question relevant when: ${C.10} ='1'*	0 Simbyemera<br>1 Ndabyemera
C.12.2	C.12.2 Umugabo wafunzwe burundu akomeza gushimisha umugore we mu gihe bakora imibonano mpuzabitsina.<br>*Question relevant when: ${C.10} ='1'*	0 Simbyemera<br>1 Ndabyemera
C.12.3	C.12.3 Iyo abantu bamenye ko umugabo yaboneje urubyaro akoresheje uburyo bwa burundu baramuseka batekereza ko atakiri umugabo nyamugabo.<br>*Question relevant when: ${C.10} ='1'*	0 Simbyemera<br>1 Ndabyemera
N.C.12.4	N.C.12.4 Kwifungisha burundu kw'abagabo ni uburyo buboneye bwo kwirinda inda mugihe umugabo n'umugore we batifuza kubyara abandi bana<br>*Question relevant when: ${C.10} ='1'*	0 Simbyemera<br>1 Ndabyemera
C.13	C.13 Ese wumva wakwifungisha burundu nk'uburyo bwo kuboneza urubyaro?<br>*Question relevant when: ${C.10} ='1'*	0 Oya<br>1 Yego<br>999 Yanze gusubiza
C.17	C.17 Ngiye kugusomera interuro zikurikira, urajya umbwira niba ibikubiyemo ubyemera cyane, niba ubyemera, niba ntacyo wabivugaho, niba ubihakana, cyangwa niba utabyemera na gato.	
C.17.1	C.17.1 Ntekereza ko gukoresha uburyo bwo kuboneza urubyaro atari byiza	1 Ndabyemera cyane<br>2 Ndabyemera<br>3 Simbyemeye kandi simbihakanye<br>4 Simbyemera<br>5 Simbyemera na gato
C.17.3	C.17.3 Numva byantera ipfunwe kuganira n'umufasha wanjye uburyo bwo kuboneza urubyaro	1 Ndabyemera cyane<br>2 Ndabyemera<br>3 Simbyemeye kandi simbihakanye

		4 Simbyemera<br>5 Simbyemera na gato
C.17.4	C.17.4 Uburyo bwo kuboneza urubyaro buraruhije kuboneka	1 Ndabyemera cyane<br>2 Ndabyemera<br>3 Simbyemeye kandi simbihakanye<br>4 Simbyemera<br>5 Simbyemera na gato
C.17.5	C.17.5 Bitera ipfunwe gusaba uburyo bwo kuboneza urubyaro	1 Ndabyemera cyane<br>2 Ndabyemera<br>3 Simbyemeye kandi simbihakanye<br>4 Simbyemera<br>5 Simbyemera na gato
C.17.6	C.17.6 Abashakanye bagomba kuganira ku buryo bwo kuboneza urubyaro mbere yo gukora imibonano mpuzabitsina	1 Ndabyemera cyane<br>2 Ndabyemera<br>3 Simbyemeye kandi simbihakanye<br>4 Simbyemera<br>5 Simbyemera na gato
C.17.7	C.17.7 Abantu babiri bakorana imibonano mpuzabitsina bagomba gukoresha bumwe mu buryo bwo kuboneza urubyaro niba batiteguye kubyara	1 Ndabyemera cyane<br>2 Ndabyemera<br>3 Simbyemeye kandi simbihakanye<br>4 Simbyemera<br>5 Simbyemera na gato
C.17.8	C.17.8 Uburyo bwo kuboneza urubyaro bugira ingaruka mbi ku buzima bw'abagore	1 Ndabyemera cyane<br>2 Ndabyemera<br>3 Simbyemeye kandi simbihakanye<br>4 Simbyemera<br>5 Simbyemera na gato
C.17.10	C.17.10 Agakingirizo ni uburyo bwizewe bwo kwirinda gusama	1 Ndabyemera cyane<br>2 Ndabyemera<br>3 Simbyemeye kandi simbihakanye<br>4 Simbyemera<br>5 Simbyemera na gato
C.17.11	C.17.11 Byantera ipfunwe gusaba ko dukoresha agakingirizo mu mibonano mpuzabitsina	1 Ndabyemera cyane<br>2 Ndabyemera<br>3 Simbyemeye kandi simbihakanye<br>4 Simbyemera<br>5 Simbyemera na gato
C.17.12	C.17.12 Agakingirizo kabishya imibonano mpuzabitsina	1 Ndabyemera cyane<br>2 Ndabyemera<br>3 Simbyemeye kandi simbihakanye<br>4 Simbyemera<br>5 Simbyemera na gato
survey > part4 Igice cya 4. Imibanire<br>*Group relevant when: ${A.1} >=18 and ${A.7} ='2' or ${A.7} ='3'*		
note_d	Ngiye kukuganiriza ku birebana n'imibanire yawe n'umugore wawe. Ndakwizeza ko ibisubizo byawe byose ari ibanga.	
D.1	D.1 Ngiye kugusomera interuro zikurikira maze umbwire ibizikubiyemo inshuro bikubaho. Maze unsubize niba bikubaho kenshi, rimwe na rimwe, gake gashoboka, cyangwa nta na rimwe.	
D.1.1	D.1.1 Iyo mfite ibibazo umugore wanjye aranyumva	1 Kenshi<br>2 Rimwe na rimwe<br>3 Gake gashoboka<br>4 Nta na rimwe<br>998 Simbizi

D.1.2	D.1.2 Iyo hari ibintu bitagenda neza, umugore wanjye arabingayira	1 Kenshi<br>2 Rimwe na rimwe<br>3 Gake gashoboka<br>4 Nta na rimwe<br>998 Simbizi
D.1.3	D.1.3 Numva umugore wanjye anyishimiye	1 Kenshi<br>2 Rimwe na rimwe<br>3 Gake gashoboka<br>4 Nta na rimwe<br>998 Simbizi
D.1.4	D.1.4 Numva nubashywe n'ubwo haba hari ibyo tutumvikanyeho	1 Kenshi<br>2 Rimwe na rimwe<br>3 Gake gashoboka<br>4 Nta na rimwe<br>998 Simbizi
D.1.5	D.1.5 Ibibazo byacu tubikemura neza mu bwumvikane	1 Kenshi<br>2 Rimwe na rimwe<br>3 Gake gashoboka<br>4 Nta na rimwe<br>998 Simbizi
D.1.6	D.1.6 Umugore wanjye agaya ibitekerezo, imbamutima n'ibyifuzo byanjye	1 Kenshi<br>2 Rimwe na rimwe<br>3 Gake gashoboka<br>4 Nta na rimwe<br>998 Simbizi
D.1.7	D.1.7 Umugore wanjye angaragariza urukundo n'ubwuzu	1 Kenshi<br>2 Rimwe na rimwe<br>3 Gake gashoboka<br>4 Nta na rimwe<br>998 Simbizi
D.2	D.2 Ngiye kugusomera interuro zikurikira, urajya umbwira niba ibikubiyemo ubyemera cyane, niba ubyemera, niba ubihakana, niba utabyemera na gato cyangwa se niba ntacyo wabivugaho.	
D.2.1	D.2.1 Ku mugoroba, njye n'umugore wanjye tuganira kenshi ku byatubayeho uwo umunsi	1 Ndabyemera cyane<br>2 Ndabyemera<br>3 Simbyemeye kandi simbihakanye<br>4 Simbyemera<br>5 Simbyemera na gato
D.3	D.3 Ushobora kumbwira inshuro wowe n'umugore wawe mugirana amakimbirane?<br>*MUSOMERE IBISUBIZO BYOSE BYATANZWE.*	1 Kenshi<br>2 Rimwe na rimwe<br>3 Gake gashoboka<br>4 Nta na rimwe<br>998 Simbizi
note_d5	Tugiye kuganira ku buryo uganira n'umugore wawe.	
D.5	D.5 Ni kangahe uganira n'uwo mwashakanye ku mikoreshereze y'amafaranga mwinjiza n'ayo musohora mu rugo mu cyumweru cyangwa mu kwezi?	1 Kenshi<br>2 Rimwe na rimwe<br>3 Gake gashoboka<br>4 Nta na rimwe<br>997 Ntibimureba<br>999 Yanze gusubiza
D.5.3	D.5.3 Ni nde ufata icyemezo cya nyuma ku bijyanye n'ikoreshwa ry'amafaranga mwinjiza n'ayo musohora mu rugo mu cyumweru cyangwa mu kwezi?<br>*Question relevant when: ${D.5} !='997'*	1 Wowe<br>2 Umugore wawe<br>3 Mufite uruhare rungana<br>4 Undi muntu<br>998 Simbizi<br>999 Yanze gusubiza
D.6	D.6 Ni kangahe muganira nuwo mwashakanye ku bijyanye no kugura ibintu bitwara amafaranga menshi (urugero: inka, ishyamba, umurima)?	1 Kenshi<br>2 Rimwe na rimwe<br>3 Gake gashoboka<br>4 Nta na rimwe

		997	Ntibimureba
		999	Yanze gusubiza
D.6.3	D.6.3 Ni nde ufata icyemezo cya nyuma ku bijyanye no kugura ibintu bitwara amafaranga menshi? (urugero: Isambu, umurima, inka…)?<br>*Question relevant when: ${D.6} !='997'*	1	Wowe
		2	Umugore wawe
		3	Mufite uruhare rungana
		4	Undi muntu
		998	Simbizi
		999	Yanze gusubiza
D.7	D.7 Ni kangahe muganira n'uwo mwashakanye ku bijyanye n'umubare w'abana mwifuza kubyara n'uko mwabakurikiza?	1	Kenshi
		2	Rimwe na rimwe
		3	Gake gashoboka
		4	Nta na rimwe
		997	Ntibimureba
		999	Yanze gusubiza
D.7.3	D.7.3 Ni nde ufata icyemezo cya nyuma mu bijyanye n'umubare w'abana mwifuza kubyara cyangwa uko mwabakurikiza?<br>*Question relevant when: ${D.7} !='997'*	1	Wowe
		2	Umugore wawe
		3	Mufite uruhare rungana
		4	Undi muntu
		998	Simbizi
		999	Yanze gusubiza
D.8	D.8 Ni kangahe muganira n'uwo mwashakanye niba bishoboka ko yakorera hanze yo mu rugo?	1	Kenshi
		2	Rimwe na rimwe
		3	Gake gashoboka
		4	Nta na rimwe
		997	Ntibimureba
		999	Yanze gusubiza
D.8.3	D.8.3 Ni nde ufata icyemezo cya nyuma ku bijyanye no kuba uwo mwashakanye yajya gukorera ahandi hatari mu rugo?<br>*Question relevant when: ${D.8} !='997'*	1	Wowe
		2	Umugore wawe
		3	Mufite uruhare rungana
		4	Undi muntu
		998	Simbizi
		999	Yanze gusubiza
D.9	D.9 Ni kangahe muganira n'uwo mwashakanye ku bijyanye n'imikoreshereze y'amafaranga umugore wawe yinjiza?	1	Kenshi
		2	Rimwe na rimwe
		3	Gake gashoboka
		4	Nta na rimwe
		997	Ntibimureba
		999	Yanze gusubiza
D.9.3	D.9.3 Ni nde ufata icyemezo cya nyuma ku bijyanye n'uburyo amafaranga umugore yinjiza akoreshwa?<br>*Question relevant when: ${D.9} !='997'*	1	Wowe
		2	Umugore wawe
		3	Mufite uruhare rungana
		4	Undi muntu
		998	Simbizi
		999	Yanze gusubiza
D.10	D.10 Ni kangahe muganira n'uwo mwashakanye uburyo amafaranga winjiza akoreshwa?	1	Kenshi
		2	Rimwe na rimwe
		3	Gake gashoboka
		4	Nta na rimwe
		997	Ntibimureba
		999	Yanze gusubiza
D.10.3	D.10.3 Ni nde ufata icyemezo cya nyuma ku bijyanye n'uburyo amafaranga winjiza akoreshwa?<br>*Question relevant when: ${D.10} !='997'*	1	Wowe
		2	Umugore wawe
		3	Mufite uruhare rungana
		4	Undi muntu
		998	Simbizi
		999	Yanze gusubiza
D.11	D.11 Ni kangahe muganira n'uwo mwashakanye niba mwaboneza urubyaro cyangwa uburyo bwo kuboneza urubyaro mukoresha?	1	Kenshi
		2	Rimwe na rimwe
		3	Gake gashoboka
		4	Nta na rimwe

		997 Ntibimureba<br>999 Yanze gusubiza
D.11.3	D.11.3 Ni nde ufata icyemezo cya nyuma ku bijyanye no kuboneza urubyaro cyangwa uburyo mwakoresha mu kuboneza urubyaro?<br>*Question relevant when: ${D.11} !='997'*	1 Wowe<br>2 Umugore wawe<br>3 Mufite uruhare rungana<br>4 Undi muntu<br>998 Simbizi<br>999 Yanze gusubiza
D.13	D.13 Ni kangahe muganira n'uwo mwashakanye ku bijyanye n'amashuli n'imyigire by'abana?	1 Kenshi<br>2 Rimwe na rimwe<br>3 Gake gashoboka<br>4 Nta na rimwe<br>997 Ntibimureba<br>999 Yanze gusubiza
D.13.3	D.13.3 Ni nde ufata icyemezo cya nyuma ku bijyanye n'amashuli n'imyigire by'abana?<br>*Question relevant when: ${D.13} !='997'*	1 Wowe<br>2 Umugore wawe<br>3 Mufite uruhare rungana<br>4 Undi muntu<br>998 Simbizi<br>999 Yanze gusubiza
survey > part5 PART 5. Kwita ku bandi<br>*Group relevant when: ${A.1} >=18 and ${A.7} ='2' or ${A.7} ='3'*		
note_5	Ngiye kukubaza ibibazo bike birebana n'umuryango wawe n'imirimo yo mu rugo.<br>*Question relevant when: ${A.16} !='0'*	
note_5b	Ubu ngiye kukubaza ukuntu wowe n'umugore wawe mugabana imirimo itandukanye yo kwita ku mwana.<br>*ICYITONDERWA: Ibibazo bikurikira birabaza uko umugabo n'umugore we basaranganya imirimo yo kwita ku mwana. Kubibazo bimwe na bimwe nkurugero, urazagusanga umwana akiri muto kuba jajya kwishuri, cyangwa hari ibikorwa byabajijwe bikaba bikorwa n'umukozi wo murugo cyangwa umuvandimwe. Kubibazo nkibyo, andika 'Ntibimureba'*<br>*Question relevant when: ${A.16} !='0'*	
E.12	E.12 Ukuyemo ubufasha bw'abantu baba hanze y'urugo rwanyu, ni gute wowe n'umugore wawe musaranganya ibi bikorwa? Kwita ku mwana cyangwa abana banyu buri munsi<br>*Question relevant when: ${A.16} !='0'*	1 Burigihe ni wowe<br>2 Kenshi ni wowe<br>3 Turasaranganya cyangwa tubikorera hamwe<br>4 Kenshi ni uwomwashakanye<br>5 Burigihe ni uwomwashakanye<br>997 Ntibimureba
E.12a	E.12a Mu minsi 7 ishize, wakoze kino gikorwa iminsi ingahe?<br>*Question relevant when: ${A.16} !='0' and ${E.12} !='997'*<br>*Response constrained to: .<=7*	
E.12b	E.12b Ku munsi usanzwe, iyo wakoze kino gikorwa, ukoresha amasaha angahe?<br>*Gerageza wandi ighe mu icyikubo cya 15 (Urugero: niba umuntu avuze ko yakoresheje iminota 15 ku murimo andiko 0.25. burungushura ugeze ku minota 15, burungushura indi mibare yose kuza ku gikubo cya 15 kiri hafi aho.(urugero: niba ari iminota 35 uburungushure ushyire ku minota 30, hanyuma wandike amasaha 0.5) [Andika umubare w'amasaha]*<br>*Question relevant when: ${A.16} !='0' and ${E.12} !='997' and ${E.12a} !='0'*<br>*Response constrained to: .<=24*	
E.13	E.13 Ukuyemo ubufasha bw'abantu baba hanze y'urugo rwanyu, ni gute wowe n'umugore wawe musaranganya ibi bikorwa? Guterura uruhinja rwawe rukimara kuvuka<br>*Question relevant when: ${A.16} !='0'*	1 Burigihe ni wowe<br>2 Kenshi ni wowe<br>3 Turasaranganya cyangwa tubikorera hamwe<br>4 Kenshi ni uwomwashakanye<br>5 Burigihe ni uwomwashakanye<br>997 Ntibimureba
E.13a	E.13a Mu minsi 7 ishize, wakoze kino gikorwa iminsi ingahe?<br>*[Andika umubare w'iminsi]*<br>*Question relevant when: ${A.16} !='0' and ${E.13} !='997'*<br>*Response constrained to: .<=7*	
E.13b	E.13b Ku munsi usanzwe, iyo wakoze kino gikorwa, ukoresha amasaha angahe?<br>*Gerageza wandi ighe mu icyikubo cya 15 (Urugero: niba umuntu avuze ko yakoresheje iminota 15 ku murimo andiko 0.25. burungushura ugeze ku minota 15, burungushura indi mibare yose kuza ku gikubo cya 15 kiri hafi aho.(urugero: niba ari iminota 35 uburungushure ushyire ku minota 30, hanyuma wandike amasaha 0.5) [Andika umubare w'amasaha]*<br>*Question relevant when: ${A.16} !='0' and ${E.13} !='997'and ${E.13a} !='0'*<br>*Response constrained to: .<=24*	

E.14	E.14 Ukuyemo ubufasha bw'abantu baba hanze y'urugo rwanyu, ni gute wowe n'umugore wawe musaranganya ibi bikorwa? Kugaburira umwana cg abana banyu<br>*Question relevant when: ${A.16} !='0'*	1 Burigihe ni wowe<br>2 Kenshi ni wowe<br>3 Turasaranganya cyangwa tubikorera hamwe<br>4 Kenshi ni uwomwashakanye<br>5 Burigihe ni uwomwashakanye<br>997 Ntibimureba
E.14a	E.14a Mu minsi 7 ishize, wakoze kino gikorwa iminsi ingahe?<br>*[Andika umubare w'iminsi]*<br>*Question relevant when: ${A.16} !='0' and ${E.14} !='997'*<br>*Response constrained to: .<=7*	
E.14b	E.14b Ku munsi usanzwe, iyo wakoze kino gikorwa, ukoresha amasaha angahe?<br>*Gerageza wandi ighe mu icyikubo cya 15 (Urugero: niba umuntu avuze ko yakoresheje iminota 15 ku murimo andiko 0.25. burungushura ugeze ku minota 15, burungushura indi mibare yose kuza ku gikubo cya 15 kiri hafi aho.(urugero: niba ari iminota 35 uburungushure ushyire ku minota 30, hanyuma wandike amasaha 0.5) [Andika umubare w'amasaha]*<br>*Question relevant when: ${A.16} !='0' and ${E.14} !='997' and ${E.14a} !='0'*<br>*Response constrained to: .<=24*	
E.15	E.15 Ukuyemo ubufasha bw'abantu baba hanze y'urugo rwanyu, ni gute wowe n'umugore wawe musaranganya ibi bikorwa? Kwuhagira umwana cg abana banyu<br>*Question relevant when: ${A.16} !='0'*	1 Burigihe ni wowe<br>2 Kenshi ni wowe<br>3 Turasaranganya cyangwa tubikorera hamwe<br>4 Kenshi ni uwomwashakanye<br>5 Burigihe ni uwomwashakanye<br>997 Ntibimureba
E.15a	E.15a Mu minsi 7 ishize, wakoze kino gikorwa iminsi ingahe?<br>*[Andika umubare w'iminsi]*<br>*Question relevant when: ${A.16} !='0' and ${E.15} !='997'*<br>*Response constrained to: .<=7*	
E.15b	E.15b Ku munsi usanzwe, iyo wakoze kino gikorwa, ukoresha amasaha angahe?<br>*Gerageza wandi ighe mu icyikubo cya 15 (Urugero: niba umuntu avuze ko yakoresheje iminota 15 ku murimo andiko 0.25. burungushura ugeze ku minota 15, burungushura indi mibare yose kuza ku gikubo cya 15 kiri hafi aho.(urugero: niba ari iminota 35 uburungushure ushyire ku minota 30, hanyuma wandike amasaha 0.5) [Andika umubare w'amasaha]*<br>*Question relevant when: ${A.16} !='0' and ${E.15} !='997' and ${E.15a} !='0'*<br>*Response constrained to: .<=24*	
E.16	E.16 Ukuyemo ubufasha bw'abantu baba hanze y'urugo rwanyu, ni gute wowe n'umugore wawe musaranganya ibi bikorwa? Guhoza umwana cg abana banyu igihe arize cyangwa yarakaye<br>*Question relevant when: ${A.16} !='0'*	1 Burigihe ni wowe<br>2 Kenshi ni wowe<br>3 Turasaranganya cyangwa tubikorera hamwe<br>4 Kenshi ni uwomwashakanye<br>5 Burigihe ni uwomwashakanye<br>997 Ntibimureba
E.16a	E.16a Mu minsi 7 ishize, wakoze kino gikorwa iminsi ingahe?<br>*[Andika umubare w'iminsi]*<br>*Question relevant when: ${A.16} !='0' and ${E.16} !='997'*<br>*Response constrained to: .<=7*	
E.16b	E.16b Ku munsi usanzwe, iyo wakoze kino gikorwa, ukoresha amasaha angahe?<br>*Gerageza wandi ighe mu icyikubo cya 15 (Urugero: niba umuntu avuze ko yakoresheje iminota 15 ku murimo andiko 0.25. burungushura ugeze ku minota 15, burungushura indi mibare yose kuza ku gikubo cya 15 kiri hafi aho.(urugero: niba ari iminota 35 uburungushure ushyire ku minota 30, hanyuma wandike amasaha 0.5) [Andika umubare w'amasaha]*<br>*Question relevant when: ${A.16} !='0' and ${E.16} !='997' and ${E.16a} !='0'*<br>*Response constrained to: .<=24*	
E.171	E.171 Ukuyemo ubufasha bw'abantu baba hanze y'urugo rwanyu, ni gute wowe n'umugore wawe musaranganya ibi bikorwa? Kujyana umwana cg abana banyu ku kigo nderabuzima mu gihe arwaye cyangwa kumukingiza<br>*Question relevant when: ${A.16} !='0'*	1 Burigihe ni wowe<br>2 Kenshi ni wowe<br>3 Turasaranganya cyangwa tubikorera hamwe<br>4 Kenshi ni uwomwashakanye<br>5 Burigihe ni uwomwashakanye<br>997 Ntibimureba
E.171a	E.171a Mu minsi 7 ishize, wakoze kino gikorwa iminsi ingahe?<br>*[Andika umubare w'iminsi]*<br>*Question relevant when: ${A.16} !='0' and ${E.171} !='997'*<br>*Response constrained to: .<=7*	

E.17	E.17 Ukuyemo ubufasha bw'abantu baba hanze y'urugo rwanyu, ni gute wowe n'umugore wawe musaranganya ibi bikorwa? Kujyana no kuvana umwana/ abana banyu ku ishuri<br>*Question relevant when: ${A.16} !='0'*	1 Burigihe ni wowe<br>2 Kenshi ni wowe<br>3 Turasaranganya cyangwa tubikorera hamwe<br>4 Kenshi ni uwomwashakanye<br>5 Burigihe ni uwomwashakanye<br>997 Ntibimureba
E.17a	E.17a Mu minsi 7 ishize, wakoze kino gikorwa iminsi ingahe?<br>*[Andika umubare w'iminsi]*<br>*Question relevant when: ${A.16} !='0' and ${E.17} !='997'*<br>*Response constrained to: .<=7*	
E.17b	E.17b Ku munsi usanzwe, iyo wakoze kino gikorwa, ukoresha amasaha angahe?<br>*Gerageza wandi ighe mu icyikubo cya 15 (Urugero: niba umuntu avuze ko yakoresheje iminota 15 ku murimo andiko 0.25. burungushura ugeze ku minota 15, burungushura indi mibare yose kuza ku gikubo cya 15 kiri hafi aho.(urugero: niba ari iminota 35 uburungushure ushyire ku minota 30, hanyuma wandike amasaha 0.5) [Andika umubare w'amasaha]*<br>*Question relevant when: ${A.16} !='0' and ${E.17} !='997' and ${E.17a} !='0'*<br>*Response constrained to: .<=24*	
E.18	E.18 Ukuyemo ubufasha bw'abantu baba hanze y'urugo rwanyu, ni gute wowe n'umugore wawe musaranganya ibi bikorwa? Gucira umwana/abana banyu imigani, kumuririmbira no kumukinisha<br>*Question relevant when: ${A.16} !='0'*	1 Burigihe ni wowe<br>2 Kenshi ni wowe<br>3 Turasaranganya cyangwa tubikorera hamwe<br>4 Kenshi ni uwomwashakanye<br>5 Burigihe ni uwomwashakanye<br>997 Ntibimureba
E.18a	E.18a Mu minsi 7 ishize, wakoze kino gikorwa iminsi ingahe?<br>*[Andika umubare w'iminsi]*<br>*Question relevant when: ${A.16} !='0' and ${E.18} !='997'*<br>*Response constrained to: .<=7*	
E.18b	E.18b Ku munsi usanzwe, iyo wakoze kino gikorwa, ukoresha amasaha angahe?<br>*Gerageza wandi ighe mu icyikubo cya 15 (Urugero: niba umuntu avuze ko yakoresheje iminota 15 ku murimo andiko 0.25. burungushura ugeze ku minota 15, burungushura indi mibare yose kuza ku gikubo cya 15 kiri hafi aho.(urugero: niba ari iminota 35 uburungushure ushyire ku minota 30, hanyuma wandike amasaha 0.5) [Andika umubare w'amasaha]*<br>*Question relevant when: ${A.16} !='0' and ${E.18} !='997' and ${E.18a} !='0'*<br>*Response constrained to: .<=24*	
E.19	E.19 Ukuyemo ubufasha bw'abantu baba hanze y'urugo rwanyu, ni gute wowe n'umugore wawe musaranganya ibi bikorwa? Kwigisha umwana/ abana banyu ikintu icyo aricyo cyose.<br>*Question relevant when: ${A.16} !='0'*	1 Burigihe ni wowe<br>2 Kenshi ni wowe<br>3 Turasaranganya cyangwa tubikorera hamwe<br>4 Kenshi ni uwomwashakanye<br>5 Burigihe ni uwomwashakanye<br>997 Ntibimureba
E.19a	E.19a Mu minsi 7 ishize, wakoze kino gikorwa iminsi ingahe?<br>*[Andika umubare w'iminsi]*<br>*Question relevant when: ${A.16} !='0' and ${E.19} !='997'*<br>*Response constrained to: .<=7*	
E.19b	E.19b Ku munsi usanzwe, iyo wakoze kino gikorwa, ukoresha amasaha angahe?<br>*Gerageza wandi ighe mu icyikubo cya 15 (Urugero: niba umuntu avuze ko yakoresheje iminota 15 ku murimo andiko 0.25. burungushura ugeze ku minota 15, burungushura indi mibare yose kuza ku gikubo cya 15 kiri hafi aho.(urugero: niba ari iminota 35 uburungushure ushyire ku minota 30, hanyuma wandike amasaha 0.5) [Andika umubare w'amasaha]*<br>*Question relevant when: ${A.16} !='0' and ${E.19} !='997' and ${E.19a} !='0'*<br>*Response constrained to: .<=24*	
E.20	E.20 Ukuyemo ubufasha bw'abantu baba hanze y'urugo rwanyu, ni gute wowe n'umugore wawe musaranganya ibi bikorwa? Gucyaha umwana/ abana banyu<br>*Question relevant when: ${A.16} !='0'*	1 Burigihe ni wowe<br>2 Kenshi ni wowe<br>3 Turasaranganya cyangwa tubikorera hamwe<br>4 Kenshi ni uwomwashakanye<br>5 Burigihe ni uwomwashakanye<br>997 Ntibimureba
E.20a	E.20a Mu minsi 7 ishize, wakoze kino gikorwa iminsi ingahe?<br>*[Andika umubare w'iminsi]*<br>*Question relevant when: ${A.16} !='0' and ${E.20} !='997'*<br>*Response constrained to: .<=7*	

E.21	E.21 Ukuyemo ubufasha bw'abantu baba hanze y'urugo rwanyu, ni gute wowe n'umugore wawe musaranganya ibi bikorwa? Kunyuza akanyafu ku mwana/ abana banyu<br>*Question relevant when: ${A.16} !='0'*	1 Burigihe ni wowe<br>2 Kenshi ni wowe<br>3 Turasaranganya cyangwa tubikorera hamwe<br>4 Kenshi ni uwomwashakanye<br>5 Burigihe ni uwomwashakanye<br>997 Ntibimureba
E.21a	E.21a Mu minsi 7 ishize, wakoze kino gikorwa iminsi ingahe?<br>*[Andika umubare w'iminsi]*<br>*Question relevant when: ${A.16} !='0' and ${E.21} !='997'*<br>*Response constrained to: .<=7*	
E.40	E.40 Wifuza ko wagira uruhare rungana iki mu kwita ku mwana wawe cg ababa bawe?<br>*Question relevant when: ${A.16} !='0'*	1 Nshimishijwe n'uruhare rwanjye ubu.<br>2 Nifuza kugira uruhare rurenzeho<br>3 Nifuza kugira uruhare rukeya.
E.4	E.4 Utekereza ko umugore wawe yifuza ko wagira uruhare rungana iki mu kwita ku mwana wanyu/abana banyu?<br>*MUSOMERE IBISUBIZO BYOSE BYATANZWE*<br>*Question relevant when: ${A.16} !='0'*	1 Yishimiye uruhare rwanjye ubu<br>2 Yakwifuje yuko nongera uruhare rwanjye<br>3 Yifuza ko nagabanya uruhare rwanjye<br>998 Simbizi
E.10	E.10 Ngiye kukubaza ibizazo bike birebana no kurera abana. Ababyeyi bakoresha uburyo runaka mu kwigisha abana babo uko bitwara neza no kubakosora igihe bitwaye nabi. Ndasoma bumwe mu buryo ababyeyi bakoresha. Umbwire niba warigeze ukoresha bumwe muri ubu buryo mu ukwezi gushize kuri umwe mu bana bawe.<br>*Question relevant when: ${A.16} !='0'*	
E.10.1	E.10.1 Wamuvanye k'ubutoni, wabujije umwana wawe ikintu yakoze cyangwa ntiwawemereye umwana wawe gusohoka mu rugo<br>*Iki kibazo kirabaza ku byabaye mu gihe cy'ukwezi gushize(Iminsi 30 ishize) kandi bikareba gusa abana bawe wibyariye*<br>*Question relevant when: ${A.16} !='0'*	0 Oya<br>1 Yego
E.10.2	E.10.2 Wasobanuriye umwana wawe buryo ki imyitwarire ye itari ikwiye<br>*Iki kibazo kirabaza ku byabaye mu gihe cy'ukwezi gushize(Iminsi 30 ishize) kandi bikareba gusa abana bawe wibyariye*<br>*Question relevant when: ${A.16} !='0'*	0 Oya<br>1 Yego
E.10.3	E.10.3 Watigishije umwana wawe<br>*Iki kibazo kirabaza ku byabaye mu gihe cy'ukwezi gushize(Iminsi 30 ishize) kandi bikareba gusa abana bawe wibyariye*<br>*Question relevant when: ${A.16} !='0'*	0 Oya<br>1 Yego
E.10.4	E.10.4 Watonganyije umwana wawe uvuga cyane<br>*Iki kibazo kirabaza ku byabaye mu gihe cy'ukwezi gushize(Iminsi 30 ishize) kandi bikareba gusa abana bawe wibyariye*<br>*Question relevant when: ${A.16} !='0'*	0 Oya<br>1 Yego
E.10.5	E.10.5 Washakiye umwana wawe ikintu cyo gukora<br>*Iki kibazo kirabaza ku byabaye mu gihe cy'ukwezi gushize(Iminsi 30 ishize) kandi bikareba gusa abana bawe wibyariye*<br>*Question relevant when: ${A.16} !='0'*	0 Oya<br>1 Yego
E.10.6	E.10.6 Wakubise umwana wawe urushyi ku kibuno<br>*Iki kibazo kirabaza ku byabaye mu gihe cy'ukwezi gushize(Iminsi 30 ishize) kandi bikareba gusa abana bawe wibyariye*<br>*Question relevant when: ${A.16} !='0'*	0 Oya<br>1 Yego
E.10.7	E.10.7 Wakubise umwana wawe umukandara, inkoni cyangwa ikindi kintu kibabaza ku kibuno cyangwa ahandi ku mubiri<br>*Iki kibazo kirabaza ku byabaye mu gihe cy'ukwezi gushize(Iminsi 30 ishize) kandi bikareba gusa abana bawe wibyariye*<br>*Question relevant when: ${A.16} !='0'*	0 Oya<br>1 Yego
E.10.8	E.10.8 Wise umwana wawe igicucu, umunebwe cyangwa irindi zina nk'iryo<br>*Iki kibazo kirabaza ku byabaye mu gihe cy'ukwezi gushize(Iminsi 30 ishize) kandi bikareba gusa abana bawe wibyariye*<br>*Question relevant when: ${A.16} !='0'*	0 Oya<br>1 Yego
E.10.9	E.10.9 Wakubise urushyi umwana wawe mu maso, mu mutwe cyangwa ku matwi<br>*Iki kibazo kirabaza ku byabaye mu gihe cy'ukwezi gushize(Iminsi 30 ishize) kandi bikareba gusa abana bawe wibyariye*<br>*Question relevant when: ${A.16} !='0'*	0 Oya<br>1 Yego
E.10.10	E.10.10 Wakubise inshyi umwana wawe ku biganza, amaboko cyangwa amaguru<br>*Iki kibazo kirabaza ku byabaye mu gihe cy'ukwezi gushize(Iminsi 30 ishize) kandi bikareba gusa abana bawe wibyariye*<br>*Question relevant when: ${A.16} !='0'*	0 Oya<br>1 Yego
E.10.11	E.10.11 Wakubise umwana wawe uko wari ushoboye kose<br>*Iki kibazo kirabaza ku byabaye mu gihe cy'ukwezi gushize(Iminsi 30 ishize) kandi bikareba gusa abana bawe wibyariye*<br>*Question relevant when: ${A.16} !='0'*	0 Oya<br>1 Yego
E.10.12	E.10.12 Wapfukamishije umwana wawe<br>*Iki kibazo kirabaza ku byabaye mu gihe cy'ukwezi gushize(Iminsi 30 ishize) kandi bikareba gusa abana bawe wibyariye*<br>*Question relevant when: ${A.16} !='0'*	0 Oya<br>1 Yego
E.10.13	E.10.13 Wikoreje umwana wawe amatafari, amabuye, mu biganza yicaye, ahagaze cyangwa apfukamye	0 Oya

	Iki kibazo kirabaza ku byabaye mu gihe cy'ukwezi gushize(Iminsi 30 ishize) kandi bikareba gusa abana bawe wibyariye<br>*Question relevant when: ${A.16} !='0'*	1 Yego
E.11	E.11 Ngiye kugusomera interuro hanyuma umbwire niba ibikubiyemo ubyemera cyane, niba ubyemera, niba ntacyo wabivugaho, niba utabyemerea cyangwa se niba utabyemera na gato.<br>*Question relevant when: ${A.16} !='0'*	
E.11.1	E.11.1 Kugirango abana barerwe bikwiye, bakeneye guhabwa ibihano byo ku mubiri<br>*Question relevant when: ${A.16} !='0'*	1 Ndabyemera cyane<br>2 Ndabyemera<br>3 Simbyemeye kandi simbihakanye<br>4 Simbyemera<br>5 Simbyemera na gato
E.11.2	E.11.2 Ibihano byo ku mubiri bigira ingaruka mbi ku bana<br>*Question relevant when: ${A.16} !='0'*	1 Ndabyemera cyane<br>2 Ndabyemera<br>3 Simbyemeye kandi simbihakanye<br>4 Simbyemera<br>5 Simbyemera na gato
note_E22	Ubu ngiye kukubaza ukuntu wowe n'umugore wawe mugabana imirimo itandukanye yo mu rugo.<br>*ICYITONDERWA: Ibibazo bikurikira birabaza uko umugabo n'umugore we basaranganya imirimo yo murugo. Kubibazo bimwe na bimwe urazagusanga igikorwa kitareba uwo ariwe wese murugo, cyangwa gikaba gokorwa n'undi muntu wo murugo (urugero umukozi wo murugo cyangwa umuvandimwe). Kubibazo nkibyo, andika 'Ntibimureba'*	
E.22	E.22 Ukuyemo ubufasha bw'abantu baba hanze y'urugo rwanyu, ni gute wowe n'umugore wawe musaranganya ibi bikorwa? Kuvoma amazi yo gukoresha murugo (ubariyemo igihe gikoreshwa kujyayo no kugaruka)	1 Burigihe ni wowe<br>2 Kenshi ni wowe<br>3 Turasaranganya cyangwa tubikorera hamwe<br>4 Kenshi ni uwomwashakanye<br>5 Burigihe ni uwomwashakanye<br>997 Ntibimureba
E.22a	E.22a Mu minsi 7 ishize, wakoze kino gikorwa iminsi ingahe?<br>*[Andika umubare w'iminsi]*<br>*Question relevant when: ${E.22} !='997'*<br>*Response constrained to: .<=7*	
E.22b	E.22b Ku munsi usanzwe, iyo wakoze kino gikorwa, ukoresha amasaha angahe?<br>*Gerageza wandi ighe mu icyikubo cya 15 (Urugero: niba umuntu avuze ko yakoresheje iminota 15 ku murimo andiko 0.25. burungushura ugeze ku minota 15, burungushura indi mibare yose kuza ku gikubo cya 15 kiri hafi aho.(urugero: niba ari iminota 35 uburungushure ushyire ku minota 30, hanyuma wandike amasaha 0.5) [Andika umubare w'amasaha]*<br>*Question relevant when: ${E.22} !='997' and ${E.22a} !='0'*<br>*Response constrained to: .<=24*	
E.23	E.23 Ukuyemo ubufasha bw'abantu baba hanze y'urugo rwanyu, ni gute wowe n'umugore wawe musaranganya ibi bikorwa? Kumesa imyenda	1 Burigihe ni wowe<br>2 Kenshi ni wowe<br>3 Turasaranganya cyangwa tubikorera hamwe<br>4 Kenshi ni uwomwashakanye<br>5 Burigihe ni uwomwashakanye<br>997 Ntibimureba
E.23a	E.23a Mu minsi 7 ishize, wakoze kino gikorwa iminsi ingahe?<br>*[Andika umubare w'iminsi]*<br>*Question relevant when: ${E.23} !='997'*<br>*Response constrained to: .<=7*	
E.23b	E.23b Ku munsi usanzwe, iyo wakoze kino gikorwa, ukoresha amasaha angahe?<br>*Gerageza wandi ighe mu icyikubo cya 15 (Urugero: niba umuntu avuze ko yakoresheje iminota 15 ku murimo andiko 0.25. burungushura ugeze ku minota 15, burungushura indi mibare yose kuza ku gikubo cya 15 kiri hafi aho.(urugero: niba ari iminota 35 uburungushure ushyire ku minota 30, hanyuma wandike amasaha 0.5) [Andika umubare w'amasaha]*<br>*Question relevant when: ${E.23} !='997' and ${E.23a} !='0'*<br>*Response constrained to: .<=24*	
E.24	E.24 Ukuyemo ubufasha bw'abantu baba hanze y'urugo rwanyu, ni gute wowe n'umugore wawe musaranganya ibi bikorwa? Kujya kwisoko guhahira urugo	1 Burigihe ni wowe<br>2 Kenshi ni wowe<br>3 Turasaranganya cyangwa tubikorera hamwe<br>4 Kenshi ni uwomwashakanye<br>5 Burigihe ni uwomwashakanye<br>997 Ntibimureba

E.24a	E.24a Mu minsi 7 ishize, wakoze kino gikorwa iminsi ingahe?<br>*[Andika umubare w'iminsi]*<br>*Question relevant when: ${E.24} !='997'*<br>*Response constrained to: .<=7*	
E.24b	E.24b Ku munsi usanzwe, iyo wakoze kino gikorwa, ukoresha amasaha angahe?<br>*Gerageza wandi ighe mu icyikubo cya 15 (Urugero: niba umuntu avuze ko yakoresheje iminota 15 ku murimo andiko 0.25. burungushura ugeze ku minota 15, burungushura indi mibare yose kuza ku gikubo cya 15 kiri hafi aho.(urugero: niba ari iminota 35 uburungushure ushyire ku minota 30, hanyuma wandike amasaha 0.5) [Andika umubare w'amasaha]*<br>*Question relevant when: ${E.24} !='997' and ${E.24a} !='0'*<br>*Response constrained to: .<=24*	
E.25	E.25 Ukuyemo ubufasha bw'abantu baba hanze y'urugo rwanyu, ni gute wowe n'umugore wawe musaranganya ibi bikorwa? Gusukura munzu no hanze yayo	1 Burigihe ni wowe<br>2 Kenshi ni wowe<br>3 Turasaranganya cyangwa tubikorera hamwe<br>4 Kenshi ni uwomwashakanye<br>5 Burigihe ni uwomwashakanye<br>997 Ntibimureba
E.25a	E.25a Mu minsi 7 ishize, wakoze kino gikorwa iminsi ingahe?<br>*[Andika umubare w'iminsi]*<br>*Question relevant when: ${E.25} !='997'*<br>*Response constrained to: .<=7*	
E.25b	E.25b Ku munsi usanzwe, iyo wakoze kino gikorwa, ukoresha amasaha angahe?<br>*Gerageza wandi ighe mu icyikubo cya 15 (Urugero: niba umuntu avuze ko yakoresheje iminota 15 ku murimo andiko 0.25. burungushura ugeze ku minota 15, burungushura indi mibare yose kuza ku gikubo cya 15 kiri hafi aho.(urugero: niba ari iminota 35 uburungushure ushyire ku minota 30, hanyuma wandike amasaha 0.5) [Andika umubare w'amasaha]*<br>*Question relevant when: ${E.25} !='997' and ${E.25a} !='0'*<br>*Response constrained to: .<=24*	
E.26	E.26 Ukuyemo ubufasha bw'abantu baba hanze y'urugo rwanyu, ni gute wowe n'umugore wawe musaranganya ibi bikorwa? Gusukurura ubwiyuhagiriro n'ubwiherero	1 Burigihe ni wowe<br>2 Kenshi ni wowe<br>3 Turasaranganya cyangwa tubikorera hamwe<br>4 Kenshi ni uwomwashakanye<br>5 Burigihe ni uwomwashakanye<br>997 Ntibimureba
E.26a	E.26a Mu minsi 7 ishize, wakoze kino gikorwa iminsi ingahe?<br>*[Andika umubare w'iminsi]*<br>*Question relevant when: ${E.26} !='997'*<br>*Response constrained to: .<=7*	
E.26b	E.26b Ku munsi usanzwe, iyo wakoze kino gikorwa, ukoresha amasaha angahe?<br>*Gerageza wandi ighe mu icyikubo cya 15 (Urugero: niba umuntu avuze ko yakoresheje iminota 15 ku murimo andiko 0.25. burungushura ugeze ku minota 15, burungushura indi mibare yose kuza ku gikubo cya 15 kiri hafi aho.(urugero: niba ari iminota 35 uburungushure ushyire ku minota 30, hanyuma wandike amasaha 0.5) [Andika umubare w'amasaha]*<br>*Question relevant when: ${E.26} !='997' and ${E.26a} !='0'*<br>*Response constrained to: .<=24*	
E.27	E.27 Ukuyemo ubufasha bw'abantu baba hanze y'urugo rwanyu, ni gute wowe n'umugore wawe musaranganya ibi bikorwa? Gutekera urugo	1 Burigihe ni wowe<br>2 Kenshi ni wowe<br>3 Turasaranganya cyangwa tubikorera hamwe<br>4 Kenshi ni uwomwashakanye<br>5 Burigihe ni uwomwashakanye<br>997 Ntibimureba
E.27a	E.27a Mu minsi 7 ishize, wakoze kino gikorwa iminsi ingahe?<br>*[Andika umubare w'iminsi]*<br>*Question relevant when: ${E.27} !='997'*<br>*Response constrained to: .<=7*	
E.27b	E.27b Ku munsi usanzwe, iyo wakoze kino gikorwa, ukoresha amasaha angahe?<br>*Gerageza wandi ighe mu icyikubo cya 15 (Urugero: niba umuntu avuze ko yakoresheje iminota 15 ku murimo andiko 0.25. burungushura ugeze ku minota 15, burungushura indi mibare yose kuza ku gikubo cya 15 kiri hafi aho.(urugero: niba ari iminota 35 uburungushure ushyire ku minota 30, hanyuma wandike amasaha 0.5) [Andika umubare w'amasaha]*<br>*Question relevant when: ${E.27} !='997' and ${E.27a} !='0'*<br>*Response constrained to: .<=24*	
E.28	E.28 Ukuyemo ubufasha bw'abantu baba hanze y'urugo rwanyu, ni gute wowe n'umugore wawe musaranganya ibi bikorwa? Gucunga amafaranga yinjira n'asohoka buri cyumweru	1 Burigihe ni wowe<br>2 Kenshi ni wowe<br>3 Turasaranganya cyangwa

		tubikorera hamwe<br>4 Kenshi ni uwomwashakanye<br>5 Burigihe ni uwomwashakanye<br>997 Ntibimureba
E.28a	E.28a Mu minsi 7 ishize, wakoze kino gikorwa iminsi ingahe?<br>*[Andika umubare w'iminsi]*<br>*Question relevant when: ${E.28} !='997'*<br>*Response constrained to: .<=7*	
E.28b	E.28b Ku munsi usanzwe, iyo wakoze kino gikorwa, ukoresha amasaha angahe?<br>*Gerageza wandi ighe mu icyikubo cya 15 (Urugero: niba umuntu avuze ko yakoresheje iminota 15 ku murimo andiko 0.25. burungushura ugeze ku minota 15, burungushura indi mibare yose kuza ku gikubo cya 15 kiri hafi aho.(urugero: niba ari iminota 35 uburungushure ushyire ku minota 30, hanyuma wandike amasaha 0.5) [Andika umubare w'amasaha]*<br>*Question relevant when: ${E.28} !='997' and ${E.28a} !='0'*<br>*Response constrained to: .<=24*	
E.29	E.29 Ukuyemo ubufasha bw'abantu baba hanze y'urugo rwanyu, ni gute wowe n'umugore wawe musaranganya ibi bikorwa? Gutashya inkwi	1 Burigihe ni wowe<br>2 Kenshi ni wowe<br>3 Turasaranganya cyangwa tubikorera hamwe<br>4 Kenshi ni uwomwashakanye<br>5 Burigihe ni uwomwashakanye<br>997 Ntibimureba
E.29a	E.29a Mu minsi 7 ishize, wakoze kino gikorwa iminsi ingahe?<br>*[Andika umubare w'iminsi]*<br>*Question relevant when: ${E.29} !='997'*<br>*Response constrained to: .<=7*	
E.29b	E.29b Ku munsi usanzwe, iyo wakoze kino gikorwa, ukoresha amasaha angahe?<br>*Gerageza wandi ighe mu icyikubo cya 15 (Urugero: niba umuntu avuze ko yakoresheje iminota 15 ku murimo andiko 0.25. burungushura ugeze ku minota 15, burungushura indi mibare yose kuza ku gikubo cya 15 kiri hafi aho.(urugero: niba ari iminota 35 uburungushure ushyire ku minota 30, hanyuma wandike amasaha 0.5) [Andika umubare w'amasaha]*<br>*Question relevant when: ${E.29} !='997' and ${E.29a} !='0'*<br>*Response constrained to: .<=24*	
E.30	E.30 Ukuyemo ubufasha bw'abantu baba hanze y'urugo rwanyu, ni gute wowe n'umugore wawe musaranganya ibi bikorwa? Gushaka ibiryo by'amatungo cyangwa kuragira amatungo yo murugo?	1 Burigihe ni wowe<br>2 Kenshi ni wowe<br>3 Turasaranganya cyangwa tubikorera hamwe<br>4 Kenshi ni uwomwashakanye<br>5 Burigihe ni uwomwashakanye<br>997 Ntibimureba
E.30a	E.30a Mu minsi 7 ishize, wakoze kino gikorwa iminsi ingahe?<br>*[Andika umubare w'iminsi]*<br>*Question relevant when: ${E.30} !='997'*<br>*Response constrained to: .<=7*	
E.30b	E.30b Ku munsi usanzwe, iyo wakoze kino gikorwa, ukoresha amasaha angahe?<br>*Gerageza wandi ighe mu icyikubo cya 15 (Urugero: niba umuntu avuze ko yakoresheje iminota 15 ku murimo andiko 0.25. burungushura ugeze ku minota 15, burungushura indi mibare yose kuza ku gikubo cya 15 kiri hafi aho.(urugero: niba ari iminota 35 uburungushure ushyire ku minota 30, hanyuma wandike amasaha 0.5) [Andika umubare w'amasaha]*<br>*Question relevant when: ${E.30} !='997' and ${E.30a} !='0'*<br>*Response constrained to: .<=24*	
E.31	E.31 Ukuyemo ubufasha bw'abantu baba hanze y'urugo rwanyu, ni gute wowe n'umugore wawe musaranganya ibi bikorwa: Gukora mu buhinzi-bworozi akorera amafaranga cyangwa ikindi gihembo?	1 Burigihe ni wowe<br>2 Kenshi ni wowe<br>3 Turasaranganya cyangwa tubikorera hamwe<br>4 Kenshi ni uwomwashakanye<br>5 Burigihe ni uwomwashakanye<br>997 Ntibimureba
E.31a	E.31a Mu minsi 7 ishize, wakoze kino gikorwa iminsi ingahe?<br>*[Andika umubare w'iminsi]*<br>*Question relevant when: ${E.31} !='997'*<br>*Response constrained to: .<=7*	
E.31b	E.31b Ku munsi usanzwe, iyo wakoze kino gikorwa, ukoresha amasaha angahe?<br>*Gerageza wandi ighe mu icyikubo cya 15 (Urugero: niba umuntu avuze ko yakoresheje iminota 15 ku murimo andiko 0.25. burungushura ugeze ku minota 15, burungushura indi mibare yose kuza ku gikubo cya 15 kiri hafi aho.(urugero: niba ari iminota 35*	

	uburungushure ushyire ku minota 30, hanyuma wandike amasaha 0.5) [Andika umubare w'amasaha]<br>*Question relevant when: ${E.31} !='997' and ${E.31a} !='0'*<br>*Response constrained to: .<=24*	
E.32	E.32 Ukuyemo ubufasha bw'abantu baba hanze y'urugo rwanyu, ni gute wowe n'umugore wawe musaranganya ibi bikorwa? Gukora mu buhinzi-bworozi nta amafaranga cyangwa ikindi gihembo akorera?	1 Burigihe ni wowe<br>2 Kenshi ni wowe<br>3 Turasaranganya cyangwa tubikorera hamwe<br>4 Kenshi ni uwomwashakanye<br>5 Burigihe ni uwomwashakanye<br>997 Ntibimureba
E.32a	E.32a Mu minsi 7 ishize, wakoze kino gikorwa iminsi ingahe?<br>*[Andika umubare w'iminsi]*<br>*Question relevant when: ${E.32} !='997'*<br>*Response constrained to: .<=7*	
E.32b	E.32b Ku munsi usanzwe, iyo wakoze kino gikorwa, ukoresha amasaha angahe?<br>*Gerageza wandi ighe mu icyikubo cya 15 (Urugero: niba umuntu avuze ko yakoresheje iminota 15 ku murimo andiko 0.25. burungushura ugeze ku minota 15, burungushura indi mibare yose kuza ku gikubo cya 15 kiri hafi aho.(urugero: niba ari iminota 35 uburungushure ushyire ku minota 30, hanyuma wandike amasaha 0.5) [Andika umubare w'amasaha]*<br>*Question relevant when: ${E.32} !='997' and ${E.32a} !='0'*<br>*Response constrained to: .<=24*	
E.33	E.33 Ukuyemo ubufasha bw'abantu baba hanze y'urugo rwanyu, ni gute wowe n'umugore wawe musaranganya ibi bikorwa? Gukora umurimo utari uw'ubuhinzi ubyara amafaranga cyangwa indi nyungu nko gukora muri butike cyangwa ikindi gikorwa kibyara inyungu	1 Burigihe ni wowe<br>2 Kenshi ni wowe<br>3 Turasaranganya cyangwa tubikorera hamwe<br>4 Kenshi ni uwomwashakanye<br>5 Burigihe ni uwomwashakanye<br>997 Ntibimureba
E.33a	E.33a Mu minsi 7 ishize, wakoze kino gikorwa iminsi ingahe?<br>*[Andika umubare w'iminsi]*<br>*Question relevant when: ${E.33} !='997'*<br>*Response constrained to: .<=7*	
E.33b	E.33b Ku munsi usanzwe, iyo wakoze kino gikorwa, ukoresha amasaha angahe?<br>*Gerageza wandi ighe mu icyikubo cya 15 (Urugero: niba umuntu avuze ko yakoresheje iminota 15 ku murimo andiko 0.25. burungushura ugeze ku minota 15, burungushura indi mibare yose kuza ku gikubo cya 15 kiri hafi aho.(urugero: niba ari iminota 35 uburungushure ushyire ku minota 30, hanyuma wandike amasaha 0.5) [Andika umubare w'amasaha]*<br>*Question relevant when: ${E.33} !='997' and ${E.33a} !='0'*<br>*Response constrained to: .<=24*	
E.34	E.34 Ukuyemo ubufasha bw'abantu baba hanze y'urugo rwanyu, ni gute wowe n'umugore wawe musaranganya ibi bikorwa? Gukora umurimo utari uw'ubuhinzi ubyara amafaranga cyangwa indi nyungu ukorerwa undi umuntu wo mumuryango atawuhemberwa?	1 Burigihe ni wowe<br>2 Kenshi ni wowe<br>3 Turasaranganya cyangwa tubikorera hamwe<br>4 Kenshi ni uwomwashakanye<br>5 Burigihe ni uwomwashakanye<br>997 Ntibimureba
E.34a	E.34a Mu minsi 7 ishize, wakoze kino gikorwa iminsi ingahe?<br>*[Andika umubare w'iminsi]*<br>*Question relevant when: ${E.34} !='997'*<br>*Response constrained to: .<=7*	
E.34b	E.34b Ku munsi usanzwe, iyo wakoze kino gikorwa, ukoresha amasaha angahe?<br>*Gerageza wandi ighe mu icyikubo cya 15 (Urugero: niba umuntu avuze ko yakoresheje iminota 15 ku murimo andiko 0.25. burungushura ugeze ku minota 15, burungushura indi mibare yose kuza ku gikubo cya 15 kiri hafi aho.(urugero: niba ari iminota 35 uburungushure ushyire ku minota 30, hanyuma wandike amasaha 0.5) [Andika umubare w'amasaha]*<br>*Question relevant when: ${E.34} !='997' and ${E.34a} !='0'*<br>*Response constrained to: .<=24*	
E.35	E.35 Ukuyemo ubufasha bw'abantu baba hanze y'urugo rwanyu, ni gute wowe n'umugore wawe musaranganya ibi bikorwa? Gusana inzu	1 Burigihe ni wowe<br>2 Kenshi ni wowe<br>3 Turasaranganya cyangwa tubikorera hamwe<br>4 Kenshi ni uwomwashakanye<br>5 Burigihe ni uwomwashakanye<br>997 Ntibimureba

E.35a	E.35a Mu minsi 7 ishize, wakoze kino gikorwa iminsi ingahe?<br>*[Andika umubare w'iminsi]*<br>*Question relevant when: ${E.35} !='997'*<br>*Response constrained to: .<=7*	
E.35b	E.35b Ku munsi usanzwe, iyo wakoze kino gikorwa, ukoresha amasaha angahe?<br>*Gerageza wandi ighe mu icyikubo cya 15 (Urugero: niba umuntu avuze ko yakoresheje iminota 15 ku murimo andiko 0.25. burungushura ugeze ku minota 15, burungushura indi mibare yose kuza ku gikubo cya 15 kiri hafi aho.(urugero: niba ari iminota 35 uburungushure ushyire ku minota 30, hanyuma wandike amasaha 0.5) [Andika umubare w'amasaha]*<br>*Question relevant when: ${E.35} !='997' and ${E.35a} !='0'*<br>*Response constrained to: .<=24*	
E.36	E.36 Ukuyemo ubufasha bw'abantu baba hanze y'urugo rwanyu, ni gute wowe n'umugore wawe musaranganya ibi bikorwa? Gusasa igitanda	1 Burigihe ni wowe<br>2 Kenshi ni wowe<br>3 Turasaranganya cyangwa tubikorera hamwe<br>4 Kenshi ni uwomwashakanye<br>5 Burigihe ni uwomwashakanye<br>997 Ntibimureba
E.36a	E.36a Mu minsi 7 ishize, wakoze kino gikorwa iminsi ingahe?<br>*[Andika umubare w'iminsi]*<br>*Question relevant when: ${E.36} !='997'*<br>*Response constrained to: .<=7*	
E.36b	E.36b Ku munsi usanzwe, iyo wakoze kino gikorwa, ukoresha amasaha angahe?<br>*Gerageza wandi ighe mu icyikubo cya 15 (Urugero: niba umuntu avuze ko yakoresheje iminota 15 ku murimo andiko 0.25. burungushura ugeze ku minota 15, burungushura indi mibare yose kuza ku gikubo cya 15 kiri hafi aho.(urugero: niba ari iminota 35 uburungushure ushyire ku minota 30, hanyuma wandike amasaha 0.5) [Andika umubare w'amasaha]*<br>*Question relevant when: ${E.36} !='997' and ${E.36a} !='0'*<br>*Response constrained to: .<=24*	
E.37	E.37 Mu minsi 7 ishize, wasabanye n'inshuti n'abandimwe iminsi ingahe?<br>*[Andika umubare w'iminsi]*<br>*Response constrained to: .<=7*	
E.37a	E.37a Ku munsi usanzwe, iyo wakoze kino gikorwa, ukoresha amasaha angahe?<br>*Gerageza wandi ighe mu icyikubo cya 15 (Urugero: niba umuntu avuze ko yakoresheje iminota 15 ku murimo andiko 0.25. burungushura ugeze ku minota 15, burungushura indi mibare yose kuza ku gikubo cya 15 kiri hafi aho.(urugero: niba ari iminota 35 uburungushure ushyire ku minota 30, hanyuma wandike amasaha 0.5) [Andika umubare w'amasaha]*<br>*Question relevant when: ${E.37} !='0'*<br>*Response constrained to: .<=24*	
E.38	E.38 Ugereranyije waryame amasaha angahe ku munsi mu minsi 7 ishize?<br>*Gerageza wandi ighe mu icyikubo cya 15 (Urugero: niba umuntu avuze ko yakoresheje iminota 15 ku murimo andiko 0.25. burungushura ugeze ku minota 15, burungushura indi mibare yose kuza ku gikubo cya 15 kiri hafi aho.(urugero: niba ari iminota 35 uburungushure ushyire ku minota 30, hanyuma wandike amasaha 0.5) [Andika umubare w'amasaha]*<br>*Response constrained to: .<=24*	
E.39	E.39 Wifuza ko wagira uruhare rungana iki mu mirimo yo murugo?<br>*MUSOMERE IBISUBIZO BYOSE BYATANZWE*	1 Nshimishijwe n'uruhare rwanjye ubu.<br>2 Nifuza kugira uruhare rurenzeho<br>3 Nifuza kugira uruhare rukeya.
E.8	E.8 Utekerezako uwo mwashakanye yifuzako wagira uruhare rungana gute mu mirimo yo mu rugo?<br>*MUSOMERE IBISUBIZO BYOSE BYATANZWE*	1 Yishimiye uruhare rwanjye ubu<br>2 Yakwifuje yuko nongera uruhare rwanjye<br>3 Yifuza ko nagabanya uruhare rwanjye<br>998 Simbizi
survey > part6 Igice cya 6. Inzoga<br>*Group relevant when: ${A.1} >=18 and ${A.7} ='2' or ${A.7} ='3'*		
note_6	Ngiye kukuganiriza ku ikoreshwa ry'inzoga. Nk'uko nakomeje kubikwizeza ibisubizo uribumpe biraba ibanga.	
N.F.1	N.F.1 Kuva mu mwaka ushize (kuva kwibazwa riheruka), umaze kunywa inzoga inshuro zingahe?<br>*MUSOMERE IBISUBIZO BYOSE BYATANZWE*	0 Nta na rimwe<br>1 Inshuro nke mu mwaka ushize<br>2 Rimwe buri mezi abiri (2)<br>3 Rimwe buri kwezi<br>4 Inshuro nke mu kwezi<br>5 Rimwe cyangwa kabiri mu cyumweru<br>6 Buri munsi cyangwa hafi ya buri munsi

		998	Simbizi
		999	Yanze gusubiza
N.F.2	N.F.2 Kuva mu mwaka ushize (kuva kwibazwa riheruka), ni kangahe wanyweye inzoga nyinshi kugeza aho usinda?<br>*ICYITONDERWA: SOMERA UBAZWA IBISUBIZO*<br>*Question relevant when: ${N.F.1} !='0'*	0	Ntibirabaho (aranywa ariko ntageza aho gusinda)
		1	Inshuro nke mu mwaka ushize
		2	Rimwe buri mezi abiri
		3	Rimwe mu kwezi
		4	Inshuro nke mu kwezi
		5	Rimwe cyangwa kabiri mu cyumweru
		6	Buri munsi cyangwa hafi ya buri munsi
		998	Simbizi
		999	Yanze gusubiza
F.3	F.3 Kuva mu mwaka ushize (kuva kwibazwa riheruka), ni kangahe…<br>*Question relevant when: ${N.F.1} !='0'*		
F.3.1	F.3.1 Wanyoye amacupa 5 y'inzoga cyangwa arenga ku inshuro imwe?<br>*Question relevant when: ${N.F.1} !='0'*	1	Kenshi
		2	Rimwe na rimwe
		3	Gake gashoboka
		0	Nta na rimwe
		999	Yanze gusubiza
F.3.2	F.3.2 Inzoga zakubujije gukora ibyo wagombaga gukora?<br>*Question relevant when: ${N.F.1} !='0'*	1	Kenshi
		2	Rimwe na rimwe
		3	Gake gashoboka
		0	Nta na rimwe
		999	Yanze gusubiza
F.3.3.	F.3.3. Wumvise wigaye kubera inzoga wanyoye?<br>*Question relevant when: ${N.F.1} !='0'*	1	Kenshi
		2	Rimwe na rimwe
		3	Gake gashoboka
		0	Nta na rimwe
		999	Yanze gusubiza
F.4	F.4 Wigeze ukomeretswa cyangwa undi umuntu yigeze gukomeretswa n'ibikorwa wakoze wasinze?<br>*Question relevant when: ${N.F.1} !='0'*	0	Oya
		1	Yego
		998	Simbizi
		999	Yanze gusubiza
survey > PART 7. Ihohoterwa rikorerwa hagati y'abashakanye n'irishingiye kugitsina<br>*Group relevant when: ${A.1} >=18 and ${A.7} ='2' or ${A.7} ='3'*			
note_7	Muri aka kanya ndagirango nkubazi ibibazo bike byerekeranye n'ihohoterwa aho utuye. Wibukeko nakubwiye ko ibyo turi buvugane ari ibanga.		
N.G.1	N.G.1 Igihe umugabo akubise umugore we, utekereza ko ari ngombwa ko abandi bo hanze babakiza?	0	Oya
		1	Yego
		999	Yanze gusubiza
N.G.2	N.G.2 Ku bwawe, umugabo afite impamvu yumvikana ituma akubita umugore we mu gihe:		
N.G.2.1	N.G.2.1 Ku bwawe, umugabo afite impamvu yumvikana ituma akubita umugore we mu gihe: atamwubashye	0	Oya
		1	Yego
		999	Yanze gusubiza
N.G.2.2	N.G.2.2 Ku bwawe, umugabo afite impamvu yumvikana ituma akubita umugore we mu gihe: yanze gukorana imibonano mpuzabitsina nawe	0	Oya
		1	Yego
		999	Yanze gusubiza
N.G.2.3	N.G.2.3 Ku bwawe, umugabo afite impamvu yumvikana ituma akubita umugore we mu gihe: asanze yajyaga amuca inyuma	0	Oya
		1	Yego
		999	Yanze gusubiza
N.G.2.4	N.G.2.4 Ku bwawe, umugabo afite impamvu yumvikana ituma akubita umugore we mu gihe: yirengagiza kwita ku mwana (abana)	0	Oya
		1	Yego
		999	Yanze gusubiza
N.G.2.5	N.G.2.5 Ku bwawe, umugabo afite impamvu yumvikana ituma akubita umugore we mu gihe: adakora imirimo yo mu rugo ku buryo bumushimisha	0	Oya
		1	Yego
		999	Yanze gusubiza

N.G.2.6	N.G.2.6 Ku bwawe, umugabo afite impamvu yumvikana ituma akubita umugore we mu gihe: umugore amushinja kumuca inyuma	0	Oya
		1	Yego
		999	Yanze gusubiza
N.G.2.7	N.G.2.7 Ku bwawe, umugabo afite impamvu yumvikana ituma akubita umugore we mu gihe: yakoresheje amafaranga atabanje kumugisha inama	0	Oya
		1	Yego
		999	Yanze gusubiza
N.G.2.8	N.G.2.8 Ku bwawe, umugabo afite impamvu yumvikana ituma akubita umugore we mu gihe: atakira abashyitsi mu rugo kuburyo bumushimisha	0	Oya
		1	Yego
		999	Yanze gusubiza
N.G.2.9	N.G.2.9 Ku bwawe, umugabo afite impamvu yumvikana ituma akubita umugore we mu gihe: Asanze atateguye ibyo kurya	0	Oya
		1	Yego
		999	Yanze gusubiza
G.3	G.3 Waba ufite umugabo w'inshuti yawe cyangwa muturanye uhohotera umugore we?	0	Oya
		1	Yego
		998	Simbizi
		999	Yanze gusubiza
G.4	G.4 Ese washobora kubaganiriza kugira ngo bahindure iyo imyitwarire?<br>*ICYITONDERWA: SOMERA UBAZWA IBISUBIZO*<br>*Question relevant when: ${G.3} ='1'*	0	Oya
		1	Yego, nabishobora
		2	Yego, ndabikora
		999	Yanze gusubiza
G.5	G.5 Wakoze iki ubonye umugabo w'inshuti yawe cyangwa umugabo muturanye ahohotera uwo bashakanye?<br>*Question relevant when: ${G.3} ='1' and ${G.4} ='2'*	1	Natabaye muri icyo gihe
		2	Naramuganirije nyuma yo gukora ihohotera
		3	Sinongeye kumuvugisha
		4	Nahamagaye polisi
		5	Nahamagaye abayobozi b'inzego z'ibanze
		6	Nahamagaye abaturanyi
		7	Ibindi
		999	Yanze gusubiza
N.G.3	N.G.3 Umugore ufite umugabo aramutse akubiswe n'umugabo we, wumva bikwiye ko yabibwira abandi?	0	Oya
		1	Yego
G.6	G.6 Mbese mu Rwanda haba amategeko ahana ihohoterwa rishingiye ku gitsina?	0	Oya
		1	Yego
		998	Simbizi
G.7	G.7 Ngiye kugusomera interuro zijyanye n'amategeko ajyanye n'ihohoterwa rishingiye ku gitsina, urajya umbwira niba ibikubiyemo ubyemera cyane, niba ubyemera, niba ntacyo wabivugaho, niba ubihakana, cyangwa niba utabyemera na gato.<br>*Question relevant when: ${G.6} ='1'*		
G.7.1	G.7.1 Amategeko ariho yorohereza abagore birenze urugero mu gutanga ibirego by'ihohoterwa bakorerwa n'abagabo<br>*Question relevant when: ${G.6} ='1'*	1	Ndabyemera cyane
		2	Ndabyemera
		3	Simbyemera kandi sinabihakana
		4	Simbyemera
		5	Simbyemera na gato
		999	Yanze gusubiza
G.7.2	G.7.2 Amategeko ariho arakarishye cyane<br>*Question relevant when: ${G.6} ='1'*	1	Ndabyemera cyane
		2	Ndabyemera
		3	Simbyemera kandi sinabihakana
		4	Simbyemera
		5	Simbyemera na gato
		999	Yanze gusubiza
G.7.3	G.7.3 Amategeko ariho ntakanganye<br>*Question relevant when: ${G.6} ='1'*	1	Ndabyemera cyane
		2	Ndabyemera
		3	Simbyemera kandi sinabihakana
		4	Simbyemera
		5	Simbyemera na gato
		999	Yanze gusubiza

G.7.4	G.7.4 Amategeko ariho arengera ku buryo buhagije uwahohotewe.<br>*Question relevant when: ${G.6} ='1'*	1	Ndabyemera cyane
		2	Ndabyemera
		3	Simbyemera kandi sinabihakana
		4	Simbyemera
		5	Simbyemera na gato
		999	Yanze gusubiza
G.7.5	G.7.5 Amategeko ariho arengera n'abagabo<br>*Question relevant when: ${G.6} ='1'*	1	Ndabyemera cyane
		2	Ndabyemera
		3	Simbyemera kandi sinabihakana
		4	Simbyemera
		5	Simbyemera na gato
		999	Yanze gusubiza
G.7.6	G.7.6 Amategeko ariho agira uruhare mu gukurura no gutiza umurindi amakimbirane yo mu rugo<br>*Question relevant when: ${G.6} ='1'*	1	Ndabyemera cyane
		2	Ndabyemera
		3	Simbyemera kandi sinabihakana
		4	Simbyemera
		5	Simbyemera na gato
		999	Yanze gusubiza
G.7.7	G.7.7 Abagore bitwaza amategeko mu gupyinagaza abagabo<br>*Question relevant when: ${G.6} ='1'*	1	Ndabyemera cyane
		2	Ndabyemera
		3	Simbyemera kandi sinabihakana
		4	Simbyemera
		5	Simbyemera na gato
		999	Yanze gusubiza

survey > PART 8. Imyumvire irebana n'uburinganire

Group relevant when: ${A.1} >=18 and ${A.7} ='2' or ${A.7} ='3'

note_8	Ibi bibazo bikurikira birabaza ibirebana n'imyumvire yawe ku mibanire y'umugabo n'umugore. Ngiye kugusomera interuro zikurikira, urajya umbwira niba ibikubiyemo ubyemera cyane, niba ubyemera, niba ntacyo wabivugaho, niba ubihakana, cyangwa niba utabyemera na gato.		
H.1	H.1 Inshingano zikomeye z’umugore ni ukwita ku rugo rwe no gutekera abagize umuryango we.	1	Ndabyemera cyane
		2	Ndabyemera
		3	Simbyemera kandi sinabihakana
		4	Simbyemera
		5	Simbyemera na gato
		999	Yanze gusubiza
H.2	H.2 Kwambika, gukarabya no kugaburira abana ni inshingano z’abagore.	1	Ndabyemera cyane
		2	Ndabyemera
		3	Simbyemera kandi sinabihakana
		4	Simbyemera
		5	Simbyemera na gato
		999	Yanze gusubiza
H.4	H.4 Umugabo niwe ugomba gufata icyemezo cya nyuma mu rugo rwe	1	Ndabyemera cyane
		2	Ndabyemera
		3	Simbyemera kandi sinabihakana
		4	Simbyemera
		5	Simbyemera na gato
		999	Yanze gusubiza
H.5	H.5 Umugabo agomba kubahwa nk'umutware w'urugo.	1	Ndabyemera cyane
		2	Ndabyemera
		3	Simbyemera kandi sinabihakana
		4	Simbyemera
		5	Simbyemera na gato

		999	Yanze gusubiza
H.10	H.10 Umugore w'umutima ntiyibaza kubyemezo by'umugabo we n'ubwo yaba atemeranywa nawe.	1	Ndabyemera cyane
		2	Ndabyemera
		3	Simbyemera kandi sinabihakana
		4	Simbyemera
		5	Simbyemera na gato
		999	Yanze gusubiza
H.11	H.11 Umugore niwe ufite inshingano zo kwirinda gusama.	1	Ndabyemera cyane
		2	Ndabyemera
		3	Simbyemera kandi sinabihakana
		4	Simbyemera
		5	Simbyemera na gato
		999	Yanze gusubiza
H.13	H.13 Ni byiza ko abagore bakora kugirango urugo rubone ibirutunga bihagije.	1	Ndabyemera cyane
		2	Ndabyemera
		3	Simbyemera kandi sinabihakana
		4	Simbyemera
		5	Simbyemera na gato
		999	Yanze gusubiza
H.16	H.16 Umugore ugendana agakingirizo “aba ari indaya."	1	Ndabyemera cyane
		2	Ndabyemera
		3	Simbyemera kandi sinabihakana
		4	Simbyemera
		5	Simbyemera na gato
		999	Yanze gusubiza
H.20	H.20 Birasanzwe kandi birakwiye ko abagabo bagira ububasha buruta ubw'abagore mu muryango.	1	Ndabyemera cyane
		2	Ndabyemera
		3	Simbyemera kandi sinabihakana
		4	Simbyemera
		5	Simbyemera na gato
		999	Yanze gusubiza
H.21	H.21 Ihohoterwa rishingiye ku gitsina (gufata ku ngufu) ribaho hagati y'abashakanye.	1	Ndabyemera cyane
		2	Ndabyemera
		3	Simbyemera kandi sinabihakana
		4	Simbyemera
		5	Simbyemera na gato
		999	Yanze gusubiza
H.22	H.22 Rimwe na rimwe umugore agomba gukubitwa.	1	Ndabyemera cyane
		2	Ndabyemera
		3	Simbyemera kandi sinabihakana
		4	Simbyemera
		5	Simbyemera na gato
		999	Yanze gusubiza
N.H.3	N.H.3 Iyo umugabo atetse cyangwa akoze isuku, kiba ari igisebo ku mugore we	1	Ndabyemera cyane
		2	Ndabyemera
		3	Simbyemera kandi sinabihakana
		4	Simbyemera
		5	Simbyemera na gato
		999	Yanze gusubiza
H.24	H.24 Umugabo niwe ufata icyemezo cya nyuma cy’ikoreshwa ry’amafaranga mu rugo.	1	Ndabyemera cyane
		2	Ndabyemera
		3	Simbyemera kandi

			sinabihakana
		4	Simbyemera
		5	Simbyemera na gato
		999	Yanze gusubiza
H.25	H.25 Umugore yakagombye kwihanganira ihohoterwa kugira ngo urugo rwe rudasenyuka.	1	Ndabyemera cyane
		2	Ndabyemera
		3	Simbyemera kandi sinabihakana
		4	Simbyemera
		5	Simbyemera na gato
		999	Yanze gusubiza
H.26	H.26 Niba amafaranga y'ishuli ari makeya, ni byiza kuyaharira abana b'abahungu.	1	Ndabyemera cyane
		2	Ndabyemera
		3	Simbyemera kandi sinabihakana
		4	Simbyemera
		5	Simbyemera na gato
		999	Yanze gusubiza
H.27	H.27 Iyo umwana akoze nabi agomba gukubitwa.	1	Ndabyemera cyane
		2	Ndabyemera
		3	Simbyemera kandi sinabihakana
		4	Simbyemera
		5	Simbyemera na gato
		999	Yanze gusubiza
H.29	H.29 Umugabo ashobora kwita ku bana kimwe nk'uko umugore abikora.	1	Ndabyemera cyane
		2	Ndabyemera
		3	Simbyemera kandi sinabihakana
		4	Simbyemera
		5	Simbyemera na gato
		999	Yanze gusubiza
H.30	H.30 Umugore yagombye kwihanganira ingorane zose ahura nazo mu rugo rwe (niko zubakwa).	1	Ndabyemera cyane
		2	Ndabyemera
		3	Simbyemera kandi sinabihakana
		4	Simbyemera
		5	Simbyemera na gato
		999	Yanze gusubiza
N.H.1	N.H.1 Umugabo aramutse abwiye inshuti ze ko afatanya n'umugore we mu gufata ibyemezo, inshuti ze ntizaba zikimwubashye	1	Ndabyemera cyane
		2	Ndabyemera
		3	Simbyemera kandi sinabihakana
		4	Simbyemera
		5	Simbyemera na gato
		999	Yanze gusubiza
N.H.2	N.H.2 Umugabo ugaragaye atetse cyangwa asukura inzu ye/urugo rwe abandi batuye muri ako gace baramukwena	1	Ndabyemera cyane
		2	Ndabyemera
		3	Simbyemera kandi sinabihakana
		4	Simbyemera
		5	Simbyemera na gato
		999	Yanze gusubiza

survey > PART 9. Ibikorwa by'ubukangurambaga n'amahuriro atanga ubufasha

Group relevant when: ${A.1} >=18 and ${A.7} ='2' or ${A.7} ='3'

note_9	Turi hafi gusoza ikiganiro cyacu.		
I.9	I.9 Ngiye kugusomera interuro zikurikira, urajya umbwira niba ibikubiyemo ubyemera cyane, niba ubyemera, niba ntacyo wabivugaho, niba ubihakana, cyangwa niba utabyemera na gato.		
I.9.1	I.9.1 Ndi intangarugero aho ntuye	1	Ndabyemera cyane
		2	Ndabyemera

		3 Simbyemeye kandi simbihakanye<br>4 Simbyemera<br>5 Simbyemera na gato
I.9.2	I.9.2 Numva ubuzima bwanjye bufitiye akamaro abandi	1 Ndabyemera cyane<br>2 Ndabyemera<br>3 Simbyemeye kandi simbihakanye<br>4 Simbyemera<br>5 Simbyemera na gato
I.9.3	I.9.3 Muri rusange mfite byinshi byo kwishimira	1 Ndabyemera cyane<br>2 Ndabyemera<br>3 Simbyemeye kandi simbihakanye<br>4 Simbyemera<br>5 Simbyemera na gato
N.I.9.4	N.I.9.4 Numva ndi umugabo ukwiye	1 Ndabyemera cyane<br>2 Ndabyemera<br>3 Simbyemeye kandi simbihakanye<br>4 Simbyemera<br>5 Simbyemera na gato
N.I.9.5	N.I.9.5 Numva ndi umubyeyi mwiza<br>*Question relevant when: ${partcipant_group} ='2'*	1 Ndabyemera cyane<br>2 Ndabyemera<br>3 Simbyemeye kandi simbihakanye<br>4 Simbyemera<br>5 Simbyemera na gato
I.1	I.1 Kuva mu mwaka ushize (kuva kwibazwa riheruka), waba warigeze witabira ibikorwa by'aho utuye cyangwa aho ukorera bivuga ku ihotera abagabo bakorera abagore?<br>*Question relevant when: ${partcipant_group} ='2'*	0 Oya<br>1 Yego<br>999 Yanze gusubiza
I.2	I.2 Kuva mu mwaka ushize (kuva kwibazwa riheruka), aho utuye cyangwa ukorera, waba warigeze kwitabira ibikorwa binyuranye bigamije gukangurira abagabo kugira uruhare mu buzima bw'abana babo?<br>*Question relevant when: ${partcipant_group} ='2'*	0 Oya<br>1 Yego
N.I.1	N.I.1 Kuva mu mwaka ushize (kuva kwibazwa riheruka), waba warigeze witabira gahunda zerekeranye n'ibiganiro k'ubuzima bw'ababyeyi aho utuye cyangwa aho ukorera?<br>*Question relevant when: ${partcipant_group} ='2'*	0 Oya<br>1 Yego
I.7	I.7 Hari umuntu uzi wigize ajya cyangwa wigishaga mu matsinda ya BANDEBEREHO?<br>*Question relevant when: ${partcipant_group} ='2'*	0 Oya<br>1 Yego, umuntu 1<br>2 Yego, nzi abantu benshi<br>998 Simbizi
I.8	I.8 Uwo muntu cyangwa abo bantu mupfana iki?<br>*HITAMO IBISUBIZO BYOSE BISHOBOKA*<br>*Question relevant when: ${partcipant_group} ='2' and ${I.7} !='0' and ${I.7} !='998'*	1 Dufitanye isano ya hafi<br>2 Ni inshuti cyane<br>3 Mwene wacu wa kure<br>4 Ni umuntu ntazi neza<br>5 Umuturanyi<br>6 Undi muntu
I.8.1	I.8.1 Waba warigeze usaba ubujyanama kuva kuri umwe mubigisha ba RWAMREC Bandebereho cyangwa undi muntu witabiriye ayo matsinda?<br>*Question relevant when: ${partcipant_group} ='2' and ${I.7} !='0' and ${I.7} !='998'*	0 Oya<br>1 Yego<br>998 Simbizi
N.I.2	N.I.2 Ni inshuro zingahe umaze gusaba ubujyanama umwe mu bakangurambaga ba Bandebereho ya RWAMREC cyangwa abagize itsinda?<br>*Question relevant when: ${partcipant_group} ='2' and ${I.7} !='0' and ${I.8.1} ='1' and ${I.7} !='998'*	1 Rimwe<br>2 Inshuro nkeye (2-5)<br>3 Inshuro nyinshi (zirenze 5)
note_NI3	Ngiye kukubaza ibibazo bike bijyanye n'ibikorwa waba waritabiriye kuva Kuva mu mwaka ushize (kuva kwibazwa riheruka).<br>*Question relevant when: ${partcipant_group} ='1'*	
N.I.3	N.I.3 Kuva mu mwaka ushize (kuva kwibazwa riheruka), wigeze witabira igikorwa icyo ari cyo cyose kitari ibiganiro by'ababyeyi Bandebereho bya RWAMREC, cyaba kivuga ku ihohotera abagabo bakorera abagore?<br>*Question relevant when: ${partcipant_group} ='1'*	0 Oya<br>1 Yego
N.I.4	N.I.4 Kuva mu mwaka ushize (kuva kwibazwa riheruka), wigeze witabira igikorwa icyo ari cyo cyose kitari ibiganiro	0 Oya

	by'ababyeyi Bandebereho bya RWAMREC, cyaba kivuga ku ruhare rw'abagabo mu mibereho y'abana babo?<br>*Question relevant when: ${partcipant_group} ='1'*	1 Yego
N.I.5	N.I.5 Kuva mu mwaka ushize (kuva kwibazwa riheruka), wigeze witabira igikorwa icyo ari cyo cyose kitari ibiganiro by'ababyeyi Bandebereho bya RWAMREC, cyaba kivuga ku buzima bw'umubyeyi?<br>*Question relevant when: ${partcipant_group} ='1'*	0 Oya<br>1 Yego
N.I.10	N.I.10 Wigeze usangiza undi uwo ari we wese ibyo wigiye mu biganiro by'ababyeyi Bandebereho bya RWAMREC?<br>*Question relevant when: ${partcipant_group} ='1'*	0 Oya<br>1 Yego
N.I.11	N.I.11 Ni nde wasangije ibyo wize?<br>*MARK ALL THAT APPLY*<br>*Question relevant when: ${partcipant_group} ='1' and ${N.I.10} ='1'*	1 Umugore wanjye<br>2 Abana banjye<br>3 Abo dufitanye isano ya hafi<br>4 Ni inshuti cyane<br>5 Mwene wacu wa kure<br>6 Ni umuntu ntazi neza<br>7 Umuturanyi<br>8 Mu ruhame (urugero, natanze ubuhamya mu muganda)
I.13	I.13 Mugihe cy’umwaka umwe n’igice, witabiriye ubushakashatsi bwacu kugeza inshuro 3. Mubibazo nakubajije bimwe byari byoroshye n’ibindi byari bikomeye. Ni gute kuvuga kuri ibi bintu byatumye wiyumva?	1 Neza<br>2 Nabi<br>3 Si neza kandi sinanabi
end_note	Uyu niwo musozo w'ikiganiro twagiranye.<br><br>Wakoze cyane ku bw'uyu mwanya tumaranye.<br><br>Nk'uko nabikubwiye, ndakwizeza ko ibisubizo byawe byose ari ibanga kandi ko ntaho izina ryawe rizagaragara.<br><br>ASK THE PARTICIPANT IF HE WANTS THE REFERRAL SHEET OF SERVICES/ SUPPORT ORGANIZATIONS IN HIS SECTOR. PROVIDE IT TO THE RESPONDENT IF HE WANTS IT.	
