# Supplementary material for: Gender-transformative Bandebereho couples’ intervention to promote male engagement in reproductive and maternal health and violence prevention in Rwanda: Findings from a randomized controlled trial
Source: PLoS One. 2018 Apr 4;13(4):e0192756. doi: 10.1371/journal.pone.0192756 (PMC5884496; doi:10.1371/journal.pone.0192756)
Supplement: S5 File — (PDF) [file pone.0192756.s005.pdf]

# Bandebereho Study Protocol

**This protocol was submitted to the Rwanda National Ethics Committee on August 28, 2014. The protocol was prepared following the specific guidelines for submission for ethical approval from the Rwanda National Ethics Committee. Additional details are available in the clinicaltrials.gov registration.**

**Study title:**

*Bandebereho* Father Groups: a randomized control trial to assess the impact of maternal, newborn and child health group education for new and expecting fathers

**Principal Investigators:**


- Dr. Fidele Ngabo: Maternal and Child Health Department, Ministry of Health, Kigali, Rwanda*
- Shamsi Kazimbaya: Rwanda Men's Resource Center, Kigali, Rwanda
- Kate Doyle, Ruti Levtov, and Gary Barker: Promundo-US, Washington, DC, United States


**Principle Collaborating Institutions:**

- Rwanda Ministry of Health, MCH unit
- Rwanda Men's Resource Center (RWAMREC)
- Promundo-US

*Replacement of PI: During the study, Dr. Fidele Ngabo was dismissed by the Ministry of Health. He was replaced by Dr. Felix Sayinzoga, who subsequently became part of the research team.

# Study Protocol

**Study title:** *Bandebereho* Father Groups: a randomized control trial to assess the impact of maternal, newborn and child health group education for new and expecting fathers


## Principal Investigators:
Dr. Fidele Ngabo: Maternal and Child Health Department, Ministry of Health, Kigali, Rwanda
Shamsi Kazimbaya: Rwanda Men's Resource Center, Kigali, Rwanda
Kate Doyle, Ruti Levtov, and Gary Barker: Promundo-US, Washington, DC, United States

## Principle Collaborating Institutions:
Rwanda Ministry of Health, MCH unit
Rwanda Men's Resource Center (RWAMREC)
Promundo-US


## Summary of study


This study seeks to assess the impact of the MenCare+/Bandebereho fathers group education intervention on men's attitudes towards, and participation in, sexual and reproductive health, maternal, newborn and child health, and equitable and non-violent relationships with their children and partners. The MenCare+/ Bandebereho program is a multi-component comprehensive intervention being implemented by the Rwanda Men's Resource Center (RWAMREC) and Promundo-US, in collaboration with the Rwanda Ministry of Health and local authorities in Karongi, Musanze, Nyaruguru and Nyaruguru districts. The proposed study will focus on a 15-session group education intervention targeting young fathers, and assess its impact through a randomized control trial design with an intervention arm of men participating in the father groups and a control arm of men who do not participate in intervention. The study will collect quantitative and qualitative data over a period of approximately one year.


## Acronyms

| | |
|---|---|
| IMAGES | International Men and Gender Equality Survey |
| MCH | Maternal and Child Health |
| MNCH | Maternal, Newborn and Child Health |
| RWAMREC | Rwanda Men's Resource Center |
| SRH | Sexual and Reproductive Health |
| SRHR | Sexual and Reproductive Health and Rights |

## 1. Background

Improving maternal, newborn and child health (MNCH) and reducing unmet need for family planning are key priorities of the Government of Rwanda. The country aims to reduce maternal-related morbidity and mortality by increasing the number of women attending antenatal care and giving birth in health facilities, and to increase the number of women using modern contraceptive methods. National strategies – such as the Community Health and Family Planning policies – recognize that male involvement in MNCH, reproductive

health and family planning is critical to achieving these goals. These policies emphasize the importance of promoting gender equality in community health programs, and the need for men to share responsibilities with women and be more involved in prevention and care activities (1-3).

MenCare+ is a three-year project to engage men in maternal, newborn and child health and in sexual and reproductive health. The project, which began implementation in 2013 in collaboration with the Rwandan Ministry of Health, supports communities in achieving national goals related to MNCH, reproductive health and family planning. A core component of the project are fathers groups, which engage new and expectant fathers, aged between 21-35 years, in group education activities at the community level.

The objectives of the fathers group are: to promote men's roles as caregiving partners in maternal, newborn and child health, including when planning to have a child, during pregnancy, labor and delivery, and after the child is born; to promote gender equality within couples, including the equitable division of caregiving and domestic work; and to prevent violence against women and promote healthy and happy relationships. During the first cycle of fathers groups, implemented in the first half of 2014, 576 men and their female partners participated in the groups. The anecdotal results of the first cycle of fathers groups are very encouraging, but no systematic impact evaluation has yet been done. A second cycle of fathers groups will commence in October 2014. The impact evaluation is planned to coincide with the third cycle of fathers groups, in early 2015.

The non-violent involvement of fathers and male caregivers during the prenatal and postnatal periods is vital to the health and well-being of mother and child. Studies have shown that mothers whose male partners accompanied them to prenatal care visits were more likely to attend most or all of their visits in the first trimester (4) and beyond (5). In addition, women whose partners accompanied them during labor report more positive birth experiences (6, 7) including a shorter duration of labor, and lower levels of pain (8). Men's active engagement as allies in MNCH can also contribute to ensuring a higher quality of care for their partners.

MenCare+ fathers groups operate in collaboration with local health facilities to engage men in supporting women's attendance at all four antenatal care visits and delivery in health facilities. Expectant fathers, as well as their partners, are provided with information on what to expect during pregnancy and how to ensure the health of the mother and fetus. Group discussions and activities encourage men to think of ways that they can support their partners during pregnancy, including accompanying their wives to ANC visits and during childbirth. Men are also encouraged be active caregivers and to bond with their newborns, as well as to be supportive partners to their wives, for example, by helping them to breastfeed.

The fathers groups also encourage male involvement in family planning, which includes condom use and vasectomy, as well as men encouraging and supporting their female partners to use contraceptives. Research has shown that men often control fertility decisions and strongly influence couples' childbearing behavior (9-11). Rwanda's family planning policy recognizes the need to improve women's decision-making power to enable women to decide freely when to have a child, family size, and birth spacing. In Rwanda, use of contraceptive methods is lower among women who do not participate in any household decisions (12). In addition, numerous studies have found that couple communication regarding family planning is positively correlated with contraceptive use (13-15).

The fathers groups aim to increase men and women's knowledge, acceptability and communication regarding contraceptive methods. Health professionals from local health centers provide participants with

accurate information about the different contraceptive methods and dispel any misconceptions or fears participants may hold. Couples participate in activities designed to improve communication about contraceptive use, family size and birth spacing. The group sessions provide information men and women require to make healthy choices, and open spaces to encourage communication and improvement in women's decision-making power regarding contraceptives.

The World Health Organization has recommended that efforts to improve maternal health should also include measures to reduce partner-violence against women (16). This recommendation is mirrored by national health policies, which acknowledge intimate partner violence as a barrier to improving maternal and child health. The fathers groups promote men's involvement as equitable and non-violent partners by increasing awareness of violence and knowledge of the law, and awareness of how gender inequality perpetuates intimate partner violence. Activities encourage men to reflect on the critical role of men in ending violence against women and how to be actively involved in raising their children without violence.

The MenCare+ fathers groups promote men's involvement in MNCH and SRHR, and their equal participation in caregiving and domestic work. Recent studies confirm that engaging men in caregiving can have a range of positive health and economic benefits for women (17), children (18) and men themselves (19). Quantitative studies from six countries that participated in the International Men and Gender Equality Survey (IMAGES) also affirm that men who were more involved as fathers felt more satisfied with their lives, often took better care of their own health (20). The IMAGES studies also found that when men carried out a more equitable share of the care work within the home, their wives reported more life and relationship satisfaction, including sexual satisfaction. (20).

Conducting a randomized control trial of the Bandebereho fathers groups provides an incredible opportunity to rigorously evaluate a male engagement strategy for increasing men's involvement in MNCH and SRH within the context of Rwanda. The findings of the evaluation can provide evidence to support the scaling-up and extension of existing fathers groups, as well as advocacy for inclusion of the father group methodology within national and district MNCH initiatives. The experience and findings from Rwanda can also provide an example for other countries interested in promoting men's greater involvement in maternal, newborn and child health, and in promoting gender equality.

## 2. Aim and objectives

Men's greater involvement in maternal, newborn and child health and sexual and reproductive health are priorities of the Government of Rwanda, yet little evidence is currently available on the most effective strategies to fully engage men as equitable, non-violent partners and caregivers. Our study seeks to assess the impact and effectiveness of an existing group education intervention for new and expecting fathers in order to provide evidence and support for scale-up of such interventions within Rwanda.

The study aims to assess the impact of facilitated group education with fathers on a range of outcomes including: men's participation in reproductive health, family planning and maternal, newborn and child health; gender attitudes and household dynamics; and violence. The purpose of the study is to compare outcomes between individuals randomly assigned to intervention and control groups, as well as to assess whether any changes that occurred are sustained over time.

## 3. Methods

*3.1. Study description*

This study seeks to evaluate the impact of the Bandebereho fathers' group intervention on the participating men and their families with both quantitative and qualitative methods.

*3.2. Study design*

The study is designed as a randomized control trial with two arms: intervention and control. Study participants (both intervention and control) will be surveyed at three time points: prior to beginning the intervention (baseline), at the end of the 15-week intervention (midline), and at a point 6 months (endline) following completion of the intervention.[1]

To validate and supplement the results from men, we will also survey a sample of the participating men's female partners (wives or girlfriends) at the endline.[2] Finally, we plan to conduct qualitative in-depth interviews with a sub-sample of the men and their partners to better understand gendered household dynamics.

Should additional funds become available, we may continue to follow the men and their families for additional time.

*3.3. Study site*

The study site includes communities in 16 sectors where the MenCare+/Bandebereho project operates in collaboration with the Ministry of Health and local authorities: Bwishyura, Gishyita, Murambi, and Rubengera (Karongi district); Kinigi, Muhoza, Muko, and Remera (Musanze district); Cyahinda, Kibeho, Nyagisozi, and Rusenge (Nyaruguru district); and Kigabiro, Munyaga, Munyiginya, and Mwulire, (Rwamagana district).

*3.4. Study population*

We plan to survey approximately 1,200 men, evenly split between intervention and control groups. The target group for the study is men aged 21-35, who are expecting a child or have children under the age of 5. We will also survey a subsample of women who are the partners of study participants.

Study participants will be selected in the following manner: RWAMREC works closely with sector authorities and community health workers on the MenCare+/Bandebereho project, to identify men who belong to the target population. In each sector, community health workers will generate a list of eligible participants, and project staff will verify this eligibility. Eligible men will be asked to participate in the study and provide oral consent for participation; those who consent will then be randomized into intervention and control groups.

*3.5. Proposed intervention if interventions study*

The MenCare+/Bandebereho fathers' group education engages new and expecting fathers in 15 weeks of group discussion and activities. A trained community facilitator leads a group of 12 men, using the curriculum developed by RWAMREC and Promundo and approved by the Rwandan Ministry of Health.

[1] Follow-up survey timing will be ultimately decided based on logistical and funding possibilities.
[2] Pending logistical and funding possibilities, female partners may be interviewed at baseline as well.

Each session lasts approximately 3 hours and takes place within district and sector offices, schools, and other community venues. The sessions focus on men's role in maternal, newborn and child health, including pregnancy and delivery, family planning, sharing household responsibilities, stopping intimate partner violence, conflict resolution, family budgeting, and gender equality. Men's wives participate in 6-8 sessions to strengthen couple communication on family planning, raising children, and household budgeting.

The 15 group education sessions are designed specifically for the Rwandan context in order to address some of the socio-cultural barriers to men's greater involvement as fathers and caregivers in sexual and reproductive health, and maternal, newborn and child health. During the development of the project, we consulted the local authorities, community members and Ministry of Health in order to create activities that can help to challenge some cultural barriers – e.g. men are seen as the financial providers, while women are caregivers – that prevent men from being more involved in their children's lives. The sessions encourage men to take a greater role in caregiving work by enabling them, and their female partners, to understand the benefits it brings to children, women and men themselves.

The sessions also address women's resistance to men's participation in these areas, by creating dialogue between men and women and encouraging shared responsibilities. In addition, the group education sessions are accompanied by community campaign activities to create an enabling and supportive environment for men's involvement in pregnancy, family planning and in children's health, within the larger community. The group education sessions are summarized below:

*Fathers' Group Education Module:*

**Session 1: Gender Equality:** Men discuss existing gender attitudes and values in the community and learn about the difference between sex and gender. Men reflect on how gender norms influence men and women's roles as parents.

**Session 2: Becoming a Father:** Men hear testimony from an involved father in the community and share their own expectations and worries about becoming a father. Men reflect on the benefits of being an involved father for children, women and men.

**Session 3: Pregnancy:** Men and their female partners learn about pregnancy from a health provider, including the changes that happen during pregnancy, warning signs, fistula prevention, sex during pregnancy, nutrition, etc. from an experienced health care provider.

**Session 4: Supporting your Pregnant Partner:** Men and their female partners brainstorm some of the ways that men can support women to have a healthy pregnancy. Participants reflect on the importance of women attending all four antenatal care visits and why men should attend too.

**Session 5: Birth:** Men and their female partners role play what happens in the delivery room and discuss how men can be present at birth and support their partners. Parents to be learn about how to bond with their child directly after birth.

**Session 6: Family Planning:** Men and their female partners learn about the different contraceptive methods from an experienced health care provider and have the opportunity to ask questions and address fears and misinformation. Couples discuss the benefits of planning their family and how to communicate about family planning and contraceptive use.

**Session 7: Caring for Baby:** Men learn tangible skills about how to care for a baby (holding, dressing and bathing a baby) and discuss some of the prevailing gender norms that hinder men's participation in caregiving. Men also reflect on how to support their partners in breastfeeding.

**Session 8: Our Parents' Impact:** Men reflect on the impact that their own fathers and mothers had on them growing up. Men identify some of the positive aspects of their own parents that they want to repeat with their own children, and some of the negative aspects they want to leave behind.

**Session 9: Identifying Violence:** Men discuss what 'violence' means to them and the forms it takes in the community. Participants then reflect on their own experiences and use of violence.

**Session 10: Gender-based Violence:** Men have the opportunity to learn about the GBV law and ask questions of a police officer from the Gender Desk in the community. Men also discuss how they can break the silence about violence in their own communities and help those in need.

**Session 11: Resolving Conflict:** Men discuss the characteristics of a healthy relationship and negative and positive methods of resolving conflict. Men learn and role play ways to control their anger and discuss how they can avoid or resolve conflict in their relationships.

**Session 12: Alcohol and Drugs**: Men reflect on some of the negative impacts of alcohol abuse and its prevalence in the community. Participants also discuss other types of drugs, and discuss the risk and harms associated with drug or alcohol abuse and what protective factors they can use.

**Session 13: Raising Children:** Men and their female partners discuss their shared dreams for their children's future and the ways that physical punishment can hinder those dreams from becoming a reality. Men and women discuss tangible positive discipline skills they can use with their children.

**Session 14: Shared Responsibilities:** Men and their female partners discuss the gendered division of labor in the home and reflect on ways that they can share household responsibilities. Men and women discuss financial decision-making in the home and practice making a household budget.

**Session 15: Reflection:** Men reflect on what they have learned in the group sessions and how they can support each other to be involved, caring fathers and role models in the community.

Due to the specialized content of certain sessions, local experts from the community facilitate three of the 15 group sessions: Session 3: Pregnancy; Session 6: Family Planning; and Session 10: Sexual and Gender-based violence). Health providers from the local health facility working in antenatal care and family planning facilitate sessions 3 and 6, respectively. These health providers are identified and invited in collaboration with the District Health Officer and Director of the District Hospital, based on their knowledge and specific area of work.

For session 10, which provides explanation on the national law against gender-based violence, experts on the GBV law are invited to lead the session. These experts include police officers from the Gender Desk, chosen for their specific knowledge of the GBV law, as well as lawyers from the "Maison d'Assistance a la Justice" or the "Etat Civil" at Sector level, who have experience assisting community members in justice and legal-related issues. Group participants are also provided with a copy of the GBV law.

*3.6. Main exposures and/or confounders and/or outcomes to be measured*

i) Exposures: Exposure in this study consists of men's attendance of the Bandebereho fathers' groups. We will closely monitor the number of sessions attended by each of the intervention group participants in order to be able to assess the impact of the intensity of exposure (or "dosage") on the expected outcomes.

ii) Confounders: As this study is planned as a randomized control trial, we anticipate that any confounders will be evenly spread across the intervention and control groups, allowing us to attribute change (if any) to

the intervention itself. However, we recognize that "spill-over" between intervention and control participants is possible. As a rough measure of spill-over, we will measure contact between participants and non-participants.

iii) Outcomes to be measured:
Outcomes to be measured by this study, in both the quantitative and qualitative components, include:

- *Men's participation in reproductive health, family planning and maternal and child health,* specifically: attendance of antenatal care visits, presence at birth, support for breastfeeding, access to and communication about family planning, and attitudes about men's participation in this domain.
- *Gender attitudes and household dynamics,* specifically: attitudes about gender norms, men's participation in caregiving and domestic tasks, household decision-making, and couple communication and satisfaction.
- *Violence, risk behaviors, and men's health and well-being,* specifically: intimate partner violence, violence and harsh discipline against children, attitudes about violence, anger management or conflict resolution skills.

In addition, the survey will collect demographic information about participants, including age, marital status, level of education, and number of children). In the analysis, these variables will be used as control variables. They will also be used as quality control measures for eligibility procedures and data collection (for example, to ensure that all men participating in the study have a partner), and to track changes in family structure over time (for example, men whose status changes from living together to legally married).

*3.7 Inclusion of control participants in the intervention following positive results*

Following the analysis of the results from this study, if it is found that the study has positive, meaningful impact, control participants will be given priority to participate in subsequent cycles of MenCare+/Bandebereho fathers' groups, contingent on additional funds to continue the program. RWAMREC and its partners are actively seeking additional funds to continue and scale-up the program; positive results from an impact evaluation will substantially enhance our opportunities to do so.

## 4. Selection of study population

*4.1. Inclusion criteria*

Inclusion criteria for men in the study include: being between the ages of 21-35, expecting a child or having one or more children under the age of five, living in a stable partnership, and residing in the study site. For women, the criteria are simply being the partner (wife or girlfriend) of one of the men selected for the study.

*4.2. Exclusion criteria*

Men will be excluded from the study if they do not meet the inclusion criteria detailed above, or if they have previously participated in any Bandebereho group. Women will be excluded from the study if their partner is not selected for the study.

*4.3. Sampling*

As described in section 3.4, men will be sampled from lists of eligible and willing participants generated by district authorities and community health workers, and verified by project staff. We will then randomize the

eligible men into the intervention or control groups.

A subsample of men and their partners will be asked to also participate in qualitative interviews. Sampling for the qualitative component will be purposive, selecting men based on specific characteristics or experiences (e.g. men who are expecting their first child, men who report changing their use of violence, etc.).

*4.4. Randomization if randomized trial*

Randomization into the intervention will occur at the individual level. Random assignment from the list of eligible and willing participants will be done by computer to ensure fairness and accountability for assignment to the intervention and control groups.

## 5. Study procedures

*5.1. Procedures at enrolment*

During enrolment, all identified participants will be approached by project staff who will explain the study and intervention, including the possible costs and benefits of participation. After confirming eligibility, participants will be asked to consent to the study, and will be given a copy of the consent form. Written consent will be sought, although participants may provide consent with a fingerprint. In cases of low literacy among some of the participants, the participant can choose to have a witness accompany him or her during the enrolment procedure and sign on his or her behalf. The witness will not participate in the study.

Identifying information including name, phone number if available, village of residence, etc. will be collected to allow for later location of participants for follow-up.

*5.2. Follow-up if cohorts study or trial*

We plan two follow-ups to our baseline survey: at the end of the intervention period (15 weeks), and 3 to 6 months post intervention. Both intervention and control participants will be surveyed at all 3 points.

*5.3. Measurement of exposures and confounders*

To measure exposure to the intervention, we will be closely monitoring each intervention participant's attendance in the fathers' groups. In addition, we will also be monitoring the exposure of the control group to the fathers' workshops through questions measuring contact with participants in the questionnaire, and through monitoring of other relevant activities in the districts, such as new grassroots fathers' groups. During statistical analysis, we will use these measurements as control variables, as relevant.

*5.4. Measurement of outcomes*

The outcomes (described in section 3.6iii) will be collected primarily through survey questionnaires, administered by a team of sex-matched interviewers trained in survey administration and research ethics. The questionnaire was developed based on recognized and validated measures, including from the International Men and Gender Equality Survey (IMAGES) which was conducted by RWAMREC in Rwanda in 2010. All measures will be pilot tested to ensure reliability and validity in the context of Rwanda. Given time constraints of implementation, we request permission to make minor changes to the tools based on pre-testing prior to data collection. Changes may include deletion of some items or rewording of items for clarity and relevance. No new domains and no entirely new instruments will be used without prior approval.

Qualitative research will focus on deepening our understanding and contextualization of the quantitative results and mechanisms of change. Interviews will be conducted by trained and experienced qualitative researchers. While the interviewers will focus on the various outcome domains described above, they will not follow a strict interview guide, but instead draw out study participants' experiences and attitudes.

*5.5. Laboratory methods if the study has a lab component*

No laboratory methods will be used in this study.

*5.6. Sample size*

We plan to survey a total of approximately 1,200 men: 550-600 intervention participants and an approximately equal number of control participants. A power analysis conducted specifically for this study suggests that we will have sufficient power to detect statistically significant differences across several outcomes of interest with this sample size. The sample size for women partners will be contingent on budget considerations, but is currently estimated at approximately 200 participants. We also estimate approximately 40 qualitative interviews. If additional funds become available, we hope to also evaluate another cycle of the intervention, which would double the sample size.

*5.7. Data Management*

Data collection will likely occur on pre-programmed electronic tablets. Tablet-based data collection simplifies data collection for interviewers and improves data quality. Data will be collected on password protected individual devices, and uploaded to a general server on a regular basis by the field supervisors. Once the data are received by the server, the interview will be erased from the specific device.

Each interview will be assigned a unique ID, and identifying information (name, contact information) linked to the ID will be kept separately from the rest of the data in password-protected files, until the information is needed again for follow-up data collection rounds. The purpose of collecting identifying information is solely to allow for follow-up.

When analyzing or transmitting data, only the participant ID will be available. Matching IDs to names will only be possible for the senior research team with access to the files. Data collectors will temporarily have access to this information as needed for data collection, but will not retain any of it.

Once identifying information has been removed from the data files, the data will be available for analysis and considered joint intellectual property between RWAMREC, Promundo, and the project funders. After initial findings have been analyzed, the dataset will be available by request, with priority to Rwandan researchers.

In the event that collecting data on tablets proves not to be feasible, survey data will be collected on paper, and similar procedures for data management will be used (separating identifying data from the rest of the survey, creating a unique ID, etc.). Paper surveys and identifying information would be kept separately in locked cabinets in the RWAMREC office in Kigali.

Qualitative data will be captured as notes and quotes on paper or tablet. They will similarly be assigned an ID and separated from the rest of the data in password-protected files.

*5.8. Proposed analysis*

The purpose of the study is to evaluate the impact of MenCare+/Bandebereho fathers' groups by comparing

study participants randomly assigned to the intervention and control groups on several key outcomes (described above), over time. Proposed analyses include bivariate and multivariate "difference in difference" regressions to determine the effect, if any, of the intervention on various outcomes.

Qualitative data analysis will consist of careful reading and theme-based coding of interview notes and transcripts.

## 6. Ethical considerations

### *6.1. Confidentiality*

Confidentiality is a priority for the research team. To ensure confidentiality, the research team will not share names or other identifying information of study participants without explicit permission for project purposes. Interviewers will also be specifically trained on privacy and confidentiality. As described in section 5.7, identifying information will be kept in password protected files or locked cabinets, separate from participants' survey and interview responses, and data files will be stripped of any identifying information prior to sharing with others over email or other electronic means.

### *6.2. Informed consent*

We will seek informed consent from all participants in the study. Due to low literacy levels among participants, the interviewer will read the consent form to the participant and answer any questions that they may have. The participant can then sign or provide a thumbprint to indicate they have given their consent to participate in the study. In instances where participants prefer to be accompanied by another individual, who is literate and can read and explain to them the consent form, the signature of the witness will also be recorded.

During the explanation of the study and consent process, participants will be provided with general information about the study and the intervention (as well as contact information for study personnel), informing them of possible benefits and costs, as well as their right not to participate, or to withdraw from the study at any point without any repercussions. More information on the informed consent is available in the Appendix section of this protocol.

### *6.3. Ethical approval*

The research team seeks ethical approval from the Rwanda Health Research Commission, which best understands the specific ethical norms and considerations in Rwanda. The protocol has been reviewed and approved by the National Health Research Committee in August 2014 (approval attached as appendix). Please also note that many of the questionnaire items are derived from IMAGES, a study that was reviewed and approved by the Institutional Review Board at the International Center for Research on Women. The implementation of IMAGES in Rwanda in 2010 was also approved by the Rwanda National Ethics Committee.

## 7. Logistics

### *7.1. Distribution of responsibilities*

**Principal Investigators:** Dr. Fidele Ngabo; Shamsi Kazimbaya; Kate Doyle; Gary Barker and Ruti Levtov

will be responsible for all aspects of the evaluation, including coordination, development of materials, and ensuring research goals are met in a timely manner and within the confines of the budget. The principal investigators will be responsible for recruiting and training local data collectors (as yet unidentified) who will be responsible for collecting the primary data, under the supervision of RWAMREC and Promundo-US. The research team will coordinate closely with the staff of RWAMREC implementing the MenCare+ project in all four districts.

**RWAMREC Staff:** RWAMREC is the primary implementing partner of the MenCare+/Banderebeho project, with support from Promundo-US. During the development of the questionnaire, RWAMREC staff have provided their input on areas of impact to be measured, based on their experience of implementing the fathers groups in local communities, and translated all research materials. RWAMREC staff will trained on impact evaluation methods so that they can participate and contribute to the evaluation, and in future be able to lead their own evaluation.

**Promundo-US Staff:** Promundo is an organization international recognized for its research experience on men, masculinities and gender equality. The research team has an in country representative (Kate Doyle) who has worked closely with the RWAMREC MenCare+ team. Analysis will be undertaken by Promundo-US, with frequent consultations on interpretation of findings with RWAMREC. Promundo-US is providing the funding for the evaluation and will prioritize capacity building of local researchers and RWAMREC staff during the implementation of the evaluation.

**Ministry of Health, MCH Unit:** The Ministry of Health is a key partner on the MenCare+/Bandebereho project and has provided advice and guidance during the development of the project, as well as ongoing support during its implementation. The MCH Unit will continue to be a key partner in the evaluation of the fathers group education and will be apprised at all stages of the research.

## 8. References


1. GOR. (2013) National Community Health Strategic Plan July 2013-June 2018. Kigali: Government of Rwanda (GOR), Ministry of Health.

2. GOR. (2012) Third Health Sector Strategic Plan July 2012 – June 2018. Kigali: Government of Rwanda (GOR), Ministry of Health.

3. GOR. (2012) Family Planning Policy. Kigali: Government of Rwanda (GOR), Ministry of Health.

4. Martin, IA, Hamilton, BE, Sutton, PD, Ventura, SI, Menacker, F, Kirmeyer, S et al. (2007) Births: Final data for 2005. National Vital Statistics Reports 56(6), 1-103.

5. Teitler, JO. 2001 (2001). Father involvement, child health and maternal health behavior. *Children and Youth Services Review* 23(4/5): 403-424.

6. Anderson, BJ and K Standley. (1976) A methodology for observation of the childbirth experience. Paper presented at the meeting of the American Psychological Association, Washington, D.C; cited in Fatherhood Institute. (2007) Fatherhood Institute Research Summary: Fathers Attending Births. United Kingdom: Fatherhood Institute.

7. Henneborn, WJ and R Cogan. (1975) The effect of husband participation in reported pain and the probability of medication during labor and birth. *Journal of Psychosomatic Research* 19: 215-222; cited in Fatherhood Institute. (2007) Fatherhood Institute Research Summary: Fathers Attending Births. United Kingdom: Fatherhood Institute.

8. Tarkka, MJ, Paunonen, M and P Laippala. (2000) Importance of the Midwife in the First-time Mother's Experience of Childbirth. *Scandinavian Journal of Caring Science* 14: 184–190; cited in Fatherhood Institute. (2007) Fatherhood Institute Research Summary: Fathers Attending Births. United Kingdom: Fatherhood Institute.

9. Bankole, A and S. Singh. (1998) Couples' Fertility and Contraceptive Decision-Making In Developing Countries: Hearing the Man's Voice. *International Family Planning Perspectives* 24(1): 15-24.

10. Ezeh, A.C. (1993) The influence of spouses over each other's contraceptive attitudes in Ghana. *Studies in Family Planning* 24(3): 163–174.

11. Speizer, IS. (1999) Are husbands a barrier to women's family planning use? The case of Morocco. *Social Biology* 46(1–2): 1–16.

12. National Institute of Statistics of Rwanda (NISR), Ministry of Health (MOH), and ICF International. (2011) *Rwanda Demographic and Health Survey 2010*. Calverton, Maryland, USA: NISR, MOH, and ICF International.

13. Toure, L. (1996) Male Involvement in Family Planning: A Review of Selected Program Initiatives in Africa. SARA Project, funded by USAID.

14. Irani, L., Speizer I.S. and J. Fotso. (2014) Relationship Characteristics and Contraceptive Use Among Urban Couples in Kenya. *International Perspectives on Sexual and Reproductive Health* 40(1): 11-20.

15. Link, C. (2011) Spousal Communication and Contraceptive Use in Rural Nepal: An Event History Analysis. *Studies in Family Planning* 42(2): 83-92.

16. WHO (2005) Addressing violence against women and achieving the Millennium Development Goals. Geneva: World Health Organization. http://www.who.int/gender/documents/women_MDGs_report/en/index.html.

17. Mullany, B, Becker S, and MJ Hindin (2007) The impact of including husbands in antenatal health education services on maternal health practices in urban Nepal: Results from a randomized controlled trial. *Health Education Research* 22(2): 166-176.

18. Sarkadi, A, Robert K, Frank O and S Bremberg. (2008) Fathers' involvement and children's developmental outcomes: a systematic review of longitudinal studies. *Acta Paediatrica* 97(2): 153-158.

19. Bartlett, E. (2004) The effects of fatherhood on the health of men: A review of the literature. *Journal of Men's Health and Gender* 1(2): 159-169

20. Barker, G, Contreras, JM, Heilman, B, Singh, A, Verma, R and M Nascimento. (2011). Evolving Men: Initial Results from the International Men and Gender Equality Survey (IMAGES). Washington, DC: International Center for Research on Women (ICRW) and Rio de Janeiro: Instituto Promundo.
